# Phylogenomic analysis of Copepoda (Arthropoda, Crustacea) reveals unexpected similarities with earlier proposed morphological phylogenies

**DOI:** 10.1186/s12862-017-0883-5

**Published:** 2017-01-19

**Authors:** Seong-il Eyun

**Affiliations:** Center for Biotechnology, University of Nebraska-Lincoln, Lincoln, NE 68588 USA

**Keywords:** Copepoda, Crustacea, Arthropoda, Phylogeny, Phylogenomics, Divergence time

## Abstract

**Background:**

Copepods play a critical role in marine ecosystems but have been poorly investigated in phylogenetic studies. Morphological evidence supports the monophyly of copepods, whereas interordinal relationships continue to be debated. In particular, the phylogenetic position of the order Harpacticoida is still ambiguous and inconsistent among studies. Until now, a small number of molecular studies have been done using only a limited number or even partial genes and thus there is so far no consensus at the order-level.

**Results:**

This study attempted to resolve phylogenetic relationships among and within four major copepod orders including Harpacticoida and the phylogenetic position of Copepoda among five other crustacean groups (Anostraca, Cladocera, Sessilia, Amphipoda, and Decapoda) using 24 nuclear protein-coding genes. Phylogenomics has confirmed the monophyly of Copepoda and Podoplea. However, this study reveals surprising differences with the majority of the copepod phylogenies and unexpected similarities with postembryonic characters and earlier proposed morphological phylogenies; More precisely, Cyclopoida is more closely related to Siphonostomatoida than to Harpacticoida which is likely the most basally-branching group of Podoplea. Divergence time estimation suggests that the origin of Harpacticoida can be traced back to the Devonian, corresponding well with recently discovered fossil evidence. Copepoda has a close affinity to the clade of Malacostraca and Thecostraca but not to Branchiopoda. This result supports the hypothesis of the newly proposed clades, Communostraca, Multicrustacea, and Allotriocarida but further challenges the validity of Hexanauplia and Vericrustacea.

**Conclusions:**

The first phylogenomic study of Copepoda provides new insights into taxonomic relationships and represents a valuable resource that improves our understanding of copepod evolution and their wide range of ecological adaptations.

**Electronic supplementary material:**

The online version of this article (doi:10.1186/s12862-017-0883-5) contains supplementary material, which is available to authorized users.

## Background

Copepods represent the largest biomass of all animals on earth [1–3]. They are aquatic animals, primarily marine, and make up the dominant zooplankton assemblages in nearshore environments [2, 3]. In spite of their critical ecological roles, the taxonomic classification has received poor attention. Copepods exhibit extreme morphological diversity and occupy an enormous range of habitats in the aquatic realm, from freshwater to hypersaline, shallow pool, and cave to deep sea environments [4–6]. Humes [1] described that there are 11,302 species (198 families, 1633 genera; as of the end of 1993) and estimated that a hypothetical total of 75,347 species may exist on the planet [1]. Copepods are also particularly notorious for cryptic speciation [7–10].

**Fig. 1:**
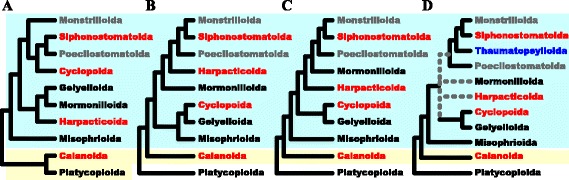
Major phylogenetic hypotheses based on morphological characters of copepod orders, redrawn from A) Ho [11], B) Huys and Boxshall [5], C) Ho [12], and D) Ho et al. [13]. Cyan and yellow boxes indicate the superorders, Podoplea and Gymnoplea [67]. After Huys and Boxshall [5], Platycopioida is classified as a newly proposed Infraclass, Progymnoplea. A new order, Thaumatopsylloida (indicated by blue) is proposed by Ho et al. [13]. Poecilostomatoida and Monstrilloida (indicated by grey) are considered as the subgroup of Cyclopoida and Siphonostomatoida, respectively [16, 19, 20]. Grey dotted lines depict the ambiguous phylogenetic relationships from Ho et al. [13]. Four copepod orders (indicated by red) are examined in this study

Traditionally, there are ten orders of the subclass Copepoda Milne-Edwards, 1840 containing a large different number of families, genera, and species [5]. The morphological phylogenetic analyses of Copepoda have been extensively investigated and there are general agreements such as the monophyletic status of Copepoda [5, 11–14]. Furthermore, copepods can be divided into two infraclasses, Progymnoplea and Neocopepoda [5]. Progymnoplea contains only one order (Platycopioida) and Neocopepoda can be further classified into two superorders, Gymnoplea and Podoplea [5, 12]. For several decades, however, the phylogenetic relationships among the copepod orders have been a matter of controversy [5, 11–17]. Due to an extreme diversity of body forms, the phylogenetic relationships based on traditional morphological data have led to much controversy (see Fig. 1). For example, Ho [11] and Huys and Boxshall [5] analyzed 21 and 54 morphological characters across ten copepod orders [5, 11]. They agreed that Platycopioida and Calanoida were the most basal groups (Fig. 1ab). However, the cladogram from Ho [11] depicted Harpacticoida and Gelyelloida were closely related, but this group was a distinct cluster to the group of Siphonostomatoida, while that of Huys and Boxshall [5] appeared that Harpacticoida had a close affinity to a sister-group of Siphonostomatoida but a discrete to Gelyelloida. Later, some modifications for the morphological phylogenetic models have been proposed [12, 13]. However, as Ho et al. [13] pointed out, the inconsistent position of Harpacticoida that represents an important ecological group in aquatic environments has been still problematical [13].

**Fig. 2:**
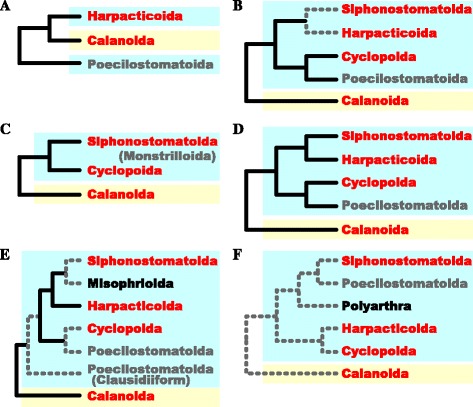
Phylogenetic hypotheses based on molecular sequence data of copepod orders, redrawn from **a**) Braga et al. [18], **b**) Huys et al. [19], **c**) Huys et al. [20], **d**) Minxiao et al. [23], E) Tung et al. [21], and F) Schizas et al. [22]. Phylogenetic trees using **a**) the large subunit ribosomal RNA (28S rRNA) gene (a total aligned sequence length of 484 bps from the D9/D10 region) [18], **b**) the small subunit ribosomal RNA (18S rRNA) gene (a total aligned sequence length of about 1,882 bp) [19], **c**) 18S rRNA (a total aligned sequence length of about 1,941 bp) [20], **d**) the concatenated twelve mitochondrial genes [23], E) 18S rRNA [21], and F) 28S rRNA (505 bp from the v-x region) [22]. Poecilostomatoida and Monstrilloida (indicated by grey) are considered as the subgroup of Cyclopoida and Siphonostomatoida, respectively [16, 19, 20]. In this study, four copepod orders (indicated by red) are examined. Cyan and yellow boxes indicate Podoplea and Gymnoplea, respectively. Grey dotted lines indicate the low bootstrap values (<60%)

Furthermore, some molecular-based studies were not congruent with morphological evidence (Fig. 2). Braga et al. [18] focused on the phylogenetic relationships within the copepod family Euchaetidae and also showed the three copepod orders (Harpacticoida, Calanoida, and Poecilostomatoida with a barnacle, *Semibalanus balanoides* as an outgroup) using the large subunit ribosomal RNA (28S rRNA) gene (a total aligned sequence length of 484 bp) [18]. The tree appeared to be markedly inconsistent with morphological phylogenies; Harpacticoida was closer to Calanoida than to Poecilostomatoida, which was in conflict to the superorder Podoplea (Fig. 2a). Later, other molecular studies recovered and supported the monophyletic podoplean group using the 18S small subunit ribosomal RNA gene (18S rRNA), but still unresolved the phylogenetic position of Harpacticoida (Fig. 2) [19–22]. Recent study using concatenated twelve mitochondrial genes showed that Harpacticoida (*Tigriopus californicus*) was more closely related to Siphonostomatoida (*Lepeophtheirus salmonis* and *Caligus rogercresseyi*) than Calanoida (*Calanus sinicus*) (Fig. 2d) [23]. This mitochondrial phylogenetic hypothesis was generally congruent with the majority of the morphological phylogenies [5, 12, 13] except for the phylogenetic position of Poecilostomatoida (Fig. 2d). Moreover, in the 18S rRNA gene trees of Poecilostomatoida, the Clausidiiform complex and the remaining poecilostomatoid taxa appeared to be paraphyletic (Fig. 2e) [21, 24]. Harpacticoida also may be a paraphyletic taxon with Polyarthra (consisting of the families Canuellidae and Longipediidae) and Oligoarthra (all remaining harpacticoid families) [17, 22, 25]. From the 28S rRNA gene tree (505 bp from the v-x region), two Polyarthra taxa (*Canuella perplexa* and *Longipedia gonzalezi*) were more closely related to other copepods than to Oligoarthra (Fig. 2F) [22]. All these molecular phylogenetic studies used a relatively short length of the sequences (<2,000 bp) or fast evolving genes that were not acceptable for interordinal relationships (Fig. 2; see details in Discussion).

The purpose of the present study was therefore to clarify the phylogenetic relationships among four major orders of copepods using phylogenomics, the inference of phylogenetic relationships using genome-scale data which has increasingly become a powerful tool to resolve difficult phylogenetic questions [26–30]. In particular, the aim was to include the following: 1) an extensive analysis of the phylogenetic position of Harpacticoida and to evaluate all possible phylogenetic hypotheses; 2) the phylogenetic relationships of copepods among other crustacean groups; and 3) the divergence times of the major copepod orders. Accordingly, the orthologous sequences of 24 nuclear protein-coding genes were retrieved from 18 arthropod species representing four copepod orders (nine species), five other crustaceans (Anostraca, Cladocera, Thecostraca, Amphipoda, and Decapoda), two insects, and two closely related outgroups (Myriapoda and Chelicerata). This study was the first report that provides a rich taxon sampling with genomics-based evidence focusing on the evolution of copepods and their divergence time. Thus, for an ecological perspective, understanding the phylogenetic relationships of copepods would have provided a first step toward elucidating an ecological interaction, habitat colonization, and speciation in Copepoda.

## Methods

### Taxonomic sampling and identification of orthologous genes

The genome and transcriptome assemblies for 18 arthropod species were obtained from multiple sources (see below). For eight copepod species, five transcriptome (*Caligus rogercresseyi*, *Lernaea cyprinacea*, *Tigriopus californicus*, *Calanus sinicus*, and *Acartia fossae*) and three genome sequences (*Lepeophtheirus salmonis*, *Mesocyclops edax*, and *Calanus finmarchicus*) were downloaded from the National Center for Biotechnology Information (NCBI) Sequence Read Archive (SRA) database (http://www.ncbi.nlm.nih.gov/sra) [31, 32]. Three additional crustacean species were also included: the giant tiger prawn *Penaeus monodon* (Malacostraca: Penaeidae), the purple barnacle *Amphibalanus amphitrite* (Thecostraca: Balanidae), and the brine shrimp *Artemia franciscana* (Branchiopoda: Artemiidae) from NCBI SRA. Three other crustacean genome sequences (two copepods and an amphipod; *Eurytemora affinis*, *Tigriopus californicus*, and *Hyalella azteca*) were downloaded from Baylor College of Medicine Human Genome Sequencing Center (BCM-HGSC), as a part of the pilot project for the i5K arthropod genomes project [33]. Among these crustacean species examined, none of the orthologous sequences for the 24 nuclear protein-coding sequences (see below) was identified in *Calanus finmarchicus* which was excluded from further analysis. Also, the orthologous sequences were further retrieved from the non-redundant (NR) protein database at NCBI (http://www.ncbi.nlm.nih.gov). All orthologous sequences from the copepod *Acanthocyclops vernalis* were obtained from NCBI NR database. All orthologous sequences identified in this study and the GenBank accession numbers were summarized in Additional file 1: Table S1. In addition to the crustacean species mentioned above, five publicly released genomes were added in this study. These sequences of the water flea *Daphnia pulex* (Branchiopoda), the fruit fly (*Drosophila melanogaster*), the red flour beetle (*Tribolium castaneum*), the centipede *Strigamia maritima* (Myriapoda), and blacklegged tick *Ixodes scapularis* (Chelicerata) were downloaded from the wFleaBase (http://wfleabase.org), FlyBase (http://flybase.org), BeetleBase (http://beetlebase.org), BCM-HGSC (https://www.hgsc.bcm.edu), and VectorBase (https://www.vectorbase.org), respectively [34–37].

The previously reported nuclear protein-coding genes that were used for the phylogenetic analysis were retrieved as search queries. These sequences were obtained from Regier et al. [28] and Wiegmann et al. [38]. The orthologous genes were defined by the Basic Local Alignment Search Tool (BLAST, ver. 2.2.30+) programs [39, 40]. The E-value threshold of 1 × 10^−30^ with the database size 1.4 × 10^10^ was used to identify orthologous candidates against the genome and transcriptome assemblies. The putative orthologous genes were verified by searches using tblastn against NCBI NR database. After partial sequences or no apparent orthologs were excluded from the analysis, 24 nuclear protein-coding genes were then determined in more than half of the copepod species (Additional file 1: Table S1). All identified copepod protein and nucleotide sequences are provided in Additional files 2 and 3.

### Multiple sequence alignments

Multiple alignments of each of the protein gene families were generated using mafft (ver. 7.245) [41] with the L-INS-i algorithm (1,000 maxiterate and 100 retree) which uses a consistency-based objective function and local pairwise alignment with affine gap costs. Alignments were adjusted manually when necessary. Poorly aligned regions with more than 70% of gaps were removed using trimAl (ver. 1.2) [42]. The corresponding coding nucleotide alignments were generated using PAL2NAL [43]. The single gene sequence alignments are available in: http://bioinformatics.unl.edu/eyun/Copepoda_Phylogenomics. All sequences were concatenated using a custom Perl script (ConCat_seq.pl). This Perl script is available upon request from the author. The concatenated dataset used in this study is available in Additional file 4.

### Phylogenetic analysis and alternative topology tests

Phylogenetic relationship using the concatenated sequences was reconstructed by the maximum-likelihood (ML) method with the Le and Gascuel (LG) matrix, gamma distributed rates, invariant sites, and the observed amino acid frequencies using PhyML (ver. 3.1) [44–46]. The best-fit model for the concatenated dataset was selected using the Akaike Information Criterion (AIC) as a statistical tool in ProtTest (ver. 3.2) [47]. Non-parametric bootstrapping with 1000 pseudo-replicates was used to estimate the confidence of branching patterns for the ML phylogeny [48]. Bayesian inferences (BI) of phylogeny were performed using MrBayes (ver. 3.2.6) [49] with the LG substitution model, gamma-distributed rate variation, and invariant sites. The Markov chain Monte Carlo search was run for 5 × 10^6^ generations, with a sampling frequency of 10^3^, using three heated and one cold chain and with a burn-in of 10^3^ trees.

The phylogenetic trees were also reconstructed using the “degen-1” coding sequences, in which nucleotides at any codon position that have the potential of synonymous substitutions were degenerated [28]. To produce the degenerated synonymous matrices (the “degen-1” coding sequences) [28], the Perl script (Degen_v1_4.pl) written by Andreas Zwick and April Hussey was used (http://www.phylotools.com). For the morphological reanalysis, the data matrix of 54 morphological characters was obtained from Ho et al. [13]. This morphological data matrix in Nexus format is available in Additional file 5. Phylogenetic inference of the morphological data was conducted with MrBayes (ver. 3.2.6) [49], using the Mk (Markov *K*) model [50], a variable rate among characters (“rates = gamma”), and 5 × 10^8^ generations. The Mk model assumes equal state frequencies. In this analysis, trees were sampled every 10^3^ generations with the first 25% discarded as burn-in and summarized using a 50% majority rule consensus tree. Presentation of the phylogenies was done with FigTree (ver. 1.4.2) (http://tree.bio.ed.ac.uk/software/figtree).

The Kishino-Hasegawa (KH) [51], the Shimodaira-Hasegawa (SH) [52], and the Approximately Unbiased (AU) [53] tests were used to statistically assess the phylogenetic hypotheses. The site log-likelihood of each tree was calculated in TREE-PUZZLE (ver. 5.3.rc16) [54], and KH, SH, and AU tests were performed in CONSEL (ver. 0.20) with default options [55].

### Divergence time estimation

The divergence times of lineages were estimated using BEAST2 (ver. 2.4.3) [56] with Bayesian inference using the calibrated Yule model for the tree prior and the uncorrelated relaxed clock model proposed by Drummond et al. [57]. BEAST2 was using a random tree with 5 × 10^7^ generations and a sample frequency of 5 × 10^3^ generations. Four fossil-based minimum ages were applied for the major splits; 497 MYA for the Diptera-Cladocera divergence, 405 MYA for the Cladocera-Anostraca divergence, 313.7 MYA for the Diptera-Coleoptera divergence, and 358.5 MYA for the Amphipoda-Decapoda divergence [58]. The fossil record of *Wujicaris muelleri* Zhang et al. [59] was also used as the minimum constraint on the crown group of Pancrustacea [58–60].

## Results

### Monophyly of copepods and their interordinal relationships

**Fig. 3:**
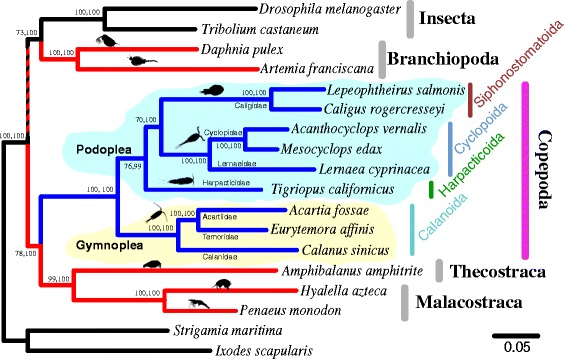
The maximum-likelihood phylogeny of nine copepod species and nine other arthropod species based on the 24 nuclear protein-coding genes. *Strigamia maritima* (Myriapoda) and *Ixodes scapularis* (Chelicerata) are used as the outgroups. Blue-colored and red-colored branches indicate the copepod groups and all other crustaceans. The numbers at internal branches show the bootstrap support values (%) for the maximum-likelihood phylogeny and the posterior probability (%) for the Bayesian phylogeny in this order. The scale bar represents the number of amino acid substitutions per site

24 nuclear protein-coding genes were obtained from 18 arthropod species including nine copepod species (four major orders of copepods) (Additional file 1: Table S1). The common names of species examined with the current taxonomic classification were listed in Table 1. Among 18 arthropods, two non-pancrustacean taxa, the centipede *Strigamia maritima* and blacklegged tick *Ixodes scapularis*, were used as the outgroups [28, 61]. All 24 nuclear protein-coding sequences were concatenated for further phylogenetic analyses (see details in Methods). The data set of the concatenated sequences consisted of 16,710 amino acid sequences (50,106 bp). The phylogenetic relationships obtained from the concatenated sequences were reconstructed by the maximum-likelihood (ML) and Bayesian inferences (BI). The two algorithms confirmed the monophyly of copepods with 100% bootstrap values (Fig. 3). The nine copepod species examined can be classified into two superorders, Gymnoplea (Calanoida) and Podoplea (Siphonostomatoida, Cyclopoida, and Harpacticoida) (Fig. 1; see Discussion). The phylogenomic analyses with ML and BI generated the same topologies supporting the monophyly of the podoplean group. The superorder Podoplea was strongly supported with the high maximum likelihood bootstrap value (MLB = 100%) and Bayesian posterior probability (BPP = 1.00) (Fig. 3).

**Table 1:** Taxonomic classification used in this study

[Class] / Species	Order and Family	Common Names
[Copepoda]		
*Lepeophtheirus salmonis*	Siphonostomatoida, Caligidae	salmon louse
*Caligus rogercresseyi*	Siphonostomatoida, Caligidae	sea louse
*Acanthocyclops vernalis*	Cyclopoida, Cyclopidae	
*Mesocyclops edax*	Cyclopoida, Cyclopidae	freshwater cyclopoid
*Lernaea cyprinacea*	Cyclopoida, Lernaeidae	anchor worm
*Tigriopus californicus*	Harpacticoida, Harpacticidae	tide pool copepod
*Acartia fossae*	Calanoida, Acartiidae	Oceanic shelf copepod
*Eurytemora affinis*	Calanoida, Temoridae	common estuarine copepod
*Calanus sinicus*	Calanoida, Calanidae	Asian Pacific copepod
[Thecostraca]		
*Amphibalanus amphitrite*	Sessilia, Balanidae	purple acorn barnacle
[Malacostraca]		
*Hyalella azteca*	Amphipoda, Dogielinotidae	
*Penaeus monodon*	Decapoda, Penaeidae	giant tiger prawn
[Branchiopoda]		
*Daphnia pulex*	Cladocera, Daphniidae,	water flea
*Artemia franciscana*	Anostraca, Artemiidae	brine shrimp
[Insecta]		
*Drosophila melanogaster*	Diptera, Drosophilidae	fruit fly
*Tribolium castaneum*	Coleoptera, Tenebrionidae	red flour beetle
[Chilopoda]		
*Strigamia maritima*	Geophilomorpha, Geophilidae	
[Arachnida]		
*Ixodes scapularis*	Ixodida, Ixodidae	blacklegged tick

Most notably, within Podoplea, the interordinal relationships inferred from the phylogenomic analysis differed from that of the widely accepted hypothesis presented in the majority of the morphological and molecular phylogenetic studies that Harpacticoida was generally affiliated with Siphonostomatoida rather than with Cyclopoida [5, 12, 19, 21, 23] (Figs. 1 and 2; see Discussion). In addition to order level relationships in this study, all family and genus level relationships were also clearly resolved by high bootstrap values (MLB = 100% and BPP = 1.00) (Fig. 3). This study included three families for Calanoida: ((Acartiidae, Temoridae), Calanidae) and three genera in Cyclopoida: ((*Acanthocyclops*, *Mesocyclops*), *Lernaea*).

### Phylogenetic position of Harpacticoida

To confirm the phylogenetic position of Harpacticoida (*Tigriopus californicus*, Oligoarthra), four different phylogenetic analyses were attempted; 1) Bayesian reestimation of morphological characters, 2) the “degen-1” coding sequences of Regier et al. [28], 3) small-scale phylogeny dealing with only nine copepod species with two closely related outgroups, and 4) statistical analyses were performed for all possible trees (three topologies here). First, the morphological phylogeny was reconstructed by Bayesian inferences (Additional file 6: Figure S1). Bayesian analysis using 54 morphological characters showed the same topology with that of Ho et al. [13] which was reconstructed by maximum parsimony. This tree yielded mostly congruent results with the majority of the copepod phylogeny above. However, all posterior probabilities with the morphology-only data set showed a general lack (BPP < 0.80) of support except for three nodes (indicated by boldfaces in Additional file 6: Figure S1). This suggested that these morphological data might not have sufficient phylogenetic signal. For the second and third attempts, the phylogenetic trees were reconstructed using the “degen-1” coding sequences and from only nine copepod species with *Amphibalanus amphitrite* (Sessilia) and *Penaeus monodon* (Decapoda) as the outgroups. Both the phylogenetic approaches returned the same topology as that obtained from Fig. 3 (Additional files 7 and 8: Figures S2 and S3). The phylogenetic relationships were less resolved (>64% MLB for the clade of Siphonostomatoida and Cyclopoida) using the “degen-1” nucleotide dataset than that of Fig. 3, but better resolved in the small-scale phylogeny by high bootstrap values (>76% MLB for that clade) (Additional files 7 and 8: Figures S2 and S3). Lastly, to evaluate those previously proposed hypotheses shown in Figs. 1 and 2, statistical analyses were performed using TREE-PUZZLE (ver. 5.3.rc16) and CONSEL (ver. 0.20) [54, 55] (see in Methods). In Table 2, the first hypothesis, as mentioned earlier, was obtained from the majority of the copepod phylogeny. The second hypothesis was the best maximum likelihood tree obtained from the concatenated PhyML tree in this study. The third hypothesis was a theoretical tree, in which Harpacticoida was closely related to Cyclopoida. All statistical tests rejected the third hypothesis. Although the KH test (*P* = 0.109) and the SH test (*P* = 0.479) were unable to reject the first hypothesis, the AU topology test was marginally rejected (*P* = 0.085) at the 0.10 level of significance. This was most likely due to the conservative nature of the KH test and the SH test. The KH test was invalid in this case because the second hypothetical tree was the best ML tree [52]. The SH test is the most conservative estimate and is sensitive to the unlikely tree (i.e., the third hypothesis in Table 2) [62]. Among the three tests, the AU test is known as the best approach to overcome these problems [53]. Thus, the results of the statistical test supported that the most likely phylogenetic scenario is the second hypothesis. Taken together, these results strongly suggested that Siphonostomatoida was closer to Cyclopoida than Harpacticoida.

**Table 2:** Statistical comparisons between the best ML tree and alternative phylogenetic hypotheses within podopean copepods

Hypothetical Affinities^a^	References claiming the hypothesis	-lnL^b^	*P*-values		
			KH^c^	SH^d^	AU^e^
((SI, HA), CY)	Huys and Boxshall (1991) [5], Ho (1994) [12], Huys et al. (2006) [19], Minxiao et al. (2011) [23], and Tung et al. (2014) [21]	177,619.7	0.109	0.479	0.085*
((SI, CY), HA)	Kabata (1979) [68], Ho (1990) [11], and Dahms (2004) [14]	177,399.7	0.377	0.750	0.445
((HA, CY), SI)	none	177,631	<0.001***	<0.001***	<0.001***

^a^SI = Siphonostomatoida, CY = Cyclopoida, and HA = Harpacticoida

^b^lnL = Log-likelihood scores

^c^ *P*-value of the Kishino-Hasegawa (KH) test [51]

^d^ *P*-value of the Shimodaira-Hasegawa (SH) test [52]

^e^ *P*-value of the Approximately Unbiased (AU) test [53]

One (*) and triple (***) asterisks denoted statistical significance at the 0.10 and 0.001 level, respectively

### Copepoda is a sister group to Communostraca

According to the present phylogenomic analysis, the resulting trees revealed that Copepoda was a sister lineage to a group of Thecostraca and Malacostraca but distinct to Branchiopoda (Fig. 3 and Additional file 7: Figure S2), consistent with results from Regier et al. [28] and Oakley et al. [30]. Both ML and BI inferred the following interclass relationships: ((Insecta, Branchiopoda), (Copepoda, (Thecostraca, Malacostraca))). The purple barnacle *A. amphitrite* (Sessilia: Balanidae) was considered to be a sister group to copepods, namely Maxillopoda. In this study, however, this species appeared to be a sister group to the group (Malacostraca) of *H. azteca* (Amphipoda) and *P. monodon* (Decapoda), but distinctly related to copepods (Fig. 3 and Additional file 7: Figure S2). The MLB and BPP values for the clade of Sessilia, Amphipoda, and Decapoda were highly supported (MLB > 87% and BPP = 1.00) (Fig. 3 and Additional file 7: Figure S2). Therefore, this study supported the newly proposed clade, Communostraca (common shelled ones) that includes Malacostraca (e.g., crabs or shrimp) and Thecostraca (e.g., barnacles) and the newly proposed clade, Multicrustacea (Copepoda, Malacostraca, and Thecostraca) with high support values (MLB > 71% and BPP = 1.00) [28] (See Discussion).

This study also supported a proposed clade of Insecta and Branchiopoda, consistent with results from Oakley et al. [30] representing the Allotriocarida (Hexapoda/Branchiopoda/Remipedia) clade [30]. A very recent study also supported the monophyly of Allotriocarida [63]. Branchiopoda (a group of Cladocera and Anostraca) was considered to belong to the subphylum Crustacea. However, this group was more closely related to Insecta but distinct to all other crustaceans examined in this study. Although the MLB value for the clade of Allotriocarida (Insecta and Branchiopoda) was not very strong (>73% in Fig. 3 and > 62% in Additional file 7: Figure S2), this hypothesis is often congruent with those obtained from recent studies [27, 30, 64, 65]. Therefore, the phylogenetic trees in this study supported the hypotheses of the three newly proposed clades, Communostraca, Multicrustacea, and Allotriocarida, but challenged the validity of Hexanauplia and Vericrustacea (See Discussion).

### Estimation of divergence time in Copepoda

**Fig. 4:**
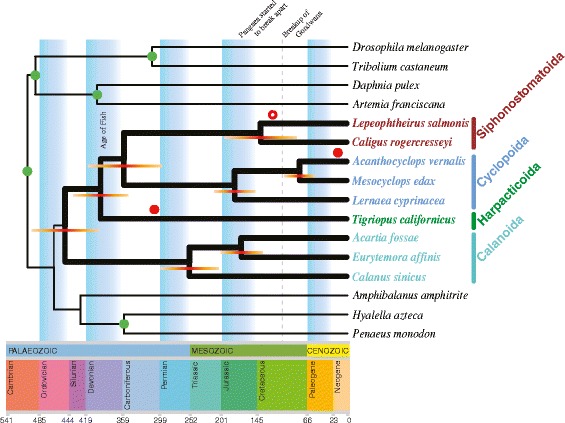
Estimated divergence times among copepods. BEAST2 (ver. 2.4.3) [56] is used with five calibration points (indicated by green circles, see details in Methods). Orange bars across nodes indicate 95% highest posterior density (HPD) of the Bayesian posterior distribution of molecular time estimates. The open and closed red circles (the parasitic and free-living forms of copepod fossils) above the branches show the oldest fossil records, most likely corresponding to the copepod orders [84–87]. The geologic time scale is according to the International Chronostratigraphic Chart (http://www.stratigraphy.org, v2015/01)

Divergence times were estimated using BEAST2 (ver. 2.4.3) [56] with Bayesian inference. The tree topology was the same as the PhyML tree shown in Fig. 3. Divergence between the groups of podopleans and gymnopleans was estimated to have occurred during the period from the late Cambrian to the Devonian (446.2 ± 47.3 MYA). The origin of *T. californicus* appeared to have occurred in the Devonian (between the late Silurian and the early Carboniferous, 381.4 ± 51.1 MYA) (Fig. 4). The divergence time between the two orders Siphonostomatoida and Cyclopoida occurred in the Carboniferous (351.8 ± 58.1 MYA) which predated approximately the origin of Harpacticoida (Fig. 4). Seven extant families in this analysis arose before the Cenozoic era and possibly prior to the early stage of breakup of Gondwana [66].

## Discussion

The present study provides the first phylogenomic evidence to support the monophyletic origin of four major orders of copepods and the group of podopleans. The monophyletic status of Copepoda has been broadly accepted by both morphological [5, 14] and large-scale phylogenomic analyses [28–30]. Although this study does not include all copepod orders, there can be no doubt of the monophyly of copepods. The subclass Copepoda consists of two infraclasses, Progymnoplea and Neocopepoda, suggested by Huys and Boxshall [5]. The infraclass Neocopepoda can be further divided into two superorder groups, Gymnoplea and Podoplea (Fig. 1). The concept of this classification was proposed by Giesbrecht [67] and became generally accepted [5, 12, 68]. However, the naupliar musculature and the molecular phylogeny using partial nuclear 28S rRNA gene (a total aligned sequence length of 484 bp from the D9/D10 region) (Fig. 2A) showed conflicting results and suggested a possible paraphyletic origin of podopleans [15, 18]. Later, morphological [13, 14] and molecular [19, 20, 23] phylogenetic analyses recovered the monophyly of podopleans. In this study, the phylogenomic analysis shows that three podoplean copepod orders are clearly clustered as a monophyletic clade (supported by high bootstrap values, MLB > 99% and BPP = 1.00) (Fig. 3 and Additional files 7 and 8: Figures S2 and S3).

Unexpectedly, the current phylogenomic evidence is in conflict to the majority of the copepod phylogenies (Figs. 1 and 2; see Results). The present schematic phylogeny resemble those found in the earlier phylogenies and postembryonic data [11, 14, 68] which show that Calanoida represents the most basal split among the four copepod orders and that Harpacticoida is the basally-branching group of Podoplea. On the basis on postembryonic apomorphies, naupliar characters can be represented by plesiomorphic states because postembryonic stages (both early and later) provide a valuable resource for evolutionary history [14]. His study implied that Harpacticoida is the more basally-branching group than Misophrioida within podopleans, which is hardly reported in previous studies [5, 11–13, 15, 17]. Interestingly, our preliminary survey based on weighted morphological characters after removing the convergent characters appears that Harpacticoida is the most basally-branching podoplean group (Eyun et al., unpublished data). For example, some morphological characters support the current phylogenomic phylogeny; following the characters from Huys and Boxshall [5], character 11 (male antennulary segment XXIII), character 21 (outer seta on basis of maxillule), and character 54 (seta *b* on exopod of male fifth leg). These morphological characters can be the candidates to investigate the order-level relationships of copepods and morphological transitions (e.g., character 21). Based on character 54 which is absent of in Harpacticoida but is present in Misophrioida and many other podopleans, Harpacticoida seems to be the most basally-branching group within Podoplea. Furthermore, as keenly pointed out by Ho [12], some characters such as character 13 (male antennulary segments XXIV and XXV), character 29 (praecoxal seta on maxilliped), and character 39 (number of setae on inner margin of second endopodal segment of first swimming leg) are confirmed as convergent characters in this study. These implies that differential weighting criteria for the morphological phylogeny [69] and the removal of convergent characters can reduce the phylogenetic noise. In fact, from the preliminary survey removing the convergent characters, the posterior probabilities in Bayesian phylogenetic inference are increased (Eyun et al., unpublished data).

Recent studies have given rise to a new taxonomic classification of Copepoda. Although many progresses have been made toward unraveling the phylogeny and taxonomy of Copepoda, there is so far no consensus of their order-level classification. This should be due to their extreme morphological diversity and a lack of genetic information. Huys and Boxshall [5] summarized ten copepod orders [5]. Ho et al. [13] proposed a new order, Thaumatopsylloida because the family Thaumatopsyllidae was a distinct group from the order Cyclopoida and differed from Monstrilloida and Siphonostomatoida [13]. Boxshall and Halsey [16] suggested that Poecilostomatoida was merged into Cyclopoida [16]. Huys et al. [19], Minxiao et al. [23], and Huys et al. [24] supported this view (but as a sister group) using 18S rRNA and the concatenated twelve mitochondrial genes [19, 23, 24]. Another molecular sequence study using 18S rRNA (a total aligned sequence length of about 1,941 bp) suggested that the order Monstrilloida (indicated by grey in Figs. 1 and 2) was nested within a fish-parasitic clade of the order Siphonostomatoida and thus was considered as the subgroup of Siphonostomatoida [20]. The 18S rRNA gene and 28S rRNA gene trees showed that Poecilostomatoida and Harpacticoida were paraphyletic, respectively (Fig. 2EF) [21, 22, 24].

Some studies have argued that adding more sequences is more important than adding taxa for improved phylogenetic accuracy [70, 71] (but see [72] for the benefits of adding taxa). Indeed, in copepods, insufficient and only partial sequences have been used and showed a limitation for certain order-level [21, 73, 74]. Blanco-Bercial et al. [75] discussed that the use of a single gene at the family or superfamily level of copepods contributed to the disparate results, and the relationships in the superfamily Centropagoidea (Order Calanoida) were still unresolved using the four concatenated genes (18S rRNA, 28S rRNA, cytochrome c oxidase subunit I, and cytochrome *b*) [75]. Therefore, the phylogenomic approach will make notable contributions to a better resolution of copepod evolution and then can be anchored to certain taxonomic clades. Furthermore, the resulting phylogenomic tree can provide an independent test of morphological character homology and can help to determine the assumptions of plesiomorphic or apomorphic characters and the convergent or homoplastic characters, which are considered as the most difficult issue for copepod taxonomy [5, 11, 12].

The class Maxillopoda (Phylum Arthropoda) is one of the most diverse groups of crustaceans including copepods, barnacles, and a number of related animals (such as a branchiuran fish louse and tongue worms) [6]. However, the monophyly of Maxillopoda seemed increasingly doubtful and the maxillopodan concept became obsolete due to the phylogenetic studies of the Arthropoda [27–30, 61, 76, 77]. These studies appear in the polyphyly of Maxillopoda. In addition, the phylogenetic position of copepods in relationship to other crustacean groups has been controversial, resulting a particularly ambiguous resolution of Copepoda, Thecostraca, Malacostraca, and Branchiopoda. Therefore, the phylogenetic relationships among crustaceans are still far from being resolved [78, 79]. Recent phylogenomic studies advocate a new taxonomic nomenclature for the crustacean groups. Regier et al. [28] and Oakley et al. [30] proposed several crustacean classifications; Communostraca (Malacostraca, Thecostraca), Multicrustacea (Copepoda, Malacostraca, and Thecostraca), and Vericrustacea (Copepoda, Malacostraca, Thecostraca, and Branchiopoda) [28] and Allotriocarida (Hexapoda, Remipedia, Cephalocarida, and Branchiopoda) and Hexanauplia (Copepoda and Thecostraca) [30]. From the currently inferred phylogenies including six crustacean groups (Cladocera, Anostraca, Copepoda, Sessilia, Amphipoda, and Decapoda), the tree supports well the hypothesis of the three newly proposed clades, Communostraca, Multicrustacea, and Allotriocarida. However, this study challenges the validity of Hexanauplia and Vericrustacea, corroborating those obtained from other phylogenomic analyses (Fig. 3 and Additional file 7: Figure S2) [27, 29, 30].

This study confirms that the rapidly evolving genes tend to generate the phylogenetic noise [30] and that the slower evolving genes contain more informative positions [80]. For instance, the phylogenies using a single gene tree from 6-phosphogluconate dehydrogenase, carbamoylphosphate synthetase, and alanyl-tRNA synthetase show the non-monophyly of Copepoda (Additional file 9: Figure S4). This may be due to incomplete sequences of genes which are not identified to cover the intact region in this study but also to a relatively high level of sequence variation. Regier et al. [28] also categorizes these genes as the fast evolving genes (the gene numbers: 11, 19, and 23) [26]. Therefore, the phylogenetic signals from the fast evolving genes could generate misleading effects in evolutionary studies [81]. Note that, however, the copepod topology after excluding these genes is same as the one shown above (data not shown).

Divergence between the groups of podopleans and gymnopleans is estimated to have occurred in the very late Ordovician. This implies that the origin of copepods may be earlier (probably Cambrian age) than this period [82, 83]. It is because all copepod taxa in this study belong to the Infraclass Neocopepoda, and Platycopioida (the other infraclass Progymnoplea) is known to be the most primitive group of copepods and possibly closer to the ancestral form [5, 12]. Only few fossil records of copepods are available because of their fragile nature and thus having a very low level of potential fossilization. Divergence time estimations in this study are in good agreement with these known fossil records [84–87]. Recently, a new fossil of freshwater harpacticoids (most likely Canthocamptidae) has been found in carboniferous bitumen, dating back to at least 303 MYA [86]. Interestingly, the origin of *T. californicus* assumed in this study is almost congruent with this fossil record (Fig. 4). The family Canthocamptidae is the largest group (>600 species) of harpacticoids and predominately inhabit fresh water [88]. Boxshall and Jaume [88] speculated that harpacticoids invaded fresh waters on Pangaea based on the pattern of colonization of continental waters. This study supports this hypothesis by molecular sequence analysis. To study the adaptation on the different types of environments (e.g., cave or groundwater) and the timing of colonization events, a strong phylogenetic hypothesis must be established. For the future, comparative genomics of copepod species will help us understanding their evolutionary history and shed light on a wide range of ecological adaptations.

## Conclusion

A series of molecular phylogenetic analyses of nine copepod species with five other crustacean groups, two hexapods, and two outgroups (myriapod and spider) is presented using the 24 orthologous nuclear protein-coding genes. Given the phylogeny, this hypothesis provides an overview of the useful directions for future studies and thus will shed a light into new taxonomic investigations. As more sequences become available in the near future, further studies with more comprehensive taxa are essential to evaluate the various hypotheses as well as fully resolve the evolutionary history and taxonomy of Copepoda. Also, some copepod orders (e.g., Thaumatopsylloida, Monstrilloida, and some groups of Poecilostomatoida and Harpacticoida) need to be refined by further phylogenomic studies. The large scale of molecular data such as genomes and transcriptomes of copepods provides us a valuable resource for understanding copepod evolution and a wide range of ecological adaptations.

## Additional files

Additional file 1:
**Table S1.** Orthologous sequences used and identified in this study. (XLSX 18 kb)
Additional file 2: This file contains the copepod amino acid sequences in FASTA format. (TXT 71 kb)
Additional file 3: This file contains the copepod nucleotide sequences in FASTA format. (TXT 207 kb)
Additional file 4: This file contains the aligned and concatenated dataset used in this study. (DOCX 102 kb)
Additional file 5: This file contains the data matrix of 54 morphological characters from Ho et al. [13] in Nexus format. (DOCX 17 kb)
Additional file 6:
**Figure S1.** Bayesian phylogenetic analysis of copepod orders with morphological characters taken from Ho et al. [13]. (DOCX 63 kb)
Additional file 7:
**Figure S2.** Bayesian phylogeny using the “degen-1” nucleotide coding sequences. (DOCX 172 kb)
Additional file 8:
**Figure S3.** Bayesian phylogeny of nine copepod species with two outgroups. (DOCX 107 kb)
Additional file 9:
**Figure S4.** Maximum-likelihood phylogenies of arthropods focused on copepod species, based on a single gene region from A) 6-phosphogluconate dehydrogenase, B) carbamoylphosphate synthetase, and C) alanyl-tRNA synthetase. (DOCX 185 kb)

## Abbreviations

AU: Approximately unbiased
BI: Bayesian inferences
BPP: Bayesian posterior probability
KH: Kishino-Hasegawa
LG: Le and Gascuel
ML: Maximum-likelihood
MLB: Maximum likelihood bootstrap value
NJ: Neighbor-joining
rRNA: Ribosomal RNA
SH: Shimodaira-Hasegawa
