# Supplementary material for: Phylogenomic analysis of Copepoda (Arthropoda, Crustacea) reveals unexpected similarities with earlier proposed morphological phylogenies
Source: BMC Evol Biol. 2017 Jan 19;17:23. doi: 10.1186/s12862-017-0883-5 (PMC5244711; doi:10.1186/s12862-017-0883-5)
Supplement: Additional file 4: — This file contains the aligned and concatenated dataset used in this study. (DOCX 102 kb) [file 12862_2017_883_MOESM4_ESM.docx]

#NEXUS

[Eyun S (2016) **Phylogenomic analysis of Copepoda (Arthropoda, Crustacea) reveals unexpected similarities with earlier proposed morphological phylogenies**. BMC Evolutionary Biology]

BEGIN DATA;

DIMENSIONS NTAX=18 NCHAR=16710;

FORMAT DATATYPE=PROTEIN INTERLEAVE MISSING=-;

MATRIX

Aver -------------------- -------------------- -------------------- -------------------- --------------------

Csin -------------------- -------------------- -------------------- -------------------- --------------------

Isca -------------------- -------------------- -------------------- -------------------- --------------------

Eaff MD----TSLDAKAIRKIFLD FFIEKKTHIYVHSSSTIPHD DPTLLFANAGMNQFKPVFVG TADPNSDMAKWTRVVNTQKC IRAGGKHNDLDDVGKDVYHH

Lcyp -------------------- -------------------- ----------MNQFKPLFLG TVDPNSEMAKYKRVANTQKC IRAGGKHNDLDDVGKDVYHH

Lsal -------------------- -------------------- -------------------- -------------------- --------------------

Aamp -------------------- -------------------- -------------------- -------------------- --------------------

Afra MC----ANMKASEVRQKYMD FFMKKKSHIYVHSSSTIPHD DPTLLFANAGMNQFKPIFLG TVDPNSEMSKWTRVVNTQKC IRAGGKHNDLDDVGKDVYHH

Hazt --------MLLKEVRSTFVE YFQKKHAHVYVHSSPTIPHD DPTLLFANAGMNQFKPIFLG NVDPSSDMARLKRAVNSQKC IRAGGKHNDLDDVGKDLYHH

Meda -------------------- -------------------- -------------------- -------------------- --------------------

Afos MD----TSLKAAKIRQIFLD FFMQKKEHTYVHSSSTIPLD DPTLLFANAGMNQFKPVFVG TVDPNSDMAKWKRVVNTQKC IRAGGKHNDLDDVGKDVYHH

Dmel MK----L-LTAKEVRNAYLD FFKEQ-KHIYVHSSSTIPLD DPTLLFANAGMNQFKPIFLG TADPNSEMSKWVRVANTQKC IRAGGKHNDLDDVGKDVYHH

Tcas MD----ISMSAKDVRNAYIN FFKEK-NHDYVHSSSTIPLD DPTLLFTNAGMNQFKPIFLG TVDPNSDLAKLTRAVNSQKC IRAGGKHNDLDDVGKDVYHH

Pmon --------MKSSEVRETFYN FFKEKHQHTYVHSSPTIPHD DPTLLFANAGMNQFKPIFLG TVDPSSDMANYVRAVNSQKC IRAGGKHNDLDDVGKDVYHH

Smar MN----ASLTSKQIRQMFLD FFTEKYNHTYVHSSVVIPYD DPTLLFANAGMNQFKPIFLG SVDPNSQMSKWVRAVNTQKC IRAGGKHNDLDDVGKDVYHH

Tcal MSARAPPPTQASEVRQAFMD FFKHSHKHTYWHSSSTIPLD DPTLLFTNAGMCQFKPIFVG SVDPNVDMAKLSRACNSQKC IRAGGKHNDLDDVGKDVYHH

Crog MD----SSMKGDEIRQLYLD FFTKKKGHTYYHSSSTIPLD DPTLLFINAGMNQFKSIFVG TIDPNNDKAKLVRAVNSQKC VRAGGKHNDLEDVGKDSYHH

Dpul MD----TSLSAAKIRQMYMD FFIEKKSHIYVHSSSTIPKD DPTLLFANAGMNQFKPLFLG TVDPNSDMSKWIRVVNTQKC IRAGGKHNDLDDVGKDVYHH

Aver -------------------- -------------------- -------------------- -------------------- ----ETGPCGPCSEIHF---

Csin -------------------- -------------------- -------------------- -------------------- --------------------

Isca -------------------- -------------------- -------------------- -------------------- --------------------

Eaff TFFEMLGNWSFGDYFKKEIC TWSWELLTEVFKLDPARLYV TYFGGDEASGLQPDLEAKQL WLDLGVGESRILPGNMKDNF WEMGDTGPCGPCSEIHY---

Lcyp TFFEMLGNWSFADYFKEEAI RMAMDLLLNEFKIDKERLYA TYFEGNESAGLQPDLETKEI WKKY-LPEDHILKGNMKDNF WEMGETGPCGPCTEIHF---

Lsal -------------------- -------------------- -------------------- -------------------- --------------------

Aamp -------------------- -------------------- -------------------- -------------------- --------------------

Afra TFFEMLGNWSFGDYFKKEIC TWAWEFLTEDLNLSKDRLYV SYFGGNASLGLDPDLEAKEI WLNLGVDKSRIIPGTMSDNF WEMGETGPCGPCSEIHF---

Hazt TFFEMLGNWSFGDYFKDEIC SWSWDLLTNVYKLPKDRLYV TYFGGEPSQQLEPDLECRQK WLDLGVPAEHILPGSMKDNF WEMGDTGPCGPCSEIHF---

Meda -------------------- -------------------- -------------------- -------------------- --------------------

Afos TFFEMLGNWSFGDYFKLEIC TWAWELLTQVYKIPSERLYV TYFGGEPSAGLEPDLEAKQI WMNLGVPEARILPGNMKDNF WEMGETGPCGPCSEIHY---

Dmel TFFEMLGNWSFGDYFKKEIC SWAWEFLTQRLALPKDRLYV TYFGGDAASGLEPDLECKQM WLDLGLKPEHILPGSMKDNF WEMGETGPCGPCSELHF---

Tcas TFFEMMGNWSFGDYFKKEIC AWAWEFLTKRLHLPGDRLYV TYFGGDKAAGLEPDNECKQI WLGLGVPESHVLPGSMKDNF WEMGETGPCGPCSELHF---

Pmon TFFEMLGNWSFGDYFKKEVC AWAWDLLVNVYKLPKERLYV TYFGGFPEQGLEPDLECKQI WMDLGVPEDRVLPGDMKDNF WEMGDTGPCGPCSEIHF---

Smar TFFEMLGNWSFGDYFKLEVC NWAWELLTEVYKIPKDRLYV TYFGGQPEMGLEPDNECRDI WLSP----DRILPFGMKDNF WEMGETGPCGPCSEIHF---

Tcal TFFEMLGNWSFGDYFKTEIC TWAWEFLTEVCQLPKDRLYV TYFGGEASANLEPDLECKNL WLSLGVPESKILPGNMKDNF WEMGDTGPCGPCSEIHY---

Crog TFFEMLGNWSFGDYFKKEIC EWSWEFLTKELKMPADRLYV TYFGGDDKTGLEPDLECKKI WLDLGVPEERILPGNMKDNF WEMGDTGPCGPCSEIHF---

Dpul TFFEMLGNWSFGDYFKKEVC TWAWELLTDVFQLPKDRLYV TYFGGNENAGLQPDEECRQI WLSLGLPAERILPGSMKDNF WEMGDVGPCGPCSEIHF---

Aver DRI-GGRDAAHLVNMDDPDV LEIWNLVFIQFNREQDSTLR SLPAKHIDCGMGFERLVSVI QNKRSNYDTDIFVPIFQAIE KESGVGVPYKGRVGDDDTDK

Csin -------------------- -------------------- -------------------- -------------------- --------------------

Isca -------------------- -------------------- -------------------- -------------------- --------------------

Eaff DRIGGGRDAAHLVNMDDPDV LEIWNLVFMQYNREQDKSLK QLPNKHIDCGMGFERLVSVV QDKRSNYDTDLFLPFFQHIQ AGTGVRA-YAGRVGAEDTDG

Lcyp DRI-GGRNAAHLVNQDDPDV LEIWNLVFIQYNREQDGSLL LLPNKHVDTGMGFERLTSVI QQKRSNYDTDIFVPIFDAIH KGTGTRP-YTGKVGAEDVDK

Lsal -------------------- -------------------- -------------------- -------------------- --------------------

Aamp -------------------- -------------------- -------------------- -------------------- --------------------

Afra DRI-GGRDAADLVNKDDPDV LEIWNLVFMTYNRESDGTLK YLPKKHIDCGLGLERLVSVV QEKRSNYDTDLFVPLFDAIQ KGTSMQA-YSGKVGVEDKEG

Hazt DRI-GGRDASSLVNQDDPDV LEIWNLVFIQFNRESNGQLK SLPKKHIDCGLGLERLASIM QDKRSNYDTDAFLPIFVKIQ ELTGARP-YTGMIGKADSDC

Meda -------------------- -------------------- -------------------- -------------------- --------------------

Afos DRI-G--------------X XEIWNLVFMQYNREADKSLR PLPNKHIDCGMGLERLISVI QDKRSNYDTDMFTPLFDAIQ KGTGAPA-YGGKVGAEDADG

Dmel DRI-GGRSVPELVNMDDPDV LEIWNLVFIQYNRESDGSLK QLPKKHIDCGMGFERLVSVI QNKRSNYDTDLFVPLFDAIQ AGTGAPP-YQGRVGADDVDG

Tcas DRI-GGRDVPELVNQDDPDV LEIWNLVFIQYNRESDGSLK FLPKKHIDCGLGLERLVSIM QNKRSNYDTDLFLPLFDAIQ KGTGAPP-YGGKIGDADKDG

Pmon DRI-GGRHVAHLVNMDDPDV LEIWNLVFIQFNRESDGTLK NLPKKHIDCGMGLERLTSIL QNKRSNYDTDLFLPIFETIQ KLTSVRP-YSGKVGKDDADG

Smar DRI-GNRDAKDLVNADVPDV LEIWNLVFIQFNRETDGSLR PLPKKHIDCGMGLERLVSVI QQKSSNYDTDFFVPIFNAIQ QATNGAP-YGGRVGDEDVNS

Tcal DRI-GGRDAAHLVNMDDPDV LEIWNLVFMQFNREQDKSLK SLPRKHIDCGMGFERLVSVL QNKRSNYDTDIFVPIFEAIQ KATGVPA-YQGKLGSEDPDK

Crog DRI-GGRNAADMVNQDDPMV LEIWNLVFIQFNREADKSLK PLPKKHIDCGLGLERLVSAI QNKPSNYDTDLFSPLFEAIQ ERSGANTPYGGKFGDEDPEQ

Dpul DRI-GGRDAAHLVNMDDPDV LEIWNLVFMTFNRETDSSLK PLPRKHIDCGMGFERLVSVI QDKRSNYDTDIFQPIFEAIQ KGTNAPA-YQGKVGAEDVNG

Aver IDMAYRVVADHIRTLTVALS DGGRPDNVGRGYVLRRILRR GIRYSSEKLNAKPGFFAALV DTVVQLLGEFFPEVGKDPES IKNIINEEETQFLKTLNRGH

Csin -------------------- -------------------- -------------------- -------------------- --------------------

Isca -------------------- -------------------- -------------------- -------------------- --------------------

Eaff IDMAYRVLADHARTLTIALS DGGRPDNVGRGYVLRRILRR GVRYASEKLGAKPGFFASLV HTVVQVLGETFPEVKKDPQT VIDIINEEETQFLKTLSRGQ

Lcyp IDMAYRVVADHIRTLVIAIS DGGRPDNIGRGYVLRRILRR AIRFAIKKLNAKPNFLADLV DVVVELLGDAFPEVRKDPQF VKEVINEEETQFLKTLTRGQ

Lsal -------------------- -------------------- -------------------- -------------------- --------------------

Aamp -------------------- -------------------- -------------------- -------------------- --------------------

Afra IDMAYRVVADHIRTLTIALS DGGRPDNVGRGYVLRRILRR GVRYATEKLKAKPGFFGSLV HVVVDILGDTFSEIKKDPET IISIIDEEERQFLKTLSRGK

Hazt VDMAYRVLADHARTLTVALS DGGMPDNVGRGYVLRRILRR AVRFASEKLGAKPGVFAELV DVVVDLLQDAFPELAKDSSY VKDVINEEERQFLKTLERGR

Meda -------------------- -------------------- -------------------- -------------------- --------------------

Afos IDMAYRVLADHARTLTISLS DGGRPDNVGRGYVLRRILRR GVRYATEVLKAKPGFFGSLV HTVVELLSDTFPEIKKDPQA VIDVINEEETQFLKTLSRGQ

Dmel IDMAYRVLADHARTITIALA DGGTPDNTGRGYVLRRILRR AVRYATEKLNAKPGFFATLV NTVVDLLGDAFPEVKKDPQH IIDIINEEELQFLKTLTRGR

Tcas IDMAYRVLADHARTITIALS DGGNPDNTGRGYVLRRILRR AVRYATEKLNAKPGFFSTLV NTVVEILGDTFPEVCKDPQS IIDTINEEEEQFLKTLSRGR

Pmon VDMAYRVLADHIRTLTIALS DGGMPDNAGRGYVLRRILRR GIRYATEVLGMKPGMFASLV DVVVQILKDAFPEVAKDPEF VKDVINEEELQFLKTLDRGH

Smar IDMAYRVLADHVRTITIALS DGGRPDNTGRGYVLRRILRR AVRYAHEKLNAKPGLFSSLV DVVVENLGSTFPEITKDPEL VKEIINEEENQFLKTLTRGH

Tcal LDMAYRVIADHIRTLTIALA DGGRPDNVGRGYVLRRILRR AIRFATEKLDAKPGFLSGLV DEVVQLLGDSFPELKKDPQS TKDIIDEEEIQFLKTLSRGR

Crog IDMAYRVVADHIRTLTIVIA DGGRPDNVGRGYVLRRILRR GIRFATEKLNASPGFFGSLV DVVVSLLGQTFPELTKDPQT IKDVINEEEVQFLKTLNRGR

Dpul VDMAYRVLADHARTLTIALS DGGRPDNVGRGYVLRRVLRR GVRYATEKLNAKPGFFASLV PTVCQVLGPTFPEVNRDPQL VIDIINDEETQFLKTLTRGR

Aver KLLDRTIAKLPQGTKVLPGD IAWRL--------------- -------------------- -------------------- --------------------

Csin -------------------- -------------------- -------------------- -------------------- --------------------

Isca -------------------- -------------------- -------------------- -------------------- --------------------

Eaff KLLDRTISKL-EGAKELPGD IAWRLYDTYGFPVDLTQLMC EERGLQVDMNSYESCKAAAQ LASQGKGAGAEDTISLDVHS INELKEK-SFQPTDDSFKYA

Lcyp KLLEKTISKLGDGCKVFPGD VAWRLYDTYGFPFDLTQLMC EESGLSIDKVKYEEAKLEAQ TRSQATGTQAVDEIMLDVHS IDELKSK-LYLPTNDAPKYD

Lsal -------------------- -------------------- -------------------- -------------------- --------------------

Aamp -------------------- -------------------- -------------------- -------------------- --------------------

Afra SLLERTVAKL-GDSKVLPGN IAWRLYDTYGFPFDLTQLMC EERGLTVDQAVYEESKKEAQ RLSQGKGSGIEDTISFDVHS ITELQDKMKLPPTDDSSKYS

Hazt KLLHRTVGKL-GVSKLIPGD VAWRLYDTYGFPVDLTQLMA EENDLNVDMDAFETCKKEAQ ERSRGEAKDRDDSYRLDVHA IEELKSR-GIPFTDDSQKYD

Meda -------------------- -------------------- -------------------- -------------------- --------------------

Afos KLLDRTIGKLASGCKQLPGD IAWRLYDTYGFPVDLTQLMC EERGIEVDIAGYEDRK---- -------------------- --------------------

Dmel NLLNRTIEKL-GNQTTIPGD VAWRLYDTYGFPVDLTQLMA EEKSLKIDMDGYEAAKHNSY VLSQGKGASKIEEINLDVHA ISQLQEQ-GVPPTNDTFKYK

Tcas NLLNRTITKL-GDSKTLPGD VAWRLYDTYGFPVDLTQLMA EEKGLNVDMAVYEESKKLAQ IASQGKGSGVEDTINLDVHA ITELQNL-GVPPTDDAPKYN

Pmon KLLKRTIDKL-GGSTVVPGD VAWRLYDTYGFPVDLTQLMA EEKGLAVDMEKFEVSKKEAQ EKSRGENKDKDNSFRLDVHA IEDLKDR-KVPFTDDSFKYM

Smar ILIERTMAKMPAGINVFPGD LAWRLYDTYGFPIDLTQLMT IEKGMTVDMDGYEGAKKAAQ LMSQGKGGGVEDELKLDIHA IAELQDM-KVPATDDSYKYN

Tcal KLLDRTIAKLPEGTKVLPGD IAWRLYDTYGFPVDLTELMS EERGLKVDLEAYEVAKQAAQ LASQGISGTVEDKLALDVHA ITELNDK-SVEPTDDLPKYA

Crog KLLDRTIGKM--ESKVLSGD VAWRLYDTYGFPIDLTQLMV EERGMSVDTVGYEEAKAKAV LMSQGKGGAAEDRLALDVHS IQELQDK-SMKTTNDSFKYN

Dpul DLLERTISKL-NGQTTLPGD VAWRLYDTYGFPVDLTQLMS EEKGLSIDMVAYEEAKKQAQ LLSQGKGSGVDDTIAFDVHA ITELQNK-GVPPTDDSFKYN

Aver -------------------- -------------------- -------------------- -------------------- --------------------

Csin -------------------- -------------------- -------------------- -------------------- --------------------

Isca -------------------- -------------------- -------------------- --MTCET-----NEDLEFAV TEVHTQGGYVVHIGRLISGT

Eaff YEAESSSKDSRYKFHACTAK ILGIRHSKQFVDSVSSGQEC GVLLDRTCFYAEQGGQIYDE GFLV--------NGENEMKV NNVQVRGGYVLHMGTV-EGT

Lcyp YEFG--TSGNEYELRPCKAV IKAIRASKKFVDEIKAGQEC GLLLDQTSFYAEQGGQTYDE GYITKEG--ASEDEQLEFVV NNVQVRGGYILHIG------

Lsal -------------------- -------------------- -------------------- -------------------- --------------------

Aamp -------------------- -------------------- -------------------- -------------------- --------------------

Afra YQVR-DNDAGVYEFGSCTAK VVALRANKQFVNEVTSGEEC GVLLDRTCFYAEAGGQIYDT GFITKQE-----DDGTECMV NNVQVRGGYVLHICKT-EGK

Hazt YKAG-NSPDADYSFGSCLGK IVAIRMNGHFVEEVKSGDRC GIVTDRTCFYAESGGQLSDE GFMVKND-----DDSTEFKV ENAEIKGGFVCHVGVV-EGT

Meda -------------------- -------------------- -------------------- -------------------- --------------------

Afos -------------------- -------------------- -------------------- -------------------- -------------------S

Dmel YEAVSDERDSAYNYGVCNSK IVALRFENQFVNEITSGQKA GIVLDKTNFYAESGGQIYDQ GALVKVN-----DEANEFLV DRVYNRGGYILHIGVV-EGT

Tcas YIAG-SGINEEYKFELCHST VIALRHNKQFVEQVHTGQEC GILLDKTNFYAEQGGQIYDT GYIVKIG-----DESVEFSV KNVQVRGGYVIHIGSL-EGT

Pmon YTAG-EAVDSPYNFEGTNSK VIAIRYNNQFVDEVKSGYRC GIVLDKTNFYAESGGQVYDE GFMVKIG-----DEETEFKV ESVEVKGGFVCHIGVV-EGT

Smar YVAD---ENSEYKFEPFTGA IVALRKNKTFVEEISDGGEC GVIFDCTCFYAEQGGQTYDE GFMVKECDGGQNDDEAEFKV KNVQVKGGYILHIGNI-EGK

Tcal YHAESDDPKANYVFEACQGK VVALRKDKKFVDMVEN-EEC GVLLDRTCFYAESGGQIFDE GFMVKEG-----DESVEFKV KNVQVRAGYVLHQGHI-EGQ

Crog YKEKSPEPDSDYEFNGIEAT ILALRFDKKFVDSISCGTEC GIILDQTSFYSEQGGQIYDE GFIVKAD-----DDSVEFKV TNVQVRAGYVLHVGTLGEGT

Dpul YKSVSDAVDSPYEFDVCTSK VIAIRFQKAFHEEVSSGVEC GILLDRTSFYAESGGQAYDE GFITKIG-----DEGTEFTV KNVQVRGGYVIHLGNV-EGT

Aver -------------------- -------------------- -------------------- -------------------- --------------------

Csin -------------------- -------------------- -------------------- -------------------- --------------------

Isca LRVGDKLKLAIDEQRRKLVM NNHTGTHILNFALRKVLTAE ADQRGSLVAPDRLRFDFANK KPMSPAEVKAAEQSAVELIS KNGEVYAKDSPLSIAKAIQG

Eaff LKLGDTVELSVDEERRKNVM NNHTGTHILNFALRQVLSSD ADQKGSLVAPDKLRFDFTSK AAMSPAQVKNVEEIVNNVIN KNQEIYAKEASLAVAKTIQG

Lcyp -------------------- -------------------- -------------------- -------------------- --------------------

Lsal -------------------- -------------------- -------------------- -------------------- --------------------

Aamp -------------------- -------------------- -------------------- -------------------- --------------------

Afra LQVGDAVNLSIDTERRRLIM ANHTGTHILNFALMKTLGDE VDQKGSLVAPDRLRFDFSKK SAMTTKQILETELKSQEVIK QNNEIYFKDSNLSTAKSIQG

Hazt LKIGDEVAITFDGVRRKLLM NNHTGTHILNFALRQVLSAD ADQKGSLVAPDRLRFDFSSK GAMTVEQVKRAEQIAVEMVK SNSQVYAKTSSLAVAKSIVG

Meda -------------------- -------------------- -------------------- -------------------- --------------------

Afos VXVGDKVQLSIDEDRRRNVM SNHSGTHILNFALRQVLSAE ADQRGSLVAPERLRFDFTNK SAMTVAQVKQVEQIANAMID RNEEIYAKEAPLAIAKTIQG

Dmel LKVGDELELHIDVERRWLTM KNHSATHALNHCLLQVLGKD TEQKGSLVVPEKLRFDFNSK AAMTIEQVSKTEQLTKEMVY KNVPIYAKESKLALAKKIRG

Tcas LKKGDKVALHVDSARRRLIM NNHTGTHVLNYALRKVLGTE ADQRGSLVAPDRLRFDFTNK GAMTVDQVKNTELNSKELIS RNDQVFAKESNLAVAKTIRG

Pmon IKLGDEMNMSIDWERRRHLM NNHTGTHILNFALRQVLSSE ADQRGSLVAPDRLRFDFTNK GAMTVDQVKKTEEICRQMVK ENKPVYAKTASLYVAKSIQG

Smar LKVGDKMKML---NKRRLVM NNHTGTHVLNFALRQVLTAD ADQKGSLVAPNRLRFDFTAK SAMTPKQVKDTETIANEMIA KNEAVYAKEAPLAAAKAIQG

Tcal LKVGEVLNLQIDAIRRKNVM NNHTGTHILNFALLASLDCE PEQRGSLVAPDKLRFDFTAK KALTIEQVKKVETKANEMIA RNEEVFAKESPLAVAKAILG

Crog IKVGDKVALQIDTIRRQNVM KNHTGTHVLNFALRQVLEGD SDQKGSLVAPDKLRFDFTCK KAMTTDQVKAVELCVNEIIR KNEEIFAKEASLAVAKTIQG

Dpul LRVGDEVKLSIDQSRRRLIM SNHTGTHVLNYALRQVVGNE ADQRGSLVAPDRMRFDFTNK AAMTVDQVKQTERIANEMIR KNQEVYAKESSLVVAKAVQG

Aver -------------------- -------------------- -------------------- -------------------- --------------------

Csin -------------------- -------------------- -------------------- -------------------- --------------------

Isca LRAVFNETYPDPVRVVSIGV PVDDLMADPSSPAGSLTSIE FCGGTHLKRAGHVGDFVITT EEAIAKGIRRIVALTGPEAA KALKKAEHYQNEVNRIKAEG

Eaff LRAVFDETYPDPVRVVSVGV PVVDLEKDPNNPAGTQTSVE FCGGTHLRRAGHIEHMVIAS EEAIAKGIRRIVALTGPEAK KALAKEKLLLGELEKVKTKV

Lcyp -------------------- -------------------- -------------------- -------------------- --------------------

Lsal -------------------- -------------------- -------------------- -------------------- --------------------

Aamp -------------------- -------------------- -------------------- -------------------- --------------------

Afra LRAVFGETYPDPVRVVSIGV PVEKLEADPNSPIGLNTSIE FCGGTHLHNSSHVGDFVIAS EEAIAKGIRRIVALTGPEAT KAIKKADRLLVNI---RELV

Hazt LRAVFGEVYPDPVRVVSVGI PVEDLEKDPQSPAGNKTSIE FCGGTHLLRSGHVGHFVIAV EEAIAKGIRRIVALTGPEAQ KAINRGALLQNKVNQLTQTL

Meda -------------------- -------------------- -------------------- -------------------- --------------------

Afos LRAVFDETYPDPVRVVSVGV PVQDFEKDPNNPKGSQTSVE FCGGTHLRRAGHAEHMVIQS EEAIAKGIRRIVALTGPEAK KARAKERLLEADVVKVKALA

Dmel LRSVFDEVYPDPVRVISFGV PVDELEQNPDSEAGEQTSVE FCGGTHLRRSGHIMDFVISS EEAIAKGIRRIVALTGPEAL KALKKSEAFEQEIVRLKATI

Tcas LRAVFEETYPDPVRIVSIGI PVEKLEVDPFGPAGDNTSIE FCGGTHLKYSGHIGDFVIAS EEAIAKGIRRIVALTGPEAT KALKRTEYLENCLNDIKFLV

Pmon LRAVFGEVYPDPVRVVSIGI PVETLEADPQSPAGSVTSIE FCGGTHLLRAGHAGDFVIVS EEAIAKGIRRIVAFTGPEAV KALNKASHLENEVNKLKKRI

Smar LRAMFSETYPDPVRIVSIGI PVEDLLDDPLGPGGNKTSVE FCGGTHLIRCGHIGDFVIAS EEAIAAGNRRIVALTGPEAT KARNKAALLQNNVNKLKLLI

Tcal LRAVFDETYPDPVRVVSVGV PVETMEADPSSPAGCKTSVE FCGGTHLKRSGHTGHFIVAS EEAIAKGIRRIIALTGPEAV KAIQKAQLLQSQMESVKATI

Crog LRAVFDETYPDPVRVVSVGI PVEKMEADPTGPAGSVTSVE FCGGTHLKRAGHLQEFVIAS EEAIAKGIRRILALTGLEAK KASLKAQQLQVKVDKASEVI

Dpul LRAVFEETYPDPVRVVSIGI PVEQLEADPANPAGNSTSIE FCGGTHVKRSGHIGDFVISA EEAIAKGIRRIVAITGPEAS KALKKADLLQKEVDAVSAKV

Aver -------------------- -------------------- -------------------- -------------------- --------------------

Csin -------------------- -------------------- -------------------- -------------------- --------------------

Isca ------AKVTQAAQGKELVK QLVELTEEISQSNISYWKKD ELRTELKNLKKQLDDLDRSN KAQAQQMAIENVKKLLEQKK DSEYIVHALEVGDNAKALDA

Eaff ------ESKDMGM--KEKVR LITELNDDISAATISYHKKD EMRGELKNVKKRIDDEDRNR KAAVMGDVVEAAKAILTGNP GLPYLVCELQAYANNKAIDG

Lcyp -------------------- -------------------- -------------------- -------------------- --------------------

Lsal -------------------- -------------------- -------------------- -------------------- --------------------

Aamp -------------------- -------------------- -------------------- -------------------- --------------------

Afra ------EEAAVTQSPKETWK KVVDVTDEVSQAIIPYWRKE EMRNSLKVLKKSLDDRERAA RAAISAAAVEASKKIAQENK GVPFLVRALHAFNDSKALDG

Hazt ------SNDSLSY--KESVK LITDLMEDINQAQIQYWLRD ELRNALDSAKKVLGDADRAR KMAVSKDAIRFVKELKAANP DVPYVVVELKAFAQNKVLND

Meda -------------------- -------------------- -------------------- -------------------- --------------------

Afos ------ADKNLSIC------ -------------------- -------------------- -------------------- --------------------

Dmel ------DNDKSGKDSKSHVK EIVELTEQISHATIPYVKKD EMRNLLKGLKKTLDDKERAL RAAVSVTVVERAKTLCEANP NATVLVEQLEAFNNTKALDA

Tcas ------ETDKNGEKSKELVK KIVQLTEEVSHAVIPYWKKE EIRNALKTLKKNLDDKERAA KAAVANQVVDQIKEFVKANP NLPILVKELKAFSNTKALDT

Pmon ------AENNMPF--KEVVR LLTEMLNDINHAQIQHWRKE ELRKAVEAVKKVQGDADRAR KTAISKEAIQITKNVLAANP NIPYLVT-------------

Smar ------NDNNESS--NVIGK QIVGLDNDISQATIPHWSKD ELRSELKKLKKILDDKDKSK KASIALEVIEQTKMLFGNDA NRPVIVHKFEAGANKKALDG

Tcal ------QDAKSSLSQKEMVK LITDLTEDINSATIPYLRKY EMRNELKTVKQTIDDKDRAR KAALMTVVVDFSKALLTLNP HLPYLVYELNACAQNKVLDG

Crog ------KEGKLSV--KELVK LITNLTEDISGSQISQWKKD EMRNSLKALKKTVDDKERAK MAAVVSKIVEEANALVEEHK EAPFLVKELNAFAQNKALDG

Dpul DAFVSQNDKTLSV--KELSR IIVDLSDDVSQANIAYWKKD DLRNILKGLKKRADDVERAI KAAVVNDVAEAAKKLVAEQV NTPFIVHEFNAFSNSKALDG

Aver -------------------- -------------------- -------------------- -------------------- --------------------

Csin -------------------- -------------------- -------------------- -------------------- --------------------

Isca ALKQVRTITPNAAAMFFSCD AEKGKVICLSSVPKDIVARG LTANGWIKEVSEVIGGKGGG REESAQAIGNNVSSLDKALE VAHNFAKLKLTC--------

Eaff ALKQVKLLSSATPTIFFSGD EDTGKILCMAYSSKEAITSG LKANEWCGSVQTLINGKGGG RPDNAQASGTNVSALNAAME AAHSFAMLKLGVEIS-----

Lcyp -------------------- -------------------- -------------------- -------------------- -------------AKRGLV-

Lsal -------------------- -------------------- -------------------- -------------------- --------------------

Aamp -------------------- -------------------- -------------------- -------------------- --------------------

Afra ALKAVKVISPSTAAMFLSVD PDTNKILALCSSSK----KD LPANEWIASITPVICGKGGG RAESAQATGTNGGCMDQAIA TANQFAKMKLKIDMH-----

Hazt ALKEVKNGPP---SLFISAD EDTGKILAMAAVPKDVVQKG LAADQWVKNLTELLDGKGGG RAENAQLSGTNTSALHKALR LSHEFAQQKLSCP-------

Meda -------------------- -------------------- -------------------- -------------------- --------------------

Afos -------------------- -------------------- -------------------- -------------------- --------------------

Dmel ALKQVRSQLPDAAAMFLSVD ADSKKIFCLSSVPKSAVEKG LKANEWVQHVSATLGGKGGG KPESAQASGTNYEKVDEIVQ LASKFAQSKLS--MHVEQTR

Tcas ALKQVRSLSPSTSALFVTVD PDSNKMFCLASVSKEGVDKG LKANEWVQAVASKLGGKGGG KADSAQASGSNSVAVEEILQ LAKQFAESRI---MQDQ---

Pmon -------------------- -------------------- -------------------- -------------------- -------------MSHN---

Smar ALKQVKLLSPQTAAMFFSSD AESEKIICLSSVPKDVVGKG LKANEWVDKISGIIDGKGGG KEESAQATGNKISALNEAME AAKEFAKLKLNN-MT-----

Tcal ALKQVKAITPNMPAMFFSAD PDDGKILCMAQVPKEVIAKG LKANEWCQQVQSLINGKGGG KPENAQASGSNVSGLKEAMK AAETYAQSKLGVSMVNT---

Crog ALKVIKS-STIPAAMLISGD ENTGKVICMAQVSKDISSK- LKANEWCKEVQSVLNGKGGG KAESAQATGTNLAGIQEAIQ MANAFAKLKLGE-M------

Dpul ALKQVKSLSPETAAIFFSVD AEANKIVVLAAAPKGANDRG LKANEWVADVSALLDGKGGG SAGSAQATGNNPAGLAEAMK KAKAFAQSKLGLDMQS----

Aver -------------------- -------------------- -------------------- -------------------- --------------------

Csin -------------------- -------------------Q TKHYPQPPQSDLTLS-YQAG DYENAEKHSMQLWRQDPTNT GILLLLSSIHFQCRKYDKTA

Isca -------------------- -------------------- -------------------- -------------------- --------------------

Eaff -------------------- -------------------- -------------------- -------------------- --------------------

Lcyp ----------FHTSDV---- -CKMANNGLGSIA------- ---IPMSGLAELAHSEYQAG DYDNAEKHCMQIWKQEPTNT GILLLLSSIHFQCRKYDKSA

Lsal -------------------- -------------------- -------------------- -------------------- --------------------

Aamp -------------------- -------------------- -------------------- -------------------- --------------------

Afra -------------------- -ATHPAIQQNIILKLPE--- -QLNTAISLAELAHREYQAG DYENAERHCLLLWRQDPTNT GVLLLLSSIYFQCRKYDKSA

Hazt -------------------- -------------------- -------------------- ---------MQLWRQDPTNT AILLLLSSIHFQCRKLDKSA

Meda -------------------- -------------------- -------------------- -------------------- --------------------

Afos -------------------- -------------------- -------------------- -------------------- --------------------

Dmel INMQSQGQSHQLPSAAHILL DQNPNSTGSNLVVKQNDI-- -QSLSSVGLLELAHREYQAV DYESAEKHCMQLWRQDSTNT GVLLLLSSIHFQCRRLDKSA

Tcas --IVVDGQNHLSSGTN---- -SGLPITGSPIVLKMNDV-- -PQLSSVGLLELAHREYQAG DYENAERHCMQLWRQDQTNT GVLLLLSSIHFQCRRLDKSA

Pmon -------SQPVSSADP---- -VKMVVKSNQATS--GNV-- -QHIATIGLAELAHREYQTG DYENAEKHCMQLWRQDPSNT AVLLLLSSIHFQCRRLDKSA

Smar -------------------- -------------------- -TILDSTGLAELAHREYQAG DYDNAERHCMQLWRQEPNNT GVLLLLSSIHFQCRRLDKSA

Tcal ----------MSTSTN---- -GSTGAGGLGTIA------- ---ISVSGLAELAHSEYQSG DYENAEKHCMQLWRQDSTNT GVLLLLSSIHFQCRKYDKSA

Crog -------------------- ----PQNGVNSGS--NGG-- -LGISVSSLAELAHSEYQAG DYENAEKHCMQLWRQDPSNT GVLLLLSSIHFQCRAYDKSA

Dpul -------------------- -LQNQQIVGGIVLKIPD--- --QLSSFSLAELAHREYQAG DYENAERHCMQLWRQDPTNT GVLLLLSSIHFQCRRYDKSA

Aver -------------------- -------------------- -------------------- -------------------- --------------------

Csin HFSTLAIKQNPLLAEAYSNL GNVYKEKGQLQEALDNYRHA VRLKPDFIDGYINLAAALVA AGDMEGAVQAYVSSLQYNP- -----DLYCVRSDLGNLLKA

Isca -------------------- -------------------- -------------------- -------------------- --------------------

Eaff -------------------- -------------------- -------------------- -------------------- --------------------

Lcyp HFSSMAIKQNPHLAEAYSNL GNVYKERGQLQEALDNYRHA VRLKPDFIDGYINLAAALVA AGDMEGAVQAYVSALQYNP- -----DLYCVRSDLGNLLKA

Lsal -------------------- -------------------- -------------------- -------------------- --------------------

Aamp -------------------- -------------------- -------------------- -------------------- --------------------

Afra QFSSLAIKQNPLLAEAYSNL GNVFKEKGQLSEALEHYRRG VRLKPDFIDGYINLAAALVA AGDMEGAVQAYVSALQYNP- -----DLYCVRSDLGNLLKA

Hazt HFSMLAIKQNPMLAEAYSNL GNVYKERGQLQEALENYRHA VRLKPDFIDGEVSPGGLIM- -------------------- ------LCQVRRTAG-----

Meda -------------------- -------------------- -------------------- -------------------- --------------------

Afos -------------------- -------------------- -------------------- -------------------- ---------CSSDLGNLLKA

Dmel QFSTLAIKQNPVLAEAYSNL GNVFKERGQLQEALDNYRRA VRLKPDFIDGYINLAAALVA ARDMESAVQAYITALQYNP- -----DLYCVRSDLGNLLKA

Tcas HFSTLAIKQNPLLAEAYSNL GNVYKERSQLQEALDNYRHA VRLKPDFIDGYINLAAALVA AGDMEQAVQAYITALQYNP- -----DLYCVRSDLGNLLKA

Pmon HFSMLAIKNNPMLAEAYSNL GNVYKERGQLQEALENYRHA VRLKPDFIDGYINLAAALVA ANDMEQAVQAYVSALQYNPK ---AEDLYCVRSDLGNLLKA

Smar HFSTLAIKQNPMLAEAYSNL GNVYKERGQLQEALDNYRHA VRLKPDFIDGYINLAAALVA AGDMEQAVQAYVSALQYNPV SIECIDLYCVRSDLGNLLKA

Tcal HFSNLAIKQNPLLAEAYSNL GNVYKERGQLPEALENYRYA VRLKPDFIDGYINLAAALVA AGDMEGAVQAYVSALQYNP- -----DLYCVRSDLGNLLKA

Crog HFSSLAIKQNPLLAEAYSNL GNVYKERSQLQEALENYRHA VRLKPDFIDGYINLAAALVA ASDMEGAVQAYVSALQYNP- -----DLYCVRSDLGNLLKA

Dpul QFSTYAIKQNPLLAEAYSNL GNVFKERGQLAEALDNYRHA VRLKPDFIDGYINLAAALVA AGDMEGAVQAYVSALQYNP- -----DLYCVRSDLGNLLKA

Aver -------------------- -------------------- -------------------- -------------------- --------------------

Csin LGRLDEAKACYLKAIETRPD FAVAWSNLGCVFNAQGEIWL AIHHFEKAVALDPNFLDAYI NLGNVLKEARIFDRAVAAYL RALNLSPNHAVVHGNLACVL

Isca -------------------- -------------------- -------------------- -------------------- --------------------

Eaff -------------------- -------------------- -------------------- -------------RAVAAYL RALNLSPNHAVVHGNLACVY

Lcyp LGRLEEAKACYLKAIETRPD FAVAWSNLGCVFNAQGEIWL AIHHFEKAVCLDPNFLDAYI NLGNVLKEARIFDRAVAAYL RALNLSPNHAVVHGNLACVY

Lsal -------------------- -------------------- -------------------- -------------------- --------------------

Aamp -------------------- -------------------- -------------------- -------------------- --------------------

Afra LGRLDEAKGCYLKAIETRPD FAVAWSNLGCVFNAQGELWL AIHHFEKAVTLDPNFLDAYI NLGNVLKEARIFDRAVAAYL RALNLSPNHAVVHGNLACVY

Hazt -------------LTAPRPD FAVAWSNLGCVFNAQGEIWL AIHHFEKAVALDPNFLDAYI NLGNVLKEARIFDRAVAAYL RALNLSPNHAVVHGNLACVY

Meda -------------------- -------------------- -------------------- -------------------- --------------------

Afos LGRLDEAKACYLKAIETRPD FAVAWSNLGCVFNAQGEIWL AIHHFEKAVALDPNFLDAYI NLGNVLKEARIFDRAVAAYL RALNLSPNHAVVHGNLACVY

Dmel LGRLEEAKACYLKAIETCPG FAVAWSNLGCVFNAQGEIWL AIHHFEKAVTLDPNFLDAYI NLGNVLKEARIFDRAVAAYL RALNLSPNNAVVHGNLACVY

Tcas LGRLDEAKACYLKAIETRPD FAVAWSNLGCVFNAQGEIWL AIHHFEKAVGLDPNFLDAYI NLGNVLKEARIFDRAVAAYL RALNLSPNNAVVHGNLACVY

Pmon LGRLDEAKACYLKAIETRPD FAVAWSNLGCVFNAQGEIWL AIHHFEKAVALDPNFLDAYI NLGNVLKEARIFDRAVAAYL RALNLSPNHAVVHGNLACVY

Smar LGRLDEAKACYLKAIETRPD FAVAWSNLGCVFNAQGEIWL AIHHFEKAVALDPNFLDAYI NLGNVLKEARIFDRAVAAYL RALNLSPNHAVVHGNLACVY

Tcal LGRLDEAKACYLKAIETRPD FAVAWSNLGCVFNAQGEIWL AIHHFEKAVALDPNFLDAYI NLGNVLKEARIFDRAVAAYL RALNLSPNHAVVHGNLACVY

Crog LGRLDEAKACYLKSIETRPD FAVAWSNLGCVFNAQGEIWL AIHHFEKAVALDPNFLDAYI NLGNVLKEARIFDRAVAAYL RALNLSPNHAVVHGNLACVY

Dpul LGRLDEAKACYLKAIETRGD FAVAWSNLGCVFNAQGDIWL AIHHFEKAVTLDPNFLDAYI NLGNVLKEARIFDRAVAAYL RALNLSPNHAVVHGNMACVY

Aver ----LIDLAIDTYRRAIELQ PNFPDAYCNLANALKEKGLV AESEECYNTALRLNPSHADS LNNLANIKREQGYIEEATRL YLKALEVFPEFAAAHSNXAS

Csin -------------------- -------------------- -------------------- -------------------- --------------------

Isca -------------------- -------------------- -------------------- -------------------- --------------------

Eaff YEQGLIDLAVDTYRRAIELQ PNFPDAYCNLANALKEKGLV VESEECYNTALQLSPTHADS LNNLANIKREQGYIEEATRL YLKALEVFPEFAAAHSNLAS

Lcyp YEQGLIDLAIDTYRRAIELQ PNFPDAYCNLANALKEKGMV PESEECYNTALKLNPSHADS LNNLANIKREQGFIEEATRC YLKALEVFPEFAAAHSNLAS

Lsal -------------------- -------------------- -------------------- -------------------- --------------------

Aamp -------------------- -------------------- -------------------- -------------------- --------------------

Afra YEQGLIDLAIDTYKRAIELQ PNFPDAYCNLANALKEKGMV QEAEDCYNTALRLCPSHADS LNNLANIKREQGYIEEATRL YLKALDVFPEFAAAHSNLAS

Hazt YEQGLIDLAIDTYRRAIELQ PNFPDAYCNLANALKEKGQV SEAEECYNTALQLCPTHADS LNNLANIKREQGHTEEATRL YLKALEVFPEFAAAHSNLAS

Meda ----LIDLAIDTYRRAIELQ PNFPDAYCNLANALKEKGLV AESEECYNTALRLNPSHADS LNNLANIKREQGYIEEATRL YLKALEVFPEFAAAHSNLAS

Afos YEQGLIDLAIDTYRRA---- -------------------- ---------ALQLSPTHADS LNNLANIKREQGFIEEATRL YLKALEVFPEFAAAHSNLAS

Dmel YEQGLIDLAIDTYRRAIELQ PNFPDAYCNLANALKEKGQV KEAEDCYNTALRLCSNHADS LNNLANIKREQGYIEEATRL YLKALEVFPDFAAAHSNLAS

Tcas YEQGLIDLAIDTYRRAIELQ PNFPDAYCNLANALKEKGQV AEAEECYNTALRLCPSHADS LNNLANIKREQGYIEEATRL YLKALEVFPEFAAAHSNLAS

Pmon YEQGLIDLAIDTYRRAIELQ PNFPDAYCNLANALKEKGQV TEAEECYNTALRLCPTHADS LNNLANIKREQGYTEDATRL YLKALEVFPEFAAAHSNLAS

Smar YEQGLIDLAIDTYRRAIELQ PNFPDAYCNLANALKEKGQV NEAEECYNTALQLCPTHADS LNNLANIKREQGFTEEATRL YLKALEVFPEFAAAHSNLAS

Tcal YEQGLIDLAVDTYRRAIELQ PNFPDAYCNLANALKEKGLV QDSEECYNTALRLCPTHADS LNNLANIKREQGYIEEATRL YLKALEVFPEFAAAHSNLAS

Crog YEQGLIDLAIDTYRRAIDLQ PNFPDAYCNLANALKEKGQV FEAEECYNTALRLSPTHADS LNNLANIKREQGFIEEATRL YLKALEVFPEFAAAHSNLAS

Dpul YEQGLIDLAIDTYRRAIELQ PHFPDAYCNLANALKEKGQV QDAEDCYSTALRLCPAHADS LNNLANIKREQGFIEEATRL YLKALDVFPDFAAAHSNLAS

Aver ILQQQGKLNEALMHYKEAIR IQPAFADAYSNMGNTLKEMH DIQGALQCYTRAIQINPAFA DAHSNLASVHKDSGNIPEAI Q----SYRTALKLKPNFPDA

Csin -------------------- -------------------- -------------------- -------------------- --------------------

Isca -------------------- -------------------- -------------------- -------------------- --------------------

Eaff ILQQQGKLNEALLHYKEAIR IQPAFADAYSNMGNTLKEMH DVQGALQCYSRAIQINPAFA DAHSNLASVHKDSGNIPEAI Q----SYRTALKLKPDFPDA

Lcyp ILQQQGKLNEALMHYKEAIR IQPTFADAYSNMGNTLKEMH DTHGALQCYTRAIHINPGFA DAHSNLASVHKDSGNIPEAI Q----SYRTALKLKPNFPDA

Lsal -------------------- -------------------- -------------------- -------------------- --------------------

Aamp -------------------- -------------------- -------------------- -------------------- --------------------

Afra VLQQQGKLNEALMHYKEAIR IQPTFADAYSNMGNTLKEMG DINGALQCYTRAIQINPGFA DAHSNLASIHKDSGNIPEAI Q----SYKTALKLKPDFPDA

Hazt ILQQQGKLNEALMHYKEAIR IQPTFADAYSNMGNTLKEMQ DIQGALQCYTRAIQINPAFA DAHSNLASIHKDSGNIPEAI QVNLSSYKTALKLKPDFPDA

Meda ILQQQGKLNEALMHYKEAIR IQPAFADAYSNMGNTLKEMH DIQGALQCYTRAIQINPAFA DAHSNLASVHKDSGNIPEAI Q----SYRTALKLKPNFPDA

Afos ILQQQGKLNEALLHYKEAIR IQPAFADAYSNMGNTLKEMH DVSGALQCYSRAIQINPAFA DAHSNLASVHKDSGNIPEAI Q----SYRTALKLKPDFPDA

Dmel VLQQQGKLKEALMHYKEAIR IQPTFADAYSNMGNTLKELQ DVSGALQCYTRAIQINPAFA DAHSNLASIHKDSGNIPEAI Q----SYRTALKLKPDFPDA

Tcas VLQQQGKLNEALMHYKEAIR IQPTFADAYSNMGNTLKEMQ DVSGALQCYTRAIQINPAFA DAHSNLASIHKDSGNIPEAI Q----SYRTALKLKPDFPDA

Pmon ILQQQGKLNEALMHYKEAIR IQPTFADAYSNMGNTLKEMQ DIQGALQCYTRAIQINPAFA DAHSNLASIHKDSGNIPEAI Q----SYRTALKLKPDFPDA

Smar VLQQQGKLNEALMHYKEAIR IQPTFADAYSNMGNTLKEMQ DIQGALQCYTRAIQINPAFA DAHSNLASIHKDSGNIPEAI A----SYRTALKLKPDFPDA

Tcal ILQQQGKLNEALMHYKEAIR IQPAFADAYSNMGNTLKEMH DVQGALQCYTRAIQINPAFA DAHSNLASVHKDSGNIPEAI Q----SYRTALKLKPNFPDA

Crog ILQQQGKLNEALMHYKEAIR IQPAFADAYSNMGNTLKEMH DVQGALQCYTRAIQINPAFA DAHSNLASVHKDSGNIPEAI Q----SYRTALKLKPNFPDA

Dpul VLQQQGKLNEALMHYKEAIR IQPSFADAYSNMGNTLKEMQ DINGALQCYTRAIQINPAFA DAHSNLASIHKDSGQIPEAI Q----SYRTALKLKPDFPDA

Aver YCNLAHCLQIVCDWTDYSAR MKTLVNIVGDQLEKNRLPSV HPHHSMLYPLSHDFRKAIAN RHAQLCIEKVSVLHRNMYKF ARNLSSDGRIRIGYVS----

Csin -------------------- ---IVSIVADQLDKNRLPSV HPHHSMLYPLSHHFRKAIAG RHAQLCLEKIQVLHKPPYRF SSNLAPDGRLRIGYVSSDFG

Isca -------------------- -------------------- -------------------- -------------------- --------------------

Eaff YCNLAHCLQIVCDWTDYNSR MKKIVAIVGDQLEKNRLPSV HPHHSMLYPLTHEYRKGIAA RHAQLCLEKIQVLHKPLYKF SPNLSPDGRLRIGYVSSDFG

Lcyp YCNLSHCLQIVCDWTDYNNR MKTLVSIVADQLEKNRLPSV HPHHSMLYPLSHEFRKAIAN KHAQLCIEKVSVLHKPPYKF SRVLPVDGRIRIGYVSSDFG

Lsal -------------------- -------------------- -------------------- -------------------- --------------------

Aamp -------------------- -------------------- -------------------- -------------------- --------------------

Afra FCNLSHCLQIVCDWTDYESR MTKLISIVGDQLQRNRLPSV HPHHSMLYPLSHDYRKAIGE RHANLCIEKVMILHKPPYKF SRDLPNGTRLRIGYVSSDFG

Hazt YCNLAHCLQIVCDWTNYDAR MKKLVTIVGEQLERNRLPSV HPHHSMLYPLSHEYRKAIAA RHANLCIEKITVLHKPPYKF PSRRASDGRLRIGYVSSDFG

Meda YCNLAHCLQIVCDWTDYPAR MKTLVNIVADQLEKNRLPSV HPHHSMLYPLSHDYRKAIAN RHAQLCIEKVSVLHRPPYKF SRSLTMDGRIRIGYVSS---

Afos YCNLAHCLQIVCDWTDYNSR MKKIVSIVGDQLEKNRLPSV HPHHSMLYPLSHEYRKGIAA RHAQLCLEKIQVLHKGAYKF SGNLAPDGRLRIGYVSSDFG

Dmel YCNLAHCLQIVCDWTDYDIR MKKLVSIVTEQLEKNRLPSV HPHHSMLYPLTHDCRKAIAA RHANLCLEKVHVLHKKPYNF LKKLPTKGRLRIGYLSSDFG

Tcas YCNLAHCLQIVCDWTDYDAR MKKLVSIVADQLEKNRLPSV HPHHSMLYPLSHEFRKAIAA RHANLCLEKIHVLHKHPYKF TTEL--KGRLRVGYVSSDFG

Pmon YCNLAHCLQIVCDWTDYDAR MKKLVSIVGEQLERNRLPSV HPHHSMLYPLSHEYRKAIAN RHANLCMEKINVLHKQPYKY PSELTSDGRIRIGYVSSDFG

Smar YCNLAHCLQIVCDWTDYESR MKKLVLIVGEQLEKNRLPSV HPHHSMLYPLSHEFRKAIAA RHANLCLEKILVLHKPPYKH PKDL-NGGRLRIGYVSSDFG

Tcal YCNLAHCLQIVCDWTDYAGR QKSLISIVGDQLEKNRLPSV HPHHSMLYPLSHEFRKNIAN RHAQLCLEKISVLHKPAYKF PMKL--NGRLRIGYVSSDFG

Crog YCNLAHCLQIVCDWTDYAAR QKNLVAIVAEQLDKNRLPSV HPHHSMLYPLNHSYRKAIAS RHAQLCLEKIAVLHKQPYKF SSEP--MKRLKIGYVSSDFG

Dpul YCNLAHCLQIVCDWTDYETR MKRLIHIVGEQLERNRLPSV HPHHSMLYPLSHEYRKAIAA RHANLCVEKILILHKANFKY PKELATNGRLRIGYVSSDFG

Aver -------------------- -------------------- -------------------- -------------------- --------------------

Csin NHPTSHLMQSIPGVHNRARV EIFCYSLSPDDGTTFRQKVS REAEHFIDLSAIQCNGKAAD RIYQDGIHVLVNMNGYTKGA RNESLFALRPAPIQAMWLGY

Isca -------------------- -------------------- -------------------- -------------------- --------------------

Eaff NHPTSHLMQSVPGFHDRSRV EIFCYSLSPDDGTAFRQKVF AEAEHFIDLSAVPCNGKAAD RIYQDGIHILVNMNGYTKGA RNE-IFALRPAPIQAMWLGY

Lcyp NHPTSHLMQSIPGMHDRSKV EIFCYSLAPDDNTTFRQKNS KESEHFVDLSSVLCNGEVAD RIYQDGIHILLNMNGYTKGA RNE-IFALRPAPIQVMWLGY

Lsal -------------------- -------------------- -------------------- -------------------- --------------------

Aamp -------------------- -------------------- -------------------- -------------------- --------------------

Afra NHPTSHLMQSLPGFHNRSKV EVFCYSLAPDDGTNFRAKVS REAEHFADLSQVTCNGKAAD RIYADGIHILVNMNGYTKGA RNE-IFALKPAPIQVMWLGY

Hazt NHPTSHLMQSIPGLHDRSRV EIFCYALSPDDGTTFRSKIA REAEHFVDLSQIPCNGKAAD RINQDGIHILVNMNGYTKGA RNE-IFALRPAPVQAMWLGY

Meda -------------------- -------------------- -------------------- -------------------- --------------------

Afos NHPTSHLMQSVPGNHDKSRV EIFCYSLSPDDGTTFRQRVA NDAEHFIDLSSIQCNGKAAD RIYADGIHILLNMNGYTKGA RNE-IFALRPAPIQAMWLGY

Dmel NHPTSHLMQSVPGLHDRSKV EIFCYALSPDDGTTFRHKIS RESENFVDLSQIPCNGKAAD KIFNDGIHILVNMNGYTKGA RNE-IFALRPAPIQVMWLGY

Tcas NHPTSHLMQSVPGLHDRSRV EIFCYALSQDDGTTFRSKIS REAEHFIDLSQIPCNGKAAD RIHADGIHILVNMNGYTKGA RNE-IFALKPAPVQVMWLGY

Pmon NHPTSHLMQSIPGLHDKSRV EIFCYALSPDDGTTFRSKIT REAEHFIDLSQIPCNGKAAD RINQDGIHILVNMNGYTKGA RNE-IFALRPAPVQVMWLGY

Smar NHPTSHLMQSIPGLHGRESV EVFCYSLSPDDGTTFRSKIS NESEHFVDLSQIPCNGKAAD RIHSDGVHILVNMNGYTKGA RNE-IFALKPAPIQVMWLGY

Tcal NHPTSHLMQSIPGVHNREKV EVFCYSLAPDDSTSFRQKIG REAEHFIDLSTLPCNGKAAD RIYQDGIHILVNMNGYTKGA RNE-IFALRPAPIQAMWLGY

Crog NHPTSHLMQSIPGLHNRDNV EIFCYSLAADDNTKFRQKIS AGADHFIDLSQVPCNGKAAD RIHADGIHILINMNGYTKGA RNE-IFALRPAPLQVMWLGY

Dpul NHPTSHLMQSVPGFHERSRV EIFCYSLSPDDGTTFRAKIA RESEHFVDLSQIICNGKAAD RINADGIHILVNMNGYTKGA RNE-IFAMRPAPIQVMWLGY

Aver -------------------- -------------------- -------------------- -------------------- --------------------

Csin PGTSGASFMDYIITDAQTSP VHLADQYSEKLAFLPNTFFI GDHMRMFPHLKERIIMADS- --TNPG-GN--VQDNVAVIN AVDIDPLKDVAHMT-QVIMR

Isca -------------------- -------------------- -------------------- -------------------- --------------------

Eaff PGTSGASFMDYIITDKQTSP LQIASQY------------- -------------------- -------------------- --------------------

Lcyp PGTSGAPFMDYIITDEQTSP LELAYQYSEKLAYMPNTFFI GDHKQMFPHLTERIIMAAKN EIKDGCLDG-PVFDNRAVLN AIDTTPILEKADIK-QVTVA

Lsal -------------------- -------------------- -------------------- -------------------- --------------------

Aamp -------------------- -------------------- -------------------- -------------------- --------------------

Afra PGTSGASFMDYIITDKVTSP LHLAHQYSEKLAYMPKTFFI GDHKQMFPHMKERVVIQDR- --EKLSSNLPAIADNVAVIN GTDLSPLYERTDVR-RVTET

Hazt PGTSGASFMDYLITDGVTSP VKLAAQYTEKLAYMKDTFFV GDHQQMFPHLKERVLIKSVE SIKNIDNEP--LKDTVALLN TADLSPVMESADLM-RVAEI

Meda -------------------- -------------------- -------------------- -------------------- --------------------

Afos PGTSGASYMDYIITDKQTSP VHLASQYSENLAYMPNTFFI GDHMRMFPHLQERIVMADA- --DNTVNGH--VQDNVAVIN AVNPEPIKNVASLI-KVTM-

Dmel PGTSGASFMDYIITDSVTSP LELAYQYSEKLSYMPHTYFI GDHKQMFPHLKERIIVCDK- --QQSS-----VVDNVTVIN ATDLSPLVENTDVK-EIKEV

Tcas PGTSGASFMDYLVTDAVTSP VELASQYSEKLAYMPYTYFV GDHRQMFPHLKERLIVSDK- --NNSTNSN--LADNVAIIN ATDLSPLVESTEVK-EIKEV

Pmon PGTSGAPFMDYLITDRITSP MKLSTQYSEKLAFMPDTFFV GDHKHMFPHLKERVLIESVD NPKLLNNEG--IKDTVALIN TTDLSPVIESAEVM-TIKEV

Smar PGTSGAPFMDFLITDRVTSP IEVSQQYSEKLAYMPNTFFI GDHRHMFPHMVERAVINTK- --DNLSKKL-DVPDNVAIVN ATDLSPIIEKADVKVKICEV

Tcal PGTSGAPFMDYIITDKQTSP LELASQYSEKLAYMPHTFFI GDHKQMFPHLGERIIMSGK- ---NINCEGMPVKDNVAVIN AVDTAPIFENAQIR-EIKQT

Crog PGTSGAPFMDYLLTDKQTSP LELSSQYSEKLVYMPDSFFI GDHMQMFPHLTERIIMAGV- --KKSSSDD--LSDTVAVIN TLDKTDLLEKTSVV-QACLS

Dpul PGTSGASYMDYIITDRVTSP LELASQFSEKLAYMPKTFFV GDHRQMFPHLKERLIITSS- ----SDKGK--VADNVALVN GTDLSPILERTEIK-KVTEV

Aver -------------------- -------------------- -------------------- -------------------- --------------------

Csin ANTRMK-----QR------- --PMLWLNFPPPRELTLSMD NCKTSVNGVNIQNGLA-TQI NAKAATGEEIPDQIMVTTRQ QYGLPDDAVVYCNFNQLYKI

Isca -------------------- -------------------- -------------------- -------------------- --------------------

Eaff -------------------- -------------------- -------------------- -------------------- RYSF----------------

Lcyp G-----------------EN EYTIAQLPTTQAISKMILNG QFETNVNGLTLQNGL-TNQL SSKAATGEVVPENILVTSRQ QYGLPDDAIVYCNFNQLYKI

Lsal -------------------- -------------------- -------------------- -------------------- --------------------

Aamp -------------------- -------------------- -------------------- -------------------- --------------------

Afra ISSSTT-----TTFEAPVVV SLTVAQLPTMQPIETMIGAG QIQTSVNGVVVQNGLATPQA HTRAATGEEVPQNIVVTTRQ QYGLPEACVVYCNFNQLYKI

Hazt VKPITSGVNGTKTSE-PIEV SMTVAQLSSTAPIQTMISSG QVQATVNGMVVQNGLATTQI NNKAATGEEVPQNILVTTRQ QYGLPDDAIVFCNFNQLYKI

Meda -------------------- -------------------- -------------------- -------------------- --------------------

Afos --------EGKHKDE-TVKA SVTVAELSTTQPISSMILNG QLQTNVDGLNIQNGLA-TQI NAKAATGEEIPDQIMVTTRQ QYGLPDDAVIYCNFNQLYKI

Dmel V----------NAQK-PVEI THKVAELPNTTQIVSMIATG QVQTSLNGVVVQNGLATTQT NNKAATGEEVPQNIVITTRR QYMLPDDAVVYCNFNQLYKI

Tcas V----------KAAK-MVEI SLKVAELPTTTPIENMIASG QVQTSLNGVVVQNGLATTQT NNKAATGEEVPQNIVITTRQ QYGLPDDAVVYCNFNQLYKI

Pmon VNPATT-----KE---SVEV AVTVAQLPSTAPIESMIQAG QVQATVNGLLVQNGLATTQM NNKAATGEEPPQNILVTTRK QYGLPEDAVIYCNFNQLYKI

Smar VVPSTS--GDGKPAK-PVEI ATTVIELPTTTPIQTMISTG QLQTSLNGIVVQNGLTTTQT NTKTATGEEVPQTILVTTRL QYGLPEDEVVYCNFNQLYKI

Tcal EMVGDK-----KDT--QVEV RVTIAQLPTTMAIESMIQNG QVQTSVNGIMIQNGLTTNLL SAKAATGEEVPEQIMVTTRK QYGLPEDAVVYCNFNQLYKI

Crog GIAGGE-----NNQE-VVEV KVAVAELPTTMAIDSMIQNG QVQTSVNGLTIQNGVTTNLL SAKTATGEEVPESILITSRQ QYGLPLDGIVFCNFNQLYKI

Dpul VTPTTT-----TGLDAPVQV SLTVAQLTTMDPIETMIGSG QVQTSVNGVVVQNGLATNQA NTRAATGEEVPQSIVVTTRQ QYGLPDDAIIYCNFNQLYKI

Aver -------------------- -------------------- -------------------- -------------------- --------------------

Csin DPSTLKMWVNILNRVPNSVI VWLLRFPQVGETNIHVAAQQ MGLKNGKLIFSNVAAKEEHV RRGQLADVCLDTPLCNGHTT GMDVLWAGTPMVTLPGETLA

Isca -------------------- -------------------- -------------------- -------------------- --------------------

Eaff -------------------- -------------------- -------------------- -------------------- --------------------

Lcyp DPPTLKMWVEILNNVPKGVL -WLLRFPQVGETNILVAAQQ MGLRSGAIIFSNVAAKEEHV RRGQLADVCLDTPLCNGHTT GMDVLWAGTPMVTLPGETLA

Lsal -------------------- -------------------- -------------------- -------------------- --------------------

Aamp -------------------- -------------------- -------------------- -------------------- --------------------

Afra DPATLKMWVNILKRVPNSIL -WLLRFPAVGETNLLHAVQN MGLPSNRVLFSNVAAKEEHV RRGQLADVCLDTPLCNGHTT GMDVLWAGTPMVTLPGETLA

Hazt DPSTLQMWCNILKRVPKGVM -WLLRFPGHGEANLLAQAQQ LGIGPGRIIFSNVAAKEEHV RRGQLVDVCLDTPLCNGHTT GMDVLWAGTPMVTLMGETLA

Meda -------------------- -------------------- -------------------- -------------------- --------------------

Afos DPGTLKMWVNILNRVPNSVL -WLLRFPQVGETNIHVAAQQ MGLKNGKIIFSNVAAKEEHV RRGQLADVCLDTPLCNGHTT GMDVLWAGTPMVTLTGETLA

Dmel DPQTLESWVEILKNVPKSVL -WLLRFPAVGEQNIKKTVSD FGISPDRVIFSNVAAKEEHV RRGQLADICLDTPLCNGHTT SMDVLWTGTPVVTLPGETLA

Tcas DPMTLHMWVNILKAVPNSVL -WLLRFPAVGEPNLQATAQQ LGLPPGRILFSNVAAKEEHV RRGQLADVCLDTPLCNGHTT SMDVLWTGTPVVTFPGETLA

Pmon DPSTLQMWCNILKRVPKSVV -WLLRFPAHGEANILAQAQQ LGVGAGRIIFSNVAAKEEHV RRGQLADVCLDTPLCNGHTT GMDVLWAGTPMITLSGETLA

Smar DPATLRMWVNILKRVPRSVL -WLLRFPAVGESNVLAAATQ MGLPAGKIIFSNVAPKEEHV RRGQLSDVCLDTPLCNGHTT GMDVLWAGTPMVTLPGETLA

Tcal DPATLKMWVNILNNVPNAVM -WLLRFPQVGETNILVAAQQ MGLRNGSIIFSNVAAKEEHV RRGQLADVCLDTPLCNGHTT GMDVLWAGTPMVTLPGETLA

Crog DPPTLKMWVAILNAVPHSVL -WLLRFPQVGETNLQVAAQQ MGLRAGAIIFSNVAAKEEHV RRGQLADVCLDTPLCNGHTT GMDVLWAGTPMVTLPGETLA

Dpul DPPTLQMWVNILKRVPNAVL -WLLRFPTVGETNMLATAQA LGLGSGRILFSNVAAKEEHV RRGQLADICLDTPLCNGHTT GMDVLWAGTPMVTLPGETLA

Aver -------------------- -------------------- -------------------- -------------------- --------------------

Csin SRVASSQLCTLDLEELVAKN PQDYENIAVKLGNDKEYRRA IRAKVWDGRISSPLFDVRVY AKDLEDLVYKMFEKFRKGEK INHITQ--------------

Isca -------------------- -------------------- -------------------- -------------------- --------------------

Eaff -------------------- -------------------- -------------------- -------------------- --------------------

Lcyp SRVASSELKALGCPELIAKS KEEYVKIAVRLGNDPDFLRA TRAKVWTSRTDSPLFNVKRY TADLERLFKVLWDKFQRGEK PDHVK------V--------

Lsal -------------------- -------------------- -------------------- -------------------- --------------------

Aamp -------------------- -------------------- -------------------- -------------------- --------------------

Afra SRVAASQLSTLGCPELIASS PKEYEDIAVRLGNDREYLKA MRAKVWKMRADSPLFDVKIY ASDMERLYYRLWERYSRGEK ADHTTDLEN-----------

Hazt SRVAASQLTCLGVSELIATT RHQYQDIAVKLGTDHDYLKA IRSRVWEARLSSPLFNCKTY ASNLENLFDAMWDKYLSGDE PDHLTEY-------------

Meda -------------------- -------------------- -------------------- -------------------- --------------------

Afos SRVASSQLTTLGLPELVADK REDYENIAVKLGTDREYLRV IRARVWQGRLKS-------- -------------------- --------------------

Dmel SRVAASQLATLGCPELIART REEYQNIAIRLGTKKKKKK- -------------------- -------------------- --------------------

Tcas SRVAASQLATLGCPELIARS RQEYQEIAIKLGTDKEYLKA MRHKVWAARTTSPLFDCKQY AQGLELLYCKMWERFAAGLK PDHITDTTAKK---------

Pmon SRVAASQLHCLGCPELVAQT RQEYEDIACKLGNDREYLKA TRAKVWKARIDSPLFDCKTY ATNLERLFGRMWKKYSGGEK PDHITEW-------------

Smar SRVAASQLTTLGCPELIAKS RQDYEEIAIRLGTDKELLKA TRAKVWGSRIKTPLFNMKKY AFDLEQLFYRMWEQYDSGRR FRHITDWNQEKILSGKDFEL

Tcal SRVASSQLVALGCPELIAKD RSDYEKIAIRLGNDKEFLRG TRAKVWKYRIDSPLFCVKTY AANIEKLFYKMWERHERGEK PDHVTELSIY----------

Crog SRVASSQLMALGCPELIAKD RKEFENIAIKLGTDKDFLRG IRAKVWKARMESPVFNVKTY ASNLERTFKLMWNKYAAGEK PDHITELANY----------

Dpul SRVAASQLTTIGCPELIASS PQEYEDIAVKLGTDSEYLKA IRAKVWIRRSDSPLFNCQIY AHDMERLFARMWQRHASGEK PNHITQWDDAE---------

Aver -------------------- -------------------- -------------------- -------------------- --------------------

Csin -------------------- -------------------- -------------------- -------------------- --------------------

Isca -------------------- -------------------- ------------MGTTLLDI IQSGVENLDSDVGLCAPDAE SHTLFAGLFNPVIEDYHGGF

Eaff ----------PDTEYVEAGF DKLQGDPECHSLLKKHLSKE ILDDLKNKKTESFKSSLKDV IQSGIENLDSGIGVYAPDAE SYMIFAALFDPIIEEYHGGF

Lcyp -----E---------MEEGF LKLQEAEHCQSLLKKYLTPQ LFTKLKGAKTPTFGSTLLDV IQSGMDNLDSGIGVYAPDAE AYEVFADLFNPIIEDYHGGF

Lsal -------------------- -------------------- -------------------- -------------------- --------------------

Aamp -------------------- -------------------- -------------------- -------------------- --------------------

Afra -------------------- -------------------- -------------------- -------------------- --------------------

Hazt -------------------- -------------------- -------------------- -------------------- --------------------

Meda -------------------- -------------------- -------------------- -------------------- --------------------

Afos --------NQKIAQYL---- -----MQESRSLLKKYLTPD LVNSLMSAKTKSYQSSLFDV VKSGIVNPDSSVGVFAPDVE AYTVFKDLLLPIIKDYHKLP

Dmel ------MVDAAVLAKLEEGY AKL-AASDSKSLLKKYLTKE VFDNLKNKVTPTFKSTLLDV IQSGLENHDSGVGIYAPDAE AYTVFADLFDPIIEDYHGGF

Tcas ------MVDAAVLEKLEAGF KKL-EASDSKSLLKKYLTRE LFDKLKTKKT-SFGSTLLDV IQSGLENHDSGIGIYAPDAD SYSVFADLFDPIIEDYHGGF

Pmon -------------------- -------------------- -------------------- -------------------- --------------------

Smar RSMGRF-------------F KKLQGAGDCKSLLKKYLTQP VFDDLKTKKT-AMGATLQDI IQSGVANLDSGVGVYAPDAE SYTLFAGLFNPIIEDYHGGF

Tcal -------------------- --LQEAEDCQSLLKKHLTKE ILDELKILKTRTFGSTLKDV IQSGIENIDSGVGVYAPDPE AYGVFANLFDPIIEDYHGGF

Crog -------------------- -------------------- -------------------- -------------------- --------------------

Dpul ------MVDAAVSEKLEAGF QKLQEATNCKSLLKKHLTRE IFDKIKDLKT-SFGSTLLDV IQSGVENLDSGFGVYAPDAE AYSVFNDLFEPMICDYHTGF

Aver -------------------- -------------------- -------------------- -------------------- --------------------

Csin -------------------- -------------------- -------------------- -------------------- --------------------

Isca KATDKHSLTNFGDLSTLANV DPDDQFVVSIGVRCGHSLQG CPFNPCLTEAQYREMEEKVR STLRMLEGELKVTYYPL--- -DKTQQQLIDDHFLFKEGAH

Eaff GAEAMHPESDFGAPEEFGNL DPDGKYIVSTRIRCGRSIDG LPFNPNMKEDDYKELETKAQ AAFISLPETHAGTYYPLTGM EEETQKQLIDDHFLFKEGDR

Lcyp GPDNYHPEPDFGDPSVFADL DPDAQYIVSTRIRCGRSVAG MPFNPNMTEHHYKYLERILK DVLEGMDDDLAGTYYSLEGM DKVVQHRLIDDHFLFKEGDR

Lsal -------------------- -------------------- -------------------- -------------------- --------------------

Aamp -------------------- -------------------- -------------------- -------------------- --------------------

Afra -------------------- -------------------- -------------------- -------------------- --------------------

Hazt -------------------- -------------------- -------------------- -------------------- --------------------

Meda -------------------- -------------------- -------------------- -------------------- --------------------

Afos EGFSGQPPQTWGEVSQIGKF GAE---VLSTRIRIARSLKG FPLNSKMTKQNLLDLEHKVQ QV---LEQNFHGKYHSLAKM SQDFRQDLVNRHFMFEDCDR

Dmel KKTDKHPASNFGDVSTFGNV DPTNEYVISTRVRCGRSMQG YPFNPCLTEAQYKEMESKVS STLSGLEGELKGKFYPLTGM EKAVQQQLIDDHFLFKEGDR

Tcas KKTDKHPPKDWGDVNAFGNL DPAGEFVVSTRVRCGRSLEG YPFNPCLTEEQYKEMEQKVS STLSGLEGELKGTFYPLTGM SKEVQQKLIDDHFLFKEGDR

Pmon -------------------- -------------------- -------------------- -------------------- --------------------

Smar KSTDKHPAKDFGDVSALGDL DPEGAFVISTRVRCGRSLQG YPFNPCLTEAQYKEMEEKVS STLSGLTGELKGTYYPLTGM SKEVQQKLIDDHFLFKEGDR

Tcal SKSQKHPPSHFGNPEDFGDL DPDREFIVSTRIRCGRSIEG FPFNPNMKKEDYENMEKLVS ETLEKFDGELKGTYHSLESM SPETQRELVDQHYLFKQGDR

Crog -------------------- -------------------- -------------------- -------------------- --------------------

Dpul KPGDAHPPRDFGDLETFGNL DPEGAFIVSTRVRCGRSLAG YAFNPCLTEANYKEMEEKVV ASLSSLEGELKGTYFPLTGM TKEVQTQLIQDRFLFKEGDR

Aver -------------------- -------------------- -------------------- -------------------- --------------------

Csin -------------------- -------------------- -------------------- -------------------- --------------------

Isca FLQAAIAA------------ --------------IKTIKT HLRSVVG------------- -------------------- -AFCPTNLGTTIRASVHIKV

Eaff FLQAAHASEFWPTGRGIFHD NEKKFLVWVGEEDHLRIISM QEGGDVGQVYSRLVQGVQEL GKTMT------FAHSPRFGW LTFCPTNLGTTIRASVHIKL

Lcyp FLQSANACRFWPVGRGIFHN ANKTFLVWCAEEDHLRIISM QNGGHVGEVYERLVRAMRYF ETQLQ------FARDDRLGF LTFCPTNLGTTIRASVHIKL

Lsal -------------------- -------------------- -------------------- -------------------- --------------------

Aamp -------------------- -------------------- -------------------- -------------------- --------------------

Afra -------------------- -------------------- -------------------- -------------------- --------------------

Hazt -------------------- -------------------- -------------------- -------------------- --------------------

Meda -------------------- -------------------- -------------------- -------------------- --------------------

Afos FNKGGGGCDFWPTGRGIFLS SDEKFLVWINEEDHLRIISM DKGGDLNAIYDRLVKSMKIL EKNIE------FSRDPYLGF HTFCPTNLGTTLRASVHIKF

Dmel FLQAANACRFWPSGRGIYHN DAKTFLVWCNEEDHLRIISM QQGGDLGQIYKRLVTAVNEI EKRVP------FSHDDRLGF LTFCPTNLGTTIRASVHIKV

Tcas FLQAANACRFWPTGRGIFHN DAKTFLVWCNEEDHLRIISM QMGGDLGQVYRRLVTGVNDI EKRLP------FSHSDRFGF LTFCPTNLGTTVRASVHIKV

Pmon -------------------- -------------------- -------------------- -------------------- --------------------

Smar FLQEANACRYWPAGRGIYHN DAKTFLVWCNEEDHLRIISM QMGGNLKEVFARLTNAVNEI EKRLP------FSHDDRLGF LTFCPTNLGTTIRASVHIKL

Tcal FLEAANAIRHWPSGRGIFFN DERSFLVWCGEEDHLRIISM QNGGHVGEVYGRLVRALEEM EKEFK------YSHDERLGF LTFCPTNLGTTIRASVHIKL

Crog -------------------- -------------------- -------------------- -------------------- --------------------

Dpul FLQAANACRYWPTGRGIYHN VDKTFLVWCNEEDHLRIISM QKGGDLKAVYARLVNAINEI EKRIP------FSHHDKYGF LTFCPTNLGTTIRASVHIAL

Aver -------------------- -------------------- -------------------- -------------------- --------------------

Csin -------------------- -------------------- -------------------- -------------------- --------------------

Isca PKLAADK--AKLESIAAKYN LQVRGTRGEHTESEGGVYDI SNKRRMGLTEYQAVKEMQDG ILELIRLEKAA-------MP AVRGDGIRGLAVFISDIRNC

Eaff PLLAAKG-MENLQSVADQFN LQVRGSAGEHSEADGGLYDI SNKERLGLTEFQAVEKMYKG VEELIRLEKELEE-----MP AVRGDGMRGLAVFISDIRNC

Lcyp PRLAANG-HEQLQAVANRFQ LQVRGSSGEHSSAIGGIYDV SNRERLGLTEFEAVQKMYHG VAELIRLEQEL-------MP TVRGDGMRGLAVFISDIRNC

Lsal -------------------- -------------------- -------------------- -------------------- --------------------

Aamp -------------------- -------------------- -------------------- -------------------- ------MRGLAVFISDIRNC

Afra -------------------- -------------------- -------------------- -----------------MMP AVKGDGMRGLAVFISDIRNC

Hazt -------------------- -------------------- -------------------- ------------------MP AVRGDGMRGLAVFISDIRNC

Meda -------------------- -------------------- -------------------- -------------------- --------------------

Afos NNLKLSE----VKEEAKKLD LQVRGTHGEHSDVRGGVMDI SNARRLGLTEFQAISQVYNG VNKILALDRDKIL------- --------------------

Dmel PKLASNK--AKLEEVAAKYN LQVRGTRGEHTEAEGGVYDI SNKRRMGLTEFEAVKEMYDG ITELIKLEKSL-------MA PVRGDGMRGLAVFISDIRNC

Tcas PKLAANK--AKLDEVAAKFN LQVRGTRGEHTEAEGGVYDI SNKRRMGLTEFDAVKEMYDG ISEIIKMEKEL-------MP AVRGDGMRGLAVFISDIRNC

Pmon -------------------- -------------------- -------------------- ------------------MP AVRGDGMRGLAVFISDIRNC

Smar PKLAQNR--DKLEEIASKFN LQVRGTSGEHTESVGGVYDI SNKRRMGLTEYQAVKEMNDG ILEMIKMEKAA-------MP AVRGDGMRGLAVFISDIRNC

Tcal PRLAAEGGIEMLQKHGDEHQ LQVRGSSGEHSEAVGGLYDI SNRERMGLTEYQAVKKMYEG VSELIKMEKALIAKDEG-MP AVRGDGMRGLAVFISDIRNC

Crog -------------------- -------------------- -------------------- -------------------- --------------------

Dpul PKLAADL--AKLEEAAGKFN LQVRGTAGEHTEAEGGVYDI SNKRRMGLTEYQAVKEMYDG LQELIRMEKEAA------MP AVRGDGMRGLAVFISDIRNC

Aver -------------------- -------------------- --------------EAVNLL SSNKYXXKQIGYLFISVLIS QNSDLINLINQSIKNDLGSR

Csin -------------------- --GDKTLDGYQKKKYVCKLL FIFLLGHDIDFGQIEAVNLL SSNKYTEKQIGYLFISVLIS GNSELIQLIVQSIKNDLASR

Isca KSKEAEIKRINKELANIRSK FKGDKTLDGYQKKKYVCKLL FIFLLGHDIDFGHMEAVNLL SSNKYSEKQIGYLFISVLLN ENSELMRLIIQSIKNDLCAR

Eaff KSKEAEVKRINKELANIRNK FKGDKTLDGYQKKKYVCKLL FIFLLGHDIDFGQMEAVNLL SSNKYTEKQIGYLFISVLIS GNSELIQLIVQSIKNDLAAR

Lcyp KSKEAEVKRINKELANIRSK FKGDKTLDGYQKKKYVCKLL FIFLLGHDIDFGHMEAVNLL SSNKYTEKQIGYLFISVLIS QNSDLINLVNQSIKNDLASR

Lsal -------------------- -------------------- -------------------- -------------------- --------------------

Aamp KSKEAEIKRINKELANIRGK FKGDKTLDGYQKKKYVCKLL FIFLLGHDIDFGHMEAVNLL SSNKYTEKQIGYLFISVLIN TNSDLIKLIIQNIKNDLASK

Afra KSKEAEIKRINKELANIRSK FKGDKTLDGYQKKKYVCKLL FIFLLGHDIDFGQMEAVNLL SSNKYTEKQIGYLFISVIIN TNSSLIQLIMQSIKNDLQSR

Hazt KSKENEVKRINKELANIRSK FKGDKTLDGYQKKKYVCKLL FIFLLGHDIDFGHMEAVNLL SSNKYTEKQIGYLFISVLVN TNSDLIKLIVQNIKNDLNSR

Meda -------------------- -------------------- --------------EAVNLL SSNKYTEKQIGYLFISVLIS QNSDLINLINQSIKNDLGSR

Afos -------------------- -------------------- -------------------- -------------------- -----------SIKNDLNSR

Dmel KSKEAEVKRINKELANIRSK FKGDKTLDGYQKKKYVCKLL FIFLLGHDIDFGHMEAVNLL SSNKYSEKQIGYLFISVLVN TNSDLIRLIIQSIKNDLQSR

Tcas KSKEAEIKRINKELANIRSK FKGDKTLDGYQKKKYVCKLL FIFLLGHDIDFGHMEAVNLL SSNKYSEKQIGYLFISVLVN TNSELIKLIIQSIKNDLASR

Pmon KSKEAEVKRINKELANIRSK FKGDKTLDGYQKKKYVCKLL FIFLLGHDIDFGHMEAVNLL SSNKYTEKQIGYLFISVLVN TNSDLIKLIVQSIKNDLGSR

Smar KSKEAEIKRINKELANIRSK FKGDKTLDGYQKKKYVCKLL FIFLLGHDIDFGHMEA---- ----------GYLFISVLVN ANSDLLRLIVQSIRNDLSSR

Tcal KSKEAEVKRINKELANIRSK FKGDKTLDGYQKKKYVCKLL FIFLLGHDIDFGHMEAVNLL SSNKYTEKQIGYLFISVLIS QNSDLINLINQSIKNDLSSR

Crog -------------------- -------------------- -------------------- -------------------- --------------------

Dpul KSKEAEIKRINKELANIRSK FKGDKTLDGYQKKKYVCKLL FIFLLGHDIDFGHMEAVNLL SSNKYTEKQIGYLFISVLVN TNSDLIRLIIQSIKNDLASR

Aver NPVHVNLALQCIANIGSKDM AEAFGXDIPKLLVSGDXLDV VKQXAALCLLRXFRTDPSII PGGEWTXRIIHLLNDQHMGV VTAASSLIDALVKRNPDEYK

Csin NPVHVSLDLQCIASIGSKDM AEAFGQEIPKLLVSG----V STQS---------------- -------------------- --------------------

Isca NPIHVNLALQCIANIGSREM AETFGGEIPKLLVSGDTIDV VKQSAALCLLRLLRTLPDIT PSGEWTSRIIHLLNDQHMGV VTAAVSLIDALVKKNPDEYK

Eaff NPVHVSLALQCIANIGSKDM AEAFGSEIPKLLVSGDTLDV VKQSAALCLLRLFRTDPSVI PSGEWTTRIVHLLNDHHMGV VTAAASLIDALVKKNPEEYK

Lcyp NPVHVNLALQCIANIGSKDM AEAFGNDIPKLLVSGDTLDV VKQSAALCLLRLFRTEPGII PGGEWTSRIIHLLNDQHMGV VTAASSLIDALVKKNPDEYK

Lsal -------------------- -------------------- -------------------- -------------------- --------------------

Aamp NPVHVNLAMQCIANIGSKDM AEAFGQEIPRLLVSADAIDV VKQSAALG------------ -------------------- --------------------

Afra -------------------- -------------------- -------------------- -------------------- ---------------PDEYK

Hazt NPVHVNLALQCIANIGSKEM AEAFGHDIPKLLVSGETMDV VKQSAALCLLRLFRTNPDII PGGEWTSRIIHLLNDQHLGV VTAASSLIEALVKLNPDEYK

Meda NPVHVNLALQCIANIGSKDM AEAFGSEIPKLLVSGDTLDV VKQSAALCLLRLFRTDPTII PGGEWTSRIIHLLNDQHMGV VTAASSLIDALVKKNPDEYK

Afos NPVHVSLALQCIANIGSKDM AEAFGVEIPKLLVSGDTLDV VKQSAALCLLRLFRTDPAVI PSGEWTTRIIHLLNDHHMGV VTAAASLIDALVKKNPDEYK

Dmel NPVHVNLALQCIANIGSRDM AESFSNEIPKLLVSGDTMDV VKQSAALCLLRLFRSSPDII PGGEWTSRIIHLLNDQHMGV VTAATSLIDALVKRNPDEYK

Tcas NPIHVNLALQCIANIGSREM AEAFGNEIPKLLVSGDTMDV VKQSAALCLLRLFRTSQEII PGGEWTSRIIHLLNDQHMGV VTAATSLIDALVKKNPEEYK

Pmon NPVHVNLALQCIANIGSKEM ADAFGTDIPKLLVSGETMDV VKQSAALCLLRLFRTTQEII PGGEWTSRIIHLLNDQHMGV VTAACSLIEALVKKNPDEYK

Smar NPIHVNLALQCIANIGSKEM AEAFGTEIPKLLISGDTIDV VKQGAALCLLRLFRTTPEII PSGEWTSRIIHLLNDQHMGV VTAATSLIDALVKKNPDEYK

Tcal NPVHISLALQCIANIGSKDM AEVFAPDIPKLLVSGDTLDV VKQSAALCLLRLFRTDPSII PGGEWTSRIIHLLNDQHMGV VTAASSLIDALVKKNPDEYK

Crog ---------QCIANIGSKDM AEAFSNDIPRLLVSGETLDV VKQSAALCLLRLFRTDPNII PEGEWTSRIIHLLNDQHMGV VTAATSLIDALVKKNADEYK

Dpul NPIHVNLALQCIANIGSKEM AETFGTEIPKLLVSGDTMDV VKQSAALCLLRLLRTCPEVI PSGEWTSRIIHLLNDQHLGV VTAAASLIEALVKRNTDEYK

Aver GCVSLAVSRLSRIXTSSYTX F------------------- -------------------- -------------------- --------------------

Csin -------------------- -------------------- -------------------- -------------------- --------------------

Isca GCVSLAVSRLSRIVTASYTD LQDYTYYFVPAPWLSMKLLR LLQNY-PPPDDPGVRGRLNE CLETILNKAQEPPKSKKVQH SNAKNAVLFEAISLIIHMDR

Eaff GCISLAVSRLSRIVTSSYTD FQDYTYYFVPAPWLSVKILR LLQNY-PPPEDPGVRARLTE CLETILNKAGEPLKSKKVQH SNAKNAVLFEAISLIIHNDS

Lcyp GCVSLAVSRLSRIVTSSYTD FQDYTYYFVPAPWLSVKLLR LLQNY-PPPEDPGVRGRLNE CLETILNKAQEPPKSKKVQH SNAKNSVLFEAISLIIHNDS

Lsal -------------------- -------------------- -------------------- -------------------- --------------------

Aamp -------------------- -------------RSVNRLR LLQNY-PPPDDPGVRGRLNE CLETILNKAQEPPKSKKVQH SNAKNAVLMEAISLIIHSDS

Afra ACVGLAVSRLSRIVTSSYTD LQDYTYYFVPAPWLSVKLLR LLQNY-PPPDDPGIRGRLTE CLETILNKAQEPPKSKKVQH SNAKNAVLFEAISLIIHMDS

Hazt GCVSLAVSRLSRIITASYTD LQDYTYYFVPAPWLSVKLLR LLQHY-PPPEDPGVRGRLNE CLDAILNKAQEPPKSKKVQH SNAKNAVLFEAISIIIHSDS

Meda GCVSLAVSRLSRIVTSSYTD F------------------- -------------------- -------------------- --------------------

Afos GCVSLAVSRLSRIVTSSYTD FQDYTYYFVPAPWLSVKILR LLQNY-PPPEDPGVRSRLTE CLETILNKAGEPLKSKKVQH SNAKNAVLFEAISLIIHNDS

Dmel GCVNLAVSRLSRIVTASYTD LQDYTYYFVPAPWLSVKLLR LLQNYNPVTEEAGVRARLNE TLETILNKAQEPPKSKKVQH SNAKNAVLFEAINLIIHSDS

Tcas GCVSLAVSRLSRIVTASYTD LQDYTYYFVPAPWLSVKLLR LLQNYTPPAEDPGVRGRLNE CLETILNKAQEPPKSKKVQH SNAKNAVLFEAISLIIHNDS

Pmon GCVSLAVSRLSRIVTASYTD LQDYTYYFVPAPWLSVKLLR LLQHY-PPPEDPGVRGRLNE CLDTILNKAQEPPKSKKVQH SNAKNAVLFEAISIIIHSDS

Smar GCVSLAVSRLSRIVTASYTD LQDYTYYFVPAPWLSVKLLR LLQNY-PPPEDPGVRGRLTE CLETILNKAQEPPKSKKVQH SNAKNAVLFEAISLIIHMDS

Tcal GCVPLAVSRLSRIVTASYQD FQDYTYYFVPAPWLSVKLLR LLQNY-AAPEDPGVRGRLNE CLETILNKAQEPPKSKKVQH SNAKNSVLFEAISLIIHNDS

Crog GCVSLAVSRLSRIVTSNYQD FQDYTYYFVPAPWLSVKLLR LLQNY-PSPEDPGVRSRLNE CLDNILNKAQEPPKSKKVQH SNAKNAVLFEAISLIIHNDS

Dpul GCVSLAVSRLSRIVTASYTD LQDYTYYFVPAPWLSVKLLR LLQNY-PPPEDAGVRGRLTE CLETILNKAQEPPKSKKVQH SNAKNAVLFEAISLIIHADS

Aver -------------------- -------------------- -------------------- -------------------- --------------------

Csin -------------------- -------------------- -------------------- -------------------- --------------------

Isca LGAVIEPNLLVRACNQLGQF LQHRETNLRYLALESLCLLA TSEFSHEAVKKHQETV---- ------VNALKTERDVSVRQ RAVDLLYAMCDKSNAEEIVA

Eaff -----EPNLLVRACNQLGQF LSHRETNLRYLALESMCILA TSEFSHEAVKKHQETV---- ------ISALKTERDVSVRQ RAVDLLYAMCDRTNSEEIVL

Lcyp -----EPNLLVRACNQLGQF LSHRETNLRYLALESMCLLA TSEFSHEAVKKHQETV---- ------INALKTERDVSVRQ RAVDLLYAMCDRTNSEEIVL

Lsal -------------------- -------------------- -------------------- -----------------VRQ RAVDLLYAMCDRTNAEDIVL

Aamp -----EPQLLVRACNQLGQF LTHRETNLRYLALESMCLLA TSEFSHEAVKKHQETVINAL KTEVGEINALKTERDVSVRQ RAVDLLYAMCDRTNAETIVQ

Afra -----DASLLVRACNQLGQF LSHRETNLRYLALESMCTLA ASEFSHEAVKKHQETV---- ------INALKTERDVSVRQ RAVDLLYAMCDKTNAEEIVQ

Hazt -----EAALLVRACNQLGQF LSHRETNLRYLALETMCLLA TSEFSHEAVKKHQETV---- ------INSLKTERDVSVRQ RAVDLLYAMCDRTNAEQIVH

Meda -------------------- -------------------- -------------------- -------------------- --------------------

Afos -----EPNLLVRACNQLGQF LSHRETNLRYLALESMCILA TSEFSHEAVKKHQETV---- ------ISALKTERDVSVRQ RAVDLLYAMCDRTNSEEIVL

Dmel -----EPNLLVRACNQLGQF LSNRETNLRYLALESMCHLA TSEFSHEEVKKHQEVV---- ------ILSMKMEKDVSVRQ MAVDLLYAMCDRGNAEEIVQ

Tcas -----EANLLVRACNQLGQF LSNRETNLRYLALESMCHLA TSEFSHEAVKKHQEVV---- ------ILSMKMEKDVSVRQ QAVDLLYAMCDKSNAEEIVQ

Pmon -----EPNLLVRGCNQLGQF LSHRETNLRYLALETMCLLA TSEFSHEAVKKHQETV---- ------INALKTERDVSVRQ RAVDLLYAMCDRSNAEEIVQ

Smar -----EPQLLVRACNQLGQF LQHRETNLRYLALESMCVLA TSEFSHEAVKKHQETV---- ------VTALKTERDVSVRQ RAVDLLYAMCDKTNAEEIVQ

Tcal -----EPNLLVRACNQLGQF LSHRETNLRYLALESMCLLA TSEFSHEAVKKHQETV---- ------INALKTERDVSVRQ RAVDLLYAMCDRSNSEDIVL

Crog -----EPNLLVRACNQLGQF LIHRETNLRYLALESMCLLA TSELSHEAVKKHQDTV---- ------IKALKTERDVSVRQ RAVDLLYAMCDRTNAEDIVL

Dpul -----DATLLVRGCNQLGQF LSHRETNLRYLALESLCLLA TSEFSHDSVKKHQETV---- ------INALKTERDVSVRQ RAVDLLYAMCDKSNAEEIVQ

Aver -------------------- -------------------- -------------------- -------------------- --------------------

Csin -------------------- -------------------- -------------------- -------------------- --------------------

Isca EMLAYLETADYSIREEMVLK VAILAEKYASDYTWYVDVIL NLIRIAGDYVSEEVWYRVIQ IVINREDVQGYAAKTVFE-- ------------------AL

Eaff EMLGYLETADYSIREEMVLK VAVLAEKYATDYKWYVDVIL NLIRLAGDYVSEEVWYRVIQ IVINRDDVQGYAAKTVFE-- ------------------AL

Lcyp EMLTYLETADYSIREEMVLK VAILAEKYATDYKWYVDVIL NLIRLAGDYVSEEVWYRVIQ IVVNREDVQGYAAKTVFE-- ------------------AL

Lsal EMLTYLESADYSIREEMVLK VAILAEKYATDYKWYVDVIL NLIRLAGDYVSEEVWYRVIQ IVINRDDVQGYAAKTVFEVF QIFIKLTLFPINLYSFFRAL

Aamp EMLNYLESADYSIREEMVLK VAILAEKYAMDYTWYVDVIL NLIRIAGDYVSEEVWYRVIQ IVVNREDVQGYAAKTVFE-- ------------------AL

Afra EMLNYLETADYSIREEMVLK VAILAEKYATDYTWYVDVIL NLIRIAGDYVSDEVWFRVIQ IVINREDVQGYAAKTVFE-- ------------------AL

Hazt EMLNYLESADYSIREEMVLK VAILAEKYAGDYTWYVDVIL NLIRIAGDYVSEEVWYRVIQ IVVNREDVQGYAAKTVFE-- ------------------AL

Meda -------------------- -------------------- -------------------- -------------------- --------------------

Afos EMLSYLETADYSIREEMVLK VAVLAEKYATDYKWYVDVIL NLIRLAGDYVSEEVWYRVIQ IVINRDDVQGYAAKTVFE-- ------------------AL

Dmel EMLNYLETADYSIREEMVLK VAILAEKYATDYTWYVDVIL NLIRIAGDYVSEEVWYRVIQ IVINREEVQGYAAKTVFE-- ------------------AL

Tcas EMLNYLETADYSIREEMVLK VAILAEKYATDYTWYVDVIL NLIRIAGDYVSEEVWYRVIQ IVINRDEVQGYAAKTVFE-- ------------------AL

Pmon EMLNYLETADYSIREEMVLK VAILAEKYAQDYTWYVDVIL NLIRIAGDYVSEEVWYRVIQ IVVNREDVQGYAAKTVFE-- ------------------AL

Smar EMLNYLETADYSIREEMVLK VAILAEKYATDYTWYVDVIL NLIRIAGDYVSEEVWYRVIQ IVVNREDVQGYAAKTVFE-- ------------------TL

Tcal EMLTYLETADYSIREEMVLK VAILAEKYATDYKWYVDVIL NLIRLAGDYVSEEVWYRVIQ IVINRDDVQGYAAKTVFE-- ------------------AL

Crog EMLTYLESADYSIREEMVLK VAILAEKYATDYKWYVDVIL NLIRLAGDYVSEEVWYRVIQ IVINRDDVQGYAAKTVFE-- ------------------AL

Dpul EMLNYLETADYSIREEMVLK VAILAEKYATDYTWYVDVIL NLIRIAGDYVSDEVWFRVIQ IVINRDDVQGYAAKTVFE-- ------------------AL

Aver -------------------- -------------------- -------------------- -------------------- --------------------

Csin -------------------- -------------------- -------------------- -------------------- --------------------

Isca QAPACHENMVKVAGYILGEF GNLIAGDQRSSPLIQFQLLH SKYHLCSASTRALLLTTYVK FINLFPEIKMDIQNILRSNS NIRCADSELQQRAVEYLNLS

Eaff QSPACHENMVKVGGYILGEF GNLIAGDERSSPRVQLLLLH SKFHLCSSMTRALLLSTYVK FINLFPEIKQQIQDIFVADS NLRSSDVELQQRASEYLALS

Lcyp QAPACHENMVKVGGYILGEF GNLIAGDQRSSPIVQFRLLH SKYHLCSLSTRALLLTTYIK FINLFPEIKAHIQTVFQMDS NLKSADAELQQRACEYLSLS

Lsal QAPACHENMVKVGGYILGEF GNLIAGDERSSPMVQFRLLH SKYHLCSSSTRALLLTTYIK FINLFPEIRKEIQDVF---- -----------CASEYLALS

Aamp QAPACHENMVKVGGYILGEF GNLIAGDARSAPAVQFKLLH SKYHLCSAPTRALLLSTYVK YINLFPEIKSQIQDVLHSDN NLRSADAELQQRASEYLSLS

Afra QAQACHENMVKVGAYILGEF GNLIAGDQRSAPAVQFQLLH SKYHLCAPGTRALLLTTYVK FVNLFPEIKGMIQDVFKSDS NLKSSDAELQQRAAEYLQLS

Hazt QAPACHENMVKVGGYILGEF GNLIAGDKRSAPQVQFQLLH SKYHLCSASTRALLLTTYIK FINLFPEIKTEIQNILKCDS NLRCSDAELQQRASEYLHLS

Meda -------------------- -------------------- -------------------- -------------------- --------------------

Afos QAQACHENMVKVGGYILGEF GNLIAGDSRSSPSVQLHLLH SKYHLCSPITRALLLSTYVK FINLFPEIKQQIQDIFVADS NLKSADVELQQRASEYLALS

Dmel QAPACHENMVKVGGYILGEF GNLIAGDSRSAPLVQFKLLH SKYHLCSPMTRALLLSTYIK FINLFPEIRTNIQDVFRQHS NLRSADAELQQRASEYLQLS

Tcas QAPACHENMVKVGGYILGEF GNLIAGDQRSSPSVQFQLLH SKYHLCSPMTRALLLSTYIK FINLFPEIRTQVQEVFKQHS NLRSADAELQQRASEYLQLS

Pmon QAPACHENMVKVGGYILGEF GNLIAGDSRSAPAVQFQLLH SKYHLCSPGTRALLLTTYIK FINLFPEIKTQIQDVLKSNS NLRSSDAELQQRASEYLHLS

Smar QAPACHENMVKVGGYILGEF GNLIAGDQRSSPIVQFQLLH SKYHLCSATTRALLLSTYIK FVNLFPEIKQTIQDVLSSDN NLRSADAELQQRASEYLQLS

Tcal QAPACHENMVKVGGYILGEF GNLIAGDQRSSPAVQFRLLH SKYHLCSSSTRALLLTTYVK FINLFPEIKDQIQKVLSQDS NLRNADAELQQRASEYLQLS

Crog QAPACHENMVKVGGYILGEF GNLIAGDDRSSSMVQFRLLH SKYHLCSSSTRALLLTTYIK FINLFPEIRREIQGVFSVDS NLRSADAELQQRASEYLALS

Dpul QAPACHENMVKVGSYVLGEF GNLIAGDQRSSPAVQFQLLH SKYHLCSANTRALLLTTYVK FVNLFPEIKHLIQDVFKADN NLRSADAELQQRASEYLQLS

Aver -------------------- -------------------- -------------------- -------------------- --------------------

Csin -------------------- -------------------- -------------------- -------------------- --------------------

Isca QIASPDVLATVLEEMPPFPE RESS-ILAILKRKKPGMTEG INA------SNKENLRAAPL LAD---GAA------TNRAA PATMDLLGLSLEPAALAGQP

Eaff KITSLDVLATVLEEMPPFPE KESNGILNLLKKKRPGRVPN NPG-----LLSGEE-STKEK STN---GIS------VQRDE SGSADLLGLGMENG------

Lcyp KVASLDVLATVLEEMPPFPE KEVGGLLNLLKRKRPGRVPE NAE-----LKSGAG-SEAAV VQV--KHVTNNLSLDGAANV SGSADLLGLGL--GVLD---

Lsal KITSLDVLATVLEEMPPFPE KESCGLLNLLKKKRPGRVPH NPE-----IKASGD-DTVAN NKI---------SIEPPSNG GGSADLLVLGDTSVPSAN--

Aamp VHATTDVLATVLEEMPPFPE RESS-ILAIL-KKKSGRIPD ISK----------------- -------------------- -----------TPRAANP--

Afra IVASTDVLATVLEEMPPFPE RESF-ILAILKKKKPGRMVE GEP--------KEAKAPLPV AIM---S--------QQKEP GATVDLLGLSSTPTTSNG--

Hazt LVTSTDVLVSTFSEL----- -------------------- -------------------- -------------------- --------------------

Meda -------------------- -------------------- -------------------- -------------------- --------------------

Afos KITSLDVLATVLEEMPPFPE KESSGILNLLQKKRPGRVPE NAG-----ILNK---SLDKE KTT---NG------------ -------GLATTAANN----

Dmel IVASTDVLATVLEEMPSFPE RESS-ILAVLKKKKPGRVPE NEI--------RESKSPAPL TSAAQNNALVNNSHSKLNNS NANTDLLGLSTPPSNNI---

Tcas IIASSDVLATVLEEMPAFPE RESS-ILAVLKKKKPGRVPE NEI--------KENKSPTPV TNH---NND------VNNSS AVSGDLLGLSTPPTAQP---

Pmon VITTTDVLATVLEEMPPFTE KESS-ILAVL-KKKSGRMSA QEAQREARVQAREG-NAAPA VEK---TQT------AQMNN STSADLLGLSTPPANQ----

Smar SIASTDVLATVLEEMPPFPE RESS-ILAILKKKKPGRVSE SDV--------KEHKMPVIN SAH---GDH---VESATPSS VTSADLLGLSTPPSLPP---

Tcal NVTSLDVLATVLEEMPPFPE KESSGLLNLLKKKRPGRVPE NPE-----IKSGEE-GGSSK PQA---IISGGIPPPSAGNG GASADLLGLGFDGGNGD---

Crog KVTSLDVLATVLEEMPPFPE KESCGLLNLLKKKRPGRVPH NPE-----IKSEDD-GSSAT GKQ---SDV---SAPPPSTT NGSADLLGLGDTTTTSSVTG

Dpul IVASTDVLATVLEEMPPFPE RESS-ILAILKKKKPGRVAE PGTAGNDSATPKERKSPALN SMG---NHS------THNTS NQSTDLLGLTSPSG------

Aver -------------------- -------------------- -------------------- -------------------- --------------------

Csin -------------------- -------------------- NGS-GTVTNNIQKFYCKNNG VLYENDIIQIGVKTECRSNL ARLALFYGNKTNHPFMNFKP

Isca APTNGTTACLVDVFEDSSSG --------IQGGTAVV---- ----VSNEEGLKKFVCKHNG ILFENDLLQIGVKAEFRQNL GRVGLFYGNKTVSQFQSFLP

Eaff -------------------- ------------TSNG---- VES-EEVTNNILKFYCKSNG VLYENSLIQIGVKTECRSNL ARLALFYGNKTNLPFMNFQP

Lcyp SSAANGTA------------ ------------KSNG---- INGFDTPVHNLSKFYCKNNG VLYEDTIIQIGVKTECRSNL ARLALFYGNKTGSPFHNFQP

Lsal TVAVNGND--LDLGG----- -------------------- ----DSPTDNIKK------- -------------------- --------------------

Aamp HASEHPASPAGGDYGAAA-- -------------------- AAV-HDQQAAVKKFICKNNG VLFENDILQIGIKSEYRLNL GRVGIFFGNKTPMALTGFST

Afra PTTSNGAASLVDVFGDQPAA --------PTPP-------- IST-ADTTDNYKKFLFKNNG VLFENDLVQIGVKSEFRQSL GRMGLYIGNKTNQALSNFTS

Hazt -------------------- -------------------- -------------FV----- -------------------- --------------------

Meda -------------------- -------------------- -------------------- -------------------- --------------------

Afos SAAAALDEGLINIGGDSGPT --------TNGYGAEN---- GGE-NAVTNNIQKFFCKNNG VLFENSYIQIGVKTECRSNL ARLALFYGNKTNLPFLNFVS

Dmel GSGSNSNSTLIDVLGDMYGS --------NS---------- NNN-SSAVYNTKKFLFKNNG VLFENEMLQIGVKSEFRQNL GRLGLFYGNKTQVPLTNFNP

Tcas --NSGNTGVLLDVLGDIYGG --------KQANNSNS---- ATQ-PVNMYNPKKFVCKNNG VLFESDLIQIGVKSEFRQNL GRLGLFYGNKTNVPLQNFVP

Pmon NNTQSNTGVLVDVLGDLYGG GGSGGGSNTNTHNGTAAPAP AST-SGATDNIKKFVCKNNG VLYENDIIQIGIKSEFRQNL GRIGIFYGNKTSMPLQAFCP

Smar TKPLSNTDMLVDVFGDPYGP --------ATN-NVAN---- NGT-SAVEDHLKKFVCKNNG VLFENELLQIGVKAEFRQNL GRIALFYGNKTSVTFSGFSA

Tcal AGGSSSSM------------ ------------NGNG---- ISA-DQPTDNLKKFYCKNNG ILYENEIVQIGVKTECKANL ARLALFYGNKTSTPFINFQP

Crog TAATNGSSSLNG-------- -------------------- LSD-SAPTDNIKKFYCKNNG VLYESDVIQIGVKTECRSNL ARIALFYGNKTSFPFLNFQP

Dpul SSASPNPAPLVDMLADVFSS --------ASPPSVNNG--F GSS-FQPIDNLKKFACKNNG VLFENDLLQIGVKSEFRQNL GRIALYFGNKTSFALQGFSS

Aver -------------------- -------------------- -------------------- -------------------- --------------------

Csin DVS----ASPALSMAILLQV KQVDSTVEAGAQFQQMVNVE CVSDFDEQPDLNLTFFYNGA PQSVSIKLPISINKFIEPTE MNGEAFFSRWKNLSLPAQE-

Isca NVT----SD---SAALSIQV KPVEPAIEAGAQVEQMVNVE CVNDFPEAPGLVVQFVYCGV QQKFTLRLPVFLNKFLEPTA MNSESFFSRWKNLSSPGQE-

Eaff HVS----CSPVLATQILLQV KDVDSTVEAGAQFQQMLNIE CITDFTEQPDLNLSFTYNGE RQTVNCKLPMSVNKFIEPTE MNAEAFFTRWKNLSIPSQE-

Lcyp IVT----CSPELQPCVSLQV KTVDSVVEAGAQFQQMVNVE CISDFTQQPDLNLSFVCNGE AHKVPLKVPISLNKFMEPTE MNADAFFARWKNLSTPSQE-

Lsal -------------------- -------------------- -------------------- -------------------- --------------------

Aamp SVT----LPDGLTNRLTVQA KPVDDNIDGGAQVQQTVNFE LIEEYVGMPDLSVSFMYSGV QQKLHLKLPVTPNKFIEPTE MNSEAFFARWKNLSGPAQE-

Afra SVD----AGVDNISKLVIQV KPIDSTIEAGAQVQQLINVE CLEEISEFPSLTVMFTYNGI QQKFNLKLPLSLNKFFEPTE MTGEIFFARWKNLNSPQQE-

Hazt -------------------- -------------------- -------------------- ---------LSLN------- --------------------

Meda -------------------- -------------------- -------------------- -------------------- --------------------

Afos RVS----CSPELSTQIMLQV KDVESTIEAGAQFQQMVNIE CVTDFYDQPDLAITFTYNGE LQTVNAKLPLSVNKFIEPAE MNA-----------------

Dmel VLQ----WSAEDALKLNVQM KVVEPTLEAGAQIQQLLTAE CIEDYADAPTIEISFRYNGT QQKFSIKLPLSVNKFFEPTE MNAESFFARWKNLSGEQQR-

Tcas TLS----WPDDQASKLTIQM KPVEPVLEAGAQIQQMINAE CVDDYTGAPSIVLSFLYLNV PQKITIKLPLTINKFFEPTE MNGESFFARWKNLGSTNQQR

Pmon SVS----VGLAQQGKVTVQV KPVEPTIDAGAQVQQLVNCE CVEDFTGLPDLIVSFTYANV PQRLALHLPLSINKFFEPTD MNGESFFARWKNLSAPSQE-

Smar DVILICLFSLMDSAQLNMQV KPADTTIEAGAQVQQYINAE CIEDFSELPLMNVQFTYNNL PQQLSLKLPITINKFFEPTE MNSESFFARWKNLSSANQE-

Tcal IVT----CSPELATALVLQV KTVDSTVEAGAQFQQMVNLE CVADFTSQPDLNVSFMNSGL PYKVSCKIPISINKFMEATE MNGDSFFSRWKNLSMPQQE-

Crog IVT----SSPELQNYISLQV KSVDSTVEAGAQFQQMVNVE CLSDFTSQPDISLTFTCNGS PHQVSLKLPIAINKFMEPTE MNDEAFFSRWKNLSIPSQE-

Dpul SVG----TPGDLATKLILQV KPVDVNVDAGAQIQQLVNVE CVDEFLEYPTLTILFLYNGV QQRLSLKLPLTINKFFEPTE MNSEAFFTRWKNLSGSNQE-

Aver -------------------- -------------------- -------------------- -------------------- ----------SGSQALKALK

Csin SQKIFKAAHPMENSDATKTK LIGYGFQLLENIDPNPDNYV CAGIIHTRSLQIGCLLRLEP NKQAQMYRLTTRSSVEAVSQ HITNVLCET-----------

Isca EQKIFKARFPMENES-IRSK LSGFGMQLLDGIDPNPENFV CAGIIHTRGMQIGSLLRLEP NRQAQMYRLTIRSSKDGVSK VLADLLQEHF----------

Eaff AQKIFKAKFPMEATDAAKTK LIGYGFQLLENIDPNHENYV CAGIIHTRTLQIGVLLRLEP NMQAQMYRLTVRSSKDTVSQ HIVNILGDVF----------

Lcyp AQKIFKAHNAMDSTQ-VKTK LIGYGFSLLENIDPNPENYV CAGIIHTRSLQIGCLLRLEP NSQAQMYRLTVRASKNSVSQ HICDVLTEQFSGSQCIKALK

Lsal -------------------- -------------------- -------------------- -------------------- --------------------

Aamp AQKIFKAKLPMDAS--LGGR LAGFGMQLLEGVDPNPENFV CAGILHGRGVQVGSLLRLEP NKQAQMYR------------ --------------------

Afra CQKIFKANYPIDTAA-SRAK LSGFGIQLLDGIDPNPDNFV GAGIIHTKTQQVGCLLRLEP NKQAQMYRLTIRSSKSTCAQ IMSNLLLDQF----------

Hazt -------------------- -------------------- -------------------- -------------------- --------------------

Meda -------------------- -------------------- -------------------- -------------------- ----------SGSQALKALK

Afos -------------------- -------------------- -------------------- -------------------- --------------------

Dmel SQKVFKAAQPLDLPG-ARNK LMGFGMQLLDQVDPNPDNMV CAGIIHTQSQQVGCLMRLEP NKQAQMFRLTVRASKETVTR EICDLLTDQFSGSQAIKAMR

Tcas SQKIFRATQQMDLQA-ARTK IMGFGMQLLDGIDPNPDNFV CAGIIHMRSQQVGCLLRLEP NKSAQMYRLTVRSSKESVSI EICDLLADQFSGSQAIKALR

Pmon AQKIFKCSGAMETAA-IKTK LLGFGMQLLEDIDPNPENFV CAAIIHTRTQQIGCLLRLEP NRQAQMYRLTIRASKESTSG ILAELLAEQF----------

Smar VQRIFKAVQPMDSEM-AKAK LLGFGIQLLEGVDPNPDNFV CAGIIHMRSTQIGCLLRLEP NRQAQMYRLTIRTSKESISK ELSELLGNQFSGSQAIKALR

Tcal SQKIFKGSLPMDPTQ-TKTK LIGYGFSLLENVDPNPENFV CAGIVHTRSLQIGCLLRLEP NSQAHMYRLTVRSSKDSVAQ HMCEVLADQF----------

Crog MQNVFKANYPMDNTQ-VKTK LIGYGFSLLENIDPNPENFV CAGIVHTRFVQIGCLLRLEP NNLANMYRLTVRTSKDTVSQ HICAVLTEQFA---------

Dpul AQKIFQATQPIDASH-IRTK LIGTGMKLLDGIDPNPENYV SAGIIHTRTQQIGCLLRLEP NRQAQMYRLTIRSSKDSVAK LICDLLCDQFSGSQAIKALK

Aver EEGIATILINPNIATVQTTV GMADKIYSLPINPEYVEQVL ESERPDGVLLTFGGQTALNC GVELERRGVFRKYKVRILGT PVQSIIDSEDRKIFAERVAE

Csin -------------------- -------------------- -------------------- -------------------- --------------------

Isca -------------------- -------------------- -------------------- -------------------- --------------------

Eaff -------------------- -------------------- -------------------- -------------------- --------------------

Lcyp EENIETILINPNIATVQTMD NFADHVYFLPVTPERVLEVI EKERPDSIIVTFGGQTALNV GVALHDSGKLKEHNVRVLGT Q-------------------

Lsal -------------------- -------------------- -------------------- -------------------- --------------------

Aamp -------------------- -------------------- -------------------- -------------------- --------------------

Afra -------------------- -------------------- -------------------- -------------------- --------------------

Hazt -------------------- -------------------- -------------------- -------------------- --------------------

Meda EEGITTVLINPNIATVQTTV GMADKIYSLPINPEYVEQVI ESERPDGVLLSFGGQTGLNC GVELERKGVFRKFGVRILGT PVQSIIDSEDRKIFAERVAE

Afos -------------------- -------------------- -------------------- -------------------- --------------------

Dmel ESNIQTVLINPNIATVQTSK GMADKCYFLPLTPHYVEQVI KSERPNGVLLTFGGQTALNC GVQLERAGVFSKYNVRILGT PIQSIIETEDRKLFAERVNE

Tcas EENIQTVLINPNIATVQTSK GLADKVYFLPLVPDYVEQVI RVERPGGVLLTFGGQTGLNC GVELQKAGIFDKYNIQILGT PIQAIIDTEDRKIFTERIAA

Pmon -------------------- -------------------- -------------------- -------------------- --------------------

Smar EEKIQTVLINPNIATVQTSK GLADTVYFLPITEHYVEEVI KSERPDGILLTFGGQTALNC GLKLNESKILSKYHVQVLGT PISSIQNSEDRKVFSSIMAE

Tcal -------------------- -------------------- -------------------- -------------------- --------------------

Crog -------------------- --------LPI--------- -------------------- -------------------- --------------------

Dpul EEGIHSILINPNIATVQTSK GMADKVYFLPITPHYVKQVI RQERPEGILLTFGGQTALNC GIELNKSGILEKYGVKVLGT PIQSIIDSEDRKLFAEKVAE

Aver IGEEVAPSEIVDNMEDALRS SERLGYPVLVRAAFALGGLG SGFADNADELRALAKKA--F AHSSQVIIDKSLKGWKEIEY EVVRDRFDNCIT--------

Csin -------------------- -------------------- -------------------- -------------------- --------------------

Isca -------------------- -------------------- -------------------- -------------------- --------------------

Eaff -------------------- -------------------- -------------------- -------------------- --------------------

Lcyp -------------------- -------------------- -------------------- -------------------- --------------------

Lsal -------------------- -------------------- -------------------- -------------------- --------------------

Aamp -------------------- -------------------- -------------------- -------------------- --------------------

Afra -------------------- -------------------- -------------------- -------------------- --------------------

Hazt -------------------- -------------------- -------------------- -------------------- --------------------

Meda IGEEVAPSVIVENMEDALRS SEHLGYPVLVRAAFALGGLG SGFADDPDELRALAKKA--F AHSSQVIIDKSLKGWKEIEY EVVRDRFDNCIT--------

Afos -------------------- -------------------- -------------------- -------------------- --------------------

Dmel IGEQVAPSEAVYSVAQALDA ASRLGYPVMARAAFSLGGLG SGFANNEEELQSLAQQA--L AHSSQLIVDKSLKGWKEVEY EVVRDAYNNCITVCNMENFD

Tcas IGEKVAPSMAAHSVEEALEA AEQLGYPVMARAAFSLGGLG SGFANSKDELKTLAVQA--L AHSSQLIIDKSLKGWKEVEY EVVRDAFDNCITVCNMENVD

Pmon -------------------- -------------------- -------------------- -------------------- --------------------

Smar IGEKVAPSASVSDDEHAIAV AEELGYPVLVRADFALGGLG SGFANNKEELLTILRQA--F IHSNQVLVDKSLKGWKEVEY EVVRDAYDNCITVCNMENVD

Tcal -------------------- -------------------- -------------------- -------------------- --------------------

Crog -------------------S AKELGYPVLARAAYALGGLG SGFANNEEELRKLTPSS--F AHSSQVILDKSLKGWKEIEY EVVRDAYDNCITVCNMENLD

Dpul VGERVAPSAAAYSFEEALDA AEKLGYPVLARAAYTLGGLG SGFADNPEQLRSLAQSA--F AHSNQLIIDKSLKGWKEVEY EVVRDAFNNCVTVCNMENVD

Aver -------------------- -------------------- -------------------- -------------------- --------------------

Csin -------------------- -------------------- -------------------- -------------------- --------------------

Isca -------------------- -------------------- -------------------- -------------------- --------------------

Eaff -------------------- -------------------- -------------------- -------------------- --------------------

Lcyp -------------------- -------------------- -------------------- -------------------- --------------------

Lsal -------------------- -------------------- -------------------- -------------------- --------------------

Aamp -------------------- -------------------- -------------------- -------------------- --------------------

Afra -------------------- -------------------- -------------------- -------------------- --------------------

Hazt -------------------- -------------------- -------------------- -------------------- --------------------

Meda -------------------- -------------------- -------------------- -------------------- --------------------

Afos -------------------- -------------------- -------------------- -------------------- --------------------

Dmel PLGIHTGESIVVAPSQTLSD REYQMLRSTALKVIRHFGV- VGECNIQYALCPHSEQYYII EVNARLSRSSALASKATGYP LAYVAAKLALGLPLPDIKNS

Tcas PLGIHTGESIVVAPSQTLSN REYNMLRTTAIKVIRHFGV- IGECNIQYAVNPNSEEFYII EVNARLSRSSALASKATGYP LAYIAAKLALGVPLPEINNS

Pmon -------------------- -------------------- -------------------- -------------------- --------------------

Smar PLGIHTGESIVVAPSQTLTN SEYNLLRTTAIKVVRHLKI- VGECNIQYALSPDSQLYYII EVNARLSRSSALASKATGYP LAYIAAKLALGYSLPNLKNS

Tcal -------------------- -------------------- -------------------- -------------------- --------------------

Crog PLGIHTGXSIVVAPSQTLTD REYNFLRTTAIKVIRHLRS- -------------------- -------------------- --------------------

Dpul PLGIHTGESIVVAPSQTLTN REYNMLRTTAIKVVRHLGV- VGECNIQYALNPNSQEYYII EVNARLSRSSALASKATGYP LAYIAAKLALGIPLPVLRNS

Aver -------------------- -------------------- -------------------- -------------------- --------------------

Csin -------------------- -------------------- -------------------- -------------------- --------------------

Isca -------------------- -------------------- -------------------- -------------------- --------------------

Eaff -------------------- -------------------- -------------------- -------------------- --------------------

Lcyp -------------------- -------------------- -------------------- -------------------- --------------------

Lsal -------------------- -------------------- -------------------- -------------------- --------------------

Aamp -------------------- -------------------- -------------------- -------------------- --------------------

Afra -------------------- -------------------- -------------------- -------------------- --------------------

Hazt -------------------- -------------------- -------------------- -------------------- --------------------

Meda -------------------- -------------------- -------------------- -------------------- --------------------

Afos -------------------- -------------------- -------------------- -------------------- --------------------

Dmel VTGNTTACFEPSLDYCVVKI PRWDLAKFVRVSKHIGSSMK SVGEVMAIGRNFEEAFQKAL RMVDSDVLGFDPDVVPLNKE QLAEQLSEPTDRRPFVIAAA

Tcas VTGVTTACFEPSLDYCVVKI PRWDLHKFSRVSTKIGSSMK SVGEVMAIGRKFEEAFQKAL RMVDENVMGFDPYL----KD VNDEELKEPTDKRMFVVAAA

Pmon -------------------- -------------------- -------------------- -------------------- -------------MLVLAAA

Smar VTNSTTACFEPSLDYCVVKI PRWDFGKFSHVSTQLGSSMK SVGEVMAIGRTFEETLQKAF RMADESLSGFNSTV----KG VSEEELIYPTDKRMLVLAAA

Tcal -------------------- -------------------- -------------------- -------------------- --------------------

Crog -------------------- -------------------- -------------------- -------------------- --------------------

Dpul VTESTTACFEPSMDYCVVKI PRWDLSKFMRVSAKIGSSMK SVGEVMAVGRRFEEAFQKAM RMVDENVLGFDPNL----NT VSDDELECPTDKRMFVLAAA

Aver -------------------- -------------------- -------------------- -------------------- --------------------

Csin -------------------- -------------------- -------------------- -------------------- --------------------

Isca -------------------- -------------------- -------------------- -------------------- --------------------

Eaff -------------------- ---MKNIINCYNTLEK---- -----------SSVTVSEKD MLTAKQLGFCDLQIAQLVGS TDTAIRALRIDMGIMPWVKQ

Lcyp -------------------- -------------------- -------------------- -------------------- ------------------KQ

Lsal -------------------- -------------------- -------------------- -------------------- --------------------

Aamp -------------------- -------------------- -------------------- -------------------- --------------------

Afra -------------------- -------------------- -------------------- -------------------- --------------------

Hazt -------------------- -------------------- -------------------- -------------------- --------------------

Meda -------------------- -------------------- -------------------- -------------------- --------------------

Afos -------------------- -------------------- -------------------- -------------------- --------------------

Dmel LQLGMSLRELHQLTNIDYWF LEKLERII-LLQSLLTR--- -----------NGSRTDAAL LLKAKRFGFSDKQIAKYIKS TELAVRHQRQEFGIRPHVKQ

Tcas LKKGYSVDRLYDLTKIDRWF LEKMKKIVDWTTFLETI--- -----------DQLHLSHDI LLKAKQIGFSDKQIATAVKS TELAIRKQREEFGIKPYVKQ

Pmon LKKGWDVEQLYNLTKIDKWF LYKMKNITDMYDKLESL--- -----------VDEELTEAV LQRAKQLGFSDKQIGNAVQC TDLAVRALREKHNIMPTVKQ

Smar FRAGFTVEKLYSLTKIDPWF LNKMRNIILCELELEALEPK LKDAATNVQEAMNEILTREV LLKAKQLGFSDKQIADCVNT TESVVCEARKAQDVKPFVKQ

Tcal -------------------- -------------------- -------------------- -------------------- --------------------

Crog -------------------- -------------------- -------------------- -------------------- --------------------

Dpul LKKGYTVDRLYELTKIDRWF LVKMQNIIGCYNNLESQ--- -----------SEKELTSQL LLEAKRMGFSDKQIASCIGS TELAIRSKREEYAITPFVKQ

Aver -------------------- -------------------- -------------------- -------------------- --------------------

Csin -------------------- XTTHDMEFNSFEHVMVIGSG VYRIGSSVEFDWCAVRYIQE LRRLGYKTIMVNYNPETVST DYDMCDRLYFDEITYEVVMD

Isca -------------------- -------------------- -------------------- -------------------- --------------------

Eaff IDTVAAEWPATTNYLYLTYN GSTHDIEFNVEEHIMVLGSG VYRIGSSVEFDWCAVRSIQE LRRLGKKTIMLNYNPETVST DYDMCSRLYFDEISFEVVMD

Lcyp IDTVAAEWPAATNYLYLTYN GGEHDVDFEPLSHIMVIGSG VYRIGSSVEFDWCAVSCIKE LRRLGRKTIMLNYNPETVST DYDMCDRLYFDQICFETVMD

Lsal -------------------- -------------------- -------------------- -------------------- --------------------

Aamp -------------------- -------------------- -------------------- -------------------- --------------------

Afra -------------------- -------------------- -------------------- -------------------- --------------------

Hazt -------------------- -------------------- -------------------- -------------------- --------------------

Meda -------------------- -------------------- -------------------- -------------------- --------------------

Afos -------------------- -------------------- -------------------- -------------------- --------------------

Dmel IDTVAGEWPASTNYLYHTYN GSEHDVDFP-GGHTIVVGSG VYRIGSSVEFDWCAVGCLRE LRKLQRPTIMINYNPETVST DYDMCDRLYFEEISFEVVMD

Tcas IDTVAAEWPATTNYLYLTYN AFEHDLTFE-EKHTMVIGSG VYRIGSSVEFDWCAVGCLRE LRRLGRKTIMINYNPETVST DYDMSDRLYFEEISFEVVMD

Pmon VDTVSAEWPAATNYLYLTYN GNTHDLAFP-PGATLVIGSG VYRIGSSVEFDWCAVQCLRT LRKLGHRTIMVNYNPETVST DYDMCDRLYFDEVSFEVVMD

Smar IDTVAAEWPAVTNYLYMTYN ASSHDLSFS-GDHVMVLGSG VYRIGSSVEFDWCAVGCIRE LRKLGKKTIMVNCNPETVST DYDMCDRLYFDEISFERVMD

Tcal -------------------- ----DVEFSTQPHTMVIGSG VYRIGSSVEFDWCAVGCIRE LRRLGKKTIMVNYNPETVST DYDMCDRLYFDEISFEVVMD

Crog -------------------- -------------------- -------------------- -------------------- --------------------

Dpul IDTVAAEWPAHTNYLYLTYN GNAHDLTFP-GGYTMVIGSG VYRIGSSVEFDWCAVGCLRE LRNLGRKTVMINYNPETVST DYDMCDRLYFEEITFEVVMD

Aver -------------------- -------------------- -------------------- -------------------- --------------------

Csin IYEMESPYGVIPSMGGQLPN NIAMDLYRSKVKVLGTSPES IDNAENRFXFSRMLDRIGVK QPRWKELTSMDTAKQFCNEV G-------------------

Isca -----------MAQGSSLPS QT------------------ ---------FFLVL------ --------PLQNIKSFCEEV GYPCLVRPSYVLSGAAMNVA

Eaff VYQLETPEGVILSMGGQLPN NIAMDLYRSNVKVLGTSPES IDNAENRFKFSRMLDRIGIK QPKWKELTNLESAKEFCDEV GYPCLVRPSYVLSGAAMNVA

Lcyp IYELEGPEGVILSMGGQLPN NIALSLYLQHVRVLGTLPDM IDSAENRFKFSRLLDNLKVM QPRWKELQDLQSAKAFCQHV GYPCLVRPSYVLSGAAMNVA

Lsal -------------------- -------------------- -------------------- -------------------- --------------------

Aamp -------------------- -------------------- -------------------- -------------------- --------------------

Afra -------------------- -------------------- -------------------- -------------------- --------------------

Hazt -------------------- -------------------- -------------------- -------------------- --------------------

Meda -------------------- -------------------- -------------------- -------------------- --------------------

Afos -------------------- -------------------- -------------------- -------------------- --------------------

Dmel IYEMENSEGIILSMGGQLPN NIAMDLHRQQAKVLGTSPES IDCAENRFKFSRMLDRKGIL QPRWKELTNLQSAIEFCEEV GYPCLVRPSYVLSGAAMNVA

Tcas IYNIEDPVGIILSMGGQLPN NIAIDLHRQQARILGTSPDS IDGAENRFKFSRMLDRIGIM QPRWKELTNLKSAIEFCEEV GYPCLVRPSYVLSGAAMNVA

Pmon IYNLENPKGVILSMGGQLPN NIALDLHRQKARILGTSPES IDGAENRFKFSRMLDRIGIS QPQWKELTNLKSAQAFCEEV GFPCLVRPSYVLSGAAMNVA

Smar IYELENPLGVILSMGGQLPN NIAMLLHMRNVRILGTSADS IDGAENRFKFSRMLDEKKIS QPQWKVLTNLESANEFCNTV GYPCLVRPSYVLSGAAMNVA

Tcal IYQLEHPEGVILSVGGQLPN NIAMSLYRQKVNVLGTSPES IDNAENRYKFSRLLDTIEIK QPRWKELKDFHSARDFCQQV GFPCLVRPSYVLSGAAMNVA

Crog -------------------- -------------------- -------------------- -------------------- --------------------

Dpul IYNMEAPEGVILSMGGQLPN NIAMELHRQRAKILGTLPES IDCAENRFKFSRMLDRIGIL QPRWKELTNLKLAIEFCEDV GFPCLVRPSYVLSGAAMNVA

Aver -------------------- -------------------- -------------------- -------------------- --------------------

Csin -------------------- -------------------- -------------------- -------------------- --------------------

Isca YSHRDLEGYLSEASAV--SK EHPVVISKFICEAKEIDVDA VAKDGVVVAMAVSEHVENAG VHSGDATLVTPAQDLNPETL TKIRAICQAIGQALLVSGPY

Eaff DCHQDLERYLKQASDV--SK EHPVVISKFILDAKEIDVDA VACDGEVICMAVSEHVENAG VHSGDATLVTPPGDVNSETL AQIKQITSAIAKALVVSGPF

Lcyp HNEKDLEHYLSAAEAVHLSN EHPVVISKFIMDAKEIDVDA VACDGVIYCMAVSEHVENAG VHSGDATLVTPPFDLNEETL DKIEQITRKIAKGLHVRGPF

Lsal -------------------- -------------------- -------------------- -------------------- --------------------

Aamp -------------------- -------------------- -------------------- -------------------- --------------------

Afra -------------------- -------------------- -------------------- -------------------- --------------------

Hazt -------------------- -------------------- -------------------- -------------------- --------------------

Meda -------------------- -------------------- -------------------- -------------------- --------------------

Afos -------------------- -------------------- -------------------- -------------------- --------------------

Dmel YSNQDLETYLNAASEV--SR EHPVVISKFLTEAKEIDVDA VASDGRILCMAVSEHVENAG VHSGDATLVTPPQDLNAETL EAIKRITCDLASVLDVTGPF

Tcas HSNQDLETYLNAASDV--SK EHPVVISKFILESKEIDVDA VACDGVILCMAVSEHVENAG VHSGDATLVTPPQDINDETL GKITQICKAIAASLEVTGPF

Pmon HSPQDLETYLNQAAAV--SK EHPVVISKFILEAKEIDVDA VAADGELVCMAVSEHVENAG VHSGDATLVTPPQDLNSETL AKITSICAAIARALEVNGPF

Smar YSDCDLKSYLSEATAV--SP KQPIVISKFILEAKEIDVDA VAKEGKVLCMAVCEHVENAG VHSGDATLILPPQDLNAETL EMISDICQKISESLCVTGPF

Tcal HNVQDLETYLNEATAVRLSK EHPVVISKFITDAKEIDVDA VARKGEIICMAVSEHVENAG VHSGDATLVTP--------- -KIKDITYKIAQALFVSGPF

Crog -------------------- -------------------- -------------------- -------------------- --------------------

Dpul HSPQDLETYLSKASAI--SK EYPVVISKFILEAKEIDVDA VAQDGQLLCMAVSEHVENAG VHSGDATLVTPPQDLNNETL SKIQNICRAIAMSLEVSGPF

Aver -------------------- -------------------- -------------------- -------------------- --------------------

Csin -------------------- -------------------- -------------------- -------------------- --------------------

Isca NMQLIAKDNQLKVIECNLRV SRSFPFVSKTLNCDFVALAT QVILG-LPIEPIEVTFGCG- KVGVKVPQFSYSRLAGADIR LGVEMASTGEVACFGENRYE

Eaff NMQLIAKENKLKVIECNLRV SRSFPFVSKTLGHDFIATAT QIIIG-QRVEPIDVMFGEG- KVGVKVPVFSFSRLSGADIK LGVEMSSTGEVACFGEDRYE

Lcyp NMQLIAKDNQLKVIECNVRV SRSFPFVSKTLDFDFIAIAT QVIVGGELPKPVGMLRGNGT KIGVKVPQFSFSRLAGADAT LGVEMSSTGEVACFGKHPEE

Lsal -------------------- -------------------- -------------------- -------------------- --------------------

Aamp -------------------- -------------------- -------------------- -------------------- XGVEMASTGEVACFGENRHE

Afra -------------------- -------------------- --MLG-EKVEPVNVLSGCG- RIGVKVPQFSFSRLQGADVT LGVEMASTGEVACFGDNRYE

Hazt -------------------- -------------------- -------------------- -------------------- --------------------

Meda -------------------- -------------------- -------------------- -------------------- --------------------

Afos -------------------- -------------------- -------------------- -------------------- --------------------

Dmel NMQLIAKNNELKVIECNVRV SRSFPFVSKTLDHDFVATAT RAIVG-LDVEPLDVLHGVG- KVGVKVPQFSFSRLAGADVQ LGVEMASTGEVACFGDNRYE

Tcas NMQLIAKDNDLKVIECNVRV SRSFPFVSKTLDHDFVAMAT RVMVG-ELVQPVDVLSGCG- KVGVKVPQFSFSRLAGADFM LGVEMASTGEVACFGDNRYE

Pmon NMQLIAKDNHLKVIETNLRV SRSFPFVSKTLDFDFVACAT KVIVG-EKVAATDVLRGCG- RVGVKVPQFSFSRLAGADVM LGVEMASTGEVACFGENRYE

Smar NLQLIAKDNQLSVIECNLRV SRSFPFVSKVLDCDFVAIAT RAIIG-LGDISCNI-DRMN- KVGVKVPQFSFSRLIGADVT LGVEMASTGEVACFGENRYE

Tcal NMQLIAKDNELKVIECNLRV SRSFPFVSKTLGYDFIATAT NVIVG-NTPEPIDVLHGIG- KVGVKVPQFSFSRLAGADVT LGVEMSSTGEVACFGEGRFE

Crog -------------------- -------------------- -------------------- -------------------- --------------------

Dpul NMQLIAKDNELQVIECNLRV SRSFPFVSKTLEHDFIAMAT KVIVG-QRVDPVDVLRGCD- KVGVKVPQFSFSRLAGADVM LGVEMASTGEVACFGENRYE

Aver -------------------- -------------------- -------------------- -------------------- --------------------

Csin -------------------- -------------------- -------------------- -------------------- --------------------

Isca AYLKALVSTGFCIPKKNIFL SIGSYKHKAEFLPSVRTLEK MGYKLYGSLGTSDFYMEHGI KIEAMTWPFHDAGEEGNANS SNVSSDGQSCSIADFLEQKG

Eaff AYLKAMIGTGFTMPKKTILL SVGSYKHKVEMMASVEALHE MGYKLYGSSGTSDYYREHGV PVETVEWLFESIGDETD--- -IDKMAGQMVSMADYLSNKH

Lcyp AYLKAIMSTGFIIPKDTILL SVGSYKHKREMLDSVRLLSQ MGYQLYGSMGTSDYYLEHGI QVQGVDWMFGDVGADGPD-- -IRPMADQMVSMSDFLSSGR

Lsal -------------------- -------------------- -------------------- -------------------- --------------------

Aamp AYLKAMLSTGFRMPRKGILL SIGTFKHKSEFLSSAKILHQ LNYKLFASIGTADFYTEHGI PVETVEWAYENIAEQGA--- ----------------TDKE

Afra AYLKGILSTGLVLPRKNILL SIGSYKHKDEFLPSAITLAE LGFNLYGSIGTADYYSNMGL KIESVDWAFENLGE------ --NGSSGQLKSLSDYIASKH

Hazt -------------------- -------------------- -------------------- -------------------- --------------------

Meda -------------------- -------------------- -------------------- -------------------- --------------------

Afos -------------------- -------------------- -------------------- -------------------- --------------------

Dmel AYLKAMMSTGFQIPKNAVLL SIGSFKHKMELLPSIRDLAK MGYKLYASMGTGDFYAEHGV NVESVQWTFDKTTP------ --DDINGELRHLAEFLANKQ

Tcas AYLKAMMSTGFSVPEKSILL SIGSFKHKMELLPSMRVLQK MGYKLYASLGTADFYNEHGV KVESVQWTFENIDA------ --ITSQGELKHLAEFLSKKQ

Pmon AYLKSMISTGFVIPQRSILL SIGSYKHKNELLPAVRTLAQ MGYKLYASLGTADYYSTHGI QIEPVEWAYENIGE------ --TPTKGQLSSMADYLARKE

Smar AYLKAMMSTGFRIPKKNILL SIGSYNQKRELTASVRSLEK MGYQLYGSRGTADFYSSYDI KINELS-TFANIDA------ -----KMDLSDIADSLAKME

Tcal AYLKAMMSTGFKIPQSNILL SVGSYKHKVEILPSIRLLHE LDYKLYGSLGTSDYFREHGI PVEPIEWMYENLGDDIK--- -INSMAGQMVSMADYLSNKH

Crog -------------------- -------------------- -------------------- -------------------- --------------------

Dpul AYLKALMSTGMAIPKNSILL SVGSYKHKNEMLPSVRTLEK LGYKLYASLGTADFYNDHGI TVESIEWAYDDIGE------ --ITSKGQLNNMADFLAHKQ

Aver -------------------- -------------------- -------------------- -------------------- --------------------

Csin -------------------- -------------------- -------------------- -------------------- --------------------

Isca FDLVVNLPMCSSGARRISTV L-----TQGYRTRRMAVDYA VPLVTD---------ALRLV RK-APPVKTHIDCMTSRKIV RLPGLIDVHVHLREPGASHK

Eaff FDLVINLPMRNTGARRVSSF VP----TYGYKTRRMAVDYG VPLITDVKCAKLLVEAIQKT KG-KLTCK-FVDCITAKKIV RLPGLIDTHVHMREPGATHK

Lcyp FDLVINLPMRTGGARRVSSF GLP---TNG----------- -------------------- -------------------- --------------------

Lsal -------------------- -------------------- -------------------- -------------------- --------------------

Aamp LN-----------ARRVSSF Q-----TQGYRTRRMAVDYG VCLVTDVKCAKLLVEALHRL GGKSPVINTGIDCISSRRIV RLPGMIDVHVHLRDPGQTHK

Afra FDLVINLPRRTAGSSRVSSF TSGYQRSRGYITRRMAVDLA VPLVTDIKCAKLLVEALRLV GG-NPPLKPHVDCITSRRLI RLPGLIDVHVHVREPGATHK

Hazt -------------------- -------------------- -------------------- -------------------- --------------------

Meda -------------------- -------------------- -------------------- -------------------- --------------------

Afos -------------------- -------------------- -------------------- -------------------- --------------------

Dmel FDLVINLPMSGGGARRVSSF M-----THGYRTRRLAVDYS IPLVTDVKCTKLLVESMRMN GG-KPPMKTHTDCMTSRRIV KLPGFIDVHVHLREPGATHK

Tcas FDLVINLPMRNGGARRVSSF M-----THGYRTRRLAIDYS IPLVTDVKCTKLLVEALRRI GT-APPMKTHTDCMSSRRLV KLPGLIDTHVHVREPGATHK

Pmon FDLVINLPMRGGGARRVSSF M-----TQGYRTRRMAVDFS VPLVTDVKCAKLLVEALRLI GG-APDLKTHVDCVTSRRIV KLPGLIDIHVHVREPGATHK

Smar FDLVINLSMQSSGSRRVSSI M-----TSGYKTRRMAVDYS IPLITDIKCAKLLIEALRLI GSKPPQVQTHIDSICSRRII RLPGLIDMHVHLREPGATHK

Tcal FDMVINLPMRTCGAGRVSSF IP----TYGYRTRRMAIDYS VPLITDVKCAKLLIESIGKL KKQSPKLKTHIDCISSSRIV RLPGLIDPHVHLRDPGATHK

Crog -------------------- -------------------- -------------------- -------------------- --------------------

Dpul LDLVINLPMRHGGARRVSSF S-----THGYRTRRMAVDYA VPLITDIKCAKLLVEALRLV GK-APKMKPHVDCMTSRRLV RIPGLIDVHVHLREPGGTHK

Aver -------------------- -------------------- -------------------- -------------------- --------------------

Csin -------------------- -------------------- -------------------- -------------------- --------------------

Isca ENFASGTAAALAGGFTLVCA MPNTKPPLCDGTALALCQQV AKAGARCDYALFAGASPTNA EEVLSLASSVVGLKMYLNDT FGPLCMDSVSDWLEHFEKWP

Eaff EDFSSGTAAALAGGVTMVCA MPNTIPPVTDETVFNLCQKL ASEKAYCDYALMVGATSTNY ACIPKLASQASGLKMYLNRT FTDLMMESTTDWLHHFQHWP

Lcyp -------------------- -------------------- -------------------- -------------------- --------------------

Lsal -------------------- -------------------- -------------------- -------------------- --------------------

Aamp EDFASGTAAALAGGVTMVMA MPNTKPAIIDRDSFQMAK-- -------------------- -------------------- --------------------

Afra EDYSTATAAALAGGVTLICA MPNTNPAITDKMALMTVKEA ATRGARCDYGLYLGATEENY ADAPALAPSVIGMKMYLNET FTTLKMDNMAAWMKHFENWP

Hazt -------------------- -------------------- -------------------- -------------------- --------------------

Meda -------------------- -------------------- -------------------- -------------------- --------------------

Afos -------------------- -------------------- -------------------- -------------------- ----------------ANWP

Dmel EDFASGTAAALAGGVTLVCA MPNTNPSIVDRETFTQFQEL AKAGARCDYALYVGASDDNW AQVNELASHACGLKMYLNDT FGTLKLSDMTSWQRHLSHWP

Tcas EDYASCTAAALAGGVTMICA MPNTNPAVTNQETLTLVENL ARKNARCDYAIYMGASSDNY SAIPELAPQVAGLKMYLNET FTTLRLNDLNTWTKHLANWP

Pmon EDWASCTAAALAGGVTLICA MPNTQPPVTDLDTLTLSKEL ASRGARCDYAIFMGASNDNY STAPELAPRSAALKMYLNET YTTLKLDDTTVWLKHLESWP

Smar EDFASGTAAALAGGITLVGV MPNTNPAVTDRESLDIMKEC AAKGARCDYAIIAGATTTNS MIISELSSEVIGLKMYLNDT FSTLKMNNVSALVQHFEHWP

Tcal EDFATGTAAALAGGVTMMLV MPNTSPSIVDAETLTMAKDI ARQKAHCDYALNIGASASNA KLIAPLAKQVGALKMYLNET FTTLRLDNMTDWMTHFEHWP

Crog -------------------- -------------------- -------------------- -------------------- --------------------

Dpul EDFSSGTAAALSGGVTMVLA MPNTQPTITDKAALHLIKDL AKKGARCDYGIFMGASATNF EEIIELSSHVVGLKMYLNET FTTLKLDGIQQWLKHFENWP

Aver -------------------- -------------------- -------------------- -------------------- --------------------

Csin -------------------- -------------------- -------------------- -------------------- --------------------

Isca KNVPLCCHAEGRTTAAVILL ASLYRRPVHICHVAREEEIL IIKRAKEQGHEVTCEVTPHH LFLTTEDVSAAAPGKGAVKP PLVTARDQKALWDNIDVIDC

Eaff RNAPICVHAEGRTVAAVILM ASLFDRPLHICHVSSKDEIE IVKAAKIKGLPITCEVCPHH LFLTEQEIHELGDNMSDVRP RVGRQEDQEALWENMDYIDS

Lcyp -------------------- -------------------- -------------------- -------------------- --------------------

Lsal -------------------- -------------------- -------------------- -------------------- --------------------

Aamp -----------XXMAAAMMV AALANRPIHICHVAKKEEIL LIKAAKQRGFPVTCEVSPHH LFLTSSELGRLG-AMGQVKP PLMVEDDQRALWDNMAVIDC

Afra KDLPICAHAEKSNTAAVILF ASLYQRSVHICHVARKEEIE VIKMAKLKGIQVTCEVAPHH LFLSENDLGSLD-SKGEVRP RLATLEDQQALWNNLEFIDC

Hazt -------------------- -------------------- -------------------- -------------------- --------------------

Meda -------------------- -------------------- -------------------- -------------------- --------------------

Afos KSAPLCVHAEGRTTAAIILL ANLHDRPVHICHVARREEIE IIKAAKLKGIPVTCEVAPHH LFLTEDDKISLGAKKSEVRP VLVTKDDQQALWDNMDYIDS

Dmel KRSPIVCHAERQSTAAVIML AHLLDRSVHICHVARKEEIQ LIRSAKEKGVKVTCEVCPHH LFLSTKDVERLGHGMSEVRP LLCSPEDQEALWENIDYIDV

Tcas KKAPICVHAEGQTTAAVILM ASLQNRPIHICHVARKEEIL IIKAAKEKGLNITCEVCPHH LFLSTEDVKTLGVCKSQVRP VLMSPEDQQALWDNLHVIDC

Pmon AHIPVCVHAEARTMAAALMT ASLASRPIHICHVARKEEIL LIRAAKAKGMQVTCEVCPHH LFLTCDNLEQLG-QKGEVRP CLVTSEDQQALWDNLDIIDC

Smar KTLPILCHAEGMTLASVVGL LSLYVRPVHICHVAKKEEID IIKQAKKMGLPISCEVCPHH LFLTEGDIADLGVCKSQVRP MIGTEEDRKALWENLNIIDC

Tcal LTSPICVHAEGRSTAAAILL ASLYDRPLHICHVARKDEIE IIKAAKLKGLAITCEVCPHH LFLTEEHAKAMG-NAGQVRP NLVTKEDQNALWENLEFIDC

Crog -------------------- -------------------- -------------------- -------------------- --------------------

Dpul KGVPICVHAEGRTTAAVILL ASLNNRSVHVCHVATRDEIE IIKAAKKKGLPVTCEVCPHH LFLNSNDLPAFG-TKGEVRP RLASPEDQQALWDNLNIIDC

Aver -------------------- -------------------- -------------------- -------------------- --------------------

Csin -------------------- -------------------- -------------------- -------------------- --------------------

Isca IATDHAPHTLEEKMDENAPP GFPGLETALPLMLTAVAEGK LTMEALVKKMYHNPKQIFNL PVQPDTYIEVDLDEEWTIPE APPYSKAQWTPFAGRKVRGQ

Eaff IVTDHAPHTLEEKKSSVDSP GFSGLETMLPLMLTAVNEGK ITLQELEEKMYHSPRRIFGL PEQPDTYIEIDMDHEWTIPD KLTHSKAGWTPFAGRKVKGI

Lcyp -------------------- -------------------- -------------------- -------------------- --------------------

Lsal -------------------- -------------------- -------------------- -------------------- --------------------

Aamp FATDHAPHTVAEKRSEKPPP GFPG---------------- -------------------- -------------------- --------------------

Afra FATDHAPHTLQEKTSDNPPP GFPGLETMLPLLLDAVSKGR LTVEDIVTRLHDNPRRIFNL PEQPSTWVEVDLDEEWIIPS SPAQSKCMWTPFAGRTVKGS

Hazt -------------------- -------------------- -------------------- -------------------- --------------------

Meda -------------------- -------------------- -------------------- -------------------- --------------------

Afos FATDHAPHLIAEKLSDKAPP GFPGLETMLPLMLTAVNDGR LTLEDLEDKMYHNPRRIFNL PEQPDTYIEVDMDKSWIIPE KTQFSKS-------------

Dmel FATDHAPHTLAEKRSERPPP GFPGVETILPLLLQAVHEGR LTMEDIKRKFHRNPKIIFNL PDQAQTYVEVDLDEEWTITG NEMKSKSGWTPFEGTKVKGR

Tcas FATDHAPHTLEEKTSSSPPP GFPGLETILPLLLNAVNENK LTIDDIVTKFYRNPKKIFNL PDQPNTYVEIDLDEEWTIPE ATTYSKAKWTPFAGRKVKGA

Pmon FATDHAPHTLEEKTGGKAPP GFPGLETMLPLLLTAVTQGR LSMEDLINKLHHNPRRIFQL PRQPRTHIEIDLDAQWTIPE ALPFSKSQWTPFAGMKVTGR

Smar FATDHAPHVLAEKLGDNPPP GYPGLETMLPLLLTAVVEGR LTLEEIEAKLHHNPKKIFKL PDQNNTYIEVDLDEEWVVGD KTSFTKAGWTPFTGHKLQGK

Tcal FATDHAPHTLEEKNSEQSPP GFPGLETMLPLMLTAVNLGR MTLNDLKQKMHDNAKRIFNL PTQSDTYIEVNMDEAWTIPE ETTFSKAKWTPFAGMPVIGR

Crog -------------------- -------------------- -------------------- -------------------- --------------------

Dpul FATDHAPHTLEEKMSDNSPP GYPGLETMLPLLLTAVNKGQ LSLQDVVNKCHHNPKRIFKL PDQSNTYVEIDLEEDWVLPD ANVFSKAKWSPFAGRKVKGA

Aver -------------------- -------------------- -------------------- -------------------- --------------------

Csin -------------------- -------------------- -------------------- -------------------- --------------------

Isca VRRVVVRGEVAFVDGQVLVE PGYGQD-------------V MTVPAQTELERTSSLVNLSA V------------------- --------------------

Eaff VRRVILRGEVAFVDGKVLVE PGYGQD-------------V KTWSSTNQVQFKTLPAVKKS SLMDAEIAPNRIRA------ --------------------

Lcyp -------------------- -------------------- -------------------- -------------------- --------------------

Lsal -------------------- -------------------- -------------------- -------------------- --------------------

Aamp -------------------- -------------------- -------------------- -------------------- --------------------

Afra VKRVILRGEVAYVDGNVVAA PGSG---------------- -------------------- -------------------- --------------------

Hazt -------------------- -------------------- -------------------- -------------------- --------------------

Meda -------------------- -------------------- -------------------- -------------------- --------------------

Afos -------------------- -------------------- -------------------- -------------------- --------------------

Dmel VHRVVLRGEVAFVDGQVLVQ PGFGQN-------------V RPK--QSPLASEASQDLLPS -------------------- --------------------

Tcas VHRVVLRGEVAFVDGQILVQ PGFGQN-------------V RTWDKKTFDEVIAPPTVTTG EY------------------ --------------------

Pmon VQRVVLRGEVAYVDGQVLLS PGFGED-------------V REA--QPLTTASSSVTGGPP V------FPSPLCAHPSTIT LPPHSQHTPAEPVLATPTTV

Smar VRRVVLNGEVAYVDGQVLAK PGYGKD-------------V CKQG-EFKKSNLRRPIMHTG G------------------- --------------------

Tcal LKRVVLRGKLAFVDGQVLID PGFGED-------------V RTW--KQKLTTLTALAKDPV -------------------- --------------------

Crog -------------------- -------------------- -------------------- -------------------- --------------------

Dpul VKRVVLRGEVVYIDGSIIAQ PGFGQDQLTMFLAVKHLAMV KTI--ELGIPKIRSLSVGPT -------------------- --------------------

Aver -------------------- -------------------- -------------------- -------------------- --------------------

Csin -------------------- -------------------- -------------------- -------------------- --------------------

Isca -------------------K MYNKMFQELGLPEHLQ---- MPSVSED------------- ----AKLLPRCPSP--LVP- ---PVGKWSLPEVPHALQGC

Eaff ------------EGERHESD ITCERQRKESGHQGIR---S TSPIFDGLEHPHYEPYKRYH GSPTKTDRPDKAEPEMLPSN FLIPIGL-PIQESSLSLAGK

Lcyp -------------------- -------------------- -------------------- -------------------- --------------------

Lsal -------------------- -------------------- -------------------- -------------------- --------------------

Aamp -------------------- -------------------- -------------------- -------------------- --------------------

Afra -------------------- -------------------- -------------------- -------------------- --------------------

Hazt -------------------- -------------------- -------------------- -------------------- --------------------

Meda -------------------- -------------------- -------------------- -------------------- --------------------

Afos -------------------- -------------------- -------------------- -------------------- --------------------

Dmel --------------DNDAND TFTRLLTSEGPGGGVHGIST KVHFVDGANFLR-------P NSPSPRIRLDSASN--TTLR EYLQRTT-NSNPVAHSLMGK

Tcas -------------NDGQTNE IFKKLLSGGG---KVHFSAT NELHVDS------------- -----RLGVTSPTP--VGPR IRTGSFS-QMEQMHHSLANK

Pmon TPSLSSPSRKVGQSLHQTSE LYSEMLMELGVGDSAE---- -----------R-------K GRSTARLKTRDASP--ARPT PSIPSGL-PGITTSHGLQGQ

Smar -------------MEKQTNE LYQEMLKTLGVSKTIK--EP CAPERDG------------- -------------------- ----VNEWLARLSTLGLKGK

Tcal --------------GPRSNS SLTNTSIKNA---------N MAPIVETKPSPR-------P SPQT------------LQKK LSIPVGI-PMIRTHHDLQNR

Crog -------------------- -------------------- -------------------- -------------------- --------------------

Dpul --------------LPTLRI SHPQQTDSSSTGRPVS---P VSRSFDS-SILS-------P PHQSKGISARPVSP--AASQ MATSSFSGSAALPHHGLQGQ

Aver -------------------- -------------------- -------------------- -------------------- --------------------

Csin -------------------- -------------------- -------------------- -------------------- --------------------

Isca HVLSADMFSKDQMHAMFNLA HAFSLFVNKDRPLDHVLRGK VMASVFYEVSTRTSCSFAAA MQRLGGTVIHMAESSSSTQK GESLEDSIAVMAGYSDVVVL

Eaff HIIKAGMFKKEQLNAIFDLA FTFHTCVVNERPIDHILKGK VMASVFYEVSTRTACSFSAA MQRLGGKVIYSDETSSSSKK GETIEDSVQVLSSYTDVVIM

Lcyp -------------------- -------------------- -------------------- -------------------- --------------------

Lsal -------------------- -------------------- -------------------- -------------------- --------------------

Aamp -------------------- -------------------- -------------------- -------------------- --------------------

Afra -------------------- -------------------- -------------------- -------------------- --------------------

Hazt -------------------- -------------------- -------------------- -------------------- --------------------

Meda -------------------- -------------------- -------------------- -------------------- --------------------

Afos -------------------- -------------------- -------------------- -------------------- -------------YTDVVVL

Dmel HILAVDMFNKDHLNDIFNLA QLLKLRGTKDRPVDELLPGK IMASVFYEVSTRTQCSFAAA MLRLGGRVISMDNITSSVKK GESLEDSIKVVSSYADVVVL

Tcas HILSVDMFTKDQLNDIFNLA QTFRVNVSKERAVDHILKGK VMASIFYEVSTRTSCSFAAA MQRLGGGVIYMDETSSSVKK GETLEDSVAVMAGYADVVVL

Pmon HVLSVQGLSKDQLNHIFNLA QTFRVCINKERPLEHILKGK LLALMFYEVSTRTFCSFQAA IQRLGGRTVTMDTNTSSVKK GETLEDSVIMMSGYADVVVL

Smar NVLSVDMFNKEQLNVLFQFA HYLRHPLPRDKPVDSILKGK LMSLVFYEASTRTHHSFASA MHRLGGSFYCLDSMSSSIQK GESLEDTIRMVTNNCDVIVL

Tcal HVLESTMFDKDTLYSIFNLA ETFRSCVALDQSIDHILKGK VMASMFYEASTRTSLSFSAA MQRLGGRVIQMDVSTSSVKK GESLEDSVQVMAGYSDVVVI

Crog -------------------- -------------------- -------------------- -------------------- --------------------

Dpul CILTVDMFSKEQLNAIFNLA QTFRLCVQKERSLDHILKGK IMASVFYEVSTRTSCSFSAA MQRLGGRVIYMDESSSSAKK GETLEDSITVMASYSDVVVL

Aver -------------------- -------------------- -------------------- -------------------- --------------------

Csin -------------------- -------------------- -------------------- -------------------- --------------------

Isca RHPQPGAVMRAAKHCRKPVI NAGDGVGEHPTQALLDIYTI REEIGTVNGLTVTLVGDLKH GRTVHSLARLLTHYD-VQLR YVAPKNLGMPDSVKELVASR

Eaff RHPATGTVERVSHVCKRPVI NAGDGVGEHPTQALLDVYTI REEIGTVNGLTITMVGDLKH GRTVHSLAKVLTKYN-VSLR YVSPAHLKMPEDVQQFVKSQ

Lcyp -------------------- -------------------- -------------------- -------------------- --------------------

Lsal -------------------- -------------------- -------------------- -------------------- --------------------

Aamp ----XXAAGRAAEVSPRPVI NAGSGTGEHPTQALLDVFTI REEIGTVNGLTITMVGDLKN GRTVHSLARLLTLYK-VHLK YVCPPGLEMPQEVVDFVAAQ

Afra -------------------- -------------------- -------------------- -------------------- --------------------

Hazt -------------------- -------------------- -------------------- -------------------- --------------------

Meda -------------------- -------------------- -------------------- -------------------- --------------------

Afos RHPVPGAVSRAAQFCKRPVI NAGDGVGEHPTQALLDVYTI REEIGTVNGLTITMVGDLLN GRTVHSLAKLLTMYN-VRIR YVSPRNLQMPDDVKQYVAKK

Dmel RHPSPGAVARAATFSRKPLI NAGDGVGEHPTQALLDIFTI REEFGTVNGLTITMVGDLKN GRTVHSLARLLTLYN-VNLQ YVAPNSLQMPDEVVQFVHQR

Tcas RHPTPGSVMRASHHCRKPLI NAGDGVGEHPSQALLDIFTI REEIGTVNGLTITLVGDLKN GRTVHSLARLLTLYN-VQLR YVSPPSLKMPSHITSFVASK

Pmon RHPEPGAVARAAAVSRRPVI NAGDGVGEHPTQALLDVFAI REEIGTVNGLIITMVGDLKH GRTVHSLARLLTLYN-VQLR YVAPPGLGMPDYVTQYAASQ

Smar RHPQKGSAKKAAPFASVPVI NGGDGIGEHPTQALLDVFTI REEIGTVNNLTITLVGDLKN GRTVHSLAHLLTLYN-VVLR YVAAPTLGMPENVKAMISAR

Tcal RHPEPGAVDRAAKVCLKPVI NAGDGVGEHPTQALLDVFTI RSEIGTVKNLTVTMIGDLKH GRTVHSLARILTLYEGVQLR YVSPKGLGMPKNVRDYVQAA

Crog -------------------- ---SDLGEHPTQALLDLFTI RNEIGTIKGLTITMIGDLKH GRTVHSLARILTLYSGVTLH YCSPLNLRMPQEIRDFVSSH

Dpul RHPEPGAVGRAAARSSRPLI NAGDGTGEHPTQALLDVFTI REEIGTVNGLTITMVGDLKH GRTVHSLARLLTLYN-VHLR YVSPANLGMPNEVMDYIGSK

Aver -------------------- -------------------- -------------------- -------------------- --------------------

Csin -------------------- -------------------- -------------------- -------------------- --------------------

Isca GIPQEEIASLEAALPDTDVL YMTRIQKERFSSAEEYEKAC GLFVVTPQLMTRAKKRMIVM HPLPRVFEISPEF-DTDPRA AYFRQAEAGMYVRMALLAMV

Eaff GIQQVEYTDMDEAIADADVL YMTRIQKERFECTKDYEDAL GQFVLTPAHMTKAKKKMAVL HPLPRNFELSPEV-DIDPRA AYFRQAENGMYVRMALLAMV

Lcyp -------------------- -------------------- -------------------- -------------------- --------------------

Lsal -------------------- -------------------- -------------------- -------------------- --------------------

Aamp GVKQTHCTSLEEALPDSDVI YMTRVQRERFADTAEYERCA GRLVLTPQLMTLAGPRTVVL HPLPRVDEVSTEL-DGDPRA AYFRQAEYGMYVRMALLTTV

Afra -------------------- -------------------- -------------------- -------------------- --------------------

Hazt -------------------- -------------------- -------------------- -------------------- --------------------

Meda -------------------- -------------------- -------------------- -------------------- --------------------

Afos GIQQDEFESLEDALPETDVL YMTRIQKERFQSQAEYKTSI GQYVLNPHLMTKAKKRMAVL HPL----------------- --------------------

Dmel GVKQLFARDLKNVLPDTDVL YMTRIQRERFDNVEDYEKCC GHLVLTPEHMMRAKKRSIVL HPLPRLNEISREI-DSDPRA AYFRQAEYGMYIRMALLAMV

Tcas GIPQNEHSTLEEVLPDTDVL YMTRIQRERFSSQEEYDKAC GHFIVTPQLMTRAKRKMVVL HPLPRVFEISPEF-DTDPRA AYFRQAECGMYVRMALLAMV

Pmon GIHQEEFSSLEAALPDTDVL YMTRIQKERFGTPEDYERAC GHFIVTPHLMTRAKRKMIVL HPLPRNQEISPEF-DTDPRA AYFRQAEGGMYVRMALLAMV

Smar GIKQEESDSLETAIVDTDVL YMTRIQKERFATEEEYKQAC GHFVITPKLMAKAKSKMIVM HPLPRLTEISPIVCIVIPEL RYFRQAENGLYVRMALLATI

Tcal GVPQTDFPSLEKAIQDTDVL YVTRIQKERFESESEYEIAC QNYIVNDKTMHHAKEKMAVL HPLPRVFEISPEF-DNDTRA AYFRQAENGLYVRMALLAMV

Crog GIIQKDFNTMEEAIVDTDVL YVTRIQKERFQNEADYQKAC ENYLVCPKSMSHAKEK---- -------------------- --------------------

Dpul GIPQTNFSSLEDALPETDVL YMTRIQRERFATQQEYDMAC GHFVVTPHLMRLAKRRMVVM HPLPRVFEISPDF-DSDPRA AYFRQAEAGMYVRMAILAMV

Aver -------------------- -------------------S RIASLLHRKSAKQCKARWFE WLDPSIKKTEWSRQEDEKLL HLAKLMPTQWRTIAPIVGRT

Csin -------------------- -------------------- -------------------- -------------------- --------------------

Isca LGRV----MPRIMIKGGVWR NTEDEILKAAVMKYGKNQWS RIASLLHRKSAKQCKARWYE WLDPSIKKTEWAREEEEKLL HLAKLMPTQWRTIAPIIGRT

Eaff LGKC----MPRVMIKGGVWR NTEDEIMKAAVMKYGKNQWS RIASLLHRKSAKQCKARWFE WLDPSIKKTEWGKEEDEKLL HLAKLMPTQWRTIAPIVGRT

Lcyp --------MPRVMIKGGVWR NTEDEILKAAVMKYGKNQWS RIASLLHRKSAKQCKARWFE WLDPSIKKTEWSRQEDEKLL HLAKLMPTQWRTIAPIVGRT

Lsal --------MPRVMIKGGVWR NTEDEILKAAVMKYGKNQWS RIASLLHRKSAKQCKARWFE WLDPSIKKTEWSREEDEKLL HLAKLMPTQWRTIAPIVGRT

Aamp LGRA---------------- -------------------- -------------------- -------------------- --------------------

Afra --------MGRVMIKGGIWR NTEDEILKAAVMKYGKNQWS RIASLLHRKSAKQCKARWYE WLDPSIKKTEWSREEDEKLL HLAKIMPTQWRTIAPIVGRT

Hazt -------------------- -------------------- -------------------- -------------------- --------------------

Meda -------------------- -------------------S RIASLLHRKSAKQCKARWFE WLDPSIKKTEWSRQEDEKLL HLAKLMPTQWRTIAPIVGRT

Afos --------MPRVMIKGGVWR NTEDEILKAAVMKYGKNQWS RIASLLHRKSAKQCKARWFE WLDPSIKKTEWSKEEDEKLL HLAKLMPTQWRTIAPIVGRT

Dmel VGGRNTALMPRIMIKGGVWR NTEDEILKAAVMKYGKNQWS RIASLLHRKSAKQCKARWYE WLDPSIKKTEWSREEDEKLL HLAKLMPTQWRTIAPIIGRT

Tcas LGKC----MPRIMIKGGVWR NTEDEILKAAVMKYGKNQWS RIASLLHRKSAKQCKARWFE WLDPSIKKTEWSREEDEKLL HLAKLMPTQWRTIAPIIGRT

Pmon LGKC---------------- -------------------- -------------------- -------------------- --------------------

Smar LSR-----MPRIMIKGGVWR NTEDEILKAAVMKYGKNQWS RIASLLHRKSAKQ-----YE WLDPSIKKTEWTREEEEKLL HLAKLMPTQWRTIAPIIGRT

Tcal LGKC----MPRVMIKGGVWR NTEDEILKAAVMKYGKNQWS RIASLIHRKSAKQCKARWFE WLDPSIKKTEWSRDEDEKLL HLAKLMPTQWRTIAPIVGRT

Crog --------MPRVMIKGGVWR NTEDEILKAAVMKYGKNQWS RIASLLHRKSAKQCKARWFE WLDPSIKKTEWSREEDEKLL HLAKLMPTQWRTIAPIVGRT

Dpul LGKC----MPRIMIKGGVWR NTEDEILKAAVMKYGKNQWS RIASLLHRKSAKQCKARWYE WLDPSIKKTEWSREEDEKLL HLAKLMPTQWRTIAPIVGRT

Aver AAQCLERYEYLLDQAQKKEE GEDA------A-AQVDDPRK LKPGEIDPNPETKPARPDPV -------------------- --------------------

Csin -------------------- -------------------- -------------------- -------------------- --------------------

Isca AAQCLEHYEYLLDQAQKKEE DGEI----------LDDPRK LKPGEIDPNPETKPARPDPK DMDEDELEMLSEARARLANT QGKKAKRKAREKQLEEARRL

Eaff AAQCLERYEHLLDMAQKKEE GTDP----------ADDPRK LKPGEIDPNPESKPARPDPK DMDEDELEMLSEARARLANT QGKKAKRKAREKQLEEARRL

Lcyp AAQCLERYEYLLDQAQKKEE GDDA------SLSQADDPRK LKPGEIDPNPETKPARPDPV DMDEDELEMLSEARARLANT QGKKAKRKAREKQLEEARRL

Lsal ASQCLERYESLLDRAQAKES GDTT-----SSSAPPGERNK LKPGEIDPNPETKPARPDPR DMDEDELEMLSEAR------ --------------------

Aamp -------------------- -------------------- -------------------- -------------------- --------------------

Afra AAQCLERYNYLLDQAQEKEE GEKP----------VDDPRK LRPGEIDPNPETKPARPDPQ DMDEDELEMLSEARARLANT QGKKAKRKAREKQLEEARRL

Hazt -------------------- -------------------- -------------------- -------------------- --------------------

Meda AAQCLERYEYLLDQAQKKEE GDDG--------XPTEDPRK LKPGEIDPNPETKPARPDPV -------------------- --------------------

Afos AAQCLERYEHLLDMAQKKEE GGEL----------ADDPRK LKPGEIDPNPESKPARPDPK DMDEDELEMLSEARARLANT QGKKAKRKAREKQLEEARRL

Dmel AAQCLERYEYLLDQAQRKED GEDT----------MDDPRK LKPGEIDPNPETKPARPDPK DMDEDELEMLSEARARLANT QGKKAKRKAREKQLEEARRL

Tcas AAQCLERYEYLLDQAQKKEE GEDV----------TDDPRK LKPGEIDPNPETKPARPDPK DMDEDELEMLSEARARLANT QGKKAKRKAREKQLEEARRL

Pmon -------------------- -------------------- -------------------- -------------------- --------------------

Smar AAQCLEHYEFLLDQAQKKEE GDEI----------GDDPRK LKPGEIDPNPETKPARPDPK DMDEDELEMLSEARARLANT QGKKAKRKAREKQLEEARRL

Tcal AAQCLERYEYLLDQAQKKEE QGGAAAD-EAAAAAHEDPRK LKPGEIDPNPETKPARPDPQ DMDEDELEMLSEARARLANT QGKKAKRKAREKQLEEARRL

Crog ASQCLERYESLLDRAQAKDA GGGGGGDLSSSASSLGERNK LKPGEIDPNPETKPARPDPR DMDEDELEMLSEARARLANT QGKKAKRKAREKQLEEARRL

Dpul AAQCLERYEYLLDQAQRKED GEEP----------GDDPRK LRPGEIDPNPETKPARPDPK DMDEDELEMLSEARARLANT QGKKAKRKAREKQLEEARRL

Aver -------------------- -------------------- -------------------- -------------------- --------------------

Csin -------------------- -------------------- -------------------- -------------------- --------------------

Isca AALQKRRELRMAGISLP--- -PR--RRKRHAIDYNKEIPF EKRPAPGLHDTSDEVY--DP TETD-FRRLRQQHLEGELRS EKEERERRKDKQKLKQRKEN

Eaff AALQKRRELRAAGIKTR--- -LKYGTRRNRPIDYNNEIPF EKKPSQGFYDTASEVY--DK NDPD-FKRLRMDQMDLKRRD EAENKERNKDKKVMKQRKEN

Lcyp ATLQKKRELRAAGIRVR--- -MF--YVKKGKMDYNSEIPF EKKPAIGFYDTSQDAY--ET PSQN-FRHLRQHQMDEKRRD EEEQRERKKDKQKLKDRKEN

Lsal -------------------- -----YIKKGRMDYNAEVPF EKRPAIGFYDTAKEIQQEET DALDMSKGLRQHQLELKRRD EEENKLRKKDKEK---RKEN

Aamp -------------------- -------------------- -------------------- -------------------- --------------------

Afra ASLQKRRELRAAGIEIA--- -KR--RSKKRFIDYNAEIPL EKKPLPGFYDTSEEQY--DR LAPN-FKRLRQQDMDGELRS QKEEAERIKDKKK-KKENGD

Hazt -------------------- -------------------- -------------------- -------------------- --------------------

Meda -------------------- -------------------- -------------------- -------------------- --------------------

Afos AALQKRRELRAAGIQTR--- -LKYGTRRNRVIDYNKEIPF EKRPAAGFYDTASEVY--DK NDPD-FKRLRMDQMDLKRRD EEENRERQKDKKILKNRKEN

Dmel ATLQKRRELRAAGIGSG--- -NR---KRIKGIDYNAEIPF EKRPAHGFYDTSEEHL--QK NEPD-FNKMRQQDLDGELRS EKEERERKRDKQKLKQRKEN

Tcas AALQKRRELRAAGIEVN--- -SR--RKKKRGVDYNEEIPF EKRPAIGFYDTSTEIV--DP MAPD-FHKMRQQHLDGELRS EQEERERRKDKQKLKQRKEN

Pmon -------------------- -------------------- -------------------- -------------------- --------------------

Smar AALQKRRELRAAGIMLH--- -PR--RKRKRGVDYNDEVPF QKKPAPGFYDTSAEIY--EP LGPD-FRSLRQQNLDGELRS EREERERKKDKQKMKKRKEN

Tcal AALQKRRELRAAGIGGR--- -RLI-RVKKGKINYNVEIPF EKKPAAGFYDTANEEV--DE SAGR-FRRVRQDQLDEKRRD EDEMKNRKKDKDKLKQRKEN

Crog AALQKRRELRAAGVKVK--- -LR--YIKKGRMDYNAEVPF EKRPAIGFYDTASEIQEEEK HSLDLSRGLRQHQLELKRRD EEENRLRKKDK---RKGGEN

Dpul AALQKRRELRAAGMGVRRGA LSH--NRRKRGVDYNAEIPF EKQPAIGFHNTAEEQF--DP FAPN-FHRLRQQQLEGELRS VKEDRERKKDKDRLKQRKEN

Aver -------------------- -------------------- -------------------- -------------------- --------------------

Csin -------------------- -------------------- -------------------- -------------------- --------------------

Isca DLPPALLSGSSEPARKRSKL VLPEPQISESELEQVVKLGK ASETAREAAEESG------H KASEPLLADYSL-TPGGAGP LRTPKGPSLSRDTILMEAQN

Eaff DIPQELLN-NDEPARKRSKL VLPSPQISDIEMEQIVKLGR HSEAARDAVIDGD-----SN RASDTLLSDYSV-TPGAA-- LRTPRTPMV-QDKILQEAQN

Lcyp EIPSQLLN-NEEPAKKRSKL VLPEPQITDAEMEQIVKLGK ASEAARESVTSTSDNDSGAT RASDTLLADYSV-TPGSA-- MRTPRTPMPQQDKVLQEAQN

Lsal EIPKAIFE-DDEPDRKRSKL VLPEPQISDTDMEQIVKLGR ASEAAKESV----------- -SSDALLADYNV-TPGPSLA SRTPRTPMPQGDKVLQEAQN

Aamp -------------------- -------------------- -------------------- -------------------- --------------------

Afra QIPAALLK-GEEPLKKRSKL VLPEPQISETELEAVVKLGK ASEAAQQLALADG------G RPSDALLGDYNLTSPGTA-- LRTPRTPAPATDRIMQEAHN

Hazt -------------------- -------------------- -------------------- -------------------- --------------------

Meda -------------------- -------------------- -------------------- -------------------- --------------------

Afos EIPQELLN-DEEPARKRSKL VLPSPQISDIEMEQIVKLGR HSEAARDSVVDGD-----SA KASDTLLADYSV-TPGAN-- LRTPRTPMA-QDKILQEAQN

Dmel EVPTAMLQ-NMEPERKRSKL VLPTPQISDMELQQVVKLGR ASEMAKEIAGESG------I ETTDALLADYSI-TPQVA-- -ATPRTPAPYTDRIMQEAQN

Tcas DIPQAMLQ-NQEPAKKRSKL VLPEPQISDLELQQVVKLGK ASEVAREVAAESG------I ESTDTLFNDYTVAAPQAA-- AVTPRTPAPQTDRILQEAQN

Pmon -------------------- -------------------- -------------------- -------------------- --------------------

Smar DIPLALIQGNAEPVRKRSKL VLPAPQISDQELEQVVKLGK ASEAAKEAAEDNG------N KASEALLSDYKM-TPSMAN- LRTPRIPAA-QDKILQEAQN

Tcal SIPQALLN-NEEPAKKRSKL VLPEPQISDMEMEQIVKLGK ASEAAREAVASSE---GDSQ RASDTLLADYAV-TPGSA-- LRTPRTPMPQQDKILTEAQN

Crog GAPKAVFM-EDEPERKRSKL VLPEPQISDMD--------- -------------------- -------------------- --------------------

Dpul EVPSALLQ-GDQPAAKRSKL VLPEPQISDRDLEQVVKLGR ASEAAQDAARETG------Q RVSDTLLADYSL-TQV---- NRTPRTPAPAMDKILQEAQN

Aver -------------------- -------------------- -------------------- -------------------- --------------------

Csin -------------------- -------------------- -------------------- -------------------- --------------------

Isca IMALTNVDTPLKGGLNTPLH E--TEIGVATPKKAGTMTPN NLIVTPFRTPGGTLEGTTPR GG--RTPGSIP-------GS TPLSSAT------P-LRDKL

Eaff IMALQNTDTPLKGGLNTPLH NEGGDFSSMTPSREIVATPN TVLTTPYRTRDGQ-VGLTPG ----QTPGRTP-------GA TPTPGAT------P-IRDKL

Lcyp IMALTNVDTPLKGGYNNELV GNGANFGGVTPQPQAIQTPN TILATPFRTKEGQ-VGL--- -----TPRMMT--------P GSSTAGT------P-LRDKL

Lsal IMALTNVDTPLKGGV----- -------------------- -------------------- -------------------- --------------------

Aamp -------------------- -------------------- -------------------- -------------------- --------------------

Afra LAALTNVETPLKGGANAPVV D--TDFSGITPRRDVIATPN TVLGTPISVREGM------- -----TPGSRP--------Q GPAVGVT------P-LRDRL

Hazt -------------------- -------------------- -------------------- -------------------- --------------------

Meda -------------------- -------------------- -------------------- -------------------- --------------------

Afos IMALQNTDTPLKGGMNTPLH NDGGDFSGVTPQRDAVATPN TVLTTPYRTKEGQ-VGLTPS ----QTPGKTP--------V GGGMTPT------P-IRDKL

Dmel MMALTHTETPLKGGLNTPLH E--SDFSGVLPKAASIATPN TVIATPFRTQREG-GAATPG GF--QTPSSGALV-----PV KGAGGATGVVNTPAYVRDKL

Tcas VMALTHVDTPLKGGRNTPLH N--SDFSGVLPQALTIATPN TVLATPFGAQ---------- -----TPGFMT-------PQ GPQGSFT------PSVRDKL

Pmon -------------------- -------------------- -------------------- -------------------- --------------------

Smar IMALTHVDTPLKGGLNTPLH E--SDFGGVTPRPQITQTPN TVFNTPFRTPKGQ-ESLTPA ----RTPVLTPAR----VIG EQTPGAT------P-LRDML

Tcal IMALTNVDTPLKGGLNPQLH ENGGDFSSVTPQHAPIKTPN TVLTTPFRTKEGQ-IGL--- -----TPGRTP--------V NAAMGVT------P-IRDKL

Crog -------------------- -------------------- -------------------- -------------------- --------------------

Dpul LMALTHVETPLMGGANAPLV N--PDFSGATPSKEGVATPN TLLSTPFRTPGGA-AGSATP GMLSITSGATP-------RT GTLPGAT------PLVRDKL

Aver -------------------- -------------------- -------------------- -------------------- --------------------

Csin -------------------- -------------------- -------------------- -------------------- --------------------

Isca NINPEDHLD--FDTNQSAKQ FQKQTKQHLLKALSSLPAPK NDYEIVVPEDDPSLLE---- ---SASQEHMVEDQADMDSA KEHERLEKLEAERKLQSLAV

Eaff AINPDESVE-GFDGG---NL SRRENTAELKMGLSSLPTPK NDYEIVVPEDGDDAVEM--- ---QDDDNTYIEDQSDVDKR AAEIARLAREAALAKRSSAV

Lcyp AINPNEGLE----SDLSNKF QQKEVRETLKMGLSSLPAPK NDFEIVVPETDEDEGEA--- QGPDDSSLTWTVDQSEANQK IAQEFRRQREAELKRRSQAV

Lsal -------------------- -------------------- -------------------- -------------------- --------------------

Aamp -------------------- -------------------- -------------------- -------------------- --------------------

Afra SINPEEEMAIALADPKRAAQ FQK---ERLRELFTSLPTPR NLIQLEVPEEEADEGK---- ---LARDKITIEDQEEIDAR REAEIRSKYEAEWRKRSKVI

Hazt -------------------- -------------------- -------------------- -------------------- --------------------

Meda -------------------- -------------------- -------------------- -------------------- --------------------

Afos AINSEDYVE--FDGS---SL NRSEFSAELKMGLSSLPAPK NDYEIVVPEDAEDTVEM--- ---ADDKNTYIEDQADVDKR AEENARKAREEALAKRSSAV

Dmel SINPEESMG-VTETPAHYKN YQKQLKSTLRDGLSTLPAPR NDYEIVVPEQEESERI---- ---ETNSEPAVEDQADVDAR LLAEQEARRKRELEKRSQVI

Tcas NINEENTEM-VAQTPAEQRS LQNNLKEQLRLGLSSLPTPK NDYEIVVPEQEEEEKE---- ---EEKTPNIVEDQADVDAR ALEEAKIRAAQELALRSQVI

Pmon -------------------- -------------------- -------------------- -------------------- --------------------

Smar NINPEEGFG--FDEVQGNKQ FQKESREQLRRELSSLPQPV NDYEIVIPDDDPSLEND--- ---QQMEEESAEDAAELDAQ REAEIKAHMEREMKLRSQSV

Tcal AINPNEGLE-SAD-----RF HQKQLRESLQQGLSSLPQPK NDFEIVVPENEEDTDV---- ---TEDDHTWVEDQAELDQQ TEEEYRKRREQELKRRSQAV

Crog -------------------- -------------------- -------------------- -------------------- --------------------

Dpul NINPEEPTE-VM-------- -QRTLKEQLKRGLSTLPAPK NDYEIVVPEEEMDTESGTPG EGELAGTYISVEDQADIDAR IEAERKKQREAELKRRSQAL

Aver -------------------- -------------------- -------------------- -------------------- --------------------

Csin -------------------- -------------------- -------------------- -------------------- --------------------

Isca QRDLPRPLDVNASVLRPAH- TEPPLTDLQRAEELIKQEML VMQHYDALHHPTAHQRPGGG PR----------KGAPMDEA QHLAYLDRHPYRKYSREDLQ

Eaff KRNMPRPLEMNHAVLRPLN- SDPPLTDLQRAEEMIKREML VMMHYDALETPALNQQGNAG KK---------GERGIVGEA VHRGYLDRHPYREYEQEDLQ

Lcyp QRDLPRPLDMNHTILRPLN- VDPPLTELQKAEELIKREMI VMLHHDCLESPTAQQMSEAS AGKKRSSMSCLDERSIVNET QHRSYLDKHPYQKFSTEEID

Lsal -------------------- -------------------- -------------------- -------------------- --------------------

Aamp -------------------- -------------------- -------------------- -------------------- --------------------

Afra QRNLPRPNDINTNILRPQG- QEAT-NPLQRAEELIKREML TMEYYDAIKNPTESQ----- ---------------SISQS AAVQYLERNPYDNFSDKDLQ

Hazt -------------------- -------------------- -------------------- -------------------- --------------------

Meda -------------------- -------------------- -------------------- -------------------- --------------------

Afos KRDLPRPLEMNHTTLRSLN- SDPPLSDFQRAEELIKREML VMMHKDALETPTLAQQGMGG KK---------GERGIVGED AHRAYLERHPYREFQKEEIE

Dmel QRSLPRPTEVNTKILRPQS- EKQNLTEQQQAEELIKHEMI TMQLYDSVKDPVPGQ----- ------------SQHKL--E QLQSYFKANPYEDISQQELA

Tcas QRGLPRPHDVNLAVLRPNH- ESHSLTELQKAEELIKCEMV TMLQFDNLKNPNQVK----- -------------RSALAQA QQLAFLEQHPYDDFKSDDLK

Pmon -------------------- -------------------- -------------------- -------------------- --------------------

Smar QRNLPRPLDVNSNVLRPTN- VDPPLTDLQKAEELIKAEML IMLHYDTVNNPSSSQMGMTI TSEK------IESTGIVNQA QHVAYLEQHQYERFTESELE

Tcal QRDLPRPLDMNETILRPLN- SDPPLTALQKAEELIKREMV VMMHHDCVETPTAAQMGEGG NKKK------GLDRAIVNEQ LHRGYLEKHPYQKFSDEDLV

Crog -------------------- -------------------- -------------------- -------------------- --------------------

Dpul QRSLPRPHEVNNAILRPANS SDASLSDLQKAEELIKKEML TMMHYDSIRNPVNLSTEKGG GL----------SRKII--E TSQNFLSNNSYQEFEDKDID

Aver -------------------- -------------------- -------------------- -------------------- --------------------

Csin -------------------- -------------------- -------------------- -------------------- --------------------

Isca AAEEVLKAEMEVVRRGMGHG DLSLEAYSQVWDECLAQVLF LPAQNRYTRANLASKKDRIE SLDKRLEQNRGHMTREAKKA AKVEKKLRVLLGGYQAIPIA

Eaff QAKVLLEREMEIVKTGMQHG DLSLDAYTQVWEECLAQVLY LPSQQRYTRANLASKKDRIE SLEKRLDTNRTHMTKEAKRA AKVEKKLKILTGGYQSRAAG

Lcyp NAKQLLEKEIKVVKESMGHG DLSQEAYAQVWEECLAQVLF LPSQGRYTRANLAGKKDRIE SLEKRLEQNRAHMKKEAKRA VKIEEKLKILTRGYQARASA

Lsal -------------------- -------------------- -------------------- -------------------- --------------------

Aamp -------------------- -------------------- -------------------- -------------------- --------------------

Afra AAEELLAAEMEIVKNGMNHG DLSQDAYNQVWEECLAQVLY LPNQGRYTRASLVSRKDRVD ALEKRLENNRNHMANEAKRA GKLEKKLRILTAGYQGRVTT

Hazt -------------------- -------------------- -------------------- -------------------- --------------------

Meda -------------------- -------------------- -------------------- -------------------- --------------------

Afos EAKRMLQVEMDVVKVGMQHG DLSLDAYTQVWEECLSQVLY MPSQQRYFRANLASKKERIE SMEKRLDCNRQHMTKEAKKA AKIEKKLKILTGGYQSRAAG

Dmel KAKQMLTEEMEVVKERMAHG ELPLDVYAQVWQECLGQVLY LPSQHRYTRANLASKKDRLE SAEKRLETNRRHMAKEAKRC GKIEKKLKILTGGYQARAQV

Tcas LAKQLLKREMEIVKYGMGHG ELPLEAYTQVWEECLGQVLF VPNLNKYTRANLVSKKERLE SAEKRLEQNRSHMAREAKKA AKMEKKLKILTGGYQSRAQA

Pmon -------------------- -------------------- -------------------- -------------------- --------------------

Smar VAKEMLKSEMEVVKQGMGHG DLSLDAYSQVWEECLSQVLF MPSQNRYTRANLASKKDRIE SLDKRLEQNRLHMTREAKRA AKMEKRLRILCGGYQSRAQV

Tcal QAKALLEQEMTVVKEGMNHG DVSLEAYTQVWEECLAQVLY LPNQQRYTRANLANKKDRID SFKERLEQNRTHMTKEAKRA AKIEKKLKVLTGGYQARAQG

Crog -------------------- -------------------- -------------------- -------------------- --------------------

Dpul QAKELLKVEMEKVKAGMGHG DLTMEAYTQVWDECLSQVLF VPSQNRYTRASLASKKDRLE SSSRHLETNRAHMAREAKKA AKLEKKLRTLTAGYQSRAQA

Aver -------------------- -------------------- -------------------- -------------------- --------------------

Csin -------------------- -------------------- -------------------- -------------------- --------------------

Isca LIKQIQELAEQIEQTHLELK TFQALQEHESLAIPKRVEAL TEDVNRQVEREKALQKRYND LLQQKELVEEALVDVQS--- ----------------M---

Eaff LIKQLTDAADQLEQSRLELS TFKQLKISETVAIPRRLETI TEDVNLQRTRERELQSKYRD MVFKLEEAKQGRLVTET--- --PQERL---MEVEA-M---

Lcyp LTKQIQDLNEQIEQAQLELS TFSFLKDRETEAIPKRLHSL MEDVARQVEREKHLQNMFSQ ASLEIRDQVPEQQTSAS--- ----------------M---

Lsal -------------------- -------------------- -------------------- -------------------- ----------------M-SS

Aamp -------------------- -------------------- -------------------- -------------------- --------------------

Afra LAKQFEELVQQYDSATLEVD AFSRLRLNESEAIPRRLQVL EDDVEKQKQREKDLQSEYDS LVRELEELKGPELPA----- ----------------M---

Hazt -------------------- -------------------- -------------------- -------------------- --------------------

Meda -------------------- -------------------- -------------------- -------------------- --------------------

Afos LIKQLTDAAEQLEQSRLELD TFKQLKLSEEVAIPRRMDII TADVDKQKEREKDLQSHYRD SLFKLEEAKQGRFSEDT-RG --------GDMEIAND----

Dmel LIKQLQDTYGQIEQNSVSLS TFRFLGEQEAIAVPRRLESL QEDVRRQMDREKELQQKYAS LVEERDSLYSQIEHITGVRP -TAQQLL---PDQEA-M---

Tcas LIKQLADYYDQIDQANLELN TFKFLQEQEKAALPRRIQSL TEDVNRQMEREKGLQGRYGE LQGQIKELQSELEKLQQ--- --EIEVARGEQEGEAEM---

Pmon -------------------- -------------------- -------------------- -------------------- --------------------

Smar LIKQVQDTHEQIEQTQLELR TFEYLRDHEIMAIPKRIETL TENVNRQMERENSLQKRYDE LMKQQEEFKHNSISTQQIRT FH--------------M---

Tcal LIKQIHDLEDQIEQSRLDLS TFTFLKDQESMAIPRRLESI SEDVSRQTERESELQKRFQE AQYQIQQLQPQPY------- ----------------M---

Crog -------------------- -------------------- -------------------- -------------------- ----------------MSSA

Dpul LHKQTQDLIDQVEAARIELD TYSFLKKHEDAAIPSRLEKI RQDVLVQTERERELQKRFQL LQDKCFEASLAAP------- ----------------M---

Aver -------------------- -------------------- -------------------- -------------------- --------------------

Csin -------------------- -------------------- -------------------- -------------------- --------------------

Isca ----TQMLPIRFQEHLQLTN IGINAANVGFNTLTMESDKF ICVREKVGDA-AQVVIVDMA NPTSPIRRPISADSAIMNPA SRVIALK------ASRTLQI

Eaff -----AILPIKFQEHLQLTN VGITAANIGFQSLTMESDKF ICVREKVGDT-SQVVIIDMS DSSNPIRRPISADSAIMNPV SKVIALK------AQKTLQI

Lcyp ----AQSLPIKFQEHLQLTN VGINAANVGFNSLTMESDKF ICVREKVGET-NQVVIIDLA DPTNPIRRPISADSAIMNPA SKVMALK------AQKTLQI

Lsal SGASNALLPIKFQEHLQLTN VGINSSNVTFASLTMESDKY ICVREKVGDT-NQVVIIDMA DPSN-------ADSAIMNPA SKVIALK------AQKTLQI

Aamp -------------------- -------------------- -------------------- -------------------- --------------------

Afra ----AQILPIKFQEHLQLTN AGIQPANIGFANLTMESDKF ICVREKVGEAGNQVVIIDMA DPTNPIRRPIAADSAIMNPK SKVIALK------AQKTLQI

Hazt -------------------- -------------------- -------------------- -------------------- --------------------

Meda -------------------- -------------------- -------------------- -------------------- --------------------

Afos -------------------- -------------------- -------------------- -------------------- --------------------

Dmel ----TQPLPIRFQEHLQLTN VGINANSFSFSTLTMESDKF ICVREKVNDT-AQVVIIDMN DATNPTRRPISADSAIMNPA SKVIALK------AQKTLQI

Tcas ---TQQLLPIKFQEHLQLTN VGINVANISFATLTMESDKF ICVREKVGDT-SQVVIIDMG DTANPIRRPITAESAIMNPA SKVIALKGKAGVEAQKTLQI

Pmon -------------------- -------------------- -------------------- -------------------- --------------------

Smar ----AQILPIRFQEHLQLTN VGIGAANIGFSTLTMESDKF ICVREKVGDS-SQVVIIDMN DATNPIRRPISADSAIMNPA -------------TQKTLQI

Tcal ----TQILPIKFQEHLQLTN VGINATNVGFNSLTMESDKF ICVREKVGET-NQVVILDMA DPTNPIRRPISADSAIMNPA SKVIALK------AQKTLQI

Crog GGANSALLPIKFQEHLQLTN VGINPNNVTFASLTMESDKY ICVREKVGET-NQVVIIDMA DPSNPTRRPITADSAIMNPA SKVIALK------AQKTLQI

Dpul ----AQMLPIRFQEHLQLTS IGINAANIGFSTLTMESDKF ICVREKVGET-AQVVIIDLH DPTNPIRRPISADSAIMNPA SKVIALK------AGRTLQI

Aver -------------------- -------------------- -------------------- -------------------- --------------------

Csin -------------------- -------------------- -------------------- -------------------- --------------------

Isca FNIEMKSKVKAHTMTEDVVF WKWINVNTIALVTEGAVYHW SMEGDSQPQKMFDRHSSLSG CQIINYRTDAKIQWLLLIGI SAQQNRVVGAMQLYSMERKV

Eaff FNIEMKTKIKSFSMQDDVIF WKWISEKMIALVTETVVYHW SMDGDAQPVKMFERHSSLSG CQIINYRCDAAANWLLLIGI SAQQNRVVGAMQLYSVERKV

Lcyp FNIEMKSKMKAHVMQEDVTF WKWINLNTIALVTETSVYHW SMEGESTPQKVFDRHASLAG CQIINYRCDPSQNWLLLIGI SAQQNRVVGAMQLYSVERKV

Lsal FNIEMKSKMKAHIMQDDVVF WKWINLNTIALVTETTVYHW SVEGDSQPQKVFDRLSSLQG CQIINYRTDAAQNWLLLIGI TAQMNRVVGNMQLYSEERKV

Aamp -------------------- -------------------- -------------------- -------------------- --------------------

Afra FNIEIKSRMKQHNMTEEVIF WKWVTVNTIAIVTETAVYHW SMEGDSQPVKMFDRHASLNG CQIINYRADEKLQWLLLIGI TAQQNRVVGAMQLYSVERKV

Hazt -------------------- -------------------- -------------------- -------------------- --------------------

Meda -------------------- -------------------- -------------------- -------------------- --------------------

Afos -------------------- -------------------- -------------------- -------------------- --------------------

Dmel FNIEMKSKMKAHTMNEDVVF WKWISLNTLALVTETSVFHW SMEGDSMPQKMFDRHSSLNG CQIINYRCNASQQWLLLVGI SALPSRVAGAMQLYSVERKV

Tcas FNIEMKSKMKAHTMSEDVIF WKWISLNTLALVTETSVYHW SMEGDSTPVKMFDRHSSLNG CQIINYRTDPKQNWLLLVGI SAQQSRVVGAMQLYSVERKC

Pmon -------------------- -------------------- -------------------- -------------------- --------------------

Smar FNIEMKSKMKAHAMTDDVIF WKWISVNTIALVTETAVYHW GMEGDSSPQKVFDRHSSLNG CQIINYRTDHKQQWLLLIGI SAQQSRVVGAMQLYSVERKV

Tcal FNIEMKSKMKAHVMTDDVIF WKWISLNTIALVTETSVYHW SMEGDSTPQKIFDRHSSLSG CQVINYRVDSSQNWLLLIGI SAQQNRVVGAMQLYSVERKV

Crog FNIEMKSKMKAHVMQDDVVF WKWINLNTIALVTESAVFHW SVEGDSLPVKVFDRLTSLAN CQIINYRTDAAQNWLLLIGI TAQMNRVVGNMQLYSMERKV

Dpul FNIEMKSKMKAHTMTEDVVF WKWISVNTIALVTEGAVYHW PMEGDSLPQKMFDRHSSLTG CQIINYRTDAKQTWLLLIGI SAQQNRVVGAMQLYSVERKV

Aver -------------------- -------------------- -------------------- -------------------- --------------------

Csin -------------------- -------------------- -------------------- -------------------- --------------------

Isca SQPIEGHAAAFAQFKQEGNT EASTLFCFAVRTPHGGKLHI IEVGQPAAGNQAYPKKAVDV FFPPEAQNDFPVAMQMSPRH DVVYLITKYGYVHLYDLESG

Eaff SQPIEGHAAAFVQFRMEGNQ QDSNLFIFAVRGAAGGKLHI IEVGTPPTGNTPFQKKAVDV FFPPEAQNDFPVAMQVSKKN AIAYLITKYGYVHLFDIESG

Lcyp SQPIEGHAAAFVLFRMDGNP ADSTLFCFAVRTAQGGKLHI IEVGSAPSGNQPFQKKAVDV FFPPEAQSDFPVAMQVSQKF GVIYLITKYGYVHLYDLESG

Lsal SQPIEGHAASFIQFRMEGNP ADSTLFCFAVRGAQGGKLHI IEVGNSPSGNQSFQKKAVDV F------------------- --------------------

Aamp -------------------- -------------------- -------------------- -------------------- --------------------

Afra SQPIEGHAAAFAQFKMDGNP LPSTIFCFAVRSAAGGKLHI IEVGTPPAGNQPFPKKAVDV FFPPEAANDFPVAMQISPKY DVIYLITKYGYIHLYDIETG

Hazt -------------------- -------------------- -------------------- -------------------- --------------------

Meda -------------------- -------------------- -------------------- -------------------- --------------------

Afos -------------------- -------------------- -------------------- -------------------- --------------------

Dmel SQAIEGHAASFATFKIDANK EPTTLFCFAVRTATGGKLHI IEVGAPPNGNQPFAKKAVDV FFPPEAQNDFPVAMQVSAKY DTIYLITKYGYIHLYDMETA

Tcas SQPIEGHAASFATFKMEGNP EPSTLFCFAVRTVQGGKLHI IEVGQSPAGNQPFPKKTVDV FFPPEAQNDFPVAMQVSAKY DVIYLITKYGYIHMYDIESA

Pmon -------------------- -------------------- -------------------- -------------------- --------------------

Smar SQPIEGHAAAFAQFKLEGNP EISTLFCFAVRTVQGGKLHI IEVGQPPTGNQPFPKKAMDV FFPAEAQNDFPVAMQISPKH DVIYLITKYGYIHLYDIETG

Tcal SQPIEGHAAAFLQFKMEGNP HESTLFCFAVRTAQGGKLHI IEVGTPPTGNQPFQKKAVDV FFPPEAQNDFPVAMQVSTKH GVVYLITKYGYVHLYDVESA

Crog SQPIEGHAASFIPFRLEGNP ADSNLFCFAVRGAQGGKLHI IEVGSAPSGNKAFQKKAMDV FFPPEAQNDFPVAMQVSEKH GVVYLITKYGYVHLYDIESG

Dpul SQPIEGHAAAFSQFKMEGNP EFSTLFCFAVRSAQGGKLHI IEVGTPPTGNQPFAKKNVDV FFPPEAQNDFPVAMQVSPKH DVLYLITKYGYIHLYDMETG

Aver -------------------- -------------------- -------------------- -------------------- --------------------

Csin -------------------- -------------------- -------------------- -------------------- --------------------

Isca TCIYMNRISADTIFVTAPHE ASSGIIGVNRKGQVLSVSVE EENIIPYI---TNVLQNPDL ALRMAVRNNLAGAEDLFVVK FNTLFGGGQYSEAAKVAANA

Eaff ACIYTNRISADTIFVTALHE PSNGIIGVNRKGQVLSVSMD EETVVPYIQQTNTIPNNADL ALRMAVRNNLGGADQLFVAK FNDLFGRGAYSEAAKVAANA

Lcyp TCIYMNRISAETIFVTAPYE SMSGIIGVNRKGQVLSVSID EDNIINYM---NGNLQNADM ALRFAVRNNLGGADELFVRK FNTFFGNGQYSEAAKIAANA

Lsal -------------------- -------------------- -------------------- -------------------- --------------------

Aamp -------------------- -------------------- -------------------- -------------------- --------------------

Afra TCIYMNRISSETIFVTAQHE GSSGIIGVNRKGQVLSVTVD EDNIVPYV---TNVLNNTDL ALRIAVRNNLPGAEELIVRK FNNLYASGQYAEAAKVAANA

Hazt -------------------- -------------------- -------------------- -------------------- --------------------

Meda -------------------- -------------------- -------------------- -------------------- --------------------

Afos -------------------- -------------------- -------------------- -------------------- --------------------

Dmel TCIYMNRISADTIFVTAPHE ASGGIIGVNRKGQVLSVTVD EEQIIPYI---NTVLQNPDL ALRMAVRNNLAGAEDLFVRK FNKLFTAGQYAEAAKVAALA

Tcas ICIYMNRISSETIFVTAPHE STGGIIGVNRRGQVLSVSVD EDSIIRYV---NQVLHNPDL ALRIATRNNLAGAEELFVNK FQMLFTNGQYAEAAKVAANA

Pmon -------------------- -------------------- -------------------- -------------------- --------------------

Smar ICIYMNRISGETIFVTAPHE PSSGIIGVNRKGQVLSVSVD EENIIAYI---TNVLQNSDL ALRIAVRNNLAGAEELFVRK FNTLFASGQYSDAAKVAANA

Tcal VCIYMNRISAETIFVTAPHE PTNGIIGVNRKGQVLSVSVD EDNIINYI---NGNLQNPDM ALRFAVRNNLGGADELFVRK FNSSFSNGNYSEAAKIAANA

Crog FCFYMNRISAETIFVTAPHE PTHGIIGVNRKGQVLSVSVD EENVVNYI---TNNMNNPDV ALRFAVRNNLGGGDDLLINK FNSLFSSGQYSEAAKIAANA

Dpul TCIYMNRISAETVFVTAPHE PSSGIIGVNRKGQVLSVSVD EETIIQYI---TTAMQNPDL ALRIATRNNLAGAEELFVRK FNTLFQGGQYAEAAKVAANA

Aver -------------------- -------------------- -------------------- -------------------- --------------------

Csin -------------------- -------------------- -------------------- -------------------- --------------------

Isca PKGILRTPQTIQRFQQVPNQ PGQTS-PLLQYFGILLDQGQ LNKYESLELCRPVLQQGRKQ LLEKWLKDDKLECSEELGDL VKQVDPTLALSVYLRANVPN

Eaff PKGILRTPQTIAKFQQVPTQ PGQTS-PLLQYFGILLDQGQ LNKYESLELCRPVLAQGRKQ LLEKWLKEDKMECSEELGDM VKAADPTLALSVYLRANVPN

Lcyp PKGILRTPQTIGKFQQVPTQ PGQTS-PLLQYFGILLDQGQ LNKYESLELCRPVLQQGRKQ LLEKWLKEDKLECSEELGDL VKASDPTLALSVYLRANVPS

Lsal -------------------- -------------------- -------------------- -------------------- --------------------

Aamp -------------------- -------------------- -------------------- -------------------- --------------------

Afra PRGLLRTPGTIQKFQQVTAG PGQ-S-PLLQYFGILLDQGQ LNRHESLELCRPVLIQQRKQ LIEKWLKEEKLECSEELGDL VKQSDATLALSVYLRANVPM

Hazt -------------------- -------------------- -------------------- -------------------- --------------------

Meda -------------------- -------------------- -------------------- -------------------- --------------------

Afos -------------------- -------------------- -------------------- -------------------- --------------------

Dmel PKAILRTPQTIQRFQQVQTP AGSTTPPLLQYFGILLDQGK LNKFESLELCRPVLLQGKKQ LCEKWLKEEKLECSEELGDL VKASDLTLALSIYLRANVPN

Tcas PKGILRTPATIQMFQQVPTQ PGQNS-PLLQYFGILLDQGQ LNRYESLELCKPVLLQGRKQ LLEKWLKEDKLECSEELGDL VKQADSTLALSVYLRANVPA

Pmon -------------------- -------------------- -------------------- -------------------- --------------------

Smar PKGILRTPQTIARFQSVPTQ AGQTS-PLLQYFGILLDQGQ LNKFESLELCRPVLQQGRKQ LLEKWLKEDKLECSEELGDL VKQADPTLALSVYLRANVPN

Tcal PKGILRTPQTISKFQQVPTP AGQTS-PLLQYFGILLDQGQ LNKFESLELCRPVLQQGRKQ LLEKWLKEDKLECSEELGDL VKAADPTLALSVYLRANVPN

Crog PQGSLRTPQTIAKFQQVQSQ PGQTS-PLLQYFGILLDQGQ LNKYESLELCRPVIQQNRKQ LIEKWLKEDKLECSEELGDL VKATDPTLALSVYLRANVPN

Dpul PKAILRTPATIQRFQAVAAQ PGQTS-PLLQYFGILLDQGQ LNKFESLELCRPVLQQGRKQ LLEKWLKEDKLECSEELGDL VKQADPTLALSVYLRANVPN

Aver -------------------- -------------------- -------------------- -------------------- --------------------

Csin -------------------- -------------------- -------------------- -------------------- --------------------

Isca KVIQCFAETGQFQKIVLYAK KVGYVPDYVLLLRQVMRVNP DQGASFAQMLVQDE-EPLAD INQIVDVFMESNLVQQCTAF LLDALKNNRPSESNLQTRLL

Eaff KVIQCFAETGQFQKIVLYAK KVGYTPDYVFLLRNVMRVNP DSGAQFAGMLVQDD-EPLAE ITQIVDVFMEQNLVQQCTAF LLDALKNNRPAEGPLQTRLL

Lcyp KVIQCFAETNQFHKIVLYSK KVGYTPDYVFLLRTVMRSSP EQGPQFAQMLVQDDGEPLAD INQVVDIFMEQGMVQQCTAF LLDALKNNRPSEGPLQTRLL

Lsal -------------------- -------------------- -------------------- -------------------- --------------------

Aamp -------------------- -------------------- -------------------- -------------------- --------------------

Afra KVIQCFAETGQYQKIVLYAK KVGYTPDYVFLLRSVMRANP EQGLAFAQMLVQDE-EPLAD VSQVVDIFLENNLIPQCTAF LLEALKNNRPTEGPLQTRLL

Hazt -------------------- -------------------- -------------------- -------------------- --------------------

Meda -------------------- -------------------- -------------------- -------------------- --------------------

Afos -------------------- -------------------- -------------------- -------------------- --------------------

Dmel KVIQCFAETGQFQKIVLYAK KVNYTPDYVFLLRSVMRSNP EQGAGFASMLVAEE-EPLAD INQIVDIFMEHSMVQQCTAF LLDALKHNRPAEGALQTRLL

Tcas KVIQSFAETGQFQKIVLYAK KVNYTPDYIYLLRSVMRTNP DQGAAFASMLVADE-EPLAD INQIVDIFMEQNMVQQCTAF LLDALKHNRPTEGHLQTRLL

Pmon -------------------- -------------------- -------------------- -------------------- --------------------

Smar KVIQCFAETGQFQKIVLYAK KVGYTPDYVFLLRNVMRINP DQGLQFAQMLVQDD-EPLAD INQIVDIFMEHNLVQQCTAF LLDALKNNRPSEGPLQTRLL

Tcal KVIQCFAETGQFQKIVLYAK KVGYTPDYPFLLRNVMRVNP EQGAQFAQMLVQDD-EPLAD INQIVDIFMEQNMVQQCTSF LLDALKNNRPSEGPLQTRLL

Crog KVTMCFAETGQYQKIVLYAK KVNYTPDYIFILRNIMRTNP DQGAQFAQMLVVDE-EPLAE IGQIVDVFMEKNMVQQCTAF LLEALKNNRPSEGPLQTRLL

Dpul KVIQCFAETGQFSKIVLYAK KVGYTPDYVFLLRSVMRISP EQGAMFAQMLVQDD-EPLAD IAQIVDIFLEQNMVQPCTAF LLDALKNNRPAEGPLQTRLL

Aver -------------------- -------------------- -------------------- -------------------- --------------------

Csin -------------------- -------------------- -------------------- -------------------- --------------------

Isca EMNLMTAPQVADAILGNQMF THYDRAHVAQLCEKAGLLQR ALEHYTDLYDIKRAIVHTHL LNAEWLVNYFGSLSVEDSLE CLRAMLTHNLRQNLQISVQV

Eaff EMNLLAAPQVADAIMGNQMF SHYDRAHIASLCEKAGLLQR ALEHYTDLYDIKRAVVHTHL LNTDWLVNFFGTLSVEDSME CLKAMLQANIRQNLQVCVQV

Lcyp EMNLMAAPQVADAIMGNQMF THYDRAHIASLCEKAGLLQR ALEHYTDLYDIKRAVVHTHM LKPEWLVSFFGTLSVEDTME CLKTMLQANIRQNLQVVVQI

Lsal -------------------- -------------------- -------------------- -------------------- --------------------

Aamp -------------------- -------------------- -------------------- -------------------- --------------------

Afra EINLLSAPQVADAILGNNMF THYDRPHIAQLCEKAGLLQR ALEHYSDLYDIKRAVVHTHL LNPDWLLNYFGTLSVEDSLE CLKAMLTANIRQNLQICVQI

Hazt -------------------- -------------------- -------------------- -------------------- --------------------

Meda -------------------- -------------------- -------------------- -------------------- --------------------

Afos -------------------- -------------------- -------------------- -------------------- --------------------

Dmel EMNLMSAPQVADAILGNAMF THYDRAHIAQLCEKAGLLQR ALEHYTDLYDIKRAVVHTHM LNAEWLVSFFGTLSVEDSLE CLKAMLTANLRQNLQICVQI

Tcas EMNLMSAPQVADAILGNNMF THYDRAHIAQLCEKAGLLQR ALEHYTDLYDIKRAVVHTHL LPMDWLVNFFGTLSVEDSLE CLKAMLTANIRQNLQICVQI

Pmon -------------------- -------------------- -------------------- -------------------- --------------------

Smar EMNLISAPQVADAILGNQMF THYDRAHVAQLCEKAGLLQR ALEHYTDLYDIKRAVVHTHL LNSEWLVNYFGSLSVEDSLE CLRAMLQANIRQNLQICVQI

Tcal EMNLMAAPQVADAIMGNQMF THYDRPHIASLCEKAGLLQR ALEHHTDLYDIKRAVVHTHL LKPDWLVTFFGTLSVEDSLE CLKTMLNANIRQNLQVCVQI

Crog EMNLMAAPQVADAIMGNHMF TYYDKAHIAQLCEKAGLLQR ALEHYTDLYDIKRAVVHTNL LKTDWLVNFFGSLGVDDSLE CLKSMLQVNLRQNLQVCVQI

Dpul EMNLLSAPQVADAILGNQMF THYDRAHVAQLCEKAGLLQR ALEHYTDLYDIKRAVVHTHL LNPEWLVNYFGSLSVEDSLE CLRAMLQANIRQNLQIGVQI

Aver -------------------- -------------------- -------------------- -------------------- --------------------

Csin -------------------- -------------------- -------------------- -------------------- --------------------

Isca ATKYHEQLTTASLIDLFESF KSYEGLFYFLGSIVNFSQDP EVHFKYIQAACKTGQIKEVE RICRESNCYNAERVKNFLKE AKLTDQLPLIIVCDRFDFVH

Eaff ATKYHEQLTTNTLIDLFESF KSFEGLFYFLGSIVNFSQDP EVHFKYIEAACKTGQIKEVE RICRESNCYAPERVKNFLKE AKLTDQLPLIIVCDRYDFVH

Lcyp ASKYHEQLTTNALIDLFEQF KSFEGLFYFLGSIVNFSQDP EVHFKYIEAACKTGQIKEVE RICRESNCYNPERVKNFLKE AKLTDQLPLIIVCDRFDFVH

Lsal -------------------- -------------------- -------------------- -------------------- --------------------

Aamp -------------------- -------------------- -------------------- -------------------- --------------------

Afra STKYYEQLGTNALIELFESF KSFEGLFYFLGSIVNFSQDP EVHFKYIQAACKTGQIKEVE RICRESNCYNAERVKNFLKE AKLTDQLPLIIVCDRFDFVH

Hazt -------------------- -------------------- -------------------- -------------------- --------------------

Meda -------------------- -------------------- -------------------- -------------------- --------------------

Afos -------------------- -------------------- -------------------- -------------------- --------------------

Dmel ATKYHEQLTNKALIDLFEGF KSYDGLFYFLSSIVNFSQDP EVHFKYIQAACKTNQIKEVE RICRESNCYNPERVKNFLKE AKLTDQLPLIIVCDRFDFVH

Tcas ATKYHEQLTTKALIDLFESF KSYEGLFYFLGSIVNFSQDP DVHFKYIQAACKTGQIKEVE RICRESNCYNPESVKNFLKE AKLTDQLPLIIVCDRFDFVH

Pmon -------------------- -------------------- -------------------- -------------------- --------------------

Smar ATKYHEQLSTQMLIDLFESF KSFEGLFYFLGSIVNYSQEP EVHFKYIQAACKTGQIKEVE RICRESNCYNPERVKNFLKE AKLTDQLPLIIVCDRFDFVH

Tcal ATKYHEQLTTNALIDLFESF KSFEGLFYFLGSIVNFSQDP EVHFKYIEAACKTGQIKEVE RICRESNCYNPERVKNFLKE AKLTDQLPLIIVCDRFDFVH

Crog ATKYHEQLTTNALIDLFENF KSYEGLFYFLGSIVNFSQEP EVHFKYIEAACKTGQIKEVE RICRESNCYNPERVKNFLKE AKLSDQLPLIIVCDRFDFVH

Dpul ATKYHEQLSTNSLIELFESF KSFEGLFYFLGSIVNFSQDP EVHFKYIQAACKTGQIKEVE RICRESNCYNAERVKNFLKE AKLTDQLPLIIVCDRFDFVH

Aver -------------------- -------------------- -------------------- -------------------- --------------------

Csin -------------------- -------------------- -------------------- -------------------- --------------------

Isca DLVLYLYRNSLQKYIEIYVQ KVNPSRLPVVVGGLLDVDCA EEVIKGLILVVRGQFSTDEL VA------------EVEKRN RLKLLLPWLEGRLHEGCQEP

Eaff DLVLYLYRNNLQKYIEIYVQ KVNPSRLPVVVGGLLDVDCG EDIIKNLILVVRGQFNTDEL VE------------EVEKRN RLKLILPWLESRVHEGSVEP

Lcyp DLVLYLYRNNLQKYIEIYVQ KVNQSRLPVVVGGLLDVDCS EDIIKNLIHVVRGQFSTDEL VE------------EVEKRN RLKLILPWLESRIHEGCDEA

Lsal -------------------- -------------------- -------------------- -------------------- --------------------

Aamp -------------------- -------------------- -------------------- -------------------- --------------------

Afra DLVLYLYRNNLQKYIEIYVQ KVNPSRLPVVVGGLLDVDCS EDIIKNLIFVVRGQFSTDEL VA------------EVEKRN RMKLLLPWLEARVHEGSVEP

Hazt -------------------- -------------------- -------------------- -------------------- --------------------

Meda -------------------- -------------------- -------------------- -------------------- --------------------

Afos -------------------- -------------------- -------------------- -------------------- --------------------

Dmel DLVLYLYRNNLQKYIEIYVQ KVNPSRLPVVVGGLLDVDCS EDIIKNLILVVKGQFSTDEL VE------------EVEKRN RLKLLLPWLESRVHEGCVEP

Tcas DLVLYLYRNSLQKYIEIYVQ KVNPSRLPVVVGGLLDVDCA EDIIKNLILVVRGQFSTDEL VE------------EVEKRN RLKLLLPWLESRVHEGCVEP

Pmon -------------------- -------------------- -------------------- -------------------- --------------------

Smar DLVLYLYRNNLQKYIEIYVQ KVNPSRLPVVVGGLLDVDCS EDVIKNLITVVRGQFSTDEL VE------------EVEKRN RLKLILSWLEMRIHDGCTEP

Tcal DLVLYLYRNNLQKYIEIYVQ KVNPSRLPVVVGGLLDVDCS EDIIKNLITVVRGQFNTDEL VE------------EVEKRN RLKLILPWLESRIHEGSDEP

Crog DLVLYLYRSNLQKYIEIYVQ KVNPSRLPVVVGGLMDVDCS EDIIKNLIAVVKGQFNTDEL VE------------EVEKRN RLKLILPWLESRVHEGSEEP

Dpul DLVLYLYRNSLQKYIEIYVQ KVNPSRLPVVIGGLLDVDCS EDVIKSLILVVRGQFSTDEL VE------------EVEKRN RLKLLLPWLESRIHEGSVEP

Aver -------------------- -------------------- -------------------- -------------------- --------------------

Csin -------------------- -------------------- -------------------- -------------------- --------------------

Isca ATHNALAKIYIDSNNNPERF LRENQFYESRVVGRYCEKRD PHLACLAYERGQCDRELVQV CNDNSLFKSEARYLVRRRDP DLWAEVLAESNPFRRPLIDQ

Eaff ATHNAVAKIYIDSNNNAERF LKENPYYDSKVVGKYCEKRD PHLACVAYERGNCDRELIAV CNENSLFKSEARYLVKRRDM ELWAEVLNDENQFRRPLIDQ

Lcyp ATHNALAKIYIDSNNNPERF LKENPYYDSKVVGRYCEKRD PHLACVAYERGQCDRELIAV CNENSLFKSEARYLVRRRDM ELWAEVLNDDNPYRRSLIDQ

Lsal -------------------- -------------------- -------------------- -------------------- --------------------

Aamp -------------------- -------------------- -------------------- -------------------- --------------------

Afra ATHNALAKIYIDCNNNPERF LKENQYYDSHIVGKYCEKRD PHLACVAYERGKCDMELINV CNENSLFKSEARYLVKRRDP ELWAEVLLESNPYRRQLIDQ

Hazt -------------------- -------------------- -------------------- -------------------- --------------------

Meda -------------------- -------------------- -------------------- -------------------- --------------------

Afos -------------------- -------------------- -------------------- ------FKSEARYLVKRRDM DLWAEVLNDENQYRRPLIDQ

Dmel ATHNALAKIYIDSNNNPERY LKENQYYDSRVVGRYCEKRD PHLACVAYERGLCDRELIAV CNENSLFKSEARYLVGRRDA ELWAEVLSESNPYKRQLIDQ

Tcas ATHNALAKIYIDSNNNAERF LKENQWYDSRVVGRYCEKRD PHLACVAYERGQCDRELIAV CNENSLFKSEARYLVRRRDP ELWAEVLQESNPYRRQLIDQ

Pmon -------------------- -------------------- -------------------- -------------------- --------------------

Smar ATHNALAKIYIDSNNNPEKF LKENQFYDSRVVGKYCEKRD PHLACVAYERGSCDRELINV CNENSLFKSEARYLVKRRDG EMWAEVLNESNQYRRQLIDQ

Tcal ATHNALAKIYIDSNNNPERF LKENPYYDSKVVGRYCEKRD PHLACVAYERGECDRELIAV CNENSL-------------- --------------------

Crog ATHNALAKIYIDSNNNPERF LKENTFYDSAVVGRYCEKRD PHLACVAYERGECDRELISV CNENSLFKSQARYLVRRRDT DLWTEVLSDENIHRRPLIDQ

Dpul ATHNALAKIYIDSNNNPERF LKENGYYDSRVVGKYCEKRD PHLACVAYERGQCDRELIKV CNENSLFKSEARYLVRRRDP DLWVEVLNESNQFRRQLIDQ

Aver -------------------- -------------------- -------------------- -------------------- --------------------

Csin -------------------- -------------------- -------------------- -------------------- --------------------

Isca VVQTALSETQDPEDISVTVK AFMTADLPNELIELLEKIVL ENSVFSDHRNLQNLLILTAI KADRTRVMEYINRLDNYDAP DIANIAIGSELYEEAFAIFR

Eaff VVQTALAETQDPEDISVTVK AFMAADLPNELIELLEKIVL ENSVFSDHRNLQNLLILTAI KADKTKVMEYISRLDNYDAP DIANIAIGAELYEEAFAIFK

Lcyp VVQTALAETQDPEDISVTVK AFMAADLPNELIELLEKIVI DNSVFSDHRNLQNLLILTAI KADKSKVMEYIDRLDNYDAP DIANIAIQSQLYEEAFAIFK

Lsal -------------------- -------------------- -------------------- -------------------- --------------------

Aamp -------------------- -------------------- -------------------- -------------------- --------------------

Afra VVQTALSETQDPDDISVTVK AFMTADLPNELIELLEKIVL DNSAFSDHRNLQNLLILTAI KADRTRVMEYINRLENYDAP DIANIAIQSQLYEEAFAIFK

Hazt -------------------- -------------------- -------------------- -------------------- --------------------

Meda -------------------- -------------------- -------------------- -------------------- --ANIAIQSQLYEEAFAIFK

Afos VVQTALAETQDPEDISVTVK AFMASDLPNELIELLEKIVL ENSVFSDHRNLQNLLILTAI KADKTKVMEYINRLDNYDAP DIANIAIGAELYEEAFAIFK

Dmel VVQTALSETQDPDDISVTVK AFMTADLPNELIELLEKIIL DSSVFSDHRNLQNLLILTAI KADRTRVMDYINRLENYDAP DIANIAISNQLYEEAFAIFK

Tcas VVQTALSETQDPEDISVTVK AFMTADLPNELIELLEKIVL DSSVFSDHRNLQNLLILTAI KADATRVMDYINRLDNYDAP DIANIAINNHLYEEAFAIFK

Pmon -------------------- -------------------- -------------------- -------------------- --------------------

Smar VVQTALSETQDPEDISVTVK AFMTADLPNELIELLEKIVL ENSVFSDHRNLQNLLILTAI KADRTRVMEYINRLDNYDAP DIANIAIGSELYEEAFAIFR

Tcal -------------------- -------------------- -------------------- -------------------- --------------------

Crog VVQTALAETTDPDDISVTVK AFMAADLPNELIELLEKIVI DSSTFSDHRNLQNLLILTAI KADTKKVMEYIDKLDNYDAP DIANIAIQNQLYEEAFAIFK

Dpul VVQTALSETQDPEDISVTVK AFMTADLPNELIELLEKIVL DSSVFSDHRNLQNLLILTAI KADRTRVMEYINRLDNYDAP DIANIAISNQLYEEAFAIFK

Aver -------------------- -------------------- -------------------- -------------------- --------------------

Csin -------------------- -------------------- -------------------- -------------------- --------------------

Isca KFDVNTSAIQVLIEHIQNLD RAYEFAERCNEPGVWSQLAR AQLGQGLVKEAIDSFIKAGD HTAYLDVVQTAHKTGSWEDL VRYLQMARKKGRESYVESEL

Eaff KFEVNSSAVQVLIENVDNLD RAYEFAERCNEPAVWSQLGK AQLQQNLVKEAIDSYIKAND PSAYLDVVATASKTESWEDL VRYLQMARKKARDTYVESEL

Lcyp KFEVNSSAVQVLIDNVQNLD RAYEFAERCNEPAVWSQLGK AQLQKGMVKEAIDSFIKAND PTDYMNVVETASSTNHWEDL VRYLQMARKKARDTYVESEL

Lsal -------------------- -------------------- -------------------- -------------------- --------------------

Aamp -------------------- -------------------- -------------------- -------------------- --------------------

Afra KFEVNTSAIQVLIDHTRNLD RAYEFAERCNEPAVWSLLGR AQLAEGLVKEAIDSFIKADD PTVYMDVVQTAHSTGHWEDL VRYLQMARKKSRESYIESEL

Hazt -------------------- -------------------- -------------------- -------------------- --------------------

Meda KFEVNSSAVQVLIENVQNLD RAYEFAERCNEPAVWSQLGK AQLQKGLVKEAIDSFIKAND PTDYMAVVETASLTDHWEDL VRYLQMARKKARDTYVESEL

Afos KFEVNSSAVQVLIENVENLD RAYEFAERCNEADVWSQLGK AQLQQNMVKEAIDSYIKAND PRAYLDVVATATKTESWEDL VRYLQMARKKARDTYVESEL

Dmel KFDVNTSAIQVLIDQVNNLE RANEFAERCNEPAVWSQLAK AQLQQGLVKEAIDSYIKADD PSAYVDVVDVASKVESWDDL VRYLQMARKKARESYIESEL

Tcas KFDVNTSAIQVLIEQVNNLD RAYEFAERCNEPAVWSQLAK AQLNQGLVKEAIDSYIKADD PSAYMAVVETASKNNSWEDL VRYLQMARKKSRESYIESEL

Pmon -------------------- -------------------- -------------------- -------------------- --------------------

Smar KFDVNTSAIQVLIEHVQNLD RAYEFAERCNEPAVWNQLAR SQLHHGMVKEAIDSFIKADD PSAYMDVVETAHKTGSWEDL VRYLIMARKKARESYIESEL

Tcal -------------------- -------------------- -------------------- -------------------- --------------------

Crog KFEVNTSAIQVLIEKVKNLD RAYEFAERCNEPAVWSQLGK AQLKQDMVKEAIDSFIKAND PTDYMDVVETSSRTNNWEDL VRYLQMARKKARDTYIESEL

Dpul KFDVNASAIQVLIENVNNLD RAYEFAERCNEPAVWSQLAK AQLQQGLVKEAIDSFIKADD PSAYLDVVSTSHRTGSWEDL VRYLQMARKKARESFIESEL

Aver -------------------- -------------------- -------------------- -------------------- --------------------

Csin -------------------- -------------------- -------------------- ---QRRVHGGARKANSTRTW KEVCFACVDNEEFRLAQMCG

Isca IYAYAKTNRLADLEEFVSGP NHADVQRIGDRCFEDGLYEP AKLLYNNVSNFARLAITLVH LKEFQGAVDSARKANSTRTW KEVCFACVDNEEFRLAQMCG

Eaff IFAYAKTSRFADLEEFVSSP NHADVGKIGDRCFDSGMYEA AKLLYNNISNFAKLAITLVH LKEYQGAVDSARKANSTRTW KEVCFACVENEEFRLAQMCG

Lcyp IFAYAKTSRFADLEEFISSP NHADVSRIGDRCYDAKMFEA AKLLYNNVSNFAKLAITLVH LKEYQGAVDSARKANSTRTW KEVCFACVDNNEFRLAQMCG

Lsal -------------------- -------------------- -------------------- -------------------- --------------------

Aamp -------------------- -------------------- -------------------- -------------------- --------------------

Afra IYAYAKTGRFADLEEFIAGP NHADVQRIGDRCFDEKMYEP AKLLYNNVSNFARLAITLVH LKEYQGAVDSARKANSTKTW KEVCFACVESGEFRLAQSCG

Hazt -------------------- -------------------- -------------------- -------------------- --------------------

Meda IYAYAKTSRFADLEEFISSP NHADVSKIGDRCFDAGMYEA AKLLYNNVSNFAKLAITLVH LKEYQGAVDSARKANSTRTW KEVCFACVDNNEFRLAQMCG

Afos LFAYAKTSRFADLEEFISSP NHADVGKIGDRCFDAEMYEA AKLLYNNISNFAKLAITLVH LKEYQGAVDSARKANSTRTW KEVCFACVDNQEFRLAQMCG

Dmel IYAYARTGRLADLEEFISGP NHADIQKIGNRCFSDGMYDA AKLLYNNVSNFARLAITLVY LKEFQGAVDSARKANSTRTW KEVCFACVDAEEFRLAQMCG

Tcas IYSYAKTGRLADLEEFISGP NHADIQKIGDRCFDDKMYDA AKLLYNNVSNFARLAITLVH LKEFQGAVDSARKANSTRTW KEVCFACVDAEEFRLAQMCG

Pmon -------------------- -------------------- -------------------- -------------------- --------------------

Smar IYAYAKTNRLADLEEFISGP NHADIQKIGDRCFDDGMYEP AKLLYNNVSNFARLAITLVH LKEFQGAVDSARKANSTRTW KEVCFACVDSQEFRLAQMCG

Tcal -------------------- -------------------- -------------------- -------------------- --------------------

Crog IYAYAKTNRLADLEEFISSP NHADFIKIGDRCFDDKMYEA AKLFYKNVSNFAKLAITLVY LKDYQGSVESARKANCTRTW KEVCFACVENNEFRLAQMCG

Dpul IYAYARTNRLADLEEFIAGP NHADIQRIGDRCYDDGMYEP AKLLYNNVSNFARLAITLVH LKEFQGAVDSARKANSTRTW KEVCFACVDNKEFRLAQMCG

Aver -------------------- -------------------- -------------------- -------------------- --------------------

Csin LHIVVHADELEDLITYYQDR GFFEELMALLEASLGLERAH MGMFSELAILYSRYKPDKLK EHLELFWSRVNIPKVLRAAE QAHLWAELVFLYDKYEEYDN

Isca LHIVVHADELEDLIHYYQDR GFFEELIALLEAALGLERAH MGMFTELAILYSKYKPAKMR EHLELFWSRVNIPKVLRAAE QAHLWAELVFLYDKYEEFDN

Eaff LHIVVHADELEDLITFYQDK GYFEELMSLLEASLGLERAH MGMFSELSILYSRYKPEKLR EHLELFWSRVNIPKVLRAAE QAHLWAELVFLYDKYEEYDN

Lcyp LHIVVHADELDDLINYYQER GFFEELMSLLEASLGLER-- -------------------- -------------------- --------------------

Lsal -------------------- -------------------- -------------------- -------------------- --------------------

Aamp -------------------- -------------------- -------------------- -------------------- --------------------

Afra LHIVVHADELEDLINFYQDL GHFEELIQLLEAALGLERAH MGMFTELAILYSLYKTEKMR EHLELFWSRVNIPKVLRAAE KAHLWAELVFLYDKYEEFDN

Hazt -------------------- -------------------- -------------------- -------------------- --------------------

Meda LHIVVHADELEDLNNYYQER GYFEELMSLLEASLGLERAH MGMFTELAILYSKYKPGKLR EHLELFWSRVNIPKVLRAAE QAHLWAELVFL---------

Afos LHIVVHADELEDLITYYQDR GYFDELMSLLEASLGLERAH MGMFSELAILYSKYKPDKLK EHLELFWSRVNIPKVLRAAE QAHLWAELVFLYDKYEEYDN

Dmel LHIVVHADELEDLINYYQNR GYFDELIALLESALGLERAH MGMFTELAILYSKFKPSKMR EHLELFWSRVNIPKVLRAAE SAHLWSELVFLYDKYEEYDN

Tcas MHIVVHADELQDLINYYQDR GYFEELIGLLEAALGLERAH MGMFTELAILYSKYKPAKMR EHLELFWSRVNIPKVLRAAE QAHLWAELVFLYDKYEEYDN

Pmon -------------------- -------------------- -------------------- -------------------- --------------------

Smar LHIVVHADELEDLINYYQDR GYFEELINLLEAALGLERAH MGMFTELAILYSKFKPSKMR EHLELFWSRVNIPKVLRAAE QAHLWAELVFLYDKYEEYDN

Tcal -------------------- -------------------- -------------------- -------------------- --------------------

Crog LHIVVHADELEDLINYYQDK GYFEELMALLEASLGLERAH MGMFTELAILYSKYKPSRLR DHLEHFWSRVNIPKVLRAAE QAHLWAELVFLYDKYEEYDN

Dpul LHIVVHADELEDLINYYQDR GYFEELINLLEAALGLERAH MGMFTELGILYSKYKPEKMR EHLELFWSRVNIPKVLRAAE QAHLWAELVFLYDKYEEYDN

Aver -ALAMMAHPTEAWRESHFKD IITKVANIELYYKAIQFYLD YKP-------------MMLN DLLLVLAPRMDHTRAVNFFS KVGHLQLVKPYLRSVQNLNN

Csin AVLAMMAHPTEAWREGHFKD IITKVANIEMYHRAIQFYLD YKP-------------MLLN DLLLVLAPRLDHTRTVNYFT KTNHLQLVKPYLRSVQNLNN

Isca AVVTMMQHPTEAWREAHFKE IITKSKQAETAEELLSWFLD DKNYACFGACLFQCYDLLHP DVILELAWR----HGIMDFA MPYFVQVMREYV-------S

Eaff AALSMMAHPTEAWRESHFKD IMTKVANIELYYKAIQFYLD YKP-------------MLLN DLLLVLAPRLEHTRTVSYFT KTNHLQLVKPYLRSVQNLNN

Lcyp -------------------- -------------------- -------------------- -------------------- --------------------

Lsal -------------------- -------------------- -------------------- -------------------- --------------------

Aamp -------------------- -------------------- -------------------- -------------------- --------------------

Afra AVVTMMAHPTEAWREGHFKD VITKVANIELYYKAIQFYLE HKP-------------LLLN DLLLVLAPRMDHTRAVNFFD KVNHLRLVKPYLRSVQSLNN

Hazt -------------------- -------------------- -------------------- -------------------- --------------------

Meda -------------------- -------------------- -------------------- -------------------- --------------------

Afos AALAMMAHPTEAWRESHFKD IITKVANIELYYKAIQFYLD FKP-------------MLLN DLLLVLAPRLDHTRTVTFFT KANHLQLVKPYLRSVQNLNN

Dmel AVLAMMAHPTEAWREGHFKD IITKVANIELYYKAIEFYLD FKP-------------LLLN DMLLVLAPRMDHTRAVSYFS KTGYLPLVKPYLRSVQSLNN

Tcas AVLAMMAHPTEAWREGHFKD IITKVANIELYYKAIQFYLD YKP-------------LLLN DLLLVLAPRMDHTRAVAFFT KTGHLQLVKSYLRSVQNLNN

Pmon -------------------- -------------------- -------------------- -------------------- --------------------

Smar AIVTMMGHPTEAWREGHFKE IITKVANIELYYKAIQFYLD YKP-------------MLLN DLLLVLAPRMDHTRAVNFFT KVDHLQLVKPYLRSVQSLNN

Tcal -------------------- -------------------- -------------------- -------------------- --------------------

Crog AVLSMMAHPTEAWKESHFKD IITKVANIELYYKAIQFYLD YKP-------------MMLN DLLIVLAPRMDHTRAVAFFT KANHLQLVKPYLRSVQNLNN

Dpul AVLTMMQHPTEAWREGHFKD VITKVANIELYYKGIQFYLD YKP-------------MMLN DLLLVLSPRMDHTRSVNFFS KYNHLEMVKPYLRSVQNLNN

Aver KAVNEALNGLLIAEEDYNGL KTSIDAF------------- -------------------- -------------------- --------------------

Csin KVVGDKA------------- -------------------- -------------------- -------------------- --------------------

Isca KAINEALNGLLIDEEDFQGL RTSIDAFDNFDNIALAQRLE KHDLVEFRRLAAYLYKGNNR WKQSVELCKKDRLFK----- ---SKQAETAEELLSWFLDD

Eaff KAVNEALNGLFINEEDFNAL KTSIDAFDNFDNIGLAQALE KHELIEFRRIAAYLYKGNNR WKQSVELCKKDNLFKDAMEY ASESKNAEVAEDLLAYFLEH

Lcyp -------------------- -------------------- -------------------- -------------------- --------------------

Lsal -------------------- -------------------- -------------------- -------------------- --------------------

Aamp -------------------- -------------------- -------------------- -------------------- --------------------

Afra KAINEALNGLLVEEEDYQGL RASIDAFDNFDTIALAQKLE RHELIEFRRIAAYLYKGNNR WKQSVELCKKDRLFKDAMEF AAESKSSQLAEELIAWFLET

Hazt -------------------- -------------------- -------------------- -------------------- --------------------

Meda -------------------- -------------------- -------------------- -------------------- --------------------

Afos KAVNEALNGLLIDEEDYNAL KTSIDAFDNFDNIGLAQRLE KHELIEFRRIAAYLYKGNNR WSQSVALCKKDNLFKDAMEY AAESKNAEVAEDLLAYFLEH

Dmel KAINEALNGLLIDEEDYQGL RNSIDGFDNFDNIALAQKLE KHELTEFRRIAAYLYKGNNR WKQSVELCKKDKLYKDAMEY AAESCKQDIAEELLGWFLER

Tcas KAINEALNSLLIEEEDFQGL RTSIDAFDNFDNIGLAQKLE KHELTEFRRIAAYLYKGNNR WKQSVELCKKDRLFRDAMEY TAESKNQELAEELLAWFLER

Pmon -------------------- -------------------- -------------------- -------------------- --------------------

Smar KAINEALNGLLIQEEDYQGL RTSIDAFDNFDNIALAQMLE KHELIEFRRIAAYLYKGNNR WKQS-----------DAMEY AGESKNADIAEELLAWFLEE

Tcal -------------------- -------------------- -------------------- -------------------- --------------------

Crog KAVNEALNGLLIDEEDYNSL KSSIDAFENFDNISLAQTLE KHELIEFRRIGAYLYKGNNR WKQSVELCKKDNLFKDAMEY AAESKNAEVAEELLAYFLDK

Dpul KAINEALNSLLIDEEDYQGL RTSIDAFDNFDTIVLAQRLE KHELIEFRRIAAYLYKGNNR WKQSVELCKKDRLFKDAMEY ASESKNAEIAEELLAWFLEQ

Aver -------------------- -------------------- -------------------- -------------------- --------------------

Csin -------------------- -------------------- -------------------- -------------------- --------------------

Isca KNYACFGACLFQCYDLLHPD VILELAWRHGIMDFAMPYFV QVMREYVSKVGAVALAALPR IR------------------ --------------------

Eaff RAFDCFAACLFQCYDLLHPD VILELAWKHNIMDFAMPYLI QVMREYVTKVDRLEDAENLR ITEKKEEEYTPVV-LESKPQ LMLT-GPGA-YPG-------

Lcyp -------------------- -------------------- -------------------- -------------------- --------------------

Lsal -------------------- -------------------- -------------------- -------------------- --------------------

Aamp -------------------- -------------------- -------------------- -------------------- --------------------

Afra ENQECFAACLYQCYDLLKPD VVLELAWRNGITDLAMPYLI QTIREYTAKVERLEELEAQR TEEKATEEVKPMM-M--QPQ LMLTSGPAGMLPPGY-----

Hazt -------------------- -------------------- -------------------- -------------------- --------------------

Meda -------------------- -------------------- -------------------- -------------------- --------------------

Afos RAYDCFAACLFQCYDLLHPD VILELAWRHNIMDFAMPYLI QVMREYVSKVDRLEEAENLR ISEKKETEYKPVV-MESQPQ LMLT-GPAG-FPGAT-----

Dmel DAYDCFAACLYQCYDLLRPD VILELAWKHKIVDFAMPYLI QVLREYTTKVDKLELNEAQR EKEDDSTEHKNII-Q-MEPQ LMITAGPAMGIPPQYAQNYP

Tcas KAYDCFAACLYQCYDLLRPD VILELAWRHKIMDLAMPYLI QVTRELTTKVEKLEQSDAQR QSEAAEETHKPMM-I-NEPQ LMLTAGPGMGIPPQA---YV

Pmon -------------------- -------------------- -------------------- -------------------- --------------------

Smar KNYECFAACLFLCYDLLHPD VILELAWRHNIMDFAMPYMI QVMREYISKVDKLEEAENNR LEENSVHEQKPIL-I-NEPQ LMITAGPGM-MPP-------

Tcal -------------------- -------------------- -------------------- -------------------- --------------------

Crog KAYDCFAACTFQCYDLLHPD VILELAWRHGIMDFAMPYLI QVMREYTSKVERLSEAEATR LEEKQEQEYKPVV-IDNQAQ LMLT-GPMT-FNNQG-----

Dpul RNFDCFAGCLYQCYDLLHPD VILELAWRHKIIDFAMPYLI QVMREYVSKVDKLEAAESER IEENDSQSQPPSM-MIASQQ LMLT-APGF-APA-------

Aver -------------------- -------------------- -------------------- -------------------- --------------------

Csin -------------------- -------------------- -------------------- -------------------- --------------------

Isca -------------------- -------------------- -------------------- -------------------- --------------------

Eaff --GAYNNVGGVPQVGYGGA- -----PGPYGMP--GQMPSY QGYSMQTDPPTLPISSL--- ---FPSGNYPEGQIMEHPIA A---DDQKELAKDRFSSEEA

Lcyp -------------------- -------------------- -----QTDPPTIPICEL--- ---YPTGNFPIGEILEHPKP KDL-DNTT--AINRMTSEEK

Lsal -------------------- -------------------- -------------------- -------------------- --------------------

Aamp -------------------- -------------------- -----QTDPPSIPVVEM--- ---YPKNTAPKGQLMEYPAV Q---DSRS--ARDRFTSEEA

Afra -----------PQAVYPNT- ---------------YAPGY -GYGMQTDPPSIPIHEL--- ---FPNESFPVGQECEYPPS K---DERL--AKDRFTAEEK

Hazt -------------------- -------------------- -----QTDPPSVPIVDL--- ---FPAGNFPVGETCEYVDY NT--DDRT--ASNRMTNPEK

Meda -------------------- -------------------- -------------------- -------------------- --------------------

Afos --GTYNGSAAPPTTGAGATG GAGAGAGTYTIP--GQMPSY QGYSMQTEPPTVPICEL--- ---FPDGNFPVGQIMEHPIA N---DDAK--AKNRFNSEEA

Dmel PGAATVTAAGGRNMGY---- ------------------PY L----QTDPPTIPIAKL--- ---YPDGNFPEGEIVEHPTP KDMPDDRT--AKDRFTSEEK

Tcas PPQAYAQPGYAPQMPYGAY- -----------P-------- ---GMQTDPPTVPIVDL--- ---FPDQVFPIGEIQEYPTK Q---DDRT--AKDRFTSEEK

Pmon -------------------- -------------------- -----QTDPPTVPIVDL--- ---YPDGNFPIGEICEYKIN K---DDRS--AINRMNSDEK

Smar --------GYQNYGAYGAG- ----------------LPSY QGYSM--------------- -------------------- --------------------

Tcal -------------------- -------------------- -----QTYPPTIPIFDL--- ---YRGGPFPVGQQMEYPDV WN--DGRT--AQDRFGTEEK

Crog --GGVNSAGVNPGGAVGTGG NFSGGGNSFVPPPGGMNASY QGYSMQTDPPSVPISQL--- ---FPDGNFPVGQEMEYLPL K---DGRM--AMERMTQEER

Dpul ---------------YAPG- ---------------FTPGY -GYGMQTDPPTIPIVEL--- ---FPDGNFPVGEEMEYPVV G---DDRT--AKDRFTSEEK

Aver -------------------- -------------------Q ICEELESTARRLINENGLQA GLAFPTGCSLNHCAAHYTPN AGDTTVLGADDVCKIDFGTH

Csin -------------DQAGGGG HRQTRQHIQRWAKPGMKMID ICEELEKTARSLINENGLEA GLAFPTGCSRNHVE------ --------------------

Isca -------------------- -------------------- -------------------- -------------------- --------------------

Eaff RAIDRLQLDMYNDVRQAAEA HRQTRQHIQRWIKPGMKMID ICEELESTARKLINENGLEA GLAFPTGCSRNNVAAHYTPN AGDDTVLEYDDVVKLDFGTH

Lcyp KALDQAHTDFYKDLRQAAEA HRQTRKYVQSWIKPGMKMID IAEKLEACSRQMIKENGLEA GLAFPTGCSLNNCAAHYTPN AGDETVLQYDDVCKIDFGTH

Lsal -------------------- -------------------- -------------------- -------------------- --------------------

Aamp KARDTAYEEIHTELRIGAEA HRQTRQYMQKWIKPGMTMIE ICEKLESIGRATIVENGLKS GLAFPTGCSINHCAAHYTPN AGDKTVLQYDDVCKIDFGTH

Afra RTLDRSQNDSYNEARRAAES HRQTRQYMQKYIKPGMTMFQ IVEELERVSRLLIGEDGLKA GLAFPTGCSLNHCAAHYTPN AGDSTVLQYDDVCKIDFGTH

Hazt KALEISYDDMYRDIRIGAEV HRTTRQYFQKWVKAGTSCMD ICDTIENMNRKLIKENGLSA GLAFPTGCSLNHCAAHYTPN AGDKTVVEYSDVCKIDFGVH

Meda -------------------- -------------------E ICEELENTARKLINENGLQA GLAFPTGCSLNHCAAHYTPN AGDKTVLGYDDVCKIDFGTH

Afos RAIDRLQLDMYNEIRQAAEA HRQTRQYIQRWVKPGMKMID ICEELENTARRMIKENGLEA GLAFPTGCSRNHVAAHYTPN AGDNTVLEFDDVVKMDFGTH

Dmel RALDRINTDIYQELRQAAEA HRQTRQYMQRYIKPGMTMIQ ICEELENTARRLIGENGLEA GLAFPTGCSLNHCAAHYTPN AGDPTVLQYDDVCKIDFGTH

Tcas RALDRMHNDIYNEVRLAAEA HRQTRQHIQKWIKPGMTMIE ICEELENTARKLIAENGLKA GLAFPTGCSRNHCAAHYTPN AGDPTVLEYDDVVKIDFGTH

Pmon KALETSFEEMYREIRIGAEV HRTTRKYFKENVKPGMSMIE ICHMIEETNRKLIKENGLKA GLAFPTGCSINHCAAHYTPN SGDKTVLNYDDVCKIDYGIH

Smar -------------------- -------------------- -------------------- -------------------- --------------------

Tcal RAIDRAQLDMYNEVRCAAEA HRQTRQHIQKWVKPGMKMID IVQELEDTARKLIKENGLEA GLAFPTGCSINHCAAHYTPN AGDETVLGQDDVVKMDFGTH

Crog RALDRSQLDMYNEVRQAAEA HRQTRQYIQSWVKPGMKLID ICETLESTARKLIDEKGLEA GLAFPTGCSVNHCAAHFTPN AGDETVLNYDDVVKIDFGTH

Dpul KALDRAQMDIYNEVRCAAEA HRQTRQYMQRYIKPGMTMIQ ICEELESTSRRLIAENGLQA GLAFPTGCSLNHCAAHYTPN AGDTTVLQADDVCKIDFGTH

Aver INGRIIDCAWTLTFNEKYDP LVKAVREATEMGIRTAGIDV RLCDVGSAIQEVMESHEIEL DGKTYQVKSIRNLNGHSIAP YQIHAGKTVPIIRGGEATMM

Csin -------------------- -------------------- -------------------- -------------------- --------------------

Isca -------------------- -------------------- -------------------- -------------------- --------------------

Eaff IKGRIIDCAWTLAFNERYDP LLLAVKEATETGIRTAGIDA RLCDVGAAIQEVMESHEIEL DGKTYQVKSIRNLNGHSICP YQIHAGKSVPIIKGGETTRM

Lcyp INGHIIDCAWTVSFNPKYDK LLEAVKAATNAGIKEAGIDV RLCDIGEAIQEVMESYEIDL DGKTYQVKCIRNLNGHSISP YRIHAGKSVPIVKGGEATRM

Lsal -------------------- -------------------- -------------------- -------------------- --------------------

Aamp INGRIIDCAFTLTFNPKYDR LLDAVRDATNTGIKCAGIDV QLCDIGEAIQEVMESYEVEL DGKTYQVKSIRNLNGHSISP YRIHAGKTVPIVKGGEKERM

Afra VNGRIIDCAFTLTFNNKYDK LLEAVREATNTGIREAGIDV RLCDIGEAIQEVMESYEVEI DGKTYQVKPIRNLNGHSIGL YRIHAGKTVPIVKGGEVDVM

Hazt VNGRVIDCAFTLTFDNKYDK LVEAVRAATNTGIKEAGIDA RLNEIGAAIQETMESYELEL DGKLYPIKPIRNLNE----- --IHAGKTVPIVKGGERVLM

Meda INGRIIDCAWTLTFNEKYDP LVKAVREATETGIRTAGIDV RLCDVGAAIQEVMESYEIEL EGKTYQVKSIRNLNGHSISP YQIHSGKTVPIIRGGEAVMM

Afos IKGRIIDCAWTMTFNEKFDP LKKAVQEATETGIKTAGIDA RLCDVGAAIQEVMESHEIEL DGKTYQVKSIRNLNGHSISP YQIHSGKTVPIIKGGETVQM

Dmel IKGRIIDCAFTLTFNNKYDK LLQAVKEATNTGIREAGIDV RLCDIGAAIQEVMESYEIEL DGKTYPIKAIRNLNGHSISP YRIHAGKTVPIVKGGESTRM

Tcas INGRIIDCAFTHTFNPKYDK LVEAVRDATNTGIKAAGIDV QLCEIGAAIQEVMESYELEL DGKTYPIKSIRNLNGHSISP YRIHAGKTVPIVKGGEATVM

Pmon INGRVIDCAFTLTFNDRYNN LVEAVRAATNTGIREAGIDA RLCDIGAAIQETMESYEIEL NGKTYPIKCIRNLNGHSIAP YRIHAGKTVPIVKGGEAEIM

Smar -------------------- -------------------- -------------------- -------------------- --------------------

Tcal INGRIIDCAWTMNFNPKFDP LKEAVRDATEMGIKTAGIDV RLCDVGAAIQEVMESHEIEL DGKTYQIKSIRNLNGHSISP YQIHAGKTVPIIKGGEATVM

Crog IKGRIIDCAWTLTFNEKYDP LVEAVRSATEEGIKQAGVDA LLCDIGESIQEVMESHEIEL NGTTYPVKSIRNLNGHSISP YQIHSGKTVPITKGGEAVRM

Dpul VNGRIIDCAFTLSFNPKYDR LLEAVKDATNTGIRESGIDV RLCDIGAAIQEVMESYEVEI DGKTYQVKPIRNLNGHSIGP YRIHAGKTVPIVRGGETTRM

Aver EENEFYAIETFGSTGRGQ-- -------------------- -------------------- -------------------- --------------------

Csin -------------------- -------------------- -------------------- -------------------- --------------------

Isca -------------------- -------------------- -------------------- -------------------- --------------------

Eaff EENEFYAIETFGSTGKGHVH EDMECSHYMKEFDVGHIPLR LQKSKQLLNVINKNFGTLAF CRRWLDRLGETRYLMALKDL GEKGIVNPYPPLVDIKGCYT

Lcyp EEGELYAIETFGSTGKGIVH DDMECSHYMKNFDVGHVPLR LPKAKSLLGTIQKHFGTLAF CRRWLDRAGETKYLMALKNL CDVGIVDPYPPLCDIKGCYT

Lsal -------------------- -------------------- -------------------- -------------------- --------------------

Aamp EENEVYAIETFGSTGRGFVH EDMECSHYMKNFDVGHVPLR LNRSKQLLNTINQNFGTLAF CRRWLDRLGETKYLMALKDL CEKEIVQAYPPLCDVKGSYT

Afra EEGEYYAIETFGSTGKGVVH DDMECSHYMKNFSAGHVPLR LATSKQLLNVINKNFDTLAF CRRWLDRLGQTKYLLALKDL CDKGAVDAYPPLCDVRGCYT

Hazt EENEVYAIETFGSTGKGIVK DDMECSHYMKNFDVGHVPLR LAKSKQLLSVINKNFGTLAF CRRWVDRVGETKYLLALKDL CDKGLVEAYPPLCDTKGCYT

Meda EENEFYAIETFGSTGRGQ-- -------------------- -------------------- -------------------- --------------------

Afos EENEFYAIETFGSTGKGFVH EDMECSHYMKNFDVGHVPLR LQKSKQLLNTINKHFGTLAF CRRWLDRIGETRYLMALKDL GEKGIVDPYPPLVDIKGCYT

Dmel EEDEFYAIETFGSTGRGLVH DDMDCSHYMKNFDLPFVPLR LQSSKQLLGTINKNFGTLAF CKRWLDRAGATKYQMALKDL CDKGIVEAYPPLCDIKGCYT

Tcas EENEFYAIETFGSTGRGVVH DDMDCSHYMKNFESSYVPLR LQSSKTLLNIINKNFGTLAF CKRWLDRAGATKYQMALKDL CDKGVVDAYPPLCDIKGCYT

Pmon EENEVYAIETFGSTGRGYVH DDMETSHYMKNYDAGHVPLR LAKSKHLLNVINQNFGTLAF CRRWLDRLGESKYLMALKDL CEKGIIDPYPPLCDVKGCYT

Smar -------------------- -------------------- -------------------- -------------------- --------------------

Tcal EENEFYAIETFGSTGRGVVH DDMETSHYMKNFDVGHVPLR LQRSKQLLNTINKHFGTLAF CRRWLDRLGETRYLMALKDL GEKGIVEPYPPLCDVKGCYT

Crog EENEFYAIETFGSTGRGYVK DDLETSHYMKNFDAPHVQLR LQRSKQLLNCINKNFSTLAF CRRWLDRLGESRYLMALKDL GEKGIVEPYPPLCDVKGSYT

Dpul EENEFYAIETFGSTGRGYVH DDMETSHYMKNWDVSHVPLR LAKSKQLLNVINQNFGTLAF CRRWLDRLDQTKYLMGLKDL CDKGIVDPYPPLCDTKGCYT

Aver -------------------- -------------------- -------------------- ---------QESGKGSFKYA WVMDKLKAERERGITIDIAL

Csin -------------------- ---MGKEKTHVNIVVIGHVD SGKSTTTGHLIYQCGGIDKR TIEKFEKESQESGKGSFKYA WVLDKLKAERERGITIDIAL

Isca -------------------- ---MGKEKTHINIVVIGHVD SGKSTTTGHLIYKCGGIDKR TIEKFEKEAQEMGKGSFKYA WVLDKLKAERERGITIDITL

Eaff AQYEHTILLRPTCKEVISRG TDYMGKEKTHINIVVIGHVD SGKSTTTGHMIYQCGGIDKR TIEKFEKEAQEMGKGSFKYA WVLDKLKAERERGITIDIAL

Lcyp AQYEHTILLRPTCKEVLSRG DDYMPKDKIHINIVVIGHVD SGKSTTTGHLIYQCGGIDKR TIEKFEKEAQEMGKGSFKYA WVLDKLKAERERGITIDIAL

Lsal -------------------- -------------------- -------------------- -------------------- --------------------

Aamp AQYEHTIVLRPTCKEVVTRG DDY----------------- -------------------- -------------------- ---DKLKAERERGITIDIAL

Afra AQFEHTILLRPTCKEVLSRG DDYMGKEKIHINIVVIGHVD SGKSTTTGHLIYKCGGIDKR TIEKFEKEAQEMGKGSFKYA WVLDKLKAERERGITIDIAL

Hazt AQFEHTIVLRPTCKEVVTRG SDYMGKEKIHISIVVVGHVD SGKSTTTGHLIYKCGGIDKR TIEKFEKEATEMGKGSFKYA WVLDKLKAERERGITIDIAL

Meda -------------------- -------------------- -------------------- ---------QEMGKGSFKYA WVLDKLKAERERGITIDIAL

Afos AQFEHTILLRPTCKEVISRG DDYMGKEKTHINIVVIGHVD SGKSTTTGHLIYQCGGIDKR TIEKFEKEAQEMGKGSFKYA WVLDKLKAERERGITIDIAL

Dmel AQYEHTIMLRPTCKEVVSRG DDYMGKEKIHINIVVIGHVD SGKSTTTGHLIYKCGGIDKR TIEKFEKEAQEMGKGSFKYA WVLDKLKAERERGITIDIAL

Tcas AQFEHTIMLRPTCKEVVSRG DDYMGKEKTHINIVVIGHVD SGKSTTTGHLIYKCGGIDKR TIEKFEKEAQEMGKGSFKYA WVLDKLKAERERGITIDIAL

Pmon AQFEHTIVLRPTCKEVVTRG DDY----------------- -------------------- -------------------- --------------------

Smar -------------------- -------------------- -------------------- -----------MGKGSFKYA WVLDKLKAERERGITIDISL

Tcal AQFEHTILLRPTCKEVISRG DDYMGKEKFHINIVVIGHVD SGKSTTTGHLIYQCGGIDKR TIEKFEKEAQEMGKGSFKYA WVLDKLKAERERGITIDIAL

Crog AQFEHTILLRPTCKEVISRG TDYMGKEKIHINIVVIGHVD SGKSTTTGHLIYQCGGIDKR TIEKFEKEAQEMGKGSFKYA WVLDKLKAERERGITIDIAL

Dpul AQWEHTILLRPTCKEVISRG TDYMGKEKTHINIVVIGHVD SGKSTTTGHLIYKCGGIDKR TIEKFEKEAQEMGKGSFKYA WVLDKLKAERERGITIDIAL

Aver WKFETPRYYVTIIDAPGHRD FIKNMITGTSQADCGVLIVA AGTGEFEAGISKNGQTREHA LLAYTLGVKQLIVGVNKMDS TEPPYSQSRFEEIHKEVSNY

Csin WKFETPKYYVTIIDAPGHRD FIKNMITGTSQADCGVLIIA SGTGEFEAGISKNGQTREHA LLAYTLGVKQLIVAVNKMDS TEPPYSQARFEEIQKEVSGF

Isca WKFETPRYYVTVIDAPGHRD FIKNMITGTSQADCAVLVVA AGTGEFEAGISKNGQTREHA LLAYTLGVKQMIVGVNKMDT TEPPFSQTRFEEIQKEVSAY

Eaff WKFETPKYYVTIIDAPGHRD FIKNMITGTSQADCGVLIIA SGTGEFEAGISKNGQTREHA LLAYTLGVKQLIVGVNKMDS TEPPYSQARFEEIQKEVSGF

Lcyp WKFETPKYYVTIIDAPGHRD FIKNMITGTSQADCGVLIVA AGTGEFEAGISKNGQTREHA LLAYTLGVKQLIVGVNKMDS TEPAYSQSRYEEIRKEVSSY

Lsal -------------------- -------------------- -------------------- -------------------- --------------------

Aamp WKFETSKYYVTIIDAPGHRD FIKNMITGTSQADCAVLIVA SGVGEFEAGISKNGQTREHA LLAYTLGVKQMIIGVNKMDT TEPPFSEARFKEIVSEVSTY

Afra WKFETAKYYVTIIDAPGHRD FIKNMITGTSQADCAVLIVA AGVGEFEAGISKNGQTREHA LLAYTLGVKQLIVGVNKMDS TEPPFSEARFEEIKKEVSAY

Hazt WKFETTKFMVTIIDAPGHRD FIKNMITGTSQADCAVLIVA AGTGEFEAGISKNGQTREHV LLCFTLGVKQMIVAVNKMDS TEPKYSEERFKEIHKEVSAY

Meda WKFETPKFYVTIIDAPGHRD FIKNMITGTSQADCGVLIVA AGTGEFEAGISKNGQTREHA LLAYTLGVKQLIVGVNKMDS TEPPYXQGRFEEIQKEVSNY

Afos WKFETPKYYVTIIDAPGHRD FIKNMITGTSQADCGVLIIA SGTGEFEAGISKNGQTREHA LLAYTLGVKQLIVGVNKMDS TEPPYSQARFEEIQKEVSNF

Dmel WKFETAKYYVTIIDAPGHRD FIKNMITGTSQADCAVLIVA AGTGEFEAGISKNGQTREHA LLAFTLGVKQLIVGVNKMDS SEPPYSEARYEEIKKEVSSY

Tcas WKFETSKYYVTIIDAPGHRD FIKNMITGTSQADCAVLIVA AGTGEFEAGISKNGQTREHA LLAFTLGVKQLIVGVNKMDS TEPPYSEARYEEIKKEVSSY

Pmon -------------------- -------------------- -------------------- -------------------- --------------------

Smar WKFETPKFYITIIDAPGHRD FIKNMITGTSQADCAVLIVA AGTGEFEAGISKNGQTREHA LLAYTLGVKQLIVGVNKMDS TEPPYSGSRFDEIQKEVSSY

Tcal WKFETPKYYVTIIDAPGHRD FIKNMITGTSQADCGVLIIA SGTGEFEAGISKNGQTREHA LLAYTLGVKQLIVGVNKMDS TEPPYSQARFEEIQKEVSGF

Crog WKFETPKFYVTIIDAPGHRD FIKNMITGTSQADCGVLIIA SGTGEFEAGISKNGQTREHA LLAYTLGVKQLIVGVNKMDS TEPPYSQNRFEEIQKEVSGY

Dpul WKFETAKFYVTIIDAPGHRD FIKNMITGTSQADCAVLIVA GGVGEFEAGISKNGQTREHA LLAYTLGVKQLIVGVNKMDS TEPPFSEARYEEIKKEVSSY

Aver IKKIGYNPATVVFVPISGWH GDNMLAPSANMGWYKGWEIT RK-EGKASGTTLLEALDAIV PPARPTDKPLRLPLQDVYKI GGIGTVPVGRVETGIIKPGM

Csin IKKVGYNPAAVPFVPISGWH GDNMLQTSSNMSWYKGWAVE RK-EGKASGTTLLEALDSII PPARPTDKPLRLPLQDVYKI GGIGTVPVGRVETGIIKPGM

Isca IKKIGYNPATVPFVPISGWN GDNMLDASPNMGWYKGWTIE RK-SGKSEGKSLLQALDAME PPTRPTDKPLRLPLQDVYKI GGIGTVPVGRVETGLLKPGM

Eaff IKKVGYNPAAVPFVPISGWH GDNMLATSPNMSWYKGWNIE RK-EGKASGTTLLEAFDAII PPTRPTDKPLRLPLQDVYKI GGIGTVPVGRVETGIIKPGM

Lcyp IKKIGYNPDAVAFVPISGWH GDNMLTPSANMSWYKGWEVN RK-EGKASGVTLLDALDAII PPTRPTDKPLRLPLQDVYKI GGIGTVPVGRVETGILKPGM

Lsal -------------------- -------------------- -------------------- -------------------- --------------------

Aamp IKKVGYDPKTVAFVPISGWH GDNMLEASDKMPWYKGWETT RK-SGSGSGKTLLEALDNIE PPTRPSDKPLRLPLQDVYKI GGIGTVPVGRVETGTIKPGM

Afra IKKIGYNPAAVAFVPISGWH GDNMLEASDRLPWYKGWNIE RK-EGKADGKTLLDALDAIL PPSRPTEKPLRLPLQDVYKI GGIGTVPVGRVETGIIKPGM

Hazt VKKVGYNPAIVPILPISGFN GDNMLERSDKMPWWKKQKIE RK-SGAYEFETLFDVLDNIE PPTRPTDKALRLPLQDVYKI GGIGTVPVGRVETGTLKPGM

Meda IKKIGYNPATVAFVPISGWH GDNMLSPSANMGWYKGWEIT RK-EGKASGTTLLEALDAIV PPARPTDKPLRLPLQDVYKI GGIGTVPVGRVETGVLKPGM

Afos IKKVGYDPKAVAFVPISGWH GDNMLSTSTNMPWFKGWQIE RKGESVAKGTTLLEALDAII PPTRPTDKPLRLPLQDVYKI GGIGTVPVGRVETGVLKPGM

Dmel IKKIGYNPAAVAFVPISGWH GDNMLEPSTNMPWFKGWKVE RK-EGNADGKTLIDALDAIL PPARPTDKALRLPLQDVYKI GGIGTVPVGRVETGVLKPGT

Tcas IKKIGYNPAAVAFVPISGWH GDNMLEPSTKMPWFKGWNIE RK-EGKAEGKCLIEALDAIL PPTRPTDKPLRLPLQDVYKI GGIGTVPVGRVETGVLKPGM

Pmon -------------------- -------------------- ---------ETLFDALDNIE PPTRPLDKALRLPLQDVYKI GGIGTVPVGRVETGILKPGM

Smar IKKIGYNPAAVPFVPISGWN GDNMLDASPNMPWHKGWDIE RK-EGKASGKTLLEALDSII PPARPTDKPLRLPLQDVYKI GGIGTVPVGRVETGTLKPGM

Tcal IKKVGYNPAAVPFVPISGWH GDNMLATSPNMSWYKGWEIT RK-EGKASGTTLLEALDSIV PPSRPFDKPLRLPLQDVYKI GGIGTVPVGRVETGIIKPGM

Crog IKKVGYNPAAVPFVPISGWH GDNMLQASKNMSWYKGWEIT RK-EGKATGTTLLDALDSII PPARPTDKPLRLPLQDVYKI GGIGTVPVGRVETGIIKPGM

Dpul IKKIGYNPVTVPFVPISGFH GDNMIEASSNLPWYKGWAVE RK-EGKADGKTLLEALDAIV PPSRPTDKALRLPLQDVYKI GGIGTVPVGRVETGVIKPGM

Aver VVTFAPVALTTEVKSVEMHH ESLPEACPGDNVGFNIKNVS VKDIRRGYVASDSKNHPAFA TKDFLAQVIVLNHPGQISNG YSPVLDCHTAHIACKFAEIK

Csin VVTFAPNQLTTEVKSVEMHH ESLPEATPGDNVGFNIKNVS VKDIKRGYVASDSKNKPASG VSDFTAQVIVLNHPGQVSNG YSPVLDCHTAHIACKFAEIK

Isca VVTFAPANITTEVKSVEMHH EALTEAVPGDNVGFNVKNVS VKELRRGYVCGDSKDSPPKS TEEFTAQVIVLNHPGQISNG YTPVLDCHTAHIACKFREIK

Eaff VVTFAPGQLSTEVKSVEMHH ESLPEATPGDNVGFNVKNVS VKDIKRGNVASDSKNRPATG VQDFTAQVIVLNHPGQISNG YSPVLDCHTAHIACKFAEIK

Lcyp VVTFAPVNLTTEVKSVEMHH EALTEAVPGDNVGFNVKNVS VKDIRRGYVASDSKSNPAFA AKDFLAQVIVLNHPGQISNG YSPVLDCHTAHIACKFAEIK

Lsal -------------------- -------------------- -------------------- -------------------- --------------------

Aamp IVCFAPSGLTTEVKSVEMHH ESLPEAFPGDNVGFNVKNVS VKELRRGYVASDSKNKPATG CEDFTAQVIILNH------- --------------------

Afra IVTFAPANITTEVKSVEMHH ESLEQASPGDNVGFNVKNVS VKELRRGYVASDSKNNPARG SQDFFAQVIVLNHPGQISNG YTPVLDCHTAHIACKFAEIK

Hazt VVTFAPTGLTTEVKSVEMHH EALVEATPGDNVGFNVKNVP VKDLKRGFVTSDSKNDAAKA AQDFVAQVIVLNHPGQIQAG YSPVVDCHTAHIACKFAELV

Meda VVTFAPVNLTTEVKSVEMHH EALPEAVPGDNVGFNIKNVS VKDIRRGYVASDSKNQPAFA AKDFLAQVIVLNHPGQISNG YSPVLDCHTAHIACKFAEIK

Afos LVTFAPTQLTTEVKSVEMHH ESLAEAVPGDNVGFNVKNVS VKDIKRGNVASDNKNKPATG VSDFTAQVIVLNHPGQISNG YSPVLDCHTAHIACKFAEIK

Dmel VVVFAPANITTEVKSVEMHH EALQEAVPGDNVGFNVKNVS VKELRRGYVAGDSKANPPKG AADFTAQVIVLNHPGQIANG YTPVLDCHTAHIACKFAEIK

Tcas VVVFAPANITTEVKSVEMHH EALPEAVPGDNVGFNVKNVS VKELRRGYVAGDSKASPPKG AADFLAQVIVLNHPGQISNG YTPVLDCHTAHIACKFAEIK

Pmon VVNFAPTGPTTEVKSVEMHH EALTEAVPGDNVGFNVKNVS VKDLKRGFVASDSKNDPAKE AADFTAQVIVLNHPGQIQAG YSPVLDCHTAHIACKFAELL

Smar VVTFAPVNLTTEVKSVEMHH EALQEAMPGDNVGFNVKNVS VKELRRGYVAGDSKNNPPKA VSDFTAQVIVLNHPGQISNG YTPVLDCHTAHIACKFAEIK

Tcal VVTFAPVQLTTEVKSVEMHH ESLPEATPGDNVGFNVKNVS VKDIKRGFVASDSKNKPATG CKDFTAQVIVLNHPGQICNG YSPVLDCHTAHIACKFLEIK

Crog VVTFAPNNLTTEVKSVEMHH ESLKEATPGDNVGFNVKNVS VKDIKRGYVASDSKNKPAVG CKDFTAQVIVLNHPGEICNG YTPVLDCHTAHIACKFNEIK

Dpul VVTFAPCQLTTEVKSVEMHH EALEEAVPGDNVGFNVKNVS VKELRRGFVAGDSKNNPPKG AADFYAQVIVLNHPGQIGNG YTPVLDCHTAHIACKFAEIK

Aver EKVDRRTGKSTEDNPKSIKS GDAAIVNLVPSKP-MCVEAF VDFPPLGR------------ -------------------- --------------------

Csin EKVDRRTGKSTEDNPKFIKS GDAAIVLLTPSKP-MCVEPF SQFPPLGRFAVRDMRQTVAV GVIKE--------------- ---------EDRQYKKIHAR

Isca EKCDRRSGKKLEDNPKFIKS GDAAIIDLVPSKP-MCVETF TDFPPLGRFAVRDMRQTVAV GVIKSVKPKEASG-GKVTKA AEKAQKKK---MPITKIFAR

Eaff EKVDRRTGKSTEDNPKFIKS GDAAIVRLVPSKP-MCVEAF SDFPPLGRFAVRDMRQTVAV GVIKATTPKDVA--GSATKS AAKAQKKK---MPIKKIHAR

Lcyp EKVDRRTGKSVEDNPKFIKS GDAAIVNLVPSKP-MCVEAF SDFPPLGRFAVRDMRQTVAV GVIKSVQAKDVS--GTTTKA AQKAQKKK---MPIKKIFAR

Lsal -------------------- -------------------- -------------------- -------------------- --------------------

Aamp -------------------- -------------------- -------------------- -------------------- -----------MPIQKVHAR

Afra EKCDRRTGKTTEAEPKFIKS GDAAMITLVPSKP-LCVEAF SDFPPLGRFAVRDMRQTVAV GVIKSVNFKDPTA-GKVTKA AEKAGKKK---MAIQKIHAR

Hazt TKIDRRTGKEIESNPKAVKS GDSCIVKMVPSKP-MCVESF QQYPPLGRFAVRDMKQTVAV GVIKEVNKKEASG-GKTTKA AEKALKKK---MSVTKVFAR

Meda EKVDRRTGKSVEDNPKSIKS GDAAIVNLVPSKP-MCVEAF SDFPPLGR------------ -------------------- --------------------

Afos EKVDRRTGKSTEDNPKFIKS GDAAIVKLIPSKP-MCVEPF SDFAPLGRFAVRDMRQTVAV GVIKATTPKEVA--GNLTKA AKTAASKKK--MPIKKIHAR

Dmel EKVDRRSGKTTEENPKFIKS GDAAIVNLVPSKP-LCVEAF QEFPPLGRFAVRDMRQTVAV GVIKAVNFKDASG-GKVTKA AEKATKGKK--MTIKAIKAR

Tcas EKVDRRSGKTTEENPKAIKS GDAAIVTLVPSKP-MCVESF QEFPPLGRFAVRDMRQTVAV GVIKSVNFKDAGA-GKVTKA AEKAAKKK---MPMKTIFAR

Pmon TKIDRRTGKELEAGPKHVKS GDSCIVKDGSQQAHVCGDLP TVCSSW----------TFLL CVT----------------- -----------MSITKVFAR

Smar EKCDRRSGKTVEENPKAIKS GDAAIVNLVPSKP-MCVESF SDFPPLGRFAVRDMRQTVAV GVIKSVNQKEATS-GKVTKA AEKAQKKK------------

Tcal EKVDRRTGKATETNPKSIKS GDAGIVELVPSKP-MCVEAF ADFPPLGRFAVRDMRQTVAV GVIKSVTLKELS--GTTTKA GAKAQKKK---MPIKKIHAR

Crog EKVDRRTGKSTESNPKSIKS GDAGIVELVPSKP-MCVEAF SDFPPLGRFAVRDMRQTVAV GVIKSMCPKDIS--GTTTKA AQKAQKKK---MPIKEIHAR

Dpul EKCDRRTGKTTEENPKFIKS GDAAMITLVPSKP-LCVESF SDFPPLGRFAVRDMRQTVAV GVIKSVNFKDLGAGGKVTKA AEKAGKKK---MSIQKIFAR

Aver -------------------- -------------------- -------------------- -------------------- --------------------

Csin QIYDSRGNPTVEVDLTTEKG IFRAAVPSGASTGIYEALEM RDKAET-WHGKGVQKAVHNV NNLIAPEIVAKCLDPVEQEK IDSLML---ATDNKNKLGAN

Isca QIFDSRGNPTVEVDLTTEKG LFRAAVPSGASTGIHEALEL RDGDKSMYHGKSVFKAVDNV NKVIAPQLIAKNFDVREQFQ IDEFMIELDGTENKGNLGAN

Eaff QIYDSRGNPTVEVDLTTEKG IFRAAVPSGASTGIYEALEL RDKAET-WHGKGVTKAVNNV NNIIAPELIKNCLDPVEQEK IDELMLKLDGTDNKNSLGAN

Lcyp QIYDSRGNPTVEVDLTTEKG IFRAAVPSGASTGIHEALEL RDNEKGKWHGKGVLKAVDNV NNVIAPALVNANLDPVDQTA IDELMLKLDGTDNKSSLGAN

Lsal -------------------- -------------------- -------------------- -------------------- --------------------

Aamp SIFDSRGNPTVEVDLFTEKG LFRAAVPSGASTGVHEALEL RDGDKSKYMGKSVFKAVENI NKIIVPELLKKNLEPTQQKE IDEFMIQLDGTPNKSKLGAN

Afra MIFDSRGNPTIEVDLVTEQG LFRAAVPSGASTGIYEALEL RDGVKAEYHGKGVFKAIRNV NEIIAPALIAKGLDVTQQEQ IDQFMLDLDGTPNKEKLGAN

Hazt SIFDSRGNPTVEVDLYTAKG LFRAAVPSGASTGVHEALEM RDGDKKLYHGKSVFNAVKNV NDVIGPELIKSGLQVTQQKE IDEFMIKLDGTENKSKLGAN

Meda -------------------- -------------------- -------------------- -------------------- --------------------

Afos QIYDSRGNPTVEVDLTTEKG IFRAAVPSGASTGIYEALEL RDKGDT-WHGKGVSKAVDNV NNLIAPELVKANLDPVEQVN IDEMMLKLDGTDNKNKLGAN

Dmel QIYDSRGNPTVEVDLTTELG LFRAAVPSGASTGVHEALEL RDNDKANYHGKSVLKAVGHV NDTLGPELIKANLDVVDQAS IDNFMIKLDGTENKSKFGAN

Tcas QIFDSRGNPTVEVDLITDLG LFRAAVPSGASTGVHEALEL RDNDKANYHGKSVQKAINNV NTIIAPELIKSNLEVTQQTE IDDLMLKLDGTENKSKLGAN

Pmon TIFDSRGNPTVEVDLYTHKG LFRAAVPSGASTGVHEALEM RDGDKSKYHGKSVFKAVNNV NSIIAPEIIKSGLKVTQQKE CDDFMCKLDGTENKSRLGAN

Smar -------------------- -------------------- -------------------- -------------------- --------------------

Tcal QIFDSRGNPTVEVDLTTEKG IFRAAVPSGASTGIYEALEL RDQDKSKYMGKGVSKAVDNI NTIIAPKLVEMSLDPVDQTN VDKVMLDMDGTDNKNKLGAN

Crog QIYDSRGNPTVEVDLTTERG IFRAAVPSGASTGVHEALEL RDKDST-WHGKSVLKAVKNV NEVLGPELVKKEFDPVKQEE IDDFMISLDGTENKSKFGAN

Dpul MIFDSRGNPTVEVDLTTEKG LFRAAVPSGASTGIHEALEL RDDIKTEYHGKGVKKAVSNI NDLIAPALLKENLQVTDQEA IDNFMIKLDGTENKSKFGAN

Aver -------------------- -------------------- -------------------- -------------------- --------------------

Csin AILGVSMAVCKAGAAHKGVP LYRHIADLAGVKDVFMPVPC LNVINGGSHAGNKLAMQEFM VLPLGASCFSRRVHGGSELY HHLKALIKKKYGLDATAVGD

Isca AILGVSLAVCRAGAAQKGVP VYRHIADLAGNSKVILPVPA FNVINGGSHAGNKLAMQEFM ILPTGASSFSEAMRMGSEVY HHLKNVIKKK----------

Eaff AILGVSMAVCKAGAAHKGVP LYRHIADLAGVKDVIMPVPA LNVINGGSHAGNKLAMQEFM VLPTGATCFSEAMRMGSELY HHLKSIIKKKYGLDATAVGD

Lcyp AILGVSMAVCKAGAAHKGVP LYRHIADLAGVKQVMMPVPA FNIINGGSHAGNKLAMQEFM ILPVGATSFTEAMKMGTETY HHLRTLIKAKYGLDATAVGD

Lsal -------------------- --------------MMPVPA FNVINGGSHAGNKLAMQEFM ILPTGAPSFTEAMRMGSEIY HHLKALIKKKYGLDATAVGD

Aamp AILGVSLAVCKAGAAAKGIP LYKHIADLAGNKNIVLPTPA FNVINGGSHAGNKLAMQEFM ILPTGAANFTEAMRMGSEVY HNLKAVIKKKFGLDATAVGD

Afra AILGVSLAACKAGAKQKGVP LYRHIADLAGTPEIILPTPA FNVINGGSHAGNKLAMQEFM ILPTGAGSFSEAMRMGSEIY HHLKSVIKSRFGLDATAVGD

Hazt AILGVSLAVCKAGAAELGLP LYRHLANLANYKDVILPVPA FNVINGGSHAGNKLAMQEFM ILPTGASSFTEAMRMGTEIY HHLKAVIKSKFGLDATAVGD

Meda -------------------- -------------------- -------------------- -------------------- --------------------

Afos AILGVSMAVCKAGAAHKGVP LYRHIADLAGVKDVIMPVPA LNVINGGSHAGNKLAMQEFM VLPTGASCFSEAMRMGSELY HHLKGIIKKKYGLDATAVGD

Dmel AILGVSLAVAKAGAAKKGVP LYKHIADLAGNKEIILPVPA FNVINGGSHAGNKLAMQEFM ILPTGATSFTEAMKMGSEVY HHLKNVIKAKFGLDATAVGD

Tcas AILGVSLAVAKAGAAKKGVP LYKHLADLAGNKNIILPVPA FNVINGGSHAGNKLAMQEFM ILPTGAKSFTEAMKMGSEVY HHLKNVIKAQFGLDATAVGD

Pmon AILGVSLAICKAGAAELGIP LYRHIANLANYSDVILPVPA FNVINGGSHAGNKLAMQEFM ILPTGATSFTEAMRMGSEVY HHLKAVIKGRFGLDATAVGD

Smar -------------------- -------------------- -------------------- -------------------- --------------------

Tcal AILGVSMAVCKAGAAHKGVP LYQHIADLAGVKEVKMPVPA FNVINGGSHAGNKLAMQEFM ILPTGAANFTEAMRMGSEIY HHLKAGIKAKYGLDATAVGD

Crog AILGISMAVCKAGAAHKGVP LYRHIADLSGVKEVMMPVPA FNVINGGSHAGNKLAMQEFM ILPTGAPSFSEAMRMGSEIY HHLKALIKKKYGLDATAVGD

Dpul AILGVSLAACKAGATHKGVP LYRHIADLAGTPEIILPTPA FNVINGGSHAGNKLAMQEFM ILPTGASSFTEAMRMGTETY HHLKKVINARFGLDATAVGD

Aver -------------------- -------------------- -------------------- -------------------- --------------------

Csin EGGFAPNFQNNGEALDLLVE AIKKSGYEGKIMIGMDIAAA EFFK-NGK-YDLDFKNPNTK EADWISSDALLEMYKGFIKD YPVVSIEDPFDQDDWAGYAK

Isca -------------------- -------------------- -------------------- -------------------- --------------------

Eaff EGGFAPNFQNNGEALELLVE AIKKSGYEGKIQIGMDIAAA EFYK-NGK-YDLDFKNPESN EADWINSDQLGEMYKGFIKD YPVVSIEDPFDQDDWEGYTK

Lcyp EGGFAPNFQNNADAIKLCTD AIEKAGYTGKIKIGMDVAAS EFYK-DGK-YDLDFKNPNSN PDDWITSDKLADMYRGFIAD APVVSIEDPFDQDDWQGWSA

Lsal EGGFAPNFQANGEAIDLLVG AIEKAGYTGKIKIGMDVAAS EFYK-NGK-YDLDFKNEESK EADWLTSEALGEMYKGFIKD APVISIEDPYDQDDWEGRTA

Aamp EGGFAPNIQNNEEALTMLME AIEKAGYTGKVDIGMDVAAS EFHKADGQ-YDLDFKTENND GSKVISGEALADMYRDWAKR YPIVSVEDPFDQDDWDSWVK

Afra EGGFAPNILNNKDGLSLIVT AIEKAGYTGKVEIGMDVAAS EFYR-EGK-YDLDFKNPNSD KTAWLSGQELANLYQEFIKE FPVTSIEDPFDQDDWDAWSS

Hazt EGGFAPNILNNKDALSLITD AIERAGYTGKIEIGMDVAAS EFYK-GNNIYDLDFKTENND GSQKISGEQLCDLYTSFCKD FPITSIEDPFDQDDWDNWTR

Meda -------------------- -------------------- -------------------- -------------------- --------------------

Afos EGGFAPNFQNNGEALELLVE AIKKSGYEGKIQIGMDIAAS EFFK-NGK-YDLDFKNPESK EADWITSDQLGEMYKGFIKD YPVVSIEDPFDQDDWEGYTK

Dmel EGGFAPNIQSNKEALNLISD AIAKAGYTGKIEIGMDVAAS EFYK-DGQ-YDLDFKNEKSD KSQWLPADKLANLYQEFIKD FPIVSIEDPFDQDHWEAWSN

Tcas EGGFAPNIQNNEDALNLIIE AIKKAGYEGKIDIGMDIAAS EFYK-DGQ-YDLDFKNPKSD KSKWISGEKLLEMYKGFIKK FPVVSIEDPFDQDDWSSWSA

Pmon EGGFAPNILNNKDALTLIQE SIEKAGYTGKIEIGMDVAAS EFYK-GENIYDLDFKTANND GSQKITGDQLRDMYMEFCKE FPIVSIEDPFDQDDWENWTK

Smar -------------------- -------------------- -------------------- -------------------- --------------------

Tcal EGGFAPNFQNNKEALELLVG AIEKAGYSGKIQIGMDVAAS EFRK-GDK-YDLDFKNPESK EADWISSDALGEMYKGFIKD FPVTSIEDPFDQDDWEGWTK

Crog EGGFAPNFQANGEAIDLLVG AIEKAGYTGKIKIGMDVAAS EFYK-KGK-YDLDFKNDASK EADWISSDALGEMYKGFIKD APVLSIEDPYDQDDWEGWTK

Dpul EGGFAPNILNNKDALDLITS AIEKAGYTGKIEIGMDVAAS EFHK-NGK-YDLDFKNPASD PATYLESNKLAELYQEFIKD FPMVSIEDPFDQDDWAAWTS

Aver -------------------- -------------------- -------------------- -------------------- --------------------

Csin LTKETSIQIVGDDLLVTNPK RIQTAIEKKACNGLLLKVNQ IGSVTESIKAHNMAKAEGWG TMVSHRSGETEDCFIADLVV GLGTGQIKTGAPCRSERLAK

Isca -------------------- -------------------- -------------------- -------------------- --------------------

Eaff LTSETSIQIVGDDLLVTNPK RIQTAIDKKACNALLLKVNQ IGSVTESIRAHQMAKAQGWG TMVSHRSGETEDCFIADLVV GLGTGQIKTGAPCRSERLAK

Lcyp LTGSTPIQIVGDDLTVTNPK RIQMAVDKKACNCLLLKVNQ IGSVTESIAAHNLAKANGWG TMVSHRSGETEDCFIADLVV GLCTGQIKTGAPCRSERLAK

Lsal LTSQTDIQIVGDDLTVTNPK RIQMAVDKKSCNCLLLKVNQ IGSVTESIRAHNLAKSNGWG TMVSHRSGETEDCFIADLVV GLCTGQIKTGAPCRSERLSK

Aamp YTASVEHQVVGDDLTVTNPQ RIQMGIDKKACNCLLLKVNQ IGSVTESIAAHQLAKSDGWG TMVSHRSGETEDPFIADLVV GLSTGQIKTGAPCRSERLCK

Afra FTANTNIQIVGDDLTVTNPI RIQTGIDKKACNCLLLKVNQ IGSVTESIRAHKLAKANGWG TMVSHRSGETEDSFIADLVV GLSTGQIKTGAPCRSERLAK

Hazt MTAGTNIQIVGDDLTVTNPK RITTAVEKKACNCLLLKVNQ IGSVTESINAHLLAKSNGWG TMVSHRSGETEDTFIADLVV GLCTGQIKTGAPCRSERLAK

Meda -------------------- -------------------- -------------------- -------------------- --------------------

Afos LTSETSIQIVGDDLLVTNPK RIQTAIDKKACNALLLKVNQ IGSVTESIKAHQMAKGQGWG TMVSHRSGETEDCFIADLVV GLGTGQIKTGAPCRSERLAK

Dmel LTGCTDIQIVGDDLTVTNPK RIATAVEKKACNCLLLKVNQ IGTVTESIAAHLLAKKNGWG TMVSHRSGETEDSFIGDLVV GLSTGQIKTGAPCRSERLAK

Tcas ITSSTDIQIVGDDLTVTNPK RIQTAVEKKACNCLLLKVNQ IGSVTESIKAHLLAKSNGWG TMVSHRSGETEDTFIADLVV GLSTGQIKTGAPCRSERLAK

Pmon MTSATNIQIVGDDLTVTNPK RIATAVEKKACNCLLLKVNQ IGSVTESIDAHLLAKKNGWG TMVSHRSGETEDCFIADLVV GLCTGQIKTGAPCRSERLAK

Smar -------------------- -------------------- -------------------- -------------------- --------------------

Tcal LTGETSIQIVGDDLTVTNPK RIQMAVDKKSCNCLLLKVNQ IGSVTESIEAHNLAKKNGWG TMVSHRSGETEDCFIADLVV GLCTGQIKTGAPCRSERLAK

Crog LTAETDIQIVGDDLTVTNPK RIQMAVEKKACNCLLLKVNQ IGSVTESIRAHNLARSNGWG TMVSHRSGETEDCFIADLVV GLCTGQIKTGAPCRSERLAK

Dpul LTGNTPIQIVGDDLTVTNPK RIQMAVDCKACNCLLLKVNQ IGTVTESIAAHKLAKANGWG TMVSHRSGETEDSFIGDLVV GLSTGQIKTGAPCRSERLAK

Aver -------------------- -------------------- -------------------- -------------------- ------------VLWALFRD

Csin YNQ----------------- -------------------- -------------------- -------------------- --------------------

Isca -------------------- -------VEMPQSSFP---P ALSPQCIALLKESIHLF--- ----EEPVQEQQVHVFIVLG ASGDLAKKKIYPTLWALFRD

Eaff YNQLLRIEEELGPGAKFAGV NFRNPQ---MIDAEC----T EGNPEGLQLLRESLRKVQLK --D-----EGVHSNVFVVFG ASGDLARKKIYPTLWALYRD

Lcyp YNQLLRIEEELGSAAKFAGE KFRNPCK--MDQNQWPLCLD CGNSEGLELLRKSLKSVQL- ----EAHVKEDACHTFVICG ASGDLAKKKIYPTLWAIFKD

Lsal YNQLLRIEEELGSNAKYVGD KFRMPF-------------- -------------------- -------------------- --------------------

Aamp YNQILRIEEELGANAKYAGK NFRRPQ-------------- -------------------- -------------------- --------------------

Afra YNQILRIEEELGSNAKYAGK NFRHPL---MG-SKTKGSRT SCPQDSLDLIRRSLRSTHMS ---NSERFEESTPHVFIVLG ASGDLAKKKIYPTLWWLFRD

Hazt YNQILRIEEELGASAKYAGK SFRMPC-------------- -------------------- -------------------- --------------------

Meda -------------------- -------------------- -------------------- -------------------- ------------VLWALFRD

Afos YNQLLRIEEELGSGAKFAGA NFRCPQ---MTSSNI----S SGEPADL------------- ------------SNVFVVFG ASGDLARKKIYPTLWALFRD

Dmel YNQILRIEEEIGAGVKFAGK SFRKPQ---MAT-------Q KEDHTALDLIIKSLKSPTMV --CEGTHFDGKIPHTFVIFG ASGDLAKKKIYPTLWWLYRD

Tcas YNQILRIEEELGANAKYAGK AFRKPQ---MPLLKIDDDDA ANSEVCLALYRKSLKSKEMD --HGGTHFDGHHPHVFITLG ASGDLARKKIYPTLWWLYRD

Pmon YNQILRIEEELGGNAKFAGK KFRKPC-------------- -------------------- -------------------- --------------------

Smar -------------------- -------------------- -------------------- -------------------- --------------------

Tcal YNQLLRIEEELGANAKFVGE KFRNPDA--MDPLAVH---D DCNQKGLELLIKSLKKIQV- ----EYQSGQGCTHIFTIFG ASGDLAKKKIYPTMWALYRD

Crog YNQLLRIEEELGSKAKYVGD KFRKPY---M---------- -ANNEGLELMRKSLRKITLS --SPQNEDPSKKCHIFVVMG ASGDLAKKKIYPTLWSLYFH

Dpul YNQILRIEEELGAAAKYAGK NFRHPL---MS--------- -------------------- ---QAEVFEETLPHSFVIFG ASGDLARKKIYPTIWWLYRD

Aver NLLPTNTRFVGYARSKITVQ AIRESCAPWCQVRENEEEKA DQFWALNSYVAGSYDQKSDF ENLDLVISEGESSSSDANRL YYLALPPSVFIPVTTHLKEA

Csin -------------------- -------------------- -------------------- -------------------- --------------------

Isca GLLPPKTKFVGYARSNMTVE SLSEKIEPYLKVKEEEKEKF SNFLKLNTYISGKYDASEDF DNLDGELRKLEGSS--ANRI FYMALPPTVFQAVATNIKRH

Eaff NLLPKGTQIFGYARSKMTVE ELRGKCAATVKAKDGEEAEL EGFWKANHYVAGSYDTKRDF ELLAQEMSAVEKGQ--NNRL FYLALPPSVFKPVTSMLKEA

Lcyp NLLPSNTKFVGYARSKIDVQ TLRANSDPWCNVKPGEEAKA DEFWSRNFYVAGSYDQKRDF ELLNQEISNLEKSNSVGNRL FYLALPPSVFESVTTFIKEA

Lsal -------------------- -------------------- -------------------- -------------------- --------------------

Aamp -------------------- -------------------- -------------------- -------------------- --------------------

Afra NLLPSNTRFVGYARSSLSVA EVRDKCQKYMKVKPEEEQRY DEFWTRNSYFHGNYDSRHDF EILDQEIRRLAACRE-ANRI FYLALPPSVFEVVTSSIQTA

Hazt -------------------- -------------------- -------------------- -------------------- --------------------

Meda NLLPSNTRFVGYARSKITVE SIRENCAPWCQVQEGEEEKA DKFWSLNSYVAGSYDNQADF ENLDAAITELESGSGIGNRL FYLALPPSVFIPVTSHLKVA

Afos NLLPKDTKIFGYARSKFTVQ QIREKCVGTVKAKPGEEEAL DKFWQSNYYVAGSYDTKRDF EMLAQEMGGVEKGR--NNRL FYLALPPSVFKPVTTMIKET

Dmel DLLPKPTKFCGYARSMLTVD SIKEQCLPYMKVQPHEQKKY EEFWALNEYVSGRYDGRTGF ELLNQQLEIMENKNK-ANRI FYLALPPSVFEEVTVNIKQI

Tcas NLLPVNTVFFGYARSKTSVQ EIKEKCKPYMKVRHGEEERF EDFWKLHYYVAGSYDSRTDF ERLNQELCPFEKGPA-ANRV FYLALPPSVFETVTVHIRNS

Pmon -------------------- -------------------- -------------------- -------------------- --------------------

Smar -------------------- -------------------- -------------------- -------------------- --------------------

Tcal SLIPENTQVFGYARSDLKVE DIRKKCEPYIKLKSGEEETF EKFWAANQYVRGTYDKRTDF EHLNHALTRAEDNKRENNRI FYLALPSSVFMESTKHIRET

Crog NLCPPNTKFIGYSRSKIDVK TIRERSAPWFSCPEEHKAKL EEFWSLNHYVAGSYDQKRDF ELLNQEMERLESGSSGRNRL FYLALPPSVFASVTVFLKEC

Dpul NLLPKNTIFYGYARSHMKVE EVRAKSHQYMKVKEEEQERY EAFWEANRYFAGGYDSRRDF EMLDQEMIQHERGPA-ANRL FYLAVPPFVFESVTANIRSS

Aver AMAKTG-WTRVIVEKPFGKD SESSATLSNHLSSLFKEXXL YRIDHYLGKEMVQNLMTI-- --------------RFGNRL FGPTWNRDNIASVVITFKEP

Csin -------------------- -------------------- ---D---------------- --------------WFGNMI FGPTWNRDSIASVTITFKEP

Isca CMSKKG-WTRVVIEKPFGKD SASSAELSNHLASLFDESQL YRIDHYLGKEMVQNLMAI-- --------------RFSNQI FGPTWNRNSIASVVISFKEP

Eaff CMSTKG-WTRVIVEKPFGKD SASSADLSNHLMKLFAEDQL YRIDHYLGKEMVQNLISL-- --------------RFGNMI FAPTWNRDNIASVVITFKEP

Lcyp VMAERG-WSRVIVEKPFGKD SESSAKLSNHLSSLFKEDQL YRIDHYLGKEMVQNLMTI-- --------------RFGNRI FGPTWNRDNIASVIITFKEP

Lsal -------------------- -----------XSLFREDQL YRIDHYLGKEMVQNLITL-- --------------RFANRI FSPTWNRDNIASVVITFKEP

Aamp -------------------- -------------------- -------------------- -------------------- --------------------

Afra CMADRG-WTRIIIEKPFGRD LESSAKLSNHLGSLFKEEQI YRIDHYLGKEMVQNLMIL-- --------------RFANRI FGPTWNRESISSVIISFKEP

Hazt -------------------- -------------------- -------------------- -------------------- --------------------

Meda TMAKTG-WTRVIVEKPFGKD SASSAKLSNHLSSLFREEQL YRIDHYLGKEMVQNLMTI-- --------------RFGNRL FGPTWNRDNIASVVITFKEP

Afos CMSDKG-WSRIIVEKPFGKD SESSAELSNHLQKLFTEEQL YRIDHYLGKEMVQNLISL-- --------------RFGNMI FGPTWNRDSIASVIISFKEP

Dmel CMSVCG-WNRVIIEKPFGRD DASSQALSDHLAGLFQEDQL YRIDHYLGKEMVQNLMTI-- --------------RFGNKI LSSTWNRENIASVLITFKEP

Tcas CMAPKG-WTRIIIEKPFGRD FDSSQKLSNHLGSLFTEQQI YRIDHYLGKEMVQNLMTL-- --------------RFGNRI FNPTWNRDNIASIQISFKEP

Pmon -------------------- -------------------- -------------------- -------------------- --------------------

Smar -------------------- -------------------- -------------------- -------------------- --------------------

Tcal CMAERG-WTRVIIEKPFGYD ADSSEKLSKHLSGLFTEEQL YRIDHYLGKEMVQNLMTL-- --------------RFANNL YTRSWDRDSIASVTITFKEP

Crog CMSSKG-WSRVIVEKPFGKD SASSAELSNHLSSLFTEDQL YRIDHYLGKEMVQNLITL-- --------------RFANRI FSPTWNRDNIASVVITFKEP

Dpul CMASKG-WTRVIVEKPFGRD LESSAHLSAHLAALFREDQI YRIDHYLGKEMVQNLMIL-- --------------RFGNRL FGPTWNRESIASVLITFKEP

Aver FGTQGRGGYFDEFGVIR--- -------------------- -------------------- -------------------- --------------------

Csin FGTQGRGGYFDEFGIIRDVM QNHLLQILCLAAMEKPVTTS PDDIRNEKVKVLKAIPSLSL SSCVLGQYSGDPDSEDPDAR LGYLEDQTVPEGSSCPTFAA

Isca FGTQGRGGYFDSFGIIRDVM QNHLLQIMCLVAMEKPVSTN AEDIRNEKVKVLKCVPPITM NHVVLGQYVGKPDGTGE-ER LGYLDDPTVPAGSRTATYAT

Eaff FGTQGRGGYFDEFGIIRDVM QNHLLQILCLTAMEKPVTTS PDDIRDEKVKVLKAVEVLSL DDVVLGQYQGNPEGADEDAR LGYLDDPTVPAGSTCPTFAA

Lcyp FGTQGRGGYFDEFGIIRDIM QNHLLQILCLTAMEKPPSTC PDDIRNEKVKVLKSCKEIDL KDVVLGQYVGNPEGADEDSR MGYLDDPTVPAGSVTPTFAA

Lsal FGTQGRGGYFDDFGIIRDIM QNHLLQIFCLTAMEKPCSTS PDDIRDEKVKVLKCCQEVVM DDVVLGQYVGNPEGKTEDAR MGYLEDKTVPDDSVTPTYAA

Aamp -------------------- -------------------- -------------------- -------------------- --------------------

Afra FGTQGRGGYFDEFGIIRDVM QNHLLQILCLTAMEKPASTS PNDIRDEKVKVLKSMPELKI EDVVLGQYVGDPNGEGE-AK EGYLDDRTVPKGSVTPTYAE

Hazt -------------------- -------------------- -------------------- -------------------- --------------------

Meda FGTQGRGGYFDEFGIIR--- -------------------- -------------------- -------------------- --------------------

Afos FGTQGRGGYFDEFGIIRDVM QNHLLQILCLTAMEKPVTTS PDHIRDEKVKVLKAVEELTM DDVVLGQYQGDPDSNDEDAR LGYLDDKTVPAGSTCPTYAA

Dmel FGTQGRGGYFDEFGIIRDVM QNHLLQILSLVAMEKPVSCH PDDIRDEKVKVLKSIEALTL DDMVLGQYLGNPQGTNDDAR TGYVEDPTVSNDSNTPTYAL

Tcas FGTQGRGGYFDEFGIIRDIM QNHLLQILTLAAMEKPASVH PDDIRNEKVKVLRCIKVIEK KDVVLGQYVGDPNGEGE-AK LGYLDDPTVPNDSVTPTYAL

Pmon -------------------- -------------------- -------------------- -------------------- --------------------

Smar -------------------- -------------------- -------------------- -------------------- --------------------

Tcal FGTQGRGGYFDQSGIIRDIL QNHLLQILCLAAMEKPASTS PDDIRDEKVKVLKTIPAITI EDCVLGQYVGDPNGKDEDSK LGYKDDDTVPKDSVTPTYAA

Crog FGTQGRGGYFDEFGIIRDIM QNHLLQIFCLTAMEKPCSTS PNDIRDEKVKVLKCCKEVDI QDVVLGQYVGDPEGKTEDAR LGYLDDKTVPEGSITPTYAC

Dpul FGTEGRGGYFDEFGIIRDVM QNHLLQMLCLTAMEKPASTH PNDIRDEKVKVLKCIPALTM DDVVLGQYIGDPEGQGE-AK ESYLDDPTVPKDSTTPTYAS

Aver -------------------- -------------------- -------------------- -------------------- --------------------

Csin AVLKINNERWDGVPFMLKCG KL------------------ -------------------- -------------------- --------------------

Isca AVAYINNERWEGVPFILRCG KALNERKAEVRIQYKEVPGD IFGGNSKRNELVLRVQPGEA IYVKLMSKKPGMAFDIEETE LDLTYGSRYKARATIRKPDF

Eaff AHLKIKNERWDGVPFILRCG KALNERKAEVRIQYKDVPGD IFNGSTVRNELVIRVQPNES VYVKVMTKTPGMSFNLEETE LDLTYKSRYK---EARLPE-

Lcyp AVIRINNERWDGVPFILKCG KALNERKAEVRIQYKEVAGD IFNGQAIRNELVLRVQPGEA IYAKVMTKTPGMSFTLEETE LDLTYNTRYK---KARLPD-

Lsal CVLRINNERWDGVPFILKCG KA------------------ -------------------- -------------------- --------------------

Aamp -------------------- -------------------- -------------------- -------------------- --------------------

Afra AVCWIDNERWDGVPFVLRCG KALNERKAEIRIQYRDVPGD IFGRQAKRNELVIRVQPGEA VYIKMMTKSPGMSFDIEETE LDLTYGTRYK---DVKMPD-

Hazt -------------------- -------------------- -------------------- -------------------- --------------------

Meda -------------------- -------------------- -------------------- -------------------- --------------------

Afos AALKIKNERWDGVPFILRCG KALNERKAEVRIQYRDVPGD IFNKQTVRNELVIRVQPNES VYAKVMTKTPGMSFTLEETE LDLTYNSRYK---DARLPE-

Dmel GVLKINNERWQGVPFILRCG KALNERKAEVRIQYQDVPGD IFEGNTKRNELVIRVQPGEA LYFKMMTKSPGITFDIEETE LDLTYEHRYK---DSYLPD-

Tcas AALHINNERWDGVPFILKCG KALNERKAEVRIQFKDVPGD IFDGKPKRNELVIRVQPGEA LYVKLMVKTPGMAFDMEETE LDLTYGHRYA---NVKLPD-

Pmon -------------------- -------------------- -------------------- -------------------- --------------------

Smar -------------------- -------------------- -------------------- -------------------- --------------------

Tcal LVLRINNERWEGVPFIMRAG KALNERKAELRIQYRDVPGN IFSKTPSRNELVLRVQPNEA IYCKMMNKSPGMSFTMEESE LDLTYRNRYA---DAKLPE-

Crog CVLRINNERWDGVPFILKCG KALNERKAEVRIQYKDVPGD IFNNSSVRNEMVVRVQPGEA VYSKVMTKTPGMNFNLEETE LDLTYKSRYK---DARLPE-

Dpul AALRINNERWDGVPFILRCG KALNERKAEVRIQYRDVPGD IFQGQAKRNELVIRVQPGEA IYVKMVTKTPGMSFDMEETE LDLTYGARYK---HVKMPD-

Aver -------------------- -------------------- -------------------- -------------------- --------------------

Csin -------------------- -------------------- -------------------- -------------------- -----MAGKIGINGFGRIGR

Isca RPERRLILDVFYGSQVHFVR SDELAEAWRIFTPLLHQIDR ERPQPVPYEYGSRGFKEADD LWKQVGFKFYGTYRWTNTAA -----M-AKVGINGFGRIGR

Eaff -AYERLILDVFVGSQMHFVR SDELAEAWRIFTPLLHKIEE EKPAPILYKYGTRGPKEADE MATKANFLYTGRYKWSAPSP --SKMM-SKIGINGFGRIGR

Lcyp -AYERLILDVFVGSQMHFVR TDELAEAWRIFTPLLKKIEG ERIQPIAYKYGSRGPDEANQ LAVANNFIYTGGYRWHGHQ- ---TDM-AKIGINGFGRIGR

Lsal -------------------- -------------------- -------------------- -------------------- -----M-VKVGINGFGRIGR

Aamp -------------------- -------------------- -------------------- -------------------- -----M-SKIGINGFGRIGR

Afra -AYERLILDVFCGSQMHFVR TDELAEAWRIFTPLLHKIEK EKIKPIPYVFGSRGPKEADE LLAKVDFKYYGSYKWEQPN- -----M-AKVGINGFGRIGR

Hazt -------------------- -------------------- -------------------- -------------------- -----M-SKIGINGFGRIGR

Meda -------------------- -------------------- -------------------- -------------------- --------------------

Afos -AYERLILDAFVGSQMHFVR SDELAEAWRIFTPLLHKIDA EKPKPIPYKYGSRGPKEADA LFAANDFKYTGRYKWQEPA- ---KLM-SKIGINGFGRIGR

Dmel -AYERLILDVFCGSQMHFVR SDELREAWRIFTPILHQIEK EHIRPITYQYGSRGPKEADR KCEENNFKYSGSYKWHGGKA ATSNHM-SKIGINGFGRIGR

Tcas -AYERLILDVFCGSQMHFVR SDELSEAWRIFTPLLHQIES ERVKPIPYVYGSRGPKEADE MLLNNNFTYTGSYKWPAPKM -----M-AKIGINGFGRIGR

Pmon -------------------- -------------------- -------------------- -------------------- -----M-SKIGINGFGRIGR

Smar -------------------- -------------------- -------------------- -------------------- -----M-SKIGINGFGRIGR

Tcal -AYERLILDVFVGSQMHFVR TDELREAWRIFTPILHKIEG EKVQPIPYKYGTRGPKEADE LAAKNNFVYTGTYSWNSDL- -----M-SKIGINGFGRIGR

Crog -AYERLILDVFLGSQMHFVR TDELAEAWRIFTPLLHKIEG EKIRPIPYKYGGRGPKEADE LTEQNNFKYTGRYIWKKD-- -----M-VKVGINGFGRIGR

Dpul -AYERLILDVFCGSQMHFVR SDELAEAWRIFTPLLHQIEG EKVKPLPYKYGSRGPREADD LLARSNFKYSGSYKWVKPS- ---SIM-SKIGINGFGRIGR

Aver -------------------- -------------------- -------------------- -------------------- --------------------

Csin LVLRAAVLKGATVVAINDPF IPLDYMVYMFKYDSTHGQFK GEVKEDGKFLYVNGNKITVF NERDPANIDWASAGAEYVVE STGVFTTTDKALAHTKGGAK

Isca LVLRAAVQKGVEVVAINDPF IDVKYMVYMFKYDSTHGRFN GDVHEEGGMLVVNGRKIHVF QELKPASIPWGKVGAEYVVE STGVFTTLEKAQQHIDGGAK

Eaff LVLRAAVEKGATVVAINDPF IPLDYMVYMFKYDSTHGQFK GEVKEDGTFLYVNGNKITVF NERDPVNINWASAGAEYVVE STGVFTTIDKALSHTKGGAK

Lcyp LVLRAALDKGVDVVAVNDPF IPLDYMVYMFKYDSTHGKYS GDVSLNGDKLVINGKSICVF NERDPSAIPWESAGAEYVVE STGVFTTKEKAGAHFKGGAK

Lsal LVLRAALDHGVDVVAVNDPF IPVDYMAYMFKYDSTHGMCK KDISYSDGKLIIDGKKITVF GERDPANIKWGEAGADYVVE STGVFTTIEKANAHIQGGAK

Aamp LVVRAALEKGAEVVAVNDPF INLEYMVYMFKYDSTHGRYK GEVKAEDGCLVIDGKKITVF NEMKPNNIPWSKAGAEYIVE STGVFTTIEKASAHFDGGAK

Afra LVLRAALEKGVDVVAVNDPF INLDYMVYMFKYDSTHGKHK GEIAAVDGKLVINGHAISVF QERDPKAIPWGSVGVEYVVE STGVFTTIEKANAHLEGGAK

Hazt LVLRCAIEKGAE-------- -------------------- ----ADGGNLVVNGHVIKVF NEMKPDNIPWGSAGAEYIVE STGVFTTLDKAKAHFSNGGK

Meda -------------------- -------------------- -------------------- -------------------- --------------------

Afos LVLRAAIEKGAMVVAINDPF IPLDYMVYMFKYDSTHGQFK GEVKEDGKFLYVNGNKITVF NERDPANINWASAGAEYIVE STGVFTTTDKALAHTKGGAK

Dmel LVLRAAIDKGASVVAVNDPF IDVNYMVYLFKFDSTHGRFK GTVAAEGGFLVVNGQKITVF SERDPANINWASAGAEYVVE STGVFTTIDKASTHLKGGAK

Tcas LVLRASLERGANVVAVNDPF IGLDYMVYLFKYDSTHGRFK GEVKAEGGFLVVNGNKISVF CERDPKSIPWGKVGADYVVE STGVFTTIEKASAHIEGGAK

Pmon LVLRAALQKGAEVVAVNDPF IALDYMVYMFKYDSTHGQHK GEVKAEDGCLVVDGHKITVF NEMKPENIPWSKAGAEYIVE STGVFTTIEKASAHFQGGAK

Smar LVLRACIDKGCEVVAINDPF IDINYMVYMFKYDSTHGRFK GDVHEEGGMLVVNGHKIHVF MEMKPNNIPWSKAGAEYVVE STGVFTTIEKAQAHFDGGAK

Tcal LVLRSAVAKGAAVVAVNDPF IPLDYMVYMFKYDSTHGRFK GEVKHDGTFLYINDQKITVF GERDPANIDWASAGAEYIVE STGAFTTLEKAGVHLTKGAK

Crog LVLRAALDNDVDVVAVNDPF IPVDYMAYMFKYDSTHGMCK KDISTSGDKLIIDGKEIVVF GERDPANIKWGASGVDYVVE STGVFTTIEKANAHIKGGAK

Dpul LVLRACIEKGATVVAVNDPF ITLDYMVYMFKYDSTHGRFK GEVKEEGGKLVVNGHHIQVF SERDPKNIPWGAAGAEYVVE STGVFTTLDKAQAHIDGGAK

Aver -------------------- -------------------- -------------------- -------------------- --------------------

Csin KVIISAPSADAPMFVMGVNH EKYDKSLQVVSNASCTTNCL APMAKVINDNFGIIEGLMTT VHAVTATQKTVDGPSGKDWR GGRGAAQNIIPSSTGAAKAV

Isca KVIISAPSADAPMFVMGVNH NSYNPSMTIVSNASCTTNCL APLAKVINDNFGIVEGLMST VHATTATQKTVDGPSAKMWR DGRGAAQNIIPASTGAAKAV

Eaff KVVISAPSADAPMFVMGVNH EKYDPSLQIVSNASCTTNCL APLAKVINDNYGILEGLMTT VHATTATQKTVDGPSGKDWR GGRGAAQNIIPSSTGAAKAV

Lcyp KVIISAPSADAPMFVMGVNE DKYQPNMHVVSNASCTTNCL APLAKVIHDHFGIIEGLMTT VHATTATQKTVDGPSNKAWR DGRGAAQNIIPAATGAAKAV

Lsal RVIISAPSADAPMFVMGVNH ESYDPSMKVISNASCTTNCL APLAKVIHDNFGIEQGLMTT VHAVTATQKTVDGPSAKNWR DGRGAGQNIIPASTGAAKAV

Aamp KVVISAPSADAPMFVCGVNL DAYSPDMKVVSNASCTTNCL APVAKVIHDNFTIVEGLMTT VHATTATQKTVDGPSGKNWR DGRGAGQNIIPASTGAAKAV

Afra HVIISAPSADAPMFVVGVNH EKYDPTMKIVSNASCTTNCL APLAKVINDKFGILEGLMTT VHAITATQKTVDGPSGKLWR DGRGAAQNIIPASTGAAKAV

Hazt KVIISAPSADAPMFVMGVNH EKYSKDMTIVSNASCTTNCL APVAKVLNDSFGIEEGLMTT VHAVTATQKTVDGPSAKDWR GGRGAGQNIIPSSTGAAKAV

Meda -------------------- -------------------- -------------------- -------------------- --------------------

Afos KVIISAPSADAPMFVMGVNH EKYDKSLQVVSNASCTTNCL APLAKVINDNYGIIEGLMTT VHAVTATQKTVDGPSGKLWR DGRGAAQNIIPASTGAAKAV

Dmel KVIISAPSADAPMFVCGVNL DAYSPDMKVVSNASCTTNCL APLAKVINDNFEIVEGLMTT VHATTATQKTVDGPSGKLWR DGRGAAQNIIPAATGAAKAV

Tcas KVIISAPSADAPMYVCGVNL DAYDPSAKVISNASCTTNCL APLAKVIHDNFEIVEGLMTT VHATTATQKTVDGPSGKLWR DGRGAGQNIIPAATGAAKAV

Pmon KVIISAPSADAPMFVCGVNM EKYTKDMKVVSNASCTTNCL APVAKVLHENFEILEGLMTT VHAVTATQKTVDGPSAKDWR GGRGAAQNIIPSSTGAAKAV

Smar KIIISAPSADAPMFVMGVNH ESYDPSMTVVSNASCTTNCL APLAKVVHDNFEIVEALMTT VHAVTATQKTVDGPSGKLWR DGRGAHQNIIPASTGAAKAV

Tcal KVVISAPSADAPMYVMGVNH EQYESSQHVVSNASCTTNCL APLAKVINDNFGIVEGLMTT VHAVTATQKTVDGPSNKDWR GGRGAGQNIIPSSTGAAKAV

Crog RVVISAPSADAPMFVMGVNH EKYDTSMTVISNASCTTNCL APLAKVIHDNFGIEQGLMTT VHAVTATQKTVDGPSAKNWR DGRGAGQNIIPASTGAAKAV

Dpul KVIISAPSADAPMFVVGVNL EAYDPSMKIVSNASCTTNCL APLAKVINDNFGIIEGLMTT VHAITATQKTVDGPSGKLWR DGRGAAQNIIPASTGAAKAV

Aver -------------------- -------------------- -------------------- -------------------- --------------------

Csin GKVIPELNGKLTGMAFRVPT PDVSVVDLTVRIEKEASYEE ICAKLKEASEG-SMKGILGY TDEDLVSSDFIGDDRSSIFD AKAGIQLSKTFVKLVSWYDN

Isca GKVIPELNGKLTGMAFRVPT PDVSVVDLTCRLAKEASYDE IKAAVKAAAESDNWKGILGY TEDEVVSSDFIGDTHSSIFD AKAGISLNKNFVKLISWYDN

Eaff GKVIPELNGKLTGMAFRVPT PDVSVVDLTVRLEKGASYED ICAKLKEAAEG-PMKGILGY TEDQVVSTDFLGDARSSIFD AKAGIQLSPNFVKLVSWYDN

Lcyp GKVIPELNGKLTGMAFRVPT PDVSVVDLTVRLAKETTYEE ICAKIKEVSEG-ELKDILGY TEDEVVSTDFLGDARSSIFD AKAGIQLSSTFVKLVSWYDN

Lsal GKVIPDLNGKLTGMAFRVPT PDVSVVDLTVILKKGATYEE ICAKIKEYSEG-PMKGILGY TEDAVVSCDFTSDPRSSIFD AKAGIQLSNNFVKLVSWYDN

Aamp GKVIPDLNGKLTGMAFRVPT PDVSVVDLTCRLGQEATMDD IKAKIRAAAEG-PLKGILGY TEDEVVSQDFLGDSHSSIFD AKACIQLSGTFVKLISWYDN

Afra GKVIPELNGKLTGMAFRVPV HDVSVVDLTCRLAKEASYED IKKAVKEASEG-AMKGILGY TEEEVVSTDFIGDNHSSIFD AKAGIQLSPTFVKLISWYDN

Hazt GKVIPELNGKLTGMAFRVPT PDVSVVDLTVRLGKECSYDE IKAAMKAASEG-PLKGYLGY TEDDVVSSDFIGDHRSSIFD AKAGIQLSKTFVKVVSWYDN

Meda -------------------- -------------------- -------------------- -------------------- --------------------

Afos GKVIPELNGKLTGMAFRVPT PDVSVVDLTVRLNKEASYEQ ICATVKAAAEG-PMKGILGF TDEQLVSTDFLGDNRSSIFD AKAGIQLSPTFVKLVSWYDN

Dmel GKVIPALNGKLTGMAFRVPT PNVSVVDLTVRLGKGATYDE IKAKVEEASKG-PLKGILGY TDEEVVSTDFFSDTHSSVFD AKAGISLNDKFVKLISWYDN

Tcas GKVIPALNGKLTGMAFRVPV PNVSVVDLTVRLGKPATYDD IKAKIKAAAEG-PLKGILGY TEEEVVSCDFVGDTHSSIFD AKAGIQLSPTFVKLISWYDN

Pmon GKVIPELNGKLTGMAFRVPT PDVSVVDLTVRLGKECSYDD IKAAMKAAAEG-PLKGVLGY TEDDVVSTDFVGDLRSSIFD AKAGIQLSKTFVKVVSWYDN

Smar GKVIPDLNGKLTGMAFRVPT PDVSVVDLTARIKKEATYDE IKAAMKSAAESDHWKGILGY TEDQVVSSDFIGDTHSSIFD ALAGISLNKTFVKLICWYDN

Tcal GKVIPELNGKLTGMAFRVPT PDVSVVDLTVRLAKEATYAE ICAAVKAAADG-PLKGYLGY SDEDVVSSDFIGDSHSSIFD SKAGIQLSPTFVKLISWYDN

Crog GKVIPDLNGKLTGMAFRVPT PDVSVVDLTVILKKGASYEE ICAKIKEVSEG-PMKGVLGY TEDQVVSCDFTSDPRSSIFD AKAGIQLSNNFVKLVSWYDN

Dpul GKVIPELNGKLTGMAFRVPV HDVSVVDLTCRLSKEASYDE IKAVVKAAAEG-PMKGILGY CDEEVVSTDFIGDNHSSIFD AAAGIALNKTFVKLISWYDN

Aver -------------------- -------------------- -------------------- -------------------- --------------------

Csin EMGYSNRVIDLITYMQSQD- -MSSNGDRRQSLAGK----- --AAKRF--------YRVDS QSDLSRYWDYGAYFSDNNRW CFECAWEVANKVGGIYTVIR

Isca EFGYSCRVVDLIKHMRSKDQ -----------MAEN----- --CLARI------------- -----PCPAGGIYTVVRSKA VLD-----SVIVGGIYTVIR

Eaff EFGYSNRVIDLIKHMQAKDA -M-ENLGRKMSLGGK----- --AAKRF--------YRVDS QGDLSKYYDHGSYFSDNNRW CFETAWEVANKVGGIYTVIR

Lcyp EYGYSNRVVDLINHMYSKDH -------------------- --MSKRF--------YRTDS QWDLSRYWDFGEHFAHQNRW CFECAWEVANKVGGIYTVIR

Lsal EYGYSNRVVDLVKYIATQE- -----------MERKGSVGS FRTSKRF--------YRSES IADLSRHLDYGEFFATNNRW CFECAWEVANKVGGIYTVIR

Aamp EWGYSNRVVDLIKFMQTKDK -------------------- -------------------- -------------------- --------------------

Afra EFGYSCRVVDLIKYMHSRA- -----------MATN---SA TPINRRF--------YKVDS TNDLMGYLDRGESAATSNIW TFECAWEVANKVGGIYTVIR

Hazt EYGYSNRVVDLIKHMQNVDA -------------------- -------------------- -------------------- --------------------

Meda -------------------- -------------------- -------------------- -------------------- --------------------

Afos EMGYSNRVIDLITYMQTKD- -----------MEQGI---- --QNDQFSRYRETSGYRMES GEDLVKFKEHQTYSFPENAW CFEASWEVANKQGGIYTVLR

Dmel EFGYSNRVIDLIKYMQSKD- -------------------- --MNRRF--------SRVES GADLKDYFDRGDIASRENRW NFEVAWEVANKVGGIYTVIR

Tcas EYGYSSRVVDLIKYISSKDS A----------MSRE----- KASSRRF--------YRSES YFNLMACMDRGTQAEEENRW TFEIAWEAANKVGGIYTVIR

Pmon EFGYSNRVIDLLKHMQTVDA -------------------- -------------------- -------------------- --------------------

Smar EYGYSHRVVDLIKYMQARDS K------------------- --MSRRF--------YRTDG QPDTV--LDHGVSAAMENRW LFEVSWEVANKVGGIYTVIR

Tcal EMGYSNRVIDLIQYMQSK-- -----------MTRQ----- --SSKRF--------YRSDS QGDLAKFWDHGEHFAMQNRW VFECAWEVANKVGGIYTVIR

Crog EYGYSNRVVDLIKFIATKE- -----------MERKGSSGN FRTSKRF--------YRSES IADLSRHLDYGESYANSNRW CFECAWEVANKVGGIYTVIR

Dpul EFGYSCRVVDLNKNRQPKD- -----------MSVK----- --AARRF--------YRVDS AHDL-QFLHSCESASAENRW TFEIAWEVANKVGGIYTVIR

Aver -------------------- -------------------- -------------------- -------------------- --------------------

Csin SKTGVSVNELGDQYILLGPY KEFHARQEVEEEEFSYAHPL GQAVQSQREKGYKIVCGRWL VEGNPQVILFDVGSAAYKMG EFKQEFYDTAGIGAPHGDME

Isca SKAEVTVEELGDQFCLIGPY NENLVRTEVEVLE-PPYSIF WNSVKALKEQGIKVVYGRWL IDGYPQVLLFDIGSAAWKLD EWKRDLWNTCNIGVPWHDRE

Eaff SKTGVSVQELGDQYVLLGPY KEQHARQEVEEEDFSPAHPL GKSVEALRQRGYRVVCGRWL VEGNPQVILFDIGSAAYKLD EFKQDLYEKAGIGVPHGDVE

Lcyp SKTGVSVNELGDQYILMGPY VEHKAKQEIEEEDFPFHHPL GQAVERARQKGYRIHCGRWL VDGNPQVILFDIGSAAHKMN EFKQQLFEKAGIGIPHEDIE

Lsal SKTGVTVSELGDQYALLGPY IEHKAKQEIEEQEFELHHPL GQAVEQMRERGYVLHTGRWL VDGNPQVILFDIGSALGKF- -----LYFNTVIFIHY-NYN

Aamp -------------------- -------------------- -------------------- -------------------- --------------------

Afra SKASISVEELGNQYILLGPY KEQFARTEVEDFEFP-AGPL NNAVSKLRDSGFRIHTGSWL VDGNPLVILFDIGSASYNMD KYKHELFEKSGVGIPHLDIE

Hazt -------------------- -------------------- -------------------- -------------------- --------------------

Meda -------------------- -------------------- -------------------- -------------------- --------------------

Afos SKAQVSTREMGNRYVMLGPL KSAHVH-EVEEFEASECSPL GRAVSAMKKRGFNVLLGNWL IEGNPTVVLFDIGSASHCMN SWKQELYENTHIGVPHSDIE

Dmel SKAYVSTEEMGEQLCMMGPY KEHCARTEMEEMEFPRGNPL LDAVNSLRSRGYKIHTGRWL VDGNPQLILFDIGSAAWKLD QFKSEMWEKCHIGIPHLDIE

Tcas SKSYVSTEEMGDQYCLIGPY KEFHARTEVEEGPLG-SDPL NEAVQALRDKGFKVHTGRWL VDGNPQIILMDIGSAAWKLD EYKTELWEKCNVGVPHLDVE

Pmon -------------------- -------------------- -------------------- -------------------- --------------------

Smar SKAGITTEELGDQFTLMGPY NETLVQTEVELIE-PEADLF GPTINAIKEKGFKVHYGRWL IEGYPRVILFEIGSGAWKLD EWKRELWNVTNIGIPWHDRE

Tcal SKTGVSVNELGDQYVLLGPY FEFKAKQEVEEEDFPYHHPL GQAVERARQKGYRIHCGRWL VEGNPQIILFDVGSAGAKMN EFKHELYEKAGIGIPHEDVE

Crog SKAGITVSELGDQYALLGPY FEDKAKTEIEEQEFELHHPL GQAVEQMRQRGYNVHTGRWL VDGNPQVVLFDVGSAAYKLD EIKQDLYEKSSISVPYLDQE

Dpul SKASASVEELGDHYILLGPY KEHCAKQEVETFELPPGTPL AIAVQKLRNRGFKVVTGNWL VDGNPLLVLFDIGSASWNLD NYKKELFEKSGIGIPNHDAE

Aver -------------------- -------------------- -----------LIMARIWKI DLATVFTTHATXLGRFLCAG ATD-----FYNNLETFNCDE

Csin VNDVTVFGFMVAQFLADFRI RAENYS------DSLPPRIS AHFHEWMAGVGLIMTRLWKV DVATLFTTHATQLGRHLCAG DTD-----FYNNLEKFDCDH

Isca SNDAIIFGYLVAWFLSDVMT AE----------KG-PPFVV AQFHEWLAGIGLMLCRTRHL DVATVFITHATLLGRYLCAG NVD-----FYNNLDKFSLDK

Eaff VNDTVIFGFMVAQFLGEFRY QSEDFS------DS-PPRIA AHFHEWMAGVGLIMSRMWKL DIATVFTTHATQLGRHLCAG DTD-----FYNRLESFDCDA

Lcyp SNDVTIFGFMVAEFLGDFRY LSENYA------DA-APRIV THFHEWMAGVALIMVRIWKI EVATVFTTHATLLGRFLCAG ATD-----FYNNLERFNCDE

Lsal NDLIXFYRNMVAEFLGSFHY WSQGYA------DV-PPHIV AHFHEWMSGVGLIFSRLWKL EIATVFTTHATLLGRMLCAG STD-----FYNNLSKFDLDE

Aamp -------------------- -------------------- -------------------- -------------------- --------------------

Afra SNDAVIFGFMVAQFIADFRY FSNRGG------EV-APKIV CHFHEWLSGIGLIMTRLWNI EVSTVFTTHATLLGRYLCAG NLD-----FYNNLDKFNIDA

Hazt -------------------- -------------------- -------------------- -------------------- --------------------

Meda -------------------- -------------------- ------MAGVALIMARIWKI DMATVFTTHATLLGRFLCAG ATD-----FYNNLQNFNCDE

Afos CND----------------- -------------------- -------------------- ------RSHATQLGRHLCAG DTD-----FYNRLEQFDCDK

Dmel TNDAIILGFMIAEFLEEFRN FAVTYS--QNN-ELSAPRIV AHFHEWQAGVGLIVLRTRLV EIATVFTTHATLLGRYLCAG NTD-----FYNNLDKFAVDE

Tcas ANDAIILGNMVADFIANFKN CADRYG------DGIPARIV THFHEWQAGVGLITLRTRLV KVATVFTTHATLLGRYLCAG NTD-----FYNNLDKFVVDE

Pmon -------------------- -------------------- -------------------- -------------------- --------------------

Smar SNDSVIFGFLVTMFLTEFIN QARSHN------E--SPLVI AHFHEWQAGIGLLLCRVRHL DIATIFTTHATLLGRYLCAG NVD-----FYNNLDRFNIDK

Tcal ANDVCIFGLMVAEFLGDFRF LAENYS------DS-PPRIV GHFHEWMAGVGLIMSRIWKI DIATVFTTHATLLGRFLCAG ALD-----FYNKLDQFNCDE

Crog TNDITIFGHMVAEFLGSFHH WSTNYA------DA-PPRIV AHFHEWMAGVGLIFSRLWKL EIATVFTTHATLLGRMLCAG STD-----FYNNLSKFDLDK

Dpul SNDSVVFGFMAAQFIAEFNY AAQQEHLALANGSP-APRIV AHFHEWLAGIGLVLLRLWGV QVATVFTTHATLLGRYLCAG NID-----FYNNLDKFNIDE

Aver EAGKRGFYHRYCMERAAAHL CHTFTTVSDITGKEAEHLIK RKADIITPNGLNVVRDXXLH EFQNLHARSKEKINEFVRGH FYGHFDFDLDKTLYIFTAGR

Csin EAGKRGIYHRYCIERASAHL AHVFTTVSEITGQECEHLVK RKPDILTPNGLNVKR--DLH EFQNLHAKCKEKINEFVRGH FHGHFDFDLDKTLYLFTAGR

Isca EAGDRGIYHRYCMERAACHS AHVFATVSEVTAMEAEHLLK RKPDAITPNGLNVKKFSALH EFQNMHAIAKEKIHDFVRGH FYGHYDFDLDKTLYCFIAGR

Eaff EAGKRGIYHRYCLERGAAHL AHIFTTVSEITGIESEHLIK RKPDIITPNGLNVKR--DLH EFQNLHAKSKESINEFVRGH FYGHFDFDLDNTLYFFTAGR

Lcyp EAGKRGFYHRYCLERAATHL AHVFTTVSEITGRESEHLVK RKPDIITPNGLSVVR--DLH EFQNQHAKSKEKINQFVRGH FYGHFNFDLDKTLFFFSAGR

Lsal EAGKRGFYHR---------- -------------------- --PDIITPNGLNVTR--DLH EFQNLHAISKNKINDFVRGH FYGHFDFDLDKTLYLFSAGR

Aamp -------------------- -------------------- -------------------- -------------------- --------------------

Afra EAGKRQFYHRYCIERAATHL AHIFTTVSEITGIEAEHLLK RKPDIITPNGLNVKKFSALH EFQNLHSVSKEKIHDFIRGH FYGHHDFDLDKTLYFFIAGR

Hazt -------------------- -------------------- -------------------- -------------------- --------------------

Meda EAGKRGFYHRYCVERAAAHL XHTFTTVSDITGKEAEHLIK RKADIITPNGLNVIR--DLH EFQNLHAKSKEKINEFVRGH FYGHFDFDLDKTLYXFXAGR

Afos EAGKRQIYHRYCLERAAATL AHIFTTVSEITGIEAEHLIK RKPDIITPNGLNVKR--DLH EFQNLHAKSKESINEFVRGH FYGHFDFDLDNTLYFFSAGR

Dmel EAGKRQIYHRYCLERGATHL AHVFTTVSEITGYEAEHLLK RKPDIITPNGLNVKKFSAIH EFQNLHAVAKEKINEFVRGH FYGHIDFDLDKTLYFFIAGR

Tcas EAGKRQIYHRYCIERAATHL AHVFTTVSDITGFEAEHLLK RKPDVITPNGLNVKKFSALH EFQNLHAISKEKIHEFIRGH FYGHYDFDLDKTLYFFIAGR

Pmon -------------------- -------------------- -------------------- -------------------- --------------------

Smar EAGDRGIYHRYCMERASAHS AHCFTTVSEITALEAEHLLK RRADIITPNGLNVKKLSALH EFQNMHAIAKEKIHDFIRGH FYGNYDFSLDKTLYFFIAGR

Tcal EAGKRGFYHRYCIERAAAHL CHCFTTVSDITGKEAEHLIK RKPDMITPNGLNVQR--DLH EFQNLHAESKEKINQFVRGH FYGHFDFDLDKTLYFFIAGR

Crog EAGSRGFYHRYCLERSATAL ADCFTTVSDITSIEAEHLIK RKPDIVTPNGLNVNR--DLH EFQNLHAVSKNKINDFVRGH FNGHFDFDLDKTLYFFSAGR

Dpul EAGRRQIYHRYCMERAATNL AHTFTTVSEITGLEAEHLLK RKPDVITPNGLNVKKFSALH EFQNLHAIYKEKINEFVRGH FYGHLDFDVDRTLYFFIAGR

Aver YEFGNKGVDIFIESLARLN- --HKLKSESPDKTVVAFMIF PTATNSFNVESLRGHAVTKG LKDTIDEIQQEIGKRMYETC LRGHVPDSMDLMSKNDIVRL

Csin YEFTNKGADMFIESLARLN- --HLLKKAGSDVTVVAFLIF PTKTNSFNVESLRGHAITKG LKDTIDSIEKDIGKRLYEKC LRGDVPESGDLLTRDDLV--

Isca YEFSNKGADLFIESLARLN- --HYLKSSGSEVTVIAFLIF PAKTNNFNVESLRGQAISKQ LRDTVQQMQSTIGKRMFEIC LSGKIPKGEELIYSEDLVRL

Eaff YEFTNKGADIFIEALARLN- --HLLKQSNSEVTVVAFLIF PTPTNSFNVESLKGHAITKG LKDTIDAVQKDIGKRLYEKC LRGYIPDASDLLTRDDLTTL

Lcyp YEFGNKGVDIFIEALARLN- --HQLKEQKSDKTVIAFLIF PTKTNSFNVESLRGHAVAKG LKDTIDEIQRDIGKRMYEIC LKGHIPDADELLAKNDIVRL

Lsal YEFINKGVDIFIESLARLN- --FMLQQSGTDRTIVVFLIF PAMNKGFNVESLRGHAVTKA LKDTIDKVQKEIGERMFDIC SKGHLPESDDLLFKSDIVRL

Aamp -------------------- -------------------- -------------------- -------------------- --------------------

Afra YEFGNKGADVFIEGLARLN- --HMMKESGSDKTVVAFLIF PTRTNNYNVESLRGQAIAKS LRDTVHLVQQEIGSKMYEAC LRGRLPENDELLSQDDIVKL

Hazt -------------------- -------------------- -------------------- -------------------- --------------------

Meda YEFGNKGVDIFIESXARLN- --HRLKAENSDKTVVAFLIF PTATNSFNVESLRGHAVTKG LKDTIDEIQKEIGKRMYETC LRGHVPDAIDLLSKNDIVRL

Afos YEFTNKGADIFIESLARLN- --HLLKSSGSDITVVAFLIF PTKTNSFNVESLKGHAITKG LKDTIDSVQKDIGKRLYEQC LRGKIPQPDEILTRDDLTKL

Dmel YEFGNKGADIFIEALARLN- --AMLKHEKPDTTVVAFLIF PTKTNNFNVDSLRGHAVIKQ LRDTINNVQQAVGKRMFDTC LQGNIPNADDLLQKDDLVKI

Tcas YEFSNKGADIFIEALARLN- --HYLKSTHPDITVVAFLIF PTRTNNFNVESLRGHAVTKS LRDTISDIQTRIGRRMYEVC LSGHLP-GDDLLKKEELVRL

Pmon -------------------- -------------------- -------------------- -------------------- --------------------

Smar YEFSNKGADMFIEALARLN- --HYLKSAGSDMTVIAFLIF PARTNNFNVESLRGQAIAKQ LHETINEIQTKIGRRMFEIS LGGRLPTNAELLQSEDIVKL

Tcal YEFGNKGADIFIESLARLN- --HLLKEAKSDKTVVAFLIF PTKTNSFNVESLRGHAVTKG LKDTIDEIQKVVGNRLYETC LRGMVPDGNDLLSKNDIVKL

Crog YEFINKGVDIFIESLARLN- --FQLQASGSNRTVIVFLIF PAPNKGFNVESLRGHAITKT LKDAIEEVKREIGQRMFDSC SRGHVPESEDLLTREHLIKL

Dpul YEFGNKGADVFIEALARLN- --HMLKVSGSDKTIVAFLIF PAKTHNFNVETLRGQAVVKS LRDTIHDIQQKIGKRIYDVC LSGRLPDSEELLQKEDTVRL

Aver KRCIFAQGASILPPITTHNI VNDATDPIMCALRRCQLFNN RYDRVKVIFHPEFLASTNPL FGLDYQDFVRGC-------- --------------------

Csin -------------------- -------------------- -------------------- ------------------LY YEPWGYTPAECTIMGIPNVS

Isca KRCIYASQRTTLPPVCTHNM LDDSSDPVLCHIRRCQLFNR REDRVKVIFHPEFLSSTNPL FSMDYEEFVRGCHLGIFPSY YEPWGYTPAECTVMGIPSVT

Eaff KRSIFAAQSSPLPPITTHNV INEYNDPILNAFRRCQLFNH RSDRVKVVFHPEFLSTTNPL FGLDYQEFVRGCHMGVFPSY YEPWGYTPAECTIMGIPSIT

Lcyp KRCIFAQGANLLPPITTHNI VDDATDPIMNAIRRCHLFNN RYDRVKIIFHPEFLASTNPL FGLDYQEFVRGCHMGVFPSY YEPWGYTPAECTVMGIPSIT

Lsal KRCVYAA-------ITTHNI VNDADDSIINAIRRCKLFNN KSDRVKIIFHPEFLSSTNPL FGLDYNEFVRGCHLGVFPSY YEPWGYTPAECTIMGIPSIT

Aamp -------------------- -------------------- -------------------- -------------------- --------------------

Afra KRCLFASQRSSLPPITTHNV VDDHADPVLNNLRRCQLFNS RNDRVKVIFHPEFLSSTSPL LGLDYEDFVRGCHLGVFPSY YEPWGYTPAECTVMGIPSVT

Hazt -------------------- -------------------- -------------------- -------------------- --------------------

Meda KRCIFAQGASLLPPITTHNI VNDATDPIMCALRRCQLFNN RXDRVKVIFHPEFLASTNPL FGLDYQDFVRGCHMGVFPS- --------------------

Afos KRSIFAAQSSPLPPITTHNI CDEGKDPVLNAFRRCHLFNN SHDRVKVVFHPEFLNSTNPL FGLDYQDFVRGCHMGVFPSY YEPWGYTPAECTIMGIPSIT

Dmel KRCMFAMQRDSMPPVTTHNV ADDWNDPVLSSIRRCHLFNS RHDRVKMVFHPEFLTSTNPL FGIDYEEFVRGCHLGVFPSY YEPWGYTPAECTVMGIPSVT

Tcas KRCIFSLQRDGLPPVTTHNI VDDWNDPVLASVRRCNLFNT KYDRVKIVFHPEFLSSTNPL FGLDYEEFVRGCHLGVFPSY YEPWGYTPAECTVMGIPSIT

Pmon -------------------- -------------------- -------------------- -------------------- --------------------

Smar KRCIFAAHRNSLPPICTHNM MDDSGDPVLTAIRRTQLFNN RLDRVKLIFHPEFLSPTNPL LGLEYDEFVRGCHLGVFPSY YEPWGYTPAECTVMGIPNVS

Tcal KRCIFAQAHTNLPPITTHNI VDDAMDPVMNAFRRCQLFNN RTDRVKVVFHPEFLTSTNPL FNLDYQEFVRGCHMGVFPSY YEPWGYTPAECTIMGIPSIT

Crog KRCVYAAENAGPPPVTTHNI IRDSEDQILNAIRRCKLFNG RHDRVKIIYHPEFLSSTNPL FGLDYNEFVRGCHLGVFPSY YEPWGYTPAECTIMGIPSIT

Dpul KRCIYATQRDTLPPITTHNV VDDASDPVLNSFRRCKLFNE TSDRVKVIFHPEFLSSTNPL FGLDYEDFVRGCHLGVFPSY YEPWGYTPAECTVMGIPSIS

Aver -------------------- -------------------- -------------------- -------------------- --------------------

Csin TNLS---------------- --GFGCFMDEHVVDPQSYGI YIVDRKLQSVEESVNQLAGF MFDYTKLNRRQRIIQRNRTE RLADLLDWKNLGVYYRQARV

Isca TNLS---------------- --GFGCFIAEHVADPMSYGI YIVDRRFKSAEESVQQLAQY MFDFSCLSRRQRIIQRNRTE RLSDLLDWKNLGISSKL---

Eaff TNLS---------------- --GFGCFMEEHVTDPQSYGI YIVDRRKRGLEDSVQQLAGF MHDYTKLNRRQRIIQRNRTE RLSDLLDWKNLGIYYRQARM

Lcyp TNLS---------------- --GFGCFMQENILDPQSYGI YIVDRLNIGLEDSIKQLCKY MYGFTRLNRRQRIIQRNRTE RLSDLLDWKSLGIYYRQARM

Lsal TNLS---------------- --GFGCFMEEHVVDPQSYGI YVVDRRHKGLDESINDLSK- -------------------- --------------------

Aamp -------------------- -------------------- -------------------- -------------------- --------------------

Afra TNLS---------------- --GFGCFMNDHIADPTSYGI YIVDRRFISLDESISQLANY MHDFVRLNRRQRIIQRNRTE RLSDLLDWRTLAVYYRRARL

Hazt -------------------- -------------------- -------------------- -------------------- --------------------

Meda -------------------- -------------------- -------------------- -------------------- --------------------

Afos TNLS---------------- --GFGCFMEEHVTDPQSYGI YVVDRRNIGLEESVQQLARF MHDFTRLNRRQRIIQRNRTE RLSDLLDWKNLGIYYRQARM

Dmel TNLS---------------- --GFGCFMEEHISDPKSYGI YIVDRRYIGLENSVQQLSSF MMEFSRLNRRQRIIQRNRTE RLSDLLDWRTLGIYYRQARV

Tcas TNLS---------------- --GFGCFMQEHIADPMSYGI YVVDRRYISLEASVQQLAQY MYDFARLNRRQRIIQRNRTE RLSDMLDWRSLGVYYRQARM

Pmon -------------------- -------------------- -------------------- -------------------- --------------------

Smar TNLS---------------- --GFGCFMAEHVADPMSYGI YIIDRRYKSAEDSVQQLSQY LFDFTCLSRRQRVIQRNRTE RLSDLLDWKNLGIVIQN---

Tcal TNLS---------------- --GFGCFMQEQIVDPQSYGI YIVDRMHIGVEESVSQLAKC MMDFVGLNRRQRIIMRNRTE RLSDMLDWKSLGIYYRRARM

Crog TNLS---------------- --GFGCFMEEHVMDPKSYGI YVVDRRHKGLDESINDLASS MYEFSLMNRRQRIIMRNRTE RLSDLLDWKSLGIYYRQARM

Dpul TNLS---------------- --GFGCFMGDHIADPMSYGI YVVDRRFISLDDSLNQLAQY MFDFTRLNRRQRIIQRNRTE RLSDLLDWRNLSVYYRKARQ

Aver -------------------- -------------------- -------------------- -------------------- --------------------

Csin MALEKVYPDFVDTAPSL-SG VGRMNYPRPISEPPSPCEHQ GSLPS--------------- -------------------- WEMARTKQTARKSTGGKAPR

Isca -------------------- --QLRYPRPMSEPPSPSASR TSTPAPSEHGS--DEE-EDS DEGEELKH------------ -TMARTKQTARKSTGGKAPR

Eaff KALQTVYPDISEEEAPP-RG VGRLNYPRPISEPPSPISTR AHSPVSSLADE--DDC-DSH DSDEELEQLQVAL-SKI--- -AMARTKQTARKSTGGKAPR

Lcyp RALRCVFGDIDEDEPHP-RP LSSRLYPRPFSEPPSPSSSR ATTPPPGSDADGPDDDDASH DSEEEERELCLAIDTHL--- --MARTKQTARKSTGGKAPR

Lsal -------------------- -------------------- -------------------- -------------------- --MARTKQTARKSTGGKAPR

Aamp -------------------- -------------------- -------------------- -------------------- --MARTKQTARKSTGGKAPR

Afra LALHEVFPELAVEEE---RG VRQIKYPRPISEPPSPTSSR ATTPAPSDSDE-------EI DDEKELEELGHKV-AKVIS- -LMARTKQTARKSTGGKAPR

Hazt -------------------- -------------------- -------------------- -------------------- --MARTKQTARKSTGGKAPR

Meda -------------------- -------------------- -------------------- -------------------- --------------------

Afos KALQSVFPDVSSEEAPP-AY AGRLNYPRPISEPPSPTSTR AHSPISSLNDD--DA--DSH DSDEELEQLQVAL-NNLD-- -NMARTKQTARKSTGGKAPR

Dmel KALQAVYPDYVDELSLY-GS KNNLIFSRPHSEPPSPTSSR HTTPAPSVHGS--DDE-DSV DEETELKELGIK-------- --MARTKQTARKSTGGKAPR

Tcas KALHSVFPDYKEEMD---NG IATLKYPRPISEPPSPSSSR VTTPAASLHGS--DDESDSV DEERELEELKINH------- -HMARTKQTARKSTGGKAPR

Pmon -------------------- -------------------- -------------------- -------------------- --MARTKQTARKSTGGKAPR

Smar -------------------- -------------------- -------------------- -------------------- --MARTKQTARKSTGGKAPR

Tcal RALQNVFPDVSENEEAP-RL PSRLNYPRPISEPPSLAPSR ANSPNSSAPGS--DSE-ASH DSEEEEAELQTAL-SQLQA- -SMARTKQTARKSTGGKAPR

Crog IALKHTFPDINDEDE---TL PGVINFPKPISEPPSPSLSR ASSVPPPEDSE--DS---DH DSEEEERELVIQLHNNAI-- --MARTKQTARKSTGGKAPR

Dpul MALHAVFPEFDLDEDENGSG VGRLSYPRPISEPPSPTSSR ATTPIPSDQSD--ED--DEV DEERELRELSVGAEER---- -RMARTKQTARKSTGGKAPR

Aver -------------------- -------------------- -------------------- -------------------- --------------------

Csin KQLATKAARKSAPATGGVKK PHRYRPGTVALREIRRYQKS TELLIRKLPFQRLVREIAQD FKTDLRFQSSAVMALQEASE AYLVGLFEDTNLCAIHAKRV

Isca KQLATKAARKSAPATGGIKK PHRYRPGTVALREIRRYQKS TELLIRKLPFQRLVREIAQD FKTDLRFQSSAVMAMQEACE AYLVSLFEDCNLCAIHAKRV

Eaff KQLATKAARKSAPSTGGVKK PHRYRPGTVALREIRRYQKS TELLIRKLPFQRLVREIAQD FKTDLRFQSAAIGALQEASE AYLVGLFEDTNLCAIHAKRV

Lcyp KQLATKAARKSAPATGGVKK PHRYRPGTVALREIRRYQKS TELLIRKLPFQRLVREIAQD FKTDLRFQSSAVAALQEASE AYLVGLFEDTNLCAIHAKRV

Lsal KQLATKAARKSAPSTGGVKK PHRYRPGTVALREIRRYQKS TELLIRKPPFQRLVREIAQD FRTDLRFQSAAIGALQEASE AYLVGLFEDTNLCAIHAKRV

Aamp KQLATKAARKSAPSTGGVKK PHRYRPGTVALREIRRYQKS TELLIRKLPFQRLVREIAQD FKTDLRFQSAAIGALQEASE AYLVSLFEDTNLCAIHAKRV

Afra KQLATKAARKSAPSTGGVKK PHRYRPGTVALREIRRYQKS TELLIRKLPFQRLVREIAQD FKTDLRFQSAAIGALQEASE AYLVGLFEDTNLCAIHAKRV

Hazt KQLATKAARKSAPATGGVKK PHRYRPGTVALREIRRYQKS TELLIRKLPFQRLVREIAQD FKTDLRFQSSAVMALQEASE AYLVGLFEDTNLCAIHAKRV

Meda -------------------- -------------------- -------------------- -------------------- --------------------

Afos KQLATKAARKSAPSTGGVKK PHRYRPGTVALREIRRYQKS TELLIRKLPFQRLVREIAQD FKTDLRFQSAAIGALQEASE AYLVGLFEDTNLCAIHAKRV

Dmel KQLATKAARKSAPATGGVKK PHRYRPGTVALREIRRYQKS TELLIRKLPFQRLVREIAQD FKTDLRFQSSAVMALQEASE AYLVGLFEDTNLCAIHAKRI

Tcas KQLATKAARKSAPSTGGVKK PHRYRPGTVALREIRRYQKS TELLIRKLPFQRLVREIAQD FKTDLRFQSAAIGALQEASE AYLVGLFEDTNLCAIHAKRV

Pmon KQLATKAARKSAPSTGGVKK PHRYRPGTVALREIRRYQKS TELLIRKLPFQRLVREIAQD FKTDLRFQSAAIGALQEASE AYLVGLFEDTNLCAIHAKRV

Smar KQLATKAARKSAPATGGVKK PHRYRPGTVALREIRRYQKS TELLIRKLPFQRLVREIAQD FKTDLRFQSSAVMALQEASE AYLVGLFEDTNLCAIHAKRV

Tcal KQLATKAARKSAPATGGVKK PHRYRPGTVALREIRRYQKS TELLIRKLPFQRLVREIAQD FKTDLRFQSSAVMALQEASE AYLVGLFEDTNLCAIHAKRV

Crog KQLATKAARKSAPATGGVKK PHRYRPGTVALREIRRYQKS TELLIRKLPFQRLVREIAQD FKTDLRFQSSAVMALQEASE AYLVGLFEDTNLCAIHAKRV

Dpul KQLATKAARKSAPATGGVKK PHRYRPGTVALREIRRYQKS TELLIRKLPFQRLVREIAQD FKTDLRFQSSAVMALQEASE AYLVGLFEDTNLCAIHAKRV

Aver -------------------- -------------------- -------------------- -------------------- --------------------

Csin TIMPKDIQLARRIRGERALN ELKQELEIDVHKVSVEELCK RFKTQVERGLTDTQAKANFA EYGPNALTPPPQTPEWVKFC QQMFSGFACLLWLGAILCFL

Isca TIMPKDMQLARRIRGERSLD DLKQEVSMDEHKIPIEELYA RLGTNPATGLTSQQAREIFE RDGPNSLTPPKKTPEWVKFC KNLFGGFSLLLWIGAVLCFI

Eaff TIMPKDIQLARRIRGERALN DLKQELEIDVHKISQDEILK RFGTSLENGLTLDQVKKKQE EYGLNQLTPPPTTPEWIKFC KNLFSGFAMLLWLGAILCFV

Lcyp TIMPKDIQLARRIRGERALN ALKQELELDVHKVDLDTLLK RFNSDVNRGLRKSQAEENQS KYGPNTLTPPPTTPEWIKFC KNLFGGFALLLWVGAILCFT

Lsal TIMPKDIQSARRIRGERA-- -------------------- -------------------- -------------------- ----GGFAMLLWLGAILCFL

Aamp TIMPKDIQLARRIRGERAVD DLKQELVLDEHQITIEELQQ RLGTSITAGLTEARAKENLD RYGPNALTPPKKIPEWVKFC KNLFYGFSLLLWIGAILCFV

Afra TIMPKDIQLARRIRGERALN ELKKELDIDFHKIPIEECYQ RLGSNPETGLTNAQARSNIE RDGPNCLTPPKTTPEWIKFC KNLFGGFALLLWTGAILCFL

Hazt TIMPKDIQLARRIRGERA-- -------------------- -------------------- -------------------- --------------------

Meda -------------------- -------------------- -------------------- -------------------- --------------------

Afos TIMPKDIQLARRIRGERALN ELKKELEIDAHTVSLDELCK RHGTNIERGLTPEQAKEGLE KYGPNALTPPPKTPEWVKFC KNMFSGFALLLWIGAILCFV

Dmel TIMPKDIQLARRIRGERALD DLKQELDIDFHKISPEELYQ RFQTHPENGLSHAKAKENLE RDGPNALTPPKQTPEWVKFC KNLFGGFAMLLWIGAILCFV

Tcas TIMPKDIQLARRIRGERALD DLKQELDIDYHKITPEELYQ RFQTHPENGLSHAKAKENLE RDGPNALTPPKTTPEWVKFC KNLFGGFALLLWIGAILCFI

Pmon TIMPKDIQLARRIRGERA-- -------------------- -------------------- -------------------- --------------------

Smar TIMPKDIQLARRIRGERALD DLKQELSMDEHQLAIEDLYI RLQTNPTTGLTNEQAKENLA RDGPNALTPPKTTPEWIKFC KNLFGGFALLLWVGAILCFV

Tcal TIMPKDIQLARRIRGERALQ NLKQELELDVHRIDIEELCK RFNTNLNNGLTKRQAEENLG KYGRNALTPPPTTPEWIKFC NNLFGGFSLLLWFGAILCFI

Crog TIMPKDIQLARRIRGE--LT NLKQELELDVHKISFDELCK RYGSDLDKGLSKSRVEENQK KYGLNALTPPPTTPEWIKFC QNLFGGFALLLWLGAILCFI

Dpul TIMPKDIQLARRIRGERAMN ELKQELDIDDHKIPIEELYQ RLGTNPETGLTTAQAKAIFE RDGPNALTPPKTTPEWVKFC KNLFGGFSMLLWVGAILCFV

Aver -------------------- -------------------- -------------------- -------------------- --------------------

Csin AYGIQASA-YEEPPDDNLYL GVVLTAVVTVTGIFSYYQES KSAAIMESFKNLVPQFAKVI RNGKKIDIEAKNVTLGDIVD VQFGDRMPADVRILESKGFK

Isca AYSIQAGT-FEEPPDDNLYL GAVLAIVVIVTGCFSYYQEA RSSKIMESFKNMVPQYAIVI RDGQKLNLPAEEVVVGDIAE VKGGDRIPADMRVIQAQGFK

Eaff AYTIQATN-FEEPPDDNLYL GVVLTAVVTVTGVFSYYQES KSAKIMESFKNLVPQYALVR RGGEKLNIEAKDLTIGDIVE VKFGDRLPADVRVLEARGFK

Lcyp AYSIQATV-YEEPPDDNLYL GIVLAIVVIVTGIFSYYQES KSSKIMESFKNMVPQYALCL RDGEKLTIRAEELTYGDIIE VKFGDRLPADIRILEARGFK

Lsal AYSIQAST-FEEPPDDNLYL GIVLTAVVVITGIFSYYQES KSSKIMESFKNMVPQYALCL REGVKVNIKAEQLTLGDVVE VKFGDRIPADIRVTESRGFK

Aamp AYSIQAGT-FEEPPDDNLYL GIVLSAVVIITGCFSYYQES KSSRIMESFKNMVPQYALVI RDGSKATVRAEELTLGDLVE IKFGDRIPADVRIIEARNMK

Afra AYGIEASSGNEDMLKDNLYL GIVLATVVIVTGIFSYYQEN KSSRIMDSFKNLVPQYALAL REGQRVTLKAEELTMGDIVE VKFGDRVPADLRVLEARSFK

Hazt -------------------- -------------------- -------------------- -------------------- --------------------

Meda -------------------- -------------------- -------------------- -------------------- --------------------

Afos AYSIQASA-FEEPPDDNLYL GIVLSAVVTVTGIFSYYQES KSDKIMESFKNLVPQYALAR RGGEKLNIKAEELTIGDVVE IKFGDRIPADVRVIDAKGFK

Dmel AYSIQAST-SEEPADDNLYL GIVLSAVVIVTGIFSYYQES KSSKIMESFKNMVPQFATVI RGGEKLTLRAEDLVLGDVVE VKFGDRIPADIRIIEARNFK

Tcas AYSIQAST-VEEPADDNLYL GIVLAAVVIVTGIFSYYQES KSSKIMESFKNMVPQFATVI REGEKLTLRAEDLVLGDVVE VKFGDRIPADIRIIESRGFK

Pmon -------------------- -------------------- -------------------- -------------------- --------------------

Smar AYGITAAT-QENPVADNLYL GIVLATVVIVTGIFSYYQEA KSSKIMESFKSMVPQYAIVI RNEQKLSIRAEELVVGDVVE IKFGDRVPADVRVLQAHGFK

Tcal AYSIQAGA-YEEPPDDNLYL GIVLTVVVIVTGVFSYYQES KSSKIMESFKNLVPQYALAL RDGEKLTIKAEELTLGDIIE VKFGDRVPADIRVVEARGFK

Crog AYSIQASA-YEEPPDDNLYL GIVLTVVVVVTGVFSYYQES KSSKIMESFKNLVPQYALCV RDGKKVEIKAEELTLGDIIE VKFGDRVPADMRVIESRGFK

Dpul AYSIEVST-EEDVLGDNLYL GIVLTAVVVVTGVFSYYQES KSNKIMESFKNMVPQYAVVV REGLKQTMKAEEICIGDIVE VKFGDRIPADIRIIEARSFK

Aver -------------------- -------------------- -------------------- -------------------- --------------------

Csin VDNSSLTGESEPQLRTNEYT HENPLETKNLAFFSTNAVEG TARGVVVNIGDNTVMGRIAG LASGLDSGETPIAKEIAHFI HIITGVAVFLGVSFFIIAFA

Isca VDNSSLTGESEPQTRSPEMT NENPLETRNVAFFSTNCVEG TGMGVVINTGDRTVMGRIAN LASGLEMGETPIAREISHFI HLITGVAVFLGVTFFVIAFI

Eaff VDNSSLTGESEPCSRSPEFT HENPLETKNLAFFSTNAVEG TCVGVVVHIGDNTVMGRIAG LASGLDTGETPIAKEIGHFI HLITGVAVFLGVTFFIIAFI

Lcyp VDNSSLTGESEPQSRSPEFT HENPLETRNLAFFSTNAVEG TCRGIVVNIGDNTVMGRIAG LASGLDTGDTPIAKEIAHFI HIITGVAVFLGVTFFMIAFI

Lsal VDNSSLTGEAEPQSRSPDFT HENPLETKNLAFFSTNAVEG TATGIVVNIGDKTVMGRIAG LASGLETGETPIAKEIA--- ----AVAVFLGVTFFIIAFI

Aamp VDNSSLTGESEPQSRSPEYT AENPLETKNLAFFSTNAVEG TCKGVVINIGDNTVMGRIAG LTSSIDSGSTPIAREIEHFI HIITGVAVFLGVTFFVIALI

Afra VDNSSLTGESEPQARSPEFT NDNPLETKNLAFFSTNAVEG TMRGIVIGIGDNTVMGRIAG LASGLDTGETPIAKEIAHFI HIITGVAVFLGVTFFIIAFV

Hazt -------------------- -------------------- -------------------- -------------------- --------------------

Meda -------------------- -------------------- -------------------- -------------------- --------------------

Afos VDNSSLTGESEPQSRGPECT HENPLETKNLAFFSTNAVEG TCVGIVVHIGDGTVMGRIAG LASGLSTGETPIAKEIAHFI HIITGVAVFLGVTFFIIAFI

Dmel VDNSSLTGESEPQSRGAEFT HENPLETKNLAFFSTNAVEG TAKGVVISCGDHTVMGRIAG LASGLDTGETPIAKEIHHFI HLITGVAVFLGVTFFVIAFI

Tcas VDNSSLTGESEPQSRSPEFT HENPLETKNLAFFSTNAVEG TAKGVVISCGDNTVMGRIAG LASGLDTGETPIAKEIHHFI HLITGVAVFLGVTFFVIAFI

Pmon -------------------- -------------------- -------------------- -------------------- --------------------

Smar VDNSSLTGESEPQSRNPEYT SENPLETKNLAFFSTNAVEG TCRGLVISTGDRTVMGRIAN LASGLESGETPIAKEIQHFI HIITGVAVFLGVSFFIIAFI

Tcal VDNSSLTGESEPQARSAEFT HENPLETKNLAFFSTNAVEG TARGIVVNIGDNTVMGRIAG LASGLESGDTPIAKEIAHFI HIITGVAVFLGVTFFIIAFV

Crog VDNSSLTGESEPQSRSPEFT HENPLETKNLAFFSTNAVEG TARGIVVNIGDNTVMGRIAG LASGLESGDTPIAKEIAHFI HLITGVAVFLGMTFFGIAII

Dpul VDNSSLTGESEPQSRSSEFT NENPLETKNLAFFSTNAVEG TARAIVISIGDNTVMGRIAG LTSGLSSGETPIAREIAHFI HLITGVAVFLGVTFFVIALI

Aver -------------------- -------------------- -------------------- -------------------- --------------------

Csin LGYNWLDAVIFLIGIIVANV PEGLLATVTVCLTLTAKRMA SKNCLVKNLEAVETLGSTST ICSDKTGTLTQNRMTVAHMW FDNKIVEADTSEDQSGTSFN

Isca LGYHWLDAVIFLIGIIVANV PEGLLATVTVCLTLTAKRMA AKNCLVKNLEAVETLGSTST ICSDKTGTLTQNRMTVAHMW FDNQIIEADTTEDQSGVQYD

Eaff LGYHWLDAVIFLIGIIVANV PEGLLATVTVCLTLTAKRMA SKNCLVKNLEAVETLGSTST ICSDKTGTLTQNRMTVAHMW FDNKIHEADTSEDQSGNAFN

Lcyp LGYHWLDAVIFLIGIIVANV PEGLLATVTVCLTLTAKRMA SKNCLVKNLEAVETLGSTST ICSDKTGTLTQNRMTVAHMW FDNQIHDADTSEDQSGSSNY

Lsal LGYHWLDAVIFLIGIIVANV PEGLLATVTVCLTLTAKRMA SKNCLVKNLEAVETLGSTST ICSDKTGTLTQNRMTVAHMW FDNQIVEADTSEDQSGVSMN

Aamp LGYNWLDAVIFLIGIIVANV PEGLLATVTVCLTLTAKRMA SKNCLVKNLEAVETLGSTST ICSDKTGTLTQNRMTVAHMW YDNQIMEADTTEDQSGASYD

Afra LGYHWLDAVVFLIGIIVANV PEGLLATVTVCLTLTAKRMA SKNCLVKNLEAVETLGSTST ICSDKTGTLTQNRMTVAHMW FDGTITEADTTEDQSGAQFD

Hazt -------------------- -------------------- -------------------- -------------------- --------------------

Meda -------------------- -------------------- -------------------- -------------------- --------------------

Afos LGYHWLDAVIFLIGIIVANV PEGLLATVTVCLTLTAKRMA SKNCLVKNLEAVETLGSTST ICSDKTGTLTQNRMTVAHCW FDNKIQDVDTSEDQSGKGFD

Dmel LGYHWLDAVIFLIGIIVANV PEGLLATVTVCLTLTAKRMA SKNCLVKNLEAVETLGSTST ICSDKTGTLTQNRMTVAHMW FDNQIIEADTTEDQSGVQYD

Tcas LGYHWLDAVIFLIGIIVANV PEGLLATVTVCLTLTAKRMA SKNCLVKNLEAVETLGSTST ICSDKTGTLTQNRMTVAHMW FDNQIIEADTTEDQSGVQYD

Pmon -------------------- -------------------- -------------------- -------------------- --------------------

Smar LQYQWLEAVIFLIGIIVANV PEGLLATVTVCLTLTAKRMA AKNCLVKHLEAVETLGSTST ICSDKTGTLTQNRMTVAHMW FDNQIIEVDTTENQSGLQYD

Tcal LGYHWLDAVIFLIGIIVANV PEGLLATVTVCLTLTAKRMA SKNCLVKNLEAVETLGSTST ICSDKTGTLTQNRMTVAHMW FDNQIVEADTSEDQTGSSNY

Crog LGYHWLDAVIFLIGIIVANV PEGLLATVTVCLTLTAKRMA SKNCLVKNLEAVETLGSTST ICSDKTGTLTQNRMTVAHMW FDSQIIEADTSEDQSGVSMN

Dpul LGYQWLDAVIFLIGIIVANV PEGLLATVTVCLTLTAKRMA SKNCLVKNLEAVETLGSTST ICSDKTGTLTQNRMTVAHMW FDGQIIEADTTEDQSGAQYD

Aver -------------------- -------------------- -------------------- -------------------- --------------------

Csin KEASGWKTLERVACLCNRAE FKPQQENVPILRREVNGDAS EAAILKCTELSKGNVMDYRK RNKKVCEIPFNSANKYQVSI HET-EDPNDNRYLLVMKGAP

Isca KTSAGWKSLARNCCLCSRAE FKAGQENVPILKRDCTGDAS ESAILKCMELAIGNVNAYRQ RNPKVCEIPFNSTNKYHVTV HET-EDPDDPSYIILMKGAP

Eaff KNAPGWKTLERVGALCNRAE FKSGQDNVPILKREVNGDAS EAAILKCTELSKGNIIQYRN ANKKVCEIPFNSSNKFQVSI HEQ-EDKNDQRYLLVMKGAP

Lcyp KNMGGWKRLERCAALCNRAE FKPGQSGIPVLKRDVNGDAS ESALLKCVELSIGNVVKFRE QNRKVCEIPFNSTNKYQVSI HET-ND-GDDRYLLVMKGAP

Lsal KNAPGWKALCRIAALCNRAE FKPGQDNVPILKREVNGDAS EAALLKCCELSMGQVMEYRR RNKKVCELPFNSTNKYQVSI HET-DDKNDNRYLLVMKGAP

Aamp RTTEGWKALSRVAALCNRAE FKVGQESMPVLKRDVNGDAS EAALLKCCELAMGNVMGYRD KYKKVCEIPFNSTNKFQVSI HDIRHENPKNPNLLVMKGAP

Afra KSSAGWKALVKIAALCSRAE FKPNQSTTPILKREVTGDAS EAAILKCVELTTGETEAIRK RNKKICEIPFNSANKFQVSI HEN-EDKSDGRYLLVMKGAP

Hazt -------------------- -------------------- -------------------- -------------------- --------------------

Meda -------------------- -------------------- -------------------- -------------------- --------------------

Afos KNSPGWGPLQRVAMLCNRAE FKAGQADTPILKREVNGDAS EAAILKCTELTHGNVLDYRA KNKKVCEIPFNSTNKFQVSV HENGEDPSDKRYFLVMKGAP

Dmel RTSPGFKALSRIATLCNRAE FKGGQDGVPILKKEVSGDAS EAALLKCMELALGDVMNIRK RNKKIAEVPFNSTNKYQVSI HET-EDTNDPRYLLVMKGAP

Tcas RTSPGFKALSRIATLCNRAE FKGGQNDVPILKREVNGDAS EAALLKCMELALGDVMSIRR KNKKVCEIPFNSTNKYQVSI HEN-EDASDPRHILVMKGAP

Pmon -------------------- -------------------- -------------------- -------------------- --------------------

Smar KTSIGFLALARVAALCNRAE FKIGQENAPILKRETTGDAS ESALLKCIEIAIGNVATMRQ RNKS-------------VSI HE--DDSKEPGYLLVMKGAP

Tcal KTLPGWKPLERIGTLCNRAE FKPGQAGVSVLKREVNGDAS EAAILKCTELSTGDAMAYRN KHKKVCEVPFNSTNKYQVSI HEV-EGK--DSYLLVMKGAP

Crog KNAPGWTALSRVAALCNRAE FKPGQDNVPILKREVNGDAS EAALLKCCELSMGQVMEYRR RNKKVCEIPFNSTNKYQVSV HET-DDKNDNRYLLVMKGAP

Dpul KTSSGWKALSRVAGLCSRAE FKPGQDNIPILKREVNGDAS EAALLKCIELATGEAMAIRA RNKKVCEVPFNSTNKYQVSL HDN-EDKNDGRYFLAMKGAP

Aver -------------------- -------------------- -------------------- -------------------- --------------------

Csin ERILERCTTIVVDGREMPLT EEWKNSFETAYMELGGLGER VLGFCDFMLPADKFPNGYPF DADDVNFPVEGLRFVGLMSM IDPPRAAV--PDAVLKC---

Isca ERILDRCSTIFINGKEKVLD DELKEAFNNAYLELGGLGER VIGFCDYKLPTEKYPPGYPF DADEQNFPLTGLCFLGLVSM IDPPRAAV--PDAVAKC---

Eaff ERILERCSTIIIDGTERELN QEWQDSFNNAYMELGGLGER VLGFCDMLLPADKYPRGYPF DADEENFPLEGLRFVGLMSM IDPPRAAV--PDAVAKC---

Lcyp ERILERSTTIYVDGTDIELN DYWRSAFNRAYLELGGLGER VLGFCDLRLPVNEFPRGFNF DSEEVNFPVANLRFLGLMSM IDPPRAAV--PEAVAKC---

Lsal ERIMDRCGSILVNGEEKELN DDWRDAFNSAYLELGGLGER VLGFCDFILP---------- -------------------- -----AAV--PDAVTKC---

Aamp ERILDRCSTILINGQDKPLD QEMKDAFNNAYLELGGLGER VLGFCDFSLSGDKYPSDYPF DSDEVNFPLQDLRFVGLMSM IDPPRAAV--PDAVSKC---

Afra ERILERCSTIFMNGKEIDMT EELKEAFNNAYMELGGLGER VLGFCDYLLPLDKYPHGFAF NADDANFPLTGLRFAGLMSM IDPPRAAV--PDAVAKC---

Hazt -------------------- -------------------- -------------------- -------------------- --------------------

Meda -------------------- -------------------- -------------------- -------------------- --------------------

Afos ERILDRCSTIIVDGTERPLN DEWKDAFNTAYMSLGGLGER VLGFCDYTLPADKYPRGYPF DPDESNFPLEGLRFVGLMSM IDPPRAAV--PDAVMKC---

Dmel ERILERCSTIFINGKEKVLD EEMKEAFNNAYMELGGLGER VLGFCDFMLPSDKYPNGFKF NTDDINFPIDNLRFVGLMSM IDPPRAAV--PDAVAKC---

Tcas ERILERCSTIFICGKEKVLD EEMKEAFNNAYLELGGLGER VLGFCDFMLPTDKYPIGYKF NCDDPNFPLDGLRFVGLMSM IDPPRAAV--PDAVAKC---

Pmon -------------------- -------------------- -------------------- -------------------- --------------------

Smar ERILERCTTILMDDKEITMS EDWKDAFNQAYLELGGLGER VLGFCDRKLSPEKFPSGYPF DSDEENFPLTGLRFVGLMSM IDPPRAAV--PDAVSKC---

Tcal ERILDRCSTILIDGEELEMS QEWKDNFNNAYMELGGLGER VLGFCDFELPANKFPKGYPF NQDDINFPIEGLRFSGLMSM IDPPRAAV--PDAVAKC---

Crog ERIMDRCRTILVNGEEKELT DDWRDAFNSAYMELGGLGER VLGFCDFILPADKFPEGYPF DADDANFPLEGLRFVGLMSM IDPPRAAV--PDAVAKC---

Dpul ERILDVCSTILLNGVEKPLD NEMRDAFNTAYMELGGLGER VLGFCDTLLPLDKFPKGYAF NADDPNFPLTGLRFVGLISM IDPPRAAV--PDAVAKC---

Aver -------------------- -------------------- -------------------- -------------------- --------------------

Csin RSAGIKVIMVTGDHPITAKA IARSVGIISEGTDTVEDIAA RTGVDVKDVNPREARAAVIH GGEIKDMNEKQIDEILMYHT -----EIVFARTSPQQKLII

Isca RSAGIKVIMVTGDHPITAKA IAKAVGIISEGNETVEDIAQ RLNIPVEEVNPRDAKAAVIH GSELRDITPEQLDDILRYHT -----EIVFARTSPQQKLII

Eaff RSAGIKVIMVTGDHPITAKA IAKSVGIISDGQETVDDIAE RLNIDVSKVNPRDAKAAVVH GGELKDMSEKALDDLLMYHT -----EIVFARTSPQQKLII

Lcyp RSAGIKVIMVTGDHPITAKA IAKAVGIISENQETVEDIAQ RLNVPVEQVDPTQAKACVVH GNDLKSMSASEIDGLLRAHS -----EIVFARTSPQQKLII

Lsal RSAGIKVIMVTGDHPITAKA IAKSVGIISEGTETVDDIAT RLNIPLEDVNPREAKAAVVH GGELKDLTTVQLDEILLYHT -----EIVFARTSPQQKLII

Aamp RSAGIKVIMVTGDHPITAKA IAKS---------------- -------------------- -------------------- --------------------

Afra RSAGIKVIMVTGDHPITAKA IAKSVGIISEGNETVEDIAA RLNIPVSEVNPRDAKAAVVH GGELRDITPDALDEILRHHP -----EIVFARTSPQQKLII

Hazt -------------------- -------------------- -------------------- -------------------- --------------------

Meda -------------------- -------------------- -------------------- -------------------- --------------------

Afos RSAGIKVIMVTGDHPITAKA IAKSVNIIMEGSETVEDIAE RLGCDVSKVNPRDARAAVVH GGELKDMDGKQLDDILMYHN -----EIVFARTSPQQKLII

Dmel RSAGIKVIMVTGDHPITAKA IAKSVGIISEGNETVEDIAQ RLNIPVSEVNPREAKAAVVH GAELRDVSSDQLDEILRYHT -----EIVFARTSPQQKLII

Tcas RSAGIKVIMVTGDHPITAKA IAKSVGIISEGNETVEDIAQ RLNIPVSEVNPREAKAAVVH GSDLRDLSSDQLDEILRYHT -----EIVFARTSPQQKLII

Pmon -------------------- -------------------- -------------------- -------------------- --------------------

Smar RSAGIKVIMVTGDHPITAKA IAKSVGIISEGNETVEDIAT RMEIPLEEVNLQEANAAVVH GSELKEMTQEQLDDVLRNHT -----EIVFARTSPQQKLII

Tcal RSAGIKVIMVTGDHPITAKA IAKSVGIISEGNETVEDIAE RQGIPVDKVNPRDARAAVVH GGELKDITEDQLDEILMYHT -----EIVFARTSPQQKLII

Crog RSAGIKVIMVTGDHPITAKA IAKSVGIISEGNETVEDIAS RLNIPINEVNPRDAHAAVVH GGELKEITEDHLDEILMYHT -----EIVFARTSPQQKLII

Dpul RSAGIKVIMVTGDHPITAKA IAKSVGIISEGNETVEDMAQ RLNIPVEEVDKRLAKAAVVH GGELRDMDSDDLDDVLRNHT -----EIVFARTSPQQKLII

Aver -------------------- -------------------- -------------------- -------------------- --------------------

Csin VEGCQRMGAIVAVTGDGVND SPALKKADIGVAMGIAGSDV SKQAADMILLDDNFASIVTG VEEGRLIFDNLKKSIAYTLT SNIPEISPFLLFILADVPLP

Isca VEGCQRLGAIVAVTGDGVND SPALKKADIGVAMGISGSDV SKQAADMILLDDNFASIVTG VEEGRLIFDNLKKSIAYTLT SNIPEISPFLLFILADVPLP

Eaff VEGCQRMGAIVAVTGDGVND SPALKKADIGVAMGIAGSDV SKQAADMILLDDNFASIVTG VEEGRLIFDNLKKSIAYTLT SNIPEISPFLLFILADVPLP

Lcyp VEGCQRQRAIVAVTGDGVND SPALKKADIGVAMGIAGSDV SKQAADMILLDDNFASIVTG VEEGRLIFDNLKKSIAYTLT SNIPEITPFLIYMITDIPLP

Lsal VEGCQRMGAIVAVTGDGVND SPALKKADIGVAMGIAGSDV SKQAADMILLDDNFASIVTG VEEGRLIFDNLKKSIAYTLT SNIPEISPFLLFILADIPLP

Aamp -------------------- -------------------- -------------------- -------------------- --------------------

Afra VEGCQRQGAIVAVTGDGVND SPALKKADIGVAMGIAGSDV SKQAADMILLDDNFASIVTG VEEGRLIFDNLKKSIVYTLT SNIPEISPFLLFILFDIPLP

Hazt -------------------- -------------------- -------------------- -------------------- --------------------

Meda -------------------- -------------------- -------------------- -------------------- --------------------

Afos VEGCQRMGAIVAVTGDGVND SPALKKADIGVAMGIAGSDV SKQAADMILLDDNFASIVTG VEEGRLIFDNLKKSIAYTLT SNIPEISPFLLFILADVPLP

Dmel VEGCQRMGAIVAVTGDGVND SPALKKADIGVAMGIAGSDV SKQAADMILLDDNFASIVTG VEEGRLIFDNLKKSIAYTLT SNIPEISPFLAFILCDIPLP

Tcas VEGCQRMGAIVAVTGDGVND SPALKKADIGVAMGIAGSDV SKQAADMILLDDNFASIVTG VEEGRLIFDNLKKSIAYTLT SNIPEISPFLAFILCDIPLP

Pmon -------------------- -------------------- -------------------- -------------------- --------------------

Smar VEGCQRLGAIVAVTGDGVND SPALKKADIGVAMGIAGSDV SKQAADMILLDDNFASIVTG VEEGRLIFDNLKKSIAYTLT SNIPEISPFLFFIVANIPLP

Tcal VEGCQRMGAIVAVTGDGVND SPALKKADIGVAMGIAGSDV SKQAADMILLDDNFASIVTG VEEGRLIFDNLKKSIAYTLT SNIPEISPFLLFILADVPLP

Crog VEGCQRMGAIVAVTGDGVND SPALKKADIGVAMGIAGSDV SKQAADMILLDDNFASIVTG VEEGRLIFDNLKKSIAYTLT SNIPEISPFLLFILADVPLP

Dpul VEGCQRLGAIVAVTGDGVND SPALKKADIGVAMGIAGSDV SKQAADMILLDDNFASIVTG VEEGRLIFDNLKKSIAYTLT SNIPEISPFLLFICCDIPLP

Aver -------------------- -------------------- -------------------- -------------------- --------------------

Csin LGTVTILCIDLGTDMVPAIS MAYEQAESDIMKRQPRNPFT DKLVNERLISMAYGQIGMIQ ASAGFFVYFVILCENGFWPS RLFGIRRAWDSAAVNDLEDS

Isca LGTVTILCIDLGTDMLPAIS LAYETAESDIMKRQPRNPLK DKLVNESL-SLFKGRLCSVG GMSKFRVAGMQKRQKEMQAH FLVS------SHAVNDLMDS

Eaff LGTVTILCIDLGTDMVPAIS MAYEQAESDIMKRQPRNPFT DKLVNERLISMAYGQIGMIQ ASAGFFVYFVILCENGFWPS HLLGLRRAWDSVAVNDLEDS

Lcyp LGTITILCIDLGTDMVPAIS LAYEKAENDIMKRRPRDPQH DRLVNERLISMAYGQIGMIQ ASAGFFVYLVIMAENGFWPS RLLGLRKSWESKTINDLEDS

Lsal LGTVTILCIDLGTDMIPAIS MAYEEAESDIMKRMPRNPFS DKLVNERLISMAYGMIGMIQ AA------------------ --------------------

Aamp -------------------- -------------------- -------------------- -------------------- --------------------

Afra LGTVTILCIDLGTDMVPAIS LAYEEAESDIMKRRPRNPVT DKLVNERLISLAYGQIGMIQ ASAGFFVYFVIMAECGFLPW DLFGLRKHWDSRAVNDLTDS

Hazt -------------------- -------------------- -------------------- -------------------- --------------------

Meda -------------------- -------------------- -------------------- -------------------- --------------------

Afos LGTVTILCIDLGTDMVPAIS MAYEQAESDIMKRQPRNPFT DKLVNERLISMAYGQIGMIQ ASAGFFVYFVILAENGFWPG KLFGLRRAWDSLAINDLEDS

Dmel LGTVTILCIDLGTDMVPAIS LAYEHAEADIMKRPPRDPFN DKLVNSRLISMAYGQIGMIQ AAAGFFVYFVIMAENGFLPK KLFGIRKMWDSKAVNDLTDS

Tcas LGTVTILCIDLGTDMVPAIS LAYEAPESDIMKRQPRDPYR DNLVNRRLISMAYGQIGMIQ AAAGFFVYFVIMAENGFRPT DLFGIRKQWDSKAVNDLTDS

Pmon -------------------- -------------------- -------------------- -------------------- --------------------

Smar LGTVTILCIDLGTDMVPAIS LAYEKAETDIMKRKPRDPFR DKLVNERLISMSYGQIGMIQ ASAGFFSYFVIMAENGFWPS KLFGLRKSWDASSINDLRDS

Tcal LGTVTILCIDLGTDMVPAIS MAYEEAESDIMKRQPRNPFT DKLVNERLISMAYGQIGMIQ ASAGFFVYFVILAENGFLPS RLFGLRRAWDSQAVNDLEDS

Crog LGTVTILCIDLGTDMIPAIS MAYEEAESDIMKRQPRNPFT DKLVNERLISMAYGQIGMIQ ASAGFFVYFVILAENGFFPS KLLGLRRAWDSPAINDLQDS

Dpul LGTVTILCIDLGTDMVPAIS LAYESAENDIMKRPPRCPKK DKLVNERLISMAYGQVGMIQ ASAGFFVYFVIMAENGFRPD LLFGIRKNWDSRAVNDLQDS

Aver -------------------- -------------------- -------------------- -------------------- --------------------

Csin YGQEWTYSDRKILEYTCHTA FFVSIVVVQWADLIICKTRK NSVFQQGMKNHFMNFGLCFE TALAAFLSYTPGMDKGLRMY PLKINWWLPALPFSLAIFCY

Isca YGQEWTYKNRKTLEYTCHTA FFVSIVIVQWADLIICKTRR NSILHQGMRNHVLNIGLIFE TALAAILSYTPGMDKGLRMY PLKINWWLPAMPFSLLIFVY

Eaff YGQEWTYKDRKVLEYTCHTA FFVSIVIVQWADLIICKTRK NSVFQQGMKNWFMNFGLVFE TLLAAFLSYTPGMDKGLRMY PLKFNWWLPALPFSLIIFIY

Lcyp YGQEWTFSQRKVLEYTCHTA FFVSIVIVQWADLIICKTRR NSLYHQGMTNWMLNFGLVFE TVLAAALCYTPYLDKGLNMY PLKFYWWLPALPFSLAIFIY

Lsal -------------------- -------------------- -------------------- -------------------- --------------------

Aamp -------------------- -------------------- -------------------- -------------------- --------------------

Afra YGQEWTYDARKQLESSCHTA YFVSIVIVQWADLIISKTRR NSVFQQGMRNNILNFALVFE TCLAAFLSYTPGMDKGLRMY PLKINWWFPALPFSFLIFVY

Hazt -------------------- -------------------- -------------------- -------------------- --------------------

Meda -------------------- -------------------- -------------------- -------------------- --------------------

Afos YGQEWTYKDRKILEYTCHTA FFVSIVVVQWADLIICKTRK NSVFQQGMRNWFMNFGLVFE TVLAAFLSYTPGMDKGLRMY PLKINWWLPAMPFSLLIFVY

Dmel YGQEWTYRDRKTLEYTCHTA FFISIVVVQWADLIICKTRR NSIFQQGMRNWALNFGLVFE TVLAAFLSYCPGMEKGLRMY PLKLVWWFPAIPFALAIFIY

Tcas YGQEWTYRDRKTLEYTCHTA FFVSIVVVQWADLIICKTRR NSILHQGMRNWALNFGLVFE TALAAFLSYTPGMDKGLRMF PLKFVWWLPAIPFMLSIFIY

Pmon -------------------- -------------------- -------------------- -------------------- --------------------

Smar YNQEWTYDDRKKLEYTCQTA FFVSIVVVQWADLIICKTRR LSLFHQGMTNHVMNFGLCFE TVLACILCYTPGLDKGLRMY PLKFVWWLPAIPFSITIFIY

Tcal YGQEWTYRDRKVLEYTCHTA FFVSIVIVQWADLIICKTRR LSVFQQGMKNHFMNFGLVFE TVLAAFLCYTPGMDKGLRMY PLKFNWWLPALPFSLLIFVY

Crog FGQEWTYHDRKTLEYTCHTA FFVSIVVVQWADLIICKTRR NSVFQQGMNNMFMNFGLIFE TALAAFLSYTPGMDKGLRMY PLKFHWWLPAMPFSLLIFVY

Dpul YGQEWTYDARKQLEYTCHTA FFVSIVVVQWADLIICKTRR NSIVHQGMRNHVLNFGLLFE TAMAAFLSYTPGMDKGLRMY PLKINWWLPAIPFSILIFVY

Aver -------------------- -------------------- -------------------- -------------------- --------------------

Csin DETRKFLLRRN-PGGWIEKE T-YY-------------XTD IALIGLAVMGQNLILNMNDN GFKVVAYNRTTAKVDDFLKN EAKNTDVVGAHSLEEMVAKL

Isca DEIRRFILRRN-PGGWVEME T-YYLL--------SLCRAD IGLIGLAVMGQNLILNMNDH GHVVCAFNRTTEKVDEFLKK EAKGTKVIGAHSLEELVSKL

Eaff DESRKFLLRRN-PGGWIEQE T-YYMG---------EPTGD IALIGLAVMGQNLILNMNDH GFVVVAYNRTTAKVDQFLEN EAKGTKVVGAHSLEEMVAKL

Lcyp DEVRKYIIRRN-PGGWVEQE T-YYMS--------DEGVAD IALIGLAVMGQNIILNMNDH GFKVCAFNRTVDKVEAFLKN EAKGTNVIGAKSLEDMVKKL

Lsal -------------------- -------------------- -------------------- -------------------- --------------------

Aamp -------------------- -------------------- -------------------- -------------------- --------------------

Afra DEARKFILRRN-PGGWVEQE T-YY---------------- -------------------- -------------------- --------------------

Hazt -------------------- -------------------- -------------------- -------------------- --------------------

Meda -------------------- -------------------- -------------------- -------------------- --------------------

Afos DECRKYLLRRNGPGSWIEQE T-YYMS---------EPVGD IALIGLAVMGQNLILNMNDH GFTVVAYNRTTAKVDNFLEN EAKGTKVVGAHSLEEMVAKL

Dmel DETRRFYLRRN-PGGWLEQE T-YYMS----------GQAD IALIGLAVMGQNLILNMDEK GFVVCAYNRTVAKVKEFLAN EAKDTKVIGADSLEDMVSKL

Tcas DETRRFYLRRN-PGGWLEQE T-YYMA---------QGKGD IGLIGLAVMGQNLILNMADH GFTVVAFNRSVEKVHAFLAN EAKGKCIIGAESIPDLVSKL

Pmon -------------------- -------------------- -------------------- -------------------- --------------------

Smar DECRKYILRRN-PGGWGKKK NEKMMGNSPCSKHEKEHIAD IAMIGLAVMGQNLVLNMNDH GFVVCAYNRTTSKVDEFLAK EAKGTLVVGAHTLEEMISKL

Tcal DESRKYILRHQPHGAWLERE T-YYMAP-------QEPVAD IALIGLAVMGQNIILNMNDH GFVVCAYNRTQEKVKEFLET GAKGTKVVGATSVEDMVGKL

Crog DECRKLLIRRLPPGNWVERE T-YYMS---------EPVAD IALIGLAVMGQNIILNMNDH GYVVCAYNRTTEKVDNFLAN EAKGTKIVGAHSLEEMVAKL

Dpul DECRKFILRRN-PGGWVERE T-YYMS--------AEQVAD IGLIGLAVMGQNLILNMADH GFVVCAFNRTTSKVDDFLHN EAKGKSVVGAHSVRELVAKL

Aver -------------------- -------------------- -------------------- -------------------- --------------------

Csin KKPRRVMMLVKAGSAVDAFI EKLVPLLEAGDIIIDGGNSE YQDTVRRTQDLEKKGILFVG SGVSGGEEGARYGPSLMPGG NPAAWPAIKPIFQAICAKV-

Isca KTPRRIVLLVKAGQAVDDFI NKLTPLLTKGDILIDGGNSE YQDSERRCEALKAKGILFVG SGVSGGEEGARYGPSLMPGG NKEAWPHIKDIFQGIAAKAD

Eaff KKPRKVMMLVKAGSAVDDFI AKLVPLLEQGDIIIDGGNSE YQDTRRRTKDLESKGLLFVG SGVSGGEEGARYGPSLMPGG NPAAWPALKPIFQSICAKSG

Lcyp KKPRRVMLLVKAGSAVDAFI DQLVPLLEAGDIIIDGGNSE YQDTNRRCQTLDGKGILYVG SGVSGGEEGARHGPSLMPGG NIKAWPAIQPIFQSICAKVD

Lsal -------------------- -------------------- -------------------- -------------------- --------------------

Aamp -------------------- -------------------- -------------------- -------------------- --------------------

Afra -------------------- -------------------- -------------------- -------------------- --------------------

Hazt -------------------- -------------------- -------------------- -------------------- --------------------

Meda -------------------- -------------------- -------------------- -------------------- --------------------

Afos KRPRKVMMLVKAGSAVDDFI EKLVPLLEKGDIIIDGGNSE YQDTRRRTADLEKRGILYVG SGVSGGEEGARYGPSLMPGG NPAAWPSIKPIFQAICAKSG

Dmel KSPRKVMLLVKAGSAVDDFI QQLVPLLSAGDVIIDGGNSE YQDTSRRCDELAKLGLLFVG SGVSGGEEGARHGPSLMPGG HEAAWPLIQPIFQAICAKAD

Tcas KTPRRVMMLVKAGDAVDSFI DQLVPHLQKGDIIIDGGNSE YQDSERRCKDLRAKGIRFVG AGVSGGEDGARYGPSLMPGG NKEAWPYIKDIFQSVAAKTD

Pmon -------------------- -------------------- -------------------- -------------------- --------------------

Smar KKPRRVMMLVKAGTAVDDFI EKLLPLLENGDIIIDGGNSE YQDTQRRCQSLKAKGIWFVG SGVSGGEDGARYGPSLMPGG QREAWPHIKPIFQGIAAKVD

Tcal KKPRRVMMLVKAGDPVDAFI KQVVPFLEKGDIIIDGGNSE YLDTIRRVKELSEKGILFVG SGVSGGEEGARYGPSMMPGG NPEAWPHVKDIFQSICAKSN

Crog KKPRRVMMLVKAGAAVDAFI DKLVPLLEAGDIIIDGGNSE YQDTRRRTKDLSEKGILFVG SGVSGGEEGARYGPSLMPGG NPKAWPHIKPIFQAICAKSN

Dpul KKPRRVMMLVQAGKAVDQFI DQLVEFLEAGDIIIDGGNSE HGDTTRRCTTLREKGILYVG SGVSGGEEGARYGPSLMPGG HIEAWPHIKPIFQSISAKSD

Aver -------------------- -----------LICEAYHIM KDVLGMSHDEMAETFEEWNK GELDSFLIEISANILKYKDD SGDHLVEKIRDSAGQKGTGK

Csin -GEXXXXXXXXXXTGHFVKM VHNGIEYGDMQLICEAYHLM KDTLGMDNDEMSQTFKSWNE GELDSFLIEISTNILKFRDD SGEFLLEKIRDSAGQKGTGK

Isca -GEPCCDWVGDGGSGHFVKM VHNGIEYGDMQLIGEAYHLI KDATGILHFE--QVFDEWNK GVLDSFLIEITSNILKYKDT DGEPLVTKIRDAAGQKGTGK

Eaff -DEPCCDWVGEDGSGHFVKM VHNGIEYGDMQLICEAYHLM KDALNMDNEEMSNSFTEWNK GELDSFLIEISSNILKYKDE SGDYLLEKIRDSAGQKGTGK

Lcyp NGEPCCDWVGEEGSGHFVKM VHNGIEYGDMQLICEAYHLM KNVLGMDHDAMADTFAEWNK GELDSFLIQITANILKFKDD DGKPLLEKIKDSAGQKGTGK

Lsal -------------------- -------------------- -------------------- -------------------- --------------------

Aamp -------------------- -------------------- -------------------- -------------------- --------------------

Afra -------------------- -------------------- -------------------- -------------------- --------------------

Hazt -------------------- -------------------- -------------------- -------------------- --------------------

Meda -------------------- -----------LICEAYHIM KDFLGMNHDDMADTFLEWNK GELDSFLIEITAHILKHRDD DGDHLVEKIRDSAGQKGTGK

Afos -EEPCCDWVGEDGSGHFVKM VHNGIEYGDMQLICEAYHLM KDALGLNNDEMSSIFSEWNK GELDSFLIEISANILKYKDE SGDFLLEKIRDSAGQKGTGK

Dmel -GEPCCEWVGDGGAGHFVKM VHNGIEYGDMQLICEAYHIM Q-SLGLSADQMADEFGKWNS AELDSFLIEITRDILKYKDG KG-YLLERIRDTAGQKGTGK

Tcas -GQPCCDWVGNDGAGHFVKM VHNGIEYGDMQLIGEVYHMM Q-AIGVDQGKMAKTFAEWNK GELDSFLIDITKDIMGFKDT DGKYLLPKIRDTAGQKGTGK

Pmon -------------------- -------------------- -------------------- -------------------- --------------------

Smar -NEPCCDWVGEDGSGHFVKM VHNGIEYGDMQLICEAYHLL KDALDLSHDEMSHIFGEWNK GELDSFLIEITRDILKYKDD DGKPLVEKILDSAGQKGTGK

Tcal -GEPCCDWVGEGGSGHFVKM VHNGIEYGDMQLICEAYNIM KDVLGMSHDEMSEVLNEWNK GELDSFLVEISANILAYKDK DGQPLVEKIRDSAGQKGTGK

Crog -GEPCCDWVGEDGSGHFVKM VHNGIEYGDMQLICEAYHLM KDTLGLGHDEMSDIFKEWNK GELDSFLIEISTNILKYKDA DGSPLLEKIRDAAGQKGTGK

Dpul -GEPCCDWVGEGGAGHFVKM VHNGIEYGDMQLIAEAYHLM KSVLGMTPPEMADVFDEWNK GELDSFLIEITANILRFQDD KGDYLVEKIRDSAGQKGTGK

Aver WTAISALDYGMPVTLIGEAV FSRCLSSLKDERICASQVLS GPDNQGFTGDKSLFVEQIRQ ALYASKIISYAQGFMLLREA AKVFNWNLNYGGIALMWRGG

Csin WTAISALEYGMPVTLIGESV FARCLSSLKDEREKASTILH GPETTKYEGDKKEFVEAIRQ ALYASKIVSYAQGFMLLRQA AKEFGWNLNYGGIALMWRGG

Isca WTAISALQHGIPVTLIGEAV FARCLSSLKDQRVEASKILK GPNG-KYEGQASDFVEHVRK ALYASKIVSYAQGFMLLRSA AQEYKWSLNYGAIALMWRGG

Eaff WTAISALEYGMPVTLIGESV FARCLSSLKGERERASKILK GPEGAEFTGEKAAFVEDIRQ ALYASKIVSYAQGFMLLRQA AQEFGWNLNYGGIALMWRGG

Lcyp WTAISALDYGMPVTLIGEAV FARCLSSLRDERVRASAILK GPADVCFQGDKQQFIEQIRQ ALYASKIVSYAQGFMLLREA AKVFNWKLNYGGIALMWRGG

Lsal -------------------- -------------------- -------------------- -------------------- --------------------

Aamp -------------------- -------------------- -------------------- -------------------- --------------------

Afra -------------------- -------------------- -------------------- -------------------- --------------------

Hazt -------------------- -------------------- -------------------- -------------------- --------------------

Meda WTAISALDYGMPVTLIGEAV FARCLSSLKDERLSASGVLA GPALKSFDGDTADFVEQIRE ALYASKIVSYAQGFMLLREA AKVFNWNLNYGGIALMWRGG

Afos WTAISALEYGMPVTLIGESV FARCLSSLKGERERASSILK GPDAPSFDGDKDKFVEDIRQ ALYASKIVSYAQGFMLLRQA AVEFGWNLNYGGIALMWRGG

Dmel WTAIAALQYGVPVTLIGEAV FSRCLSALKDERVQASSVLK GPSTKAQVANLTKFLDDIKH ALYCAKIVSYAQGFMLMREA ARENKWRLNYGGIALMWRGG

Tcas WTAIAALNYGMPVTLIGEAV FSRCLSALKNERITASKVLP GPTA-KFTGNVDQFLDELRQ ALYASKIVSYAQGFMLLREA AGVHNWDLDYGSIALMWRGG

Pmon -------------------- -------------------- -------------------- -------------------- --------------------

Smar WTAIASLEYGMPVTLIGESV FARCLSSLKADRVNASKQLK GPTETKYKGDKKQFAEHIRK ALYASKIVSYAQGYMLLREA AKQFKWDLNYGGIALMWRGG

Tcal WTAISALQYGMPVTLIGESV FARCLSSIKDERTLASKSLK GPDAPKFDGDKKAFIEQIRQ ALYASKIISYAQGFMLLREA AKEEDWKLNYGGIALMWRGG

Crog WTAISALEYGMPVTLIGESV FARCLSSLKDERVRASKILK GPSNTKFNGDKKEFVEQIRQ ALYASKIISYAQGFMLLREA AKVFGWNLNYGGIALMWRGG

Dpul WTAIAALDYGVPVTLIGEAV FARCLSSLQAERIEASKVLP APAFKAYSGERASFIEAIRK ALYASKIVSYAQGFMLLREA AKQYDWTLNYGGIALMWRGG

Aver CIIRSVFLGNIKSAYDKNPD LTNLLLDDFFRDAIHGSQAA WRNVVATAVQSGVPAPCFST ALAFYDGYRSARLPANLIQA QR------------------

Csin CIIRSRFLGNIKAAFDKNPA LDNLLLDDFFRDAIHASQKS WRKVVSKAVELGVPTPCFST ALAFYDGYRCKKLPANLIQA QRDILEL----ILTSFWQNL

Isca CIIRSAFLGNIKAAFDKNPE LSCLLLDSFFVDAIQDCQAS WRLVVASAAQLGIPVPAFST ALAFYDGFRAEKLPANLIQA QRDYFGAHTYERLSEPGKFV

Eaff CIIRSRFLGNIKDAFDKNPA LDNLLVDDFFRDAIHNAQGA WRRVVSQAVLMGVPTPCFST ALAFYDGYRSDMLPANLIQA QRDYFGAHTYELLGQPGAYH

Lcyp CIIRSVFLGNIKAAFDKDPE LQNLLLDDFFRDAIHRCQDA WRRVLSTAVERGVPTPCFST ALAFYDGYRSARLPANLIQA QRDYFGAHTYELLSNPGTFV

Lsal -------------------- -------------------- -------------------- -------------------- --------------------

Aamp -------------------- -------------------- -------------------- -------------------- --------------------

Afra -------------------- -------------------- -------------------- -------------------- --------------------

Hazt -------------------- -------------------- -------------------- -------------------- --------------------

Meda CIIRSVFLGKIKAAFDKNPE LTNLLLDDFFRDAVHKCQAS WRNVVSAAVQAGIPTPCFST ALAFYDGYRCARLPANLIQA QR------------------

Afos CIIRSRFLGNIKAAFDKNPA LDNLLVDDFFRDAIHNAQAA WRRVVSQAVLMGIPTPCFST ALAFYDGYRSSMLPANLIQA QRDYFGAHTYELLSAPGTYV

Dmel CIIRSVFLGNIKDAYTSQPE LSNLLLDDFFKKAIERGQDS WREVVANAFRWGIPVPALST ALSFYDGYRTAKLPANLLQA QRDYFGAHTYELLGQEGQFH

Tcas CIIRSVFLGQIKLAFDRDPS LKSLLLAPFFLNAITRSQNA WRNVVSTAIKLGVPTPALST ALAFYDGYRSERLPANLLQA QRDYFGAHTYELLGQEGKFV

Pmon -------------------- -------------------- -------------------- -------------------- --------------------

Smar CIIRSAFLGNIKSAFDKNKS LSNLLLDDFFRKAIDDCQES WRLVIMEAVKLGIPTPCFAT ALAFYDGFRLANLPANLIQA QRDYFGAHTYERIDAPGKVV

Tcal CIIRSVFLGNIKSAYDKNPQ LENLLLDDFFRDAIHNCQKG WRKVVSTAVEMGIPTPCFST ALAYYDGYRSERLPANLIQA QRDYFGAHTYELLASPGKFV

Crog CIIRSVFLGNIKNAYDKSPK LENLLLDDFFKDAVHKCQDS WRKVVSKAVELGVPTPCFST ALAFYDGYRSSKLPANLLQA QRDYFGAHTYELLNNPGKHV

Dpul CIIRSVFLGNIKLAFERNPD LKSLLMDDFFRQAVGTCQES WRDVIATAAQYGVPVPAFST ALSFYDAYRCQRLPANLIQA QRDYFGAHTYELLDQPGVFH

Aver -------------------- -------------------- -------------------- -------------------- --------------------

Csin ASMSTLTGLALGAMFQHQLT QLEMSLTDQLLQHVI----- -------------------- -------------------- --------------------

Isca HTNWTGTGGNVSSS------ -----------SYNA----- -------------------- -------------------- --------------------

Eaff HTNWTGTGGNVSAS------ -----------TYSA-MSQV PCSKFTDNYELKEELGKGAF SIVKRCVQRHTGLEFAAKII NTKKLSARDFQKLEREARIC

Lcyp HTNWTGLGGNVSAS------ -----------TYDV-MAQT ASTKFTDNYELKEELGKGAF SVVRRCVQKNTGLEFAAKII NTKKLSARDFQKLEREARIC

Lsal -------------------- -------------------- -------------------- -------------------- --------------------

Aamp -------------------- -------------------- -------------------- -------------------- --------------------

Afra -------------------- ----------------M-NN ACTRFSDNYELKEELGKGAF SIVKRCVQKATGLEFAAKII NTKKLSARDFQKLEREGRIC

Hazt -------------------- -------------------- -------------------- -------------------- --------------------

Meda -------------------- -------------------- -------------------- -------------------- --------------------

Afos HTNWTGTGGNVSAS------ -----------TYSA----- -------------------- -------------------- --------------------

Dmel HTNWTGTGGNVSAS------ -----------TYQAMAAPA ACTRFSDNYDIKEELGKGAF SIVKRCVQKSTGFEFAAKII NTKKLTARDFQKLEREARIC

Tcas HTNWTGHGGNVSSS------ -----------SYNAMAVPI ACTRFSDNYDLKEELGKGAF SVVRRCVQKSTGLEFAAKII NTKKLSARDFQKLEREARIC

Pmon -------------------- -------------------- -------------------- -------------------- --------------------

Smar HTNWTGHGGDVSAS------ -----------TYKAMASQN TSTKFSDNYELKEELGKGAF SIVRRCVQKSTGLEFAAKII NTKKLSSRDFQKLEREARIC

Tcal HTNWTGTGGNISAS------ -----------TYDA-MAQN PSTKFTDNYELKEELGKGAF SVVRRCVQKATGLEFASKII NTKKLSARDFQKLEREARIC

Crog HTNWTGTGGNVSAS------ -----------TYDA-MAQN PTTKFTDNYELKEELGKGAF SVVRRCVQKSTGLDFASKII NTKKLSARDFQKLEREARIC

Dpul HANWTGHGGRTSAS------ -----------TYQA-MALN ATTRFSDNYELKEELGKGAF SIVRRCVQKSTGLEFAAKII NTKKLSARDFQKLEREARIC

Aver -------------------- -------------------- ---------------LESVN HCHMNGVVHRDLKPENLLLA SKQKGAAVKLADFGLAIEVQ

Csin -------------------- -------------------- -------------------- -------------------- --------------------

Isca ---------RLHDSIQEEGY HYLIFDLVTGGELFEDIVAR EYYSEADASHCIQQILESVN HCHMNNVVHRDLKPENLLLA SKAKGAAVKLADFGLAIEVQ

Eaff RKLQHPNIVRLHDSIQEESF HYLIFDLVTGGELFEDIVAR EFYSEADASHCIQQILESVN HCHSNGVVHRDLKPENLLLA SKSKGAAVKLADFGLAIEVQ

Lcyp RKLQHPNIVRLHDSIQEESF HYLVFDLVTGGELFEDIVAR EFYSEADASHCIQQILESVN HCHSNGVVHRDLKPENLLLA SKQKGAAVKLADFGLAIEVQ

Lsal -------------------- -------------------- -------------------- -------------------- --------------------

Aamp -------------------- -------------------- -------------------- -------------------- --------------------

Afra RKLQHPNIVRLHDSIQEESF HYLVFDLVTGGELFEDIVAR EFYSEADASHCIQQILESVN HCHVNGVVHRDLKPENLLLA SKLKGAAVKLADFGLAIEVQ

Hazt -------------------- -------------------- -------------------- -------------------- --------------------

Meda -------------------- -------------------- ---------------LESVN HCHMNGVVHRDFKPENXLLA SKQKGAAVKLADFGXAIEVQ

Afos -------------------- -------------------- -------------------- -------------------- --------------------

Dmel RKLHHPNIVRLHDSIQEENY HYLVFDLVTGGELFEDIVAR EFYSEADASHCIQQILESVN HCHQNGVVHRDLKPENLLLA SKAKGAAVKLADFGLAIEVQ

Tcas RKLQHPNIVRLHDSIQEENF HYLVFDLVTGGELFEDIVAR EFYSEADASHCIQQILESVN HCHQNGVVHRDLKPENLLLA SKAKGAAVKLADFGLAIEVQ

Pmon -------------------- -------------------- -------------------- -------------------- --------------------

Smar RKLQHPNIVRLHDSIQEESF HYLVFDLVTGGELFEDIVAR EFYSEADASHCIQQILESVN HCHQNCVVHRDLKPENLLLA SKAKGAAVKLADFGLAIEVQ

Tcal RKLQHPNIVRLHDSIQEENF HYLVFDLVTGGELFEDIVAR EFYSEADASHCIQQILESVN HCHQNGVVHRDLKPENLLLA SKQKGAAVKLADFGLAIEVQ

Crog RKLQHPNIVRLHDSIQEENF HYLVFDLVTGGELFEDIVAR EFYSEADASHCIQQILESVN HCHCNGVVHRDLKPENLLLA SKLKGDS--------AIEVQ

Dpul RKLQHPNIVRLHDSIQEESF HYLVFDLVTGGELFEDIVAR EFYSEADASHCIQQILESVN HCHQNGVVHRDLKPENLLLA SKAKGAAVKLADFGLAIEVQ

Aver GEQQAWFGFAGTPGYLSPEV LKKEPYGKPVDIWACGVILY ILLVGYPP------------ -------------------- --------------------

Csin -------------------- -------------------- -------------------- -------------------- --------------------

Isca GEQQAWYGFAGTPGYLSPEV LKKDPYGKPVDIWACGVILY ILLVGYPPFWDEDQHRLYMQ IKAGAYDYPSPEWDTVTPEA KNLINSMLTVNPAKRITAAE

Eaff GDQQAWFGFAGTPGYLSPEV LKKEPYGKPVDIWACGVILY ILLVGYPPFWDEDQHRLYAQ IKAGAYDYPSPEWDTVTPEA KNLINQMLTVNPAKRIRAEE

Lcyp GDQQAWFGFAGTPGYLSPEV LMKEPYGKPVDIWACGVILY ILLVGYPPFWDEDQHRLYAQ IKAGAYDYPSPEWDTVTPEA KNLINQMLTVNPAKRIRSDV

Lsal -------------------- -------------------- -------------------- -------------------- --------------------

Aamp -------------------- -------------------- -------------------- -------------------- --------------------

Afra GEQQAWFGFAGTPGYLSPEV LKKEPYGKPVDIWACGVILY ILLVGYPPFWDEDQHRLYAQ IKAGAYDYPSPEWDTVTPEA KNLINQMLTVNPAKRITSTE

Hazt -------------------- -------------------- -------------------- -------------------- --------------------

Meda GEQQAWFGFAGTPGYLSPEV LKKEPYGKPVDIWACGVILY ILLVGYPP------------ -------------------- --------------------

Afos -------------------- -------------------- -------------------- -------------------- --------------------

Dmel GDHQAWFGFAGTPGYLSPEV LKKEPYGKSVDIWACGVILY ILLVGYPPFWDEDQHRLYSQ IKAGAYDYPSPEWDTVTPEA KNLINQMLTVNPNKRITAAE

Tcas GEQQAWFGFAGTPGYLSPEV LKKEPYGKPVDIWACGVILY ILLVGYPPFWDEDQHRLYAQ IKAGAYDYPSPEWDTVTPEA KNLINQMLTVNPGKRITASE

Pmon -------------------- -------------------- -------------------- -------------------- --------------------

Smar ADQQGWFGFAGTPGYLSPEV LKKEPYGKPVDIWACGVILY ILLVGYPPFWDEDQHRLYAQ IKAGAYDYPSPEWDTVTPEA KNLINSMLTVNPAKRIVAAD

Tcal GDQQAWFGFAGTPGYLSPEV LKKEPYGKAVDIWACGVILY ILLVGYPPFWDEDQHRLYAQ IKAGAYDYPSPEWDTVTPEA KNLINQMLTVNPQKRIKSEE

Crog GDQHAWFGFAGTPGYLSPEV LKKEPYGKAVDIWACGVILY ILLVGYPPFWDEDQHRLYAQ IKAGAYDYPSPEWDTVTPEA KNLINQMLTVNPAKRIRSDE

Dpul GEQQAWFGFAGTPGYLSPEV LKKEPYGKPVDIWACGVILY ILLVGYPPFWDEDQHRLYAQ IKAGAYDYPSPEWDTVTPEA KNLINQMLTVNPAKRITAAE

Aver -------------------- -------------------- -------------------- -------------------- --------------------

Csin -------------------- -------------------- -------------------- -------------------- --------------------

Isca ALKHPWICQRERVASTLHRQ ETVDCLKKFNARRKLKGAIL TTMLAT-RNFS-S------- -KGEGSQVKESTD-SSTTIE DD--DVK-------------

Eaff ALKHPWICQRERVASVFHRQ ETVDCLKKFNARRKLKGAIL TTMLVS-RNFS-TKSLVNK- -GSK---------------- --------------------

Lcyp ALKHPWICQRERVASMVHRQ ETVDCLKKFNARRKLKGAIL TTMLVS-RNFS-TRSMVGAG VKGKSGDVKESTD-SSTTIE EDEKDVKMNHV------RGS

Lsal -------------------- -------------------- -------------------- -------------------- --------------------

Aamp -------------------- -------------------- -------------------- -------------------- --------------------

Afra ALKHPWICQRERVASVVHRQ ETVDCLKKFNARRKLKGAIL TTMLAT-RNFS-SKSIIK-- -KESGSQVKESSD-SNTTLE DD--DSKEEKAKGV--DRSS

Hazt -------------------- -------------------- -------------------- -------------------- --------------------

Meda -------------------- -------------------- -------------------- -------------------- --------------------

Afos -------------------- -------------------- -------------------- -------------------- --------------------

Dmel ALKHPWICQRERVASVVHRQ ETVDCLKKFNARRKLKGAIL TTMLAT-RNFS-SRSMITK- -KGEGSQVKESTDSSSTTLE DD--DIK-------------

Tcas ALKHPWICQRERVASVVHRQ ETVDCLKKFNARRKLKGAIL TTMLAT-RNFS-SRSIIVK- -KGDGSQVKESTD-SSTTLE DD--DIK-------------

Pmon -------------------- -------------------- -------------------- -------------------- --------------------

Smar ALKHPWICQRERVASVVHRQ ETVDCLKKFNARRKLKGAIL TTMLAT-RNFS-TRSIVNK- -KGDGSQVKESTD-SSTTIE ED--DVKDASVMHLAPDRSK

Tcal ALKHPWICNRDRVASVVHRQ ETVDCLKKFNARRKLKGAIL TTMLVS-RNFS-SKGA---- -------------------- --------------------

Crog ALKHPWICQRERVASVVHRQ ETVDCLKKFNARRKLKGAIL TTMLVS-RNFS-TRSMASKG -KSDGSQVKESTD-SSTTIE DD--DKDEKKVV----DRSS

Dpul ALKHPWICQRERVASVVHRQ ETVDCLKKFNARRKLKGAIL TTMLAT-RNFS-SRSIINK- -KSDGSNVKESTD-SSTTIE ED--DVKEKSKGVV--DRSS

Aver -------------------- -------------------- -------------------- -------------------- --------------------

Csin -------------------- -------------------- -------------------- -------------------- --------------------

Isca -------------------- ----------------ARKQ EIVKLTEQLIESINGGDYET YTKLCDPHLTAFEPEALGNL VEGMEFHKFYFDNGAGKS--

Eaff -------------------- -------------------- -------------------- -------------------- --------------------

Lcyp SG------------------ AVQIRTSGSQSDPSSIARKS EIIRSTELLLEAINTSDFES YARLCDPHLTAFEPEALGNL VEGLEFHKFYFDN-LKSQ--

Lsal -------------------- -------------------- -------------------- -------------------- --------------------

Aamp -------------------- -------------------- -------------------- -------------------- --------------------

Afra TVIAKDPD------------ ----------------ARKQ EVLRMTEQLVEAISCGDFEN YTKLCDPHLTAFEPEALGNL VQGMEFHKFYFDNVLGKN--

Hazt -------------------- -------------------- -------------------- -------------------- --------------------

Meda -------------------- -------------------- -------------------- -------------------- --------------------

Afos -------------------- -------------------- -------------------- -------------------- --------------------

Dmel -------------------- ---------------AARRQ EIIKITEQLIEAINSGDFDG YTKICDPHLTAFEPEALGNL VEGIDFHKFYFENVLGKN--

Tcas -------------------- ----------------ARRQ EIIKMTEQLIEAINTGDFEA YTKICDPHLTAFEPEAMGNL VEGMDFHKFYFDNVLGKN--

Pmon -------------------- -------------------- -------------------- -------------------- --------------------

Smar TVIAREPEEDRYSLHIMAQC DAIAEAVKNDSFISLSPRKQ EIVKITEQLIDAINTGDYEA YTKICDPHITSFEPESLGNL VEGMDFHKFYFENVLGKN--

Tcal -------------------- -------------------- -------------------- -------------------- --------FLFEM-------

Crog TVIARDTAEDMKLNHVKNSS SLQIRNSNSQGDPSVSARKN EIIRCTELLLEAICSGDFES FTRLSDSSLTTFDPEALGNC VEGLEFHKFYFDNVLGKNDE

Dpul TVIAKEPEDVR-------LT ATAKTCSQLPSNWNGSARKQ EILKLTEQLLEAISAGDYET YAKICDPHVTSFEPEALGNL VEGLEFHKFFFDNLLGKN--

Aver -------------------- -------------------- -------------------- -------------------- --------------------

Csin -------------------- -------------------- -------------------- -------------------- --------------------

Isca YKSQNTTIINPTVHLLGDDA ACIAYIRLMQYIDRAGIART RQTEETRVWHRKDTKWQNVH FHRSGNS---SGMHPDPAAK --------------------

Eaff -------------------- -------------------- -------------------- -------------------- -----------MNPHSGLPF

Lcyp RHISNINIINPHVHLLGDDA AAIAYVRMTQFIDKQGSPQT QQSEETRIWHRRDGRWINVH FHRSTLA---VDSPFASKTQ -----------INPYTSQPY

Lsal -------------------- -------------------- -------------------- -------------------- -----------MNPHSALPY

Aamp -------------------- -------------------- -------------------- -------------------- --------------------

Afra CKAVNTLILNPHVHLLGEDA ACIAYVRLTQFIDKQGQAHT QQSEETRVWQRKDSRWQHVH FHRSGCP---S---FTLGHK -----------INPYNGQVY

Hazt -------------------- -------------------- -------------------- -------------------- --------------------

Meda -------------------- -------------------- -------------------- -------------------- --------------------

Afos -------------------- -------------------- -------------------- -------------------- -----------MNPHTGLPF

Dmel CKAINTTILNPHVHLLGEEA ACIAYVRLTQYIDKQGHAHT HQSEETRVWHKRDNKWQNVH FHRSASAKISGATTFDFIPQ ----------KMNPLTNTPY

Tcas CKAVNTTILNPHVHLLGEDA ACIAYVRLTQYMDKHGQAHT HQSEESRVWHKRDNKWQNVH FHRSGGT---GGAAFGFSSH ----------KINPHTGLPY

Pmon -------------------- -------------------- -------------------- -------------------- --------------------

Smar CKAINTTLLNPHVHLMGDDA ACIAYVRLIQYMDKTGGAYT QQSEETRVWHRRDGKWQHVH FHRSRSP---ANIFSTVAAT ----------KLNPFTTLPY

Tcal -------------------- -------------------- -------------------- -------------------- -----------LNPHTGLPF

Crog SRTLNVNIINPLVHLLGEDS AAIAYVRLTQSIDKSGKIST SQCEETRIWYRRETRWLNVH VHRSNNC---SSSSSISSST AINKSISTNKSMNPHTSLPY

Dpul CKSINTLILNPHIHLMGEDA ACIAYVRLTQFVDKQGQAHT QQNEETRVWYRRDGKWLNVH FHRSGAS---TPCTLPYHQN ----------KINPLTGLPY

Aver -------------------- -------------------- -------------------- -------------------- --------------------

Csin -------------------- -------------------- -------------------- -------------------- --------------------

Isca -------------------- -------------------- -------------------- -------------------- --------------------

Eaff SPRYFELFRKRCQLPVWDYQ EKFLDLLARHQNICLVGETG SGKTTQIPQWCVDFSKSMG- KKTVACTQPRRVAAMSVAQR VAEEMDVSLGQEVGYSIRFE

Lcyp TPRYFELFRKRIGLPVWEYQ EKFLELLNKHQIICLVGETG SGKTTQIPQWCVDYARKLG- TKRVSCTQPRRVAAMSVAQR VSEEMDVPLGQEVGYSIRFE

Lsal TPRYFELFRKRITLPVWEYR DKFLDLLGQHQCICLVGETG SGKTTQIPQWCAEYA-SKE- RKSVACTQPRRVAAMSVAQR --------------------

Aamp -------------------- -------------------- -------------------- -------------------- --------------------

Afra TPRYYGLFRKRIGLPVWEYR EKFFDLLNKYQTIVLVGETG SGKTTQIPQWCVEFAKQKGV KKGVACTQPRRVAAMSVAQR VSEEMDVVLGQEVGYSIRFE

Hazt -------------------- -------------------- -------------------- -------------------- --------------------

Meda -------------------- -------------------- -------------------- -------------------- --------------------

Afos SPRYFEIFRKRCQLPVWDYQ EKFLDLLARHQSICLVGETG SGKTTQIPQWCVDFSKKMG- KKGVACTQPRRVAAMSVAQR VAEEMDVALGQEVGYSIRFE

Dmel SQRYQNLYKKRIALPVFEYQ ADFMRLLSLHQCIVLVGETG SGKTTQIPQWCVDFAVSKG- RKGVSCTQPRRVAAMSVAQR VSEEMDVKLGEEVGYSIRFE

Tcas TNKYHELYRKRITLPVFEYR NDFMRLLAENQCIVLVGETG SGKTTQIPQWCVEFARSVG- KKGVCCTQPRRVAAMSVAQR VSEEMDVALGQEVGYSIRFE

Pmon -------------------- -------------------- -------------------- -------------------- --------------------

Smar TPRYNELYRKRINLPVWEYR EDFLEIINTNRIMVLVGETG SGKTTQIPQWCVEFVRSRG- KRGVACTQPRRVAAMSVAQR VSEEMDCQLGQEVGYSIRFE

Tcal TPRYHEIFKKRKALPVWEYQ DKFLDLLQKHQILCLVGETG SGKTTQIPQWCVDFSKKVG- KKCCACTQPRRVAAMSVAQR VAEEMDVSLGQEVGYSIRFE

Crog TPRYFELFRKRISLPVWEYR EKFMDLLSSHQCICLVGETG SGKTTQIPQWCAEYA-ARE- SKSVACTQPRRVAAMSVAQR VSEEMDVSLGQEVGYSIRFE

Dpul TQKYYDIYRKRIGLPVWEYR EKFFELLENNQAIVLVGETG SGKTTQIPQWSAEFARKLGV KKGVACTQPRRVAAMSVAQR VADEMDVVLGQEVGYSIRFE

Aver -------------------- -------------------- -------------------- ------------------YF DNAPLMNVPGRTHPVEIFYT

Csin -------------------- -------------------- -------------------- -------------------- --------------------

Isca -------------------- -------------------- -------------------- -------------------- --------------------

Eaff DCSGPRTLLKYMTDGMLLRE AMSDPMLENYQVILLDEAHE RTLATDILMGVLKTVVTHRP DLKLVIMSATLDAGKFQGYF DNAPLMNVPGRTHPVEIFYT

Lcyp DCSDQKTLLKYMTDGMLLRE AMSDPMLEAYQVILLDEAHE RTLATDILMGVLKTVTQHRS DLKLVIMSATLDAGKFQNYF DNAPLMNIPGRTHPVEIFYT

Lsal -------------------- -------------------- -------------------- -------------------- --------------------

Aamp -------------------- -------------------- -------------------- -------------------- --------XGRTHPVEIFYT

Afra DCSSPRTLLKYMTDGMLLRE GMSDPMLENYQVVLLDEAHE RTLATDILMGVLKEILKQRT DLKLVVMSATLDAGKFQNYF DGAPLLNVPGRTHPVEIYYT

Hazt -------------------- -------------------- -------------------- -------------------- --------------------

Meda -------------------- -------------------- -------------------- -----------------NYF DNAPLMNVPGRTHPVEIFYT

Afos DCSGPRTLLKYMTDGMLLRE AMSDPMLENYQVILLDEAHE RTLATDILMGVLKTVITHRP DLKLVIMSATLDAGKFQSYF DNAPLMNVPGRTHPVEIFYT

Dmel DCSTAKTLLKYMTDGMLLRE AMSDPMLDQYQVILLDEAHE RTLATDILMGVLKEVIRQRS DLKLVVMSATLDAGKFQQYF DNAPLMKVPGRTHPVEIFYT

Tcas DCSSAKTILKYMTDGMLLRE GMSDPMLDAYQCILLDEAHE RTLATDILMGVLKEVIKQRS DLKLVIMSATLDAGKFQQYF DNAPLMNVPGRTHPVEIFYT

Pmon -------------------- -------------------- -------------------- -------------------- --------------------

Smar DCSSQKTILKYMTDGMLLRE AMSDPLLEAYGVILLDEAHE RTLATDILMGVLKEVVKQRP DLKIIIMSATLDAGKFQHYF DNAPLMNVPGRTHPVEIFYT

Tcal DCSGPKTLLKYMTDGMLLRE AMSDPMLENYQVILLDEAHE RTLSTDILMGVLKTVIGHRP DLKLVIMSATLDAGKFQNYF DNAPLMNVPGRTHPVEIFYT

Crog DCSGPRTILKYMTDGMLLRE AMSDTMLDHYKVILLDEAHE RTLATDILMGVLKTVIGHRN DLKLVIMSATLDAGKFQNYF DNAPLMNVPGRTHPVEIFYT

Dpul DCSSPKTVLKYMTDGMLLRE AMSDPLLDSYQLVLLDEAHE RTLATDILMGVLKEVIKQRR DLKLIIMSATLDAGKFQSYF DNAPLMNVPGRTHPVEIFYT

Aver PEPERDYLEAAIRTVIQIHM CEEDDGDILLFLTGQEEIEE ACKRIKREIDNLGPEVGDMK CIPLYSTLPPNLQQRIFEAA PSRKANGAIGRKVVVSTNIA

Csin -------------------- -------------------- -------------------- -------------------- -----------KIVVSTYIA

Isca -------------------- -------------------- -------------------- -------------------- --------------------

Eaff PEPERDYLEAAIRTVVQIHM CEEVDGDLLLFLTGQEEIEE ACKRLKREIDNLGPDVGDMK CIPLYSTLPPNLQQRIFEPP PGKKANGAIGRKIVVSTNIA

Lcyp PEPERDYLEAAIRTVIQIHM CEEDDGDILLFLTGQEEIEE ACKRIKREIDNLGPEVGDLK CIPLYSTLPPNLQQRIFEPA PARKANGAAGRKVVVSTNIA

Lsal -------------------- -------------------- -------------------- -------------------- --------------------

Aamp PEPERDYLEAAIRTVVQIHM CEEVEGDMLLFLTGQEEIEE ACKRLKREIDNVGPEIGELK CIPLYSTLPPALQQRIFEPA PANRPNGAVGRKVVVSTNIA

Afra PEPERDYLEAAIRTVIQIHM CEEIAGDVLLFLTGQEEIEE ACKRLKREIDNLGNEVGDLK CIPLYSTLPPNLQQRIFEPP PPNKASGAIGRKVVVSTNIA

Hazt -------------------- -------------------- -------------------- -------------------- --------------------

Meda PEPERDYLEAAIRTVIQIHM CEEXXGDILLFLTGQEEIEE ACKRIKXEIDNXGXEVGDMK CIPXYSTLPPNLQQRIFEXA PXRKSNGAIGRKVVVSTNIA

Afos PEPERDYLEAAIRTVIQIHM CEETDGDMLLFLTGQEEIEE ACKRLKREIDNLGPEVGDLK CIPLYSTLPPNLQQRIFEAP PGKKANGAIGRKVVVSTNIA

Dmel PEPERDYLEAAIRTVIQIHM CEEIEGDILMFLTGQEEIEE ACKRIKREIDNLGSEIGELK CIPLYSTLPPNLQQRIFEPA PPPNANGAIGRKVVVSTNIA

Tcas PEPERDYLEAAIRTVIQIHM CEEIAGDILLFLTGQEEIEV ACKRIKREIDNLGPEVGELK CIPLYSTLPPNLQQRIFEEA PPNKANGAIGRKVVVSTNIA

Pmon -------------------- -------------------- -------------------- -------------------- --------------------

Smar PEPERDYLEAAIRTVIQIHM CEETEGDILLFLTGQEEIEE ACKRIKREVDNLGPEIGELK CIPLYSTLPPNLQQRIFEPA PPRKATGGIGRKVVVSTNIA

Tcal PEPERDYLEAAIRTVIQIHM CEEEAGDILMFLTGQEEIEE ACKRIKREIDNLGPDAGDLK CIPLYSTLPPNLQQRIFEAA PPRKSNGAIGRKVVVSTNIA

Crog PEPERDYLEAAIRTVIQIHM CEEQEGDALLFLTGQEEIEE ACKRIKREIDNMGNEVGDLK CIPLYSTLPPNLQQRIFEPA PPKKANGAIGRKIVISTNIA

Dpul PEPERDYLEAAIRTVMQIHM CEDIVGDVLLFLTGQEEIDE ACKRLKREIDNLGPEVGEMK CIPLYSTLPPNLQQRIFEAA PPVRPNGAIGRKVVISTNIA

Aver ETSLTIDGVVFVIDPGFSKQ KVYNPRIRVESLLVSPISKA SAQQRAGRAGRTKPGKCFRL YTEKAYK------------- --------------------

Csin ETSLTIDGVVFVIDPGISKQ KVYNLRIRVEHLLVFPISKA SAQQRAGRAGRTKPGKCFRL YTEKAYKNEMQDNTYPEILR SNLGAVVIQLKKLGIDDLVH

Isca -------------------- -------------------- -------------------- -------------------- --------------------

Eaff ETSLTIDGVVFVIDPGFSKQ KVYNPRIRVESLLVSPISKA SAQQRAGRAGRTKPGKCFRL YTEKAYKNEMQDNTYPEILR SNLGAVVIQLKKLGIDDLVH

Lcyp ETSLTIDGVVFVIDPGFSKQ KVYNPRIRVESLLVSPISKA SAQQRAGRAGRTKPGKCFRL YTEKAYKNEMQDNTYPEILR SNLGSVVLQLKKLGIDDLVH

Lsal -------------------- -------------------- -------RAGRTKPGKCFRL YTEKAFKNEMQENTYPEILR SNLGSVVIQLKKLGIDDLVH

Aamp ETSLTIDGVVFVIDPGFSKQ KVYNPRIRVESLLVSPISKA SAQQRAGRAGRTKPGKCFRL YTEKAFKSEMQDNTYPEILR SNLGTVVLQLKKLGIDDLVH

Afra ETSLTIDGVVFVIDPGFAKQ KVYNPRIRVESLLVSPISKA SAQQRAGRAGRTRPGKCFRL YTEKAYKQEMQDNTYPEILR SNLGSVVLQLKKLGIDDLVH

Hazt -------------------- -------------------- -------------------- -------------------- --------------------

Meda ETSXTIDGVVFVIDPGFSKQ KVYNPRIRVESLLVSPIXKA SAQQXAGRAGRTKPGKCFRL YTEKAYKN------------ --------------------

Afos ETSLTIDGVVFVIDPGFSKQ KVYNPRIRVESLLVSPISKA SAQQRAGRAGRTKPGKCFRL YTEKAYKNEMQDNTYPEILR SNLGAVVIQLKKLGIDDLVH

Dmel ETSLTIDGVVFVIDPGFAKQ KVYNPRIRVESLLVSPISKA SAQQRSGRAGRTRPGKCFRL YTEKAFKNEMQDNTYPEILR SNLGTVVLQLKKLGIDDLVH

Tcas ETSLTIDGVVFVIDPGFAKQ KVYNPRIRVESLLVSPISKA SAQQRAGRAGRTRPGKCFRL YTEKAYKNEMQDNTYPEILR SNLGSVVLQLKKLGIDDLVH

Pmon -------------------- -------------------- -------------------- -------------------- --------------------

Smar ETSLTIDGVVFVIDPGFSKQ KVYNPRIRVESLLVSPISKA SAQQRAGRAGRTQPGKCFRL YTEKAYKQEMQDNTYPEILR SNLGSVVLQLKKLGIDDLVH

Tcal ETSLTIDGVVFVIDPGFSKQ KVYNPRIRVESLLVSPISKA SAQQRAGRAGRTKPGKCFRL YTEKAFKNEMQENTYPEILR SNLGSVVLQLKKLGIDDLVH

Crog ETSLTIDGVVFVIDPGFSKQ KVYNPRIRVESLLVSPISKA SAQQRAGRAGRTKPGKCFRL YTEKAFKNEMQDNTYPEILR SNLGSVVLQLKKLGIDDLVH

Dpul ETSLTIDGVVFVIDPGFAKQ KVYNPRIRVESLLVSPISKA SAQQRAGRAGRTRPGKCFRL YTEKAYKQEMQDNTYPEILR SNLGSVVINLKKLGIDDLVH

Aver -------------------- -------------------- -------------------- -------------------- --------------------

Csin FDFMDPPAPETLMRALELLN YLAALDDDGNLTELGAIMAE FPLDPQLAKMLIASCEYQCS NEILSIVAMLS--------- -VPQCFVRPNEAKKASDDSK

Isca -------------------- -------------------- -------------------- -------------------- --------------------

Eaff FDFMDPPAPETLMRALELLN YLAALDDDGNLTELGAIMAE FPLDPQLAKMLIASCEYNCS NEILSIVSMLS--------- -VPQCFVRPNEAKKASDDSK

Lcyp FDFMDPPAPETLMRALELLN YLAALDDDGNLTELGAIMAE FPLDPQLAKMLIASCEYNCS NEILSITAMLS--------- -VPQCFVRPNEAKKAADDSK

Lsal FDFMDPPAPETLMRALELLN YLAALDDDGNLTELGAIMAE FPLDPQLAKMLIASCENNCS NEILSITAMLS--------- -VPQCFVRPNDAKKAADDSK

Aamp FDFMDPPAPETLMRALELLN YLSALDDSGNLTDLGAIMAE FPLDPQLAKMLIASTEYNCS NEILSITAMLS--------- -VPQCFVRPNDARKAADESK

Afra FDFMDPPAPETLMRALELLN YLAALDDDGNLTELGAIMAE FPLDPQLAKMLIASCEFNCS NEILSITAMLS--------- -VPQCFVRPNEAKKAADDAK

Hazt -------------------- -------------------- -------------------- -------------------- --------------------

Meda -------------------- -------------------- -------------------- -------------------- --------------------

Afos FDFMDPPAPETLMRALELLN YLAALDDDGNLTELGAIMAE FPLDPQLAKMLIASCEYNCS NEILSITSMLS--------- -VPQCFVRPNEAKKASDDSK

Dmel FDFMDPPAPETLMRALELLN YLAALDDDGNLTDLGAVMSE FPLDPQLAKMLIASCQHNCS NEILSITAMLS--------- -VPQCFVRPNEAKKAADEAK

Tcas FDFMDPPAPETLMRALELLN YLAALDDDGNLTDLGAVMAE FPLDPQLAKMLIASCNHNCS NEILSITAMLS--------- -VPQCFIRPNEAKKAADDAK

Pmon -------------------- -------------------- -------------------- -------------------- --------------------

Smar FDFMDPPAPETLMRALELLN YLASLDDDGNLTELGSIMAE FPLDPQLAKMLISSCDYNCS NEILSITAMLS--------- -VPQCFVRPNEAKKAADEAK

Tcal FDFMDPPAPETLMRALELLN YLAALDDDGNLTELGAIMAE FPLDPQLAKMLIASCENNCS NEILSVTAMLS--------- -VPQCFVRPNEAKKAADDSK

Crog FDFMDPPAPETLMRALELLN YLAALDDDGNLTDLGAIMAE FPLDPQLAKMLIASCENNCS NEILSITAMLS--------- -VPQCFVRPNDAKKAADDSK

Dpul FDFMDPPAPETLMRALELLN YLGALDDDGNMTELGAIMAE FPLDPQLAKMLIASTEFNCS NEILSITAMLS--------- -VPQVFMRPLEAKKAADEAK

Aver -------------------- -------------------- -------------------- -------------------- --------------------

Csin LRFA----HIDGDHLTLLNV YHAFKQNMEDPQWCYDNFVN YRSLKSSDNVRQQLCRIMDR FNLKRTSTDFTSKDYYMKV- --------------------

Isca -------------------- -------------------- -------------------- -------------------- --------------------

Eaff LRFA----HIDGDHLTLLNV YHAFKQNMEDPQWCYDNFVN YRSLKSADNVRQQLCRIMDR FNLKRTSTDFTSKDYYLNIR KALCSGFFMQVAHLERTGHY

Lcyp LRFA----HIDGDHLTLLNV YHAFKQNMEDPQWCYDNFVN YRSLKSADNVRQQLCRIMDR FYLKRTSTEFTSKDYYINIR KALCSGFFMQVAHLERTGHY

Lsal LRFA----HIDGDHLTLLNV YHAFKQNMDDPQWCYDNFV- ------ADNVRQQLCRIMDR FNLKRTSTDFASKDYYVNIR KALCAGFFMQVAHLERSGHY

Aamp MRFA----HIDGDHLTLLNV YHAFKQNMEDPHWCHDNFVN YRSLKSADNVRQQLSRIMDR FNMPRRSTDFTSRDYYINIR KALVSGFFMQVAHLERTGHY

Afra MRFA----HIDGDHLTLLNV YHAFKQNMEDPQWCFENFIN YRSLKSSDNVRAQLSRIMDR FGLKRSSTEFTSRDYYINIR KALVSGFFMQVAHLERSGTY

Hazt -------------------- -------------------- -------------------- -------------------- --------------------

Meda -------------------- -------------------- -------------------- -------------------- --------------------

Afos LRFA----HIDGDHLTLLNV YHAFKQNMEDPQWCYDNFVN YRSLKSADNVRQQLCRIMDR FNLKRTSTDFTSKDYYINIR KALCSGFFMQVAHLERTGHY

Dmel MRFA----HIDGDHLTLLNV YHAFKQSSEDPNWCYENFIN FRSLKSADNVRQQLARIMDR FNLRRTSTEFTSKDYYVNIR KALVQGFFMQVAHLERTGYY

Tcas MRFA----HIDGDHLTLLNV YHAFKQSMEDPQWCYDNFVN YRSLKSADNVRQQLSRIMDR FNLKRTSTDFTSKDYYINIR KALVNGFFMQVAHLERTGHY

Pmon -------------------- -------------------- -------------------- -------------------- --------------------

Smar MRFA----HIDGDHLTLLNV YHAFKQNHEDPQWCYDNFVN YRSLKSADNVRQQLARIMDR FNLRRTSTDFTSKDYYINIR KVLVSGFFMQVAHLERTGHY

Tcal LRFA----HIDGDHLTQLNV YHAFKQNMEDPQWCYDNFVN YRSLKSADNVRQQLCRIMDR FNLKRTSTEFTSKDYYINIR KALCAGFFMQVAHLERTGHY

Crog LRFA----HIDGDHLTLLNV YHAFKQNMEDPQWCYDNFVN YRSLKSADNVRQQLCRIMDR FNLKRTSTNFASKDYYINIR KALCAGFFMQVAHLERSGHY

Dpul MRFA----HIDGDHLTMLNV YHAFKQNMEDPQWCYDNFVN YRSMKSADNVRQQLSRIMDR FNLKRTSTEFTSKDYYVNIR KALISGFFMQVAHLERTGHY

Aver -------------------- -------------------- -------------------- -------------------- --------------------

Csin -------------------- -------------------- -------------------- -------------------- --------------------

Isca -------------------- -------------------- -------------------- -------------------- ------MADD-EYEADDVGG

Eaff LTIKDNQVVQLHPTTCLDHK PEWILYNEFVLTTKNYIRTC TDVKPEWLVRVAPQYYDMNN FPQCEAKRQLEQIIAKINSR QYQKGFMADD-EYEPDFD--

Lcyp LTIKDNQIVQLHPSTCLDHK PEWVLYNEFVLTTKNYIRTV TDVKPEWLIKTAPQYYDMDN FPQCEAKRQLEQIITKVNSR QYQRGFM-AD-DYEDNDF--

Lsal LTVKDNQIVQLHPSTCLDHK PEWVLYNEFVLTTKNYIRTV TDVKPDWLIKAAPQYYDMNN FPQCEAKRQLEQVIQRINSR QYQKGFM-GD-EYEENDF--

Aamp LTCKDNQVVQLHPSTCLDHK PEWVLYNEFVLTTKNYIRTV TDIKAEWLVKIAPQYYDMAN FPQCEAKRQLELLIAKLESK QFQQGY--------------

Afra LTIKDNQVVQLHPSTCLDHK PEWVLYNEFVLTTKNYIRTI TDVKPEWLLKIATQYYDMSN FPQGEARRQLEAILAKLESK QYKEGF--------------

Hazt -------------------- -------------------- -------------------- -------------------- --------------------

Meda -------------------- -------------------- -------------------- -------------------- --------------------

Afos LTIKDNQVVQLHPTTCLDHK PEWVLYNEFVLTTKNYIRTC TDVKPEWLVRVAPQYYDMNN FPQCEAKRQLEQIIAKINSR QYQKGFMADD-EYEPDYD--

Dmel LTIKDNQNVQLHPSTCLDHK PDWVIYNEFVLTTKNYIRTV TDVKPEWLCCLAPQYYDLNN FPQCEAKRQLELLQQRLETK QYQKGFMDDA-DYDNDDV-G

Tcas LTIKDNQNVQLHPSTCLDHK PEWVIYNEFVLTTKNYIRTV TDIKPDWLIKIAPQYYDLQN FPQCEAKRQLEIIQNRLDSK QYQHGFMNDE-EFDGDDV--

Pmon -------------------- -------------------- -------------------- -------------------- ------MADE-EFDGDDV--

Smar LTIKDNQVVQLHPSTCLDHR PEWVVYNEFVLTTKNYIRTV TDIKPEWLIKLAPQYYEMSN FPQCEAKRQLELIADKLESQ QYKEGFM-DD-DYEPDDG--

Tcal LTVKDNQIVQLHPSTCLDHK PEWVLYNEFVLTTKNYIRTV TDVKPDWLVKTAPQYYDMDN FPTCEAKRQLEQIIARVNSR QYKKGFM-GD-EYDNDND-F

Crog LTVKDNQIVQLHPSTCLDHK PEWVLYNEFVLTTKNYIRTV TDVKPDWLIKAAPQYYDMNN FPQCEAKRQLEQVIQRVNSR QYQKGFM-GD-EYEENDF--

Dpul QTIKDNQVVQLHPSTCLDHK PEWVIYNEFVLTTKNYIRTC TDVKPEWLIKVAPSYYDMSN FPEGPAKRQLEQIINRLESR EYRN--M-------------

Aver -------------------- -------------------- -------------------- -------------------- --------------------

Csin -------------------- -------------------- -------------------- -------------------- --------------------

Isca GD-DF--DDVDEDENLD--- EIDQ------NDDENIELLQ ASD--------GQPQQNQKR ITTMYMTKYERARVLGTRAL QIAMCAPVMVELEGETDPLQ

Eaff DN-DFVGDDIDGEDIDD--- AVDD--AD--AGEGNIDILS AGQ-------EGAGQKTTKR ITTPYLTKYERARVLGTRAL QISMCAPVMVELEGETDPLQ

Lcyp DD-DY----GDADDADDMMD EMEE-GAEQGAEQEHVEILH AGE----DGISGEGVNSPNR TTTRYMTKYERARVLGTRAL QIAMCAPVMVELEGETDPLE

Lsal DD-DY----QDVDEPDEMC- NLDD---D--ADADNIDVLP TNEAD-NDPSSGDSKSKEKR TTTPYMTKYERARVLGTRAL QIAMCAPVMVELEGETDPLQ

Aamp -------------------- -------------------- -------------------- -------------------- --------------------

Afra -------------------- -------------------- -------------------- -------------------- --------------------

Hazt -------------------- -------------------- -------------------- -------------------- --------------------

Meda -------------------- -------------------- -------------------- -------------------- --------------------

Afos DN-DFGGDDIDGEDLDE--- AADE--TA--GPEGGIDVLP AGE------GGAEGQKQTKR ITTPYLTKYERARVLGTRAL QIAMCAPVMVELEGETDPLQ

Dmel GD-DF--DDVD-EDVDE--- DINQ---E--EEADNIEIIA PGG------AGGGGVPKSKR ITTKYMTKYERARVLGTRAL QIAMCAPIMVELDGETDPLQ

Tcas GD-DF-DDDVD-DDNIE--- ELEQ--PE--EEGDNIDILA HGQ-------AGGGVPKNKR ITTKYMTKYERARVLGTRAL QIAMCAPVMVELDGETDPLQ

Pmon GD-DF--EEAEEDENLD--- DLQE--GE--EDENQMEILA AGE--------GNQVPTQKR ITTPYMTKYERARVLGTRAL QIAMCAPVMVEIEAESDPLQ

Smar PE-EF--DDVEDDDNLD--- EIDQ--AE--DDGDNIDLLP MNE-------ASHSQQPNKR ITTPFITKYERARVLGTRAL QIAMCAPVMVELEGETDPLQ

Tcal EE-DY----QEADEPEELE- GMNDEEAD--GDQENVEVLP AGE------GTGEGQKSAKR VTTPYMTKYERARVLGTRAL QIAMCAPVMVELEGETDPLQ

Crog DD-DY----QDVDEPDDMC- NLEE-------DGDNVELIP AHEADGGDPSSGDPKSKEKR TTTPYMTKYERARVLGTRAL QIAMCAPVMVELEGETDPLQ

Dpul -------------------- --EQ--PD---DDAQIDLLQ QGE-------GGAGQLQTKR ITTPYMTKYERARVLGTRAL QIAMCAPVMVELEGETDPLQ

Aver -------------------- -------------------- -------------------- -------------------- --------------------

Csin -------------------- -------------------- -------------------- -------------------- --------------------

Isca IAMKELKARKIPIIIRRYLP DGSYEDWGIDELIITD-TTS MTSVPKPLKFLKPHYKTLQE VYEKITETETKSF---CADI VSILAMTISEE-RECLKYRL

Eaff IALKELKQRKIPIIIRRYMP DGSHEDWGIDELIVID-IS- -------LKF---------- -------------------- --------------------

Lcyp IAMKELKQRKIPIIIRRYLP DGSYEDWSIDELIITDLTTS MTSVPKPLKFMIPHYNTMKE VYEKIDAKAMKSVKIQCADI LSVLSMTMSEN-NDCLKYKL

Lsal IAMKELKQRKIPIIIRRYLP DGSYEDWGIDELIIIDM--- -------------------- -------------------- --------------------

Aamp -------------------- -----------------TTS MTSVPKPLKFMRPHFETMKQ IQANLTDAKVKGT---CADV VSVLAMTMSDK-RECLKYKL

Afra -------------------- -----------------TTS MTSVPKPLKFLKPYFDSLKE IYEKIEDVMTKKL---CADI ISVLAMTNAEG-RESLKYRL

Hazt -------------------- -------------------- -------------------- -------------------- --------------------

Meda -------------------- -------------------- -------------------- -------------------- --------------------

Afos IAMKELKQRKIPIMIRRYMP DGSHEDWGIDELIYVD---- -------------------- -------------------- --------------------

Dmel IAMKELKQKKIPIIIRRYLP DHSYEDWSIDELIMVDNTTS MTSVPKPLKFMRPHYETMKT VYKHMPNEQARQL---CADI ISVLSMTMGSG-KDCLAYRF

Tcas IAMKELKQRKIPIIIRRYLP DHSYEDWGIDELIIIDHTTS MTSVPKPLKFMKPHYGTMKN VYEKIRDPNVKSV---CADV ISVLAMTMGEG-RECLKYRL

Pmon IAAKELRERKIPIIIRRYLP DGSYEDWGIDELIISDFTTS MTSVPKPLKFMRSHYQTMKD IYNNIKDNALKKF---AADI VSVLAMTMSEE-RECLKYRL

Smar IAMKELKARKIPIIIRRYLP DNSHEDWAIDELILNE-TTS MTSVPKPLKFLRPHFGTLKE VYAKITTEDTKRF---CADI VSILAMTLSDN-RECLTYRL

Tcal IAMKELKQRKIPIIVRRYMP DGSYEDWGIDEMIIVDMTTS MTSVPKPLKFLMPHYNTMKV SFEKLPPGQNRRE---CADI ISVLAMTAVGP-NECLKFKL

Crog IAMKELKQRKIPIIIRRYLP DGSYEDWGIDELIIIDMTTS MTSVPKPLKFLRPHYARIQG TYEALTPGSPQKP--YLADV LSVLSMTEDNNLRNTLRYKL

Dpul IAMKELKQKKIPIIIRRYLP DGSYEDWGIDELVIND-TTS MTSVPKPLKFLRPHYAVLKT IVEKITDPNTKAL---CADI ISVLAMTNTEG-TECLQYRL

Aver -------------------- RHLAGEXVAEWNSIG---DE P-VEGATDQEMLDLTKKDQX MKLVTQIVPSQMKHHAEAEA CDLLMEVERLDIL--XEFVD

Csin -------------------- -------------------- -------------------- -------------------- --------------------

Isca LG--SRED--IGS-WGHEYV RHLAGEISQEWSEVG---EG P------E----QEAKREAL LRLAQQIVPYHMAHNAEAEA CDLLMEIERLDLL--QAYVD

Eaff -------------------- -------------------- -------------------- -------------------- --------------------

Lcyp LGDTSLES--IDS-WGHEYV RHLAGEIVHEWNSMG---EE SVVSSDEDQAMVDEHKKDKL IDLVIQIVPSQMRHHAEAEA CDLLMEVERLDLL--EKFVD

Lsal -------------------- -------------------- -------------------- -------------------- --------------------

Aamp LG--YSND--IGD-WGHEYV RHLAGDIAAEWAEPA---DP A---EGTDEEA-TERRRARL VKLSAQLVAYLMEHNAEAEA CDLLMEVERLDLL--EQHVS

Afra LG--TPGG--LVD-WGHEYV RHISGEIVGEWDEIP---EV P------E----SEEQKKVL IELTKQIAPYQMKLNAEAEA CDLLMEIERLDML--EQYVD

Hazt -------------------- -------------------- -------------------- -------------------- --------------------

Meda -------------------- RHLAGEIVSEWHSLE---EE T-TTEGQDQEMADLSRKDQL TKLVMQIVPSQMKHHAEAEA CDLLMEVERLDVL--EQFVD

Afos -------------------- -------------------- -------------------- -------------------- ----MEVEQLDQL--IQYVD

Dmel LCD-RKQR--IGD-WGHEYV RHLSGEISAHYHDTT---GD ---------------FRVQL IELVKQIIPYNMEHNAEADA CDLLIEIDHLHLL--SDFVD

Tcas SS--KLDG--IGE-WGHEYV RHLAGEIAAEWAETD---VC ------------EDKDKDVL IELAKQIVPYNMSHNAEAEA CDLLMEIERLDLL--EVYVD

Pmon QG--QRGD--IGD-WGHEYV RHLAGEIAQEWETLG---ED ------------DDARRAEL ISEAHVIVPYHMQHNAEAEA CDLLMEMEQLELLTSQDYVD

Smar LG--SKEE--IGS-WGHEYV RHLAGEIAVEWVDLS---EK ------------DGEKRERL VQLSEEIVPYNMAHNAEAEA CDLLMEIERLDLL--EKFVD

Tcal QG--HTDGPNLDA-WGHEFI RHLSGEIVTEWAELD---ED E-----------QPELKKQI EDLVREIIPLQMKHHAEAEA CDLLMEVELLDLI--LQYVD

Crog EG--AKDA--VDNLWGHEYI RHLSKEIVAAWNDDE---EE E-----------KAVSKDIL LGLVKDILPYQMKHQAEAEA CDLLMEVEQMDLI--LDFVD

Dpul IG--SKEG--LVE-WGHEYV RHLAGEIAQEWDEIPFEGDE ------------GKAKRAQL SALVEQIIPFQMTHNAEAEA CDLLMEIEQLDML--EQHVD

Aver QAAFNRVXLYLTSCVPYVPE PENXTLLTTAXEIFRKFDRY PQAXRLALQXNDSKLIXEIF MDCKDPVMXXQLXFMLGRQQ HFXPDLEE-XE-FDEVIEXM

Csin -------------------- -------------------- -------------------- -------------------- --------------------

Isca QEAYPRVCLYLTSCVPYVPE PENTNLLKTAMEIFLKYERY PESSAAGHAAQQPRGGPEDL HTVQGP---KQMAFMLGRQQ MFLEPSDEIEN-YDDLAEIV

Eaff --CCPRVCLYLTSCVPYVPD PENTNLLVTALNIFRKFNQL PQAMRLALQLNDRDKVEDIF NTCPDSTVKRQLAFMLGRHQ FYLELPADSED-YDDLNEIM

Lcyp KAAYNRVCLYLTSCVPFVPE PENTNLLKTALQIFRKFDQN PQALRLALQLNDHELVMDIF NQCEDPTMQKQLAFMLARHQ DFSQYMDESKD-DDEAKEIM

Lsal -------------------- -------------------- -------------------- -------------------- --------------------

Aamp KDTYQRVCLYLTSCVPYVPD PENTRQLHVALQLFRKFEQY PQALRIAMQLQDQALIQEIF FDCKDKSLQKQLAYMLARQG VFVDLNE--DE-YGDLVEII

Afra ESVYQRVCLYLTSCVPYVPA PENTNLLLCALKLYRKFGQA PHAMRLAMQLNDKKLIEDIF NSCSDWSMKKQLAFMMGRQN TVVQLNDDEDDRNEDLVEIM

Hazt -------------------- -------------------- -------------------- -------------------- --------------------

Meda QAAYNRVCLYLTSCVPYVPE PENTTLLKTALDIFRKFDKF PQALRLALQLNDQKLIHEIF LSCKDPIMQKQLAFMLGRHQ HFLADLDE-ND-YDDVIEIM

Afos ESAYPRVCLYLTSCVPYVPD PENTNLLNTALDIFKKFNQL PQAMRLALQLNDREKVEEIF NNCTDTTIRRQLAFMLGRQQ FYLELPEDTED-YDDLNDIM

Dmel ESAYPRVCLYLQSCYPYVPD PDNTIILETALQLSRKFNQY TQAMRLALMLNDMDKIGEIF KEPKEPAMQKQLAFMLARQQ ICLELDELVPD-YDDLMEIM

Tcas ENTYPRVCLYLISCVPYVAD PENTTLLQTALLLFRNFEQY PQALRLAMQLNDHNLIEEIF TSCPDSTVQKQLAFMLGRQQ IYIEIPESTPE-YEDLVEIM

Pmon KDAYSRVCLYLTSCVPYVPD PENTNLINTALQLYRKFGQY PQAMRLALQLHDFDLIKQLF DECPDKLVRMQVAFMLGRQQ VFLELPEDMDD-YEDLTEIL

Smar ESAYSRVCLYLISCVPYVPD PENTNLLTTALNILRKFNCY PESLRLAMMLNDMPLIEKIF TNCKDTYVQKQMAFMLGRQQ IYLELTESVED-YDDLMEIM

Tcal KSAFQRVCLYLTSCVPYVPD PENTNLLKTTYKIFRKFEQY PQAMRVAMQINDPELIKAVF MECPEPLLQKQLAYMMGRQQ HYMELDE--ND-FDEVVEIM

Crog SGAYNRVCLYLTSCVPYVPS PENIMLLETSVKIFRKFDQY PRAMRLALQLNRPELVLDIF MSCKDPLMQKQLAYMLSRQQ HYVELDE--EE-FDEINEIL

Dpul EAAHNRVCLYLSSCVPYVPD PENVNLLKTALSIFRKFQQW PQALRLALQINDHALIEEVF LACNDGTMRKQLAFMLGRQQ IFLELNEELED-YDDLIEIM

Aver SNSHLNNNFLNLAREL---- -------------------- -------------------- -------------------- --------------------

Csin -------------------- -------------------- -------------------- -------------------- --------------------

Isca SNTSINDHFMALARELDIME PKTPEDIYKSHLD-NTR--- -------------------- -------------------- ---------MFSATASLGLI

Eaff SNVQLNNHFLNLARELDIME AKTPDDVYKSHLD-NVRPGF G--GGAVDSARQNLASSFVN GFINTGFGQDKLLM-EDGNK WLYKNKEHGMLSATASIGLV

Lcyp SNSHLNNYFLNLARELDIME AKTPDDVYKSHLD-NVVRAF G--SSSVDSARQNLASSFVN GFVNAGFGQDKLLLVEDGQK WLYKNKEHGMMSAAASIGLI

Lsal -------------------- -------------------- -------------------- -------------------- --------------------

Aamp NNTDLNTNFLSLARELDIME PKTPEDIYKSHLD-NARPSF G---ANVDSARQNLASSFVN GLVNAGFGKDKLMT-DDGNK WLYKNKEHGMMSAMASFGLV

Afra SNTQLNANFLSLARELDIME PKTPEDIYKSHLE-NSRPSY A--SAQVDSARQNLASSFVN GFVNAGFGKDKLLLCEDGSK AIYKNKEHGMFSTTASLGLL

Hazt -------------------- -------------------- -------------------- -------------------- --------------------

Meda SNSHLNNNFLNLAREL---- -------------------- -------------------- -------------------- --------------------

Afos SNVQLNNHFLNLGRELDIME AKIPDDVYKSHLD-NVRS-Y G--GSSVDSARQNLASSFVN GFINTGFGKDKLLM-EEGNK WLYKNKEHGMLSATASIGLV

Dmel SNANLNKHFLNLARELDIME PKTPEDIYKSHLD-NSRSRF A--SIQVDSAKQNLAASFVN GFVNAGFGVDKLLS-EDGNK WLYKNKEHGMLSATASLGLI

Tcas ANCHLNNHFLNLARELDIME PKTPEDVYKSHLE-NTRPQF G--SNQVDSARQNLAASFVN GFVNAAFGQDKLLM-EDGNK WLYKNKEHGMLSATASLGLV

Pmon SNSHLNTHFLSLGRELDIMD PKTPEDIYKSHLE-NNRPTF G--PGQVDSARQNLASSFVN GFVNAAFGTDKLLT-EDGNK WLYKNKEHGMLSATASLGLI

Smar SNAHLNNHFLSLARELDIME PKTPEDIYKSHLE-SSRPTF GPTGSQVDSARQNLASSFVN GFVNAAFGQDKLLV-EDGNR WLHKNKEHGMMSAMASLGLI

Tcal SNIHLNHHFLNLARELDILE PKTPDDVYKTHLD-NVRP-F G--GGGMDSAKHNLASSLVN GFVNAGFGKDKLLM-EDGNK WLYKHKDAGMMSATASIGLV

Crog SNSHLNGHFLNLARELDILE PKTPEDVYKSHLD-NIRTPF G--SSSVDSARQNLASSFVN GFVNAAFGSDKLIV-DEGNK WICKNKEHGMMSAMASLGMI

Dpul SNTHLNNHFLSLARELDIME PKTPEDIYKSHLDNNSRSAF G--GGQVDSARQNLASSFVN GLVNAAFGQDKLLM-EDGNK WLYKNKEHGMLSATASLGLL

Aver -------------------- -------------------- -------------------- -------------------- --------------------

Csin -------------------- -------------------- -------------------- -------------------- --------------------

Isca LLWNVDGGLTQIDKYLYSTE DYIKAGAILACGIVNCGVRN ECDPALALLSDYVLHSSTLM RIGSILGLGLAYAGSNNKEV IVLLLPVFT-----DPKSTM

Eaff LLWDVDGGLTQIDKYLYSPE DWIKSGALLACGIVNTGVRN ECDPAFALLCDYVLHSSNVM RLGAIFGLGLAYAGSNRADV IELLLRVFA-----DSKSNM

Lcyp LLWDLDGGLAQIDKYLYLND DNIKAGTFLACGIVNARVRN ATEPAYGLLSDYVSKPPNVT RIGAIFGLGLAYAGTNNANA LSCILKVFA-----DAKANM

Lsal -------------------- -------------------- -------------------- -------------------- --------------------

Aamp LLWDVDGGLTQIDKYLYSNE DFIKAGALLACGIVNSGVRN ECDPALALLSDYLLHQNNLI RVGSILGLGIAYGGTNREDV LSNLLPAMQ-----DPKSNK

Afra LLWDLDGGLTQIDRFMYSSD ENLKAGALLACGIVSCGVKN DCDPAWALLSDYVLNDKPTV RIGSIFGLGLAYSGTDRLDI TSHLCTVLS-----DPKSSN

Hazt -------------------- -------------------- ----------------MFIL RVGSVLGLGLAYAGSNRDDV IKVLLPLLT-----DEKSSL

Meda -------------------- -------------------- -------------------- -------------------- --------------------

Afos LLWDVDGGLTQIDKYLYSRE DWIKSGALLACGIVNTGVRN DCDPALALLSDYVLHSSNVM RLGAIFGLGLAYAGSNRTEV IQLLLPVFA-----DSKSNM

Dmel LLWDVDGGLTMIDKYLYSTD DNIKSGALLACGIVNCGIRN EVDPAHALLSDYIDNQNSCM RVGAILGLGIAYAGSNRSIV IDTLKTVFSFGSNNKSSASV

Tcas LLWDVDGGLTPIDKYLYSSE DHIKSGALLACGIVNCGVRN ECDPALALLSDYVLHNTNTM RIGAIVGLGLAYVGSNREAV LSLLTPVFS-----DSKSNM

Pmon LLWDVDGGLTQIDKYLYSSE DFIKSGALLACGIVNSGVRN ECDPALALLSDYIGHKTMVI RIGAILGLGLAYSGSNREAV LNVLTPVLS-----DEKSSL

Smar LLWDVDGGLTQIDRYLYSSE DYIKSGALMACGIVNCGVRN ECDPALALLSDYVLHNTTII RIGAIVGLGLAYAGSNREDV LSLLIPVMS-----DPKSTM

Tcal LLWDVDGGLTQIDKYLYSPE DHIKSGALLACGIVNTGVRN DCDPALALLSDYITHDSMIM RAGATLGLGLAYSGSNREEV IGHLLPVFA-----DSKASM

Crog LLWDVDGGLLKIDKYLYCQE DPIKAGALLACGIVNAGVRN DCDPALALLSDYILHENEIM RCGAIFGLGLAYAGSNRSDV ISLLLPVFG-----DDKVSM

Dpul LLWDVDGGLTQIDKYLYSSE EYIKAGALLACGIVNTGVRN ECDPALALLSDYVLHSSNVM RIGAILGLGLAYAGSNRDDV LSLLLPVLS-----HPKSSM

Aver -------------------- -------------------- -------------------- -------------------- --------------------

Csin -------------------- -------------------- -------------------- -------------------- --------------------

Isca EVLAVTSIACGMVAVGSCNG DVTSTIMTALLEKSET---D LKDTYARFLPLGLGLCYLGK QEGAETITAALEVLPEPFRS MATTLVDICAYAGTGNVLKI

Eaff EVIGVTALATGMVAVGSCNG DVTSTILQTLMEKSGA---D LKDSFAKYLPLGIGLCYLGK QDASEATIAALEVIPEPFRQ IAVTLVEICAYAGTGNVLKI

Lcyp EVVGITALACGLIAVGSCNN DVTTTLLQTLMEKSEA---D LKDPFAKLLPLGIGLCYLGK QDAAEATLAALEVLPEPFKS MAMTTVDICAYAGTGNVLKI

Lsal -------------------- -------------------- -------------------- -------------------- --------------------

Aamp EVVAVTALACGMIAVGSGNP EVAPAILQVIMDRTSSG--D TKDAFVKFLPLALGLIYLGK QEDAEVITCALEVCDEPFRS MATTMVEVCAYAGTGHVLKI

Afra EVIGVTALSLGMIAVGTCNT DVTQALLLTLMEMKETREAS LKEPFAKFLPLGLGLCFLGK QEEAEATIAALEGLPEPFHS MASTLIDVCAYAGTGNVLKI

Hazt EVVGVTSVALGLVAVGTTNQ EVGEALVTTLLAKFPF---Q LKDPFAKFISLGLALTYLGC QDKCEVVLSCVEGLPSPFRQ MTKTLIEVCAYAGTGNVLKV

Meda -------------------- -------------------- -------------------- -------------------- --------------------

Afos EVIGVTSLAVGMIAVGSCNG DVTSTILQTLMEKSAT---D FKDSFAKYLPLGIGLCYLGK QDAAEATIAALEVIPEPFRQ TATTLVEICAYAGTGNVLKI

Dmel EILGITALSLGLISVGSCNS EITEILLQTIMGLTKA---D LKDTYTRFLFLGLGLLYLGR QKSTEAVMMTLEVLEEPYRS MATTMVDICAYAGTGNVLKI

Tcas EVIGMAALACGMIAVGSGNS QITTAIMQTLMEKSEA---D LKDTYARFLPLGLGLCHLGK QEGIETIIAALEVIPEPFKS MAVTMVDICAYAGTGNVLKV

Pmon EVVGITALACGMIAVGTTNQ LVVEALVTTLLAKSET---E LKDTYARFISLGLALAYLGC QDKCEVVLSCIENLPAPFRQ MTATLIEVCAYAGTGNVLKV

Smar EVTAMAALSCGMIAVGSCNG EVTSTILQTLMEKTEQ---D FKDTYARFLPLGLGLCYLGK QESIEAIIAALEVIPEPFKS MSTTMVDVCAYAGTGNVLKV

Tcal ENISLTALALGLIGVGSCNN EITTVLLQTLMEKSES---D LKDPFAKFLPLGIGLCYLGK QEAAEATIATLEVLSDPFKS MAMTLVDVCAYAGTGNVLKI

Crog EIVGITALACGLIAVGSCNS DVTSTILQILMEKKEA---D LKDPFAKFLPLGIGLCYLGK QEMSEATLAALEAIPKPFNF MATTLVDICAYAGTGNVLKI

Dpul EVVGVAALACGLVAVASCNG DVTSALLQTLMEKSEV---E LKDTFSRFLPLGLGLTYLGK QEAVEAVVLALEVLAEPFKS MASTMVEVCAFAGTGNVLRV

Aver -------------------- -------------------- -------------------- -------------------- --------------------

Csin -------------------- -------------------- -------------------- -------------------- --------------------

Isca QHLLHICSEHYEVQE----- -KEEKKDDK------KDKKE DKEE--------------KS TGPPDLSSQQSVAVLGIALI AMGEEIGSEMAFRAFGHLLR

Eaff QQLLHICSEHYEQEK----- -ETEKKDKK------KDEKK DEKKDEKKDDKKE---EKEE EASHDMSGQQAVAVLGLGLI AMGEDIGSEMLYRTLGHLMR

Lcyp QGLLHICSDHYEPPE----- -KEDKKESSTGSSSSSKSKK EEKK--------------DE NSPLDLSSQQAVAVIGLALI AMGEDIGSEMLLRHFGHLLR

Lsal -------------------- -------------------- -------------------- -------------------- --------------------

Aamp QKLLHICSDHYEPEK----- -ADDKKDDK------KDDKK SGSASAKDKEDKGAAAAKKE DKPVDHSSQQSVAVLGLALI AMGEDIGSDMMLRMFGHLLR

Afra QQLQHICSEHYEPEE----- ----KNKEN------KKEKK EKEE--------------AK DDKADMSMQQAVATLGLGLI AMGEDIGSDMAYRSFGLLLR

Hazt QEMLHICTDHYETDDSSSSS KAKDKKEKTNKD---KQDKE KKDEAAKEKDAKE-SEKKED DKEVDLSSQQSIAVLGIALI AMGEDIGADMCFRQFGNLLR

Meda -------------------- -------------------- -------------------- -------------------- --------------------

Afos QQLLHICSEHYEAEK----- -EPEKKDKK------KEEKK EGEGESSKDG--------EG SGSVDLSGQQAIAVLGLGLI AMGEDIGSEMMYRTLGHLMR

Dmel QQLLHICSDHYETSS----- -ADDEKEKS------KKDKG KDKEKEKEKE-------KEK EREKDLSATQSIAVLGIALI AMGEDIGAEMAYRSFGNLLR

Tcas QHLLHICSEHYEPAD----- -KDESRDKNK-----DKSKE KEDK--------------EK ----DLSARQAVAVLGIALI SMGEDIGSDMAFRTFGHLLR

Pmon QEMLHICTDHYDQEE----T KSKDKKDK---------DKD KKED--KDKDKKE-EKDKAE EKEVDLSSQQAVAVLGIALI AMGEDIGAEMSFRQFGNLLR

Smar QSLLHICSEHYDHGD----- -KDEKKDDKK-----DKDDK DKKE--------------EK TPTVDLSSQQAIAVIGIALI AMGEDIGSEMAFRTFGHLLR

Tcal QHLLHICSEHYEPSN----- -DDDDKKDK------KKDKK DDKK--------------TT TDEVDLSMQQSVAVIGLALV AMGEDIGSEMLFRTFGHLLR

Crog QELLHICSEHHEETN----- -SKESSKSK------SSSKK EDEK--------------KE DAALDLSMQQAVAVLGLALV AMGEDIGSEMLFRTFGHLLR

Dpul QRLLHICSEHHEPQE----- -KEDRKESKE-----TAAKK DE------------------ ---TDLSSQQAVAVIGLALI GMGEDIGAEMAFRTLGHLLR

Aver -------------------- -------------------- -------------------- -------------------- --------------------

Csin -------------------- -------------------- -------------------- -------------------- --------------------

Isca YGEPVIKKAVPLALALISVS NPRLNILETLSKFSHDSDNE VAHNAIFGLGIVGAGTNNAR LAAMLRQLAQYHAKDPNHLF MVRLAQGLAHMGKGTLTLGP

Eaff YSEPCIRRAVPLALSLISVS NPQLNILDTLSKFSHDPDPE VAQNSIFGLGLIGAGTNNAR LAATLRQLAQYHAKEQNNLF MVRIAQGLVHLGKGTLTLNP

Lcyp YCEPVIRRAVPLALGLISVS NPQLNILDTLSKFSHDADAE VAHNSIFAMGLLGAGTNNAR LAALLRQLAQYHAKDTNNLF MVRIAQGLVHMGKGTMSLDP

Lsal -------------------- -------------------- -------------------- -------------------- --------------------

Aamp YCEPCIRRAVPLALGLVSVS NPKLSILDTLSKFSHDADQE VAHNAIFAMGLVGAGTNNAR LAATLRQLAQYHARDPNNLF MVRIAQGLTHMGKGTLTLNP

Afra YSELAIRRVVPLALGLLYVS NPKLNAIDTLSKFSHDADPE VAYNAILAMGLLGAGTNHAR LATMLRQLAQYHAKDANHLF MVRIAQGLVHMGKGTLTLSP

Hazt YCEPVIRRAVPLALALSSVS NPKLNILDTLSKFSHDSDHE VAHNAIFAMGLVGAGTNNAR LAAMLRQLAQYHAKDPNNLF MVRLAQGLTHLGKGTMSLSP

Meda -------------------- -------------------- -------------------- -------------------- --------------------

Afos YCEPVIRRAVPLALALTSVS NPKLNLLDTLSKFSHDSDAE VAQNAIFGLGMIGAGTNNAR LAATLRQLAQYHAKDQNNLF MVRIAQGLVHLGKGTLTLNP

Dmel YCEPAIRRAVPLALGLISAS NPKLNIIDTLSKFSHDSDAE VAHNAIFAMGLVGAGTNNAR LASMLRQLAQYHSKDPSNLF MVRIAQSLTHLGKGTLSLSP

Tcas YCEPVIRRAVPLALGLISVS NPKLSILDTLSKFSHDSDAE VAHNAIFAMGLVGAGTNNAR LAAMLRQLAQFHAKDPNNLF MVRIAQGLTHLGKGTLTLSP

Pmon YCEPVIRRAVPLALGLISVS NPRLNILDTLSKFSHDSDTD VAHNAIFAMGLVGAGTNNAR LAAMLRQLAQYHAKDPNNLF MVRLAQGLTHLGKGTMTLSP

Smar YGEPTIRRAVPLALGLVSVS NPKLNILDTLSKFSHDSDAE VAHNAIFALGLVGAGTNNAR LAAMLRQLAQYHAKEPNNLF MVRIAQGLAHLGKGTLTLCP

Tcal YCEPVIRRAVPLALGLISVS NPQLPILDTLSKFSHDADPE VAHNSIFALGLIGAGTNNAR LAAMLRQLAQYHSKDPNNLF MTRVAQGLVHMGKGTLTLNP

Crog YCEPVIRRTVPLALGLISVS NPQLIILDTLSKFSHDSDSE VAHNSIFAMGLIGAGTNNAR LSAMLRQLAQYHSKDQNNLF MVRIAQGLLHMGKGTLSLNP

Dpul YGEPCIRRAVPLALGLISVS NPRLNVLDTLSKFSHDSDSE VALNSILALGLVGAGTNNAR LAAMLRQLAQYHAKDSNALF MVRLAQGLTHLGKGTLTLQP

Aver -------------------- -------------------- -------------------- -------------------- --------------------

Csin -------------------- -------------------- -------------------- -------------------- --------------------

Isca YHSDRQLMSPVATAGLLATL LAFLDVKSIILGKSHYLLYC LVTAIQPRMLVTFDEKLQPL PVPVRVGQAVDVVGQAGKPK TITGFQTHTTPVLLAYGERA

Eaff FHSDRTLMSPVAVAGLLSVL VALLDVKNTILGKSHYLLYG LATAMQPRMLVTFDTELRPL PVSVRVGQAVDVVGQAGKPK TITGFQTHTTPVLLAHGERA

Lcyp FHSDSQLMSPVAVAGLLGCI FSFLDVKSTILGKAHYLLYS LAPAIQPRMLVTFDEELNPL PVSVRVGQAVDVVGQAGKPK TITGFQTHMTPVLLSHGERA

Lsal -------------------- -------------------- -------------------- -------------------- --------------------

Aamp WHSDRALMSPVAVAGLMATL TAFLDVKSLILGKSHYLLFC LTTAMQPRMLVTFDEELKPL PVSVRVGQAVDVVGQAGKPK TITGFQTHTTPVLLAYGERA

Afra YHSDRQLMRPVAVAGLLGLL VACIDVKQTILGKSHYLFYS VAAAIQPRMLVTFDEELKAL PVSVRVGTAVDVVGQAGKPK TITGFQTHTTPVLLSHGERA

Hazt YHSNRQLMAPVAVAGLLATC MAFLDTKTLILGKSHYLLYC LVSAMQPRMLVTFDEHLNPL PVAVRVGQAVDVVGQAGRPK AITGFQTHTSPVLLAYGERA

Meda -------------------- -------------------- -------------------- -------------------- --------------------

Afos FHSDRQLMSPVAMAGLMSVL VAMLDVKTTILGKSHYLLYG LSTAMQPRMLVTFDEELRPL PVSVRVGQAVDVVGQAGKPK TITGFQTHTTPVLLAYGERA

Dmel YHSDRQLMNPMAVAGLMATL VSLLDVKNLILNRSHYLLYT LVPAMQARMLITFDEELNQL QVPVRVGIAIDVVGQAGKPK TITGFQTHTTPVLLAIGERA

Tcas YHSDRQLMSPVAVAGLMATL VGFLDVKNIILGKSHYLLYT LAAAMQPRMLVTFDEDLNPL PVPVRVGLAVDVVGQAGKPK TITGFQTHTTPVLLAHGERA

Pmon YHSNRQLMSPVAVAGLLATC MAFLDTKTLILGKSHYLLYC LVTAIQPRMLVTFDEQLNPL PVSVRVGQAVDVVGQAGKPK TITGFQTHTTPVLLAHGERA

Smar YHSDRQLMNVVATGALLATL TAFLDVKTIILGKSHYLLYY LVAAMQPRMLVTFDENLRPL PVAVRVGQAVDVVGQAGKPK TITGFQTHTTPVLLAYGERA

Tcal YHSDSQLMSPVAVAGLLGCI ISFLDVKSTILGKSHYLLYT LATALQPRMLVTFDEELRPL SVSVRVGQAVDVVGQAGKPK TITGFQTHTTPVLLASGERA

Crog YHSDNQLMSPVAVAGLLGVL ISFLDVKQTILSKSHYLLYG LCTAMQPRMLVTFDENLSPL PVSVRVGAAVDVVGQAGKPK TITGFQTHTTPVLLSCGERA

Dpul YHSERQLMNPVAVAGLLSTV IACLDVKNIILSKSHYLLYT LAASLQPRMLITFDEELRPL PVSVRVGQAVDVVGQAGKPK TITGFQTHTTPVLLAHGERA

Aver -------------------- -------------------- -------------------- -------------------- --------------------

Csin -------------------- -------------------- -------------------- -------------------- --------------------

Isca ELATEEYI------------ ---SLTPVLEGFVILKKNEN F-TTMMDIRPNHTIYINNLN EKVKKDELKKSLYAIFSQFG QILDIVALKTLKMRGQAFVV

Eaff ELATEEYI------------ ---PVTTIMEGFVILKKNPD Y---MMENRPNHTIYINNLN EKIKKEDLKKSLYAIFSQFG QILDIVALKTLRMRGQAFVI

Lcyp ELATEEYT------------ ---PYSPIMEGFVILKKNPD Y----ME-RPNQTIYINNLN EKVKKDELKKSLYAIFSQFG QILDIVALKTLKMRGQAFVI

Lsal -------------------- -------------------- -----MDARPSHTIYINNLN EKVKKEDLKKSLYAIFSQFG QILEIVALKTLKMRGQAFVI

Aamp ELATEEYV------------ ---PLTSVLEGFVILRKNPD Y-VA-MDIRPNQTIYINNLN EKVKKPELKESLYGLFSQFG PILDVVALKTPKMRGQAFIV

Afra ELATDEYL------------ ---PLTPVLEGFVILRKNPY F-EK-MDIRPNHTIYINNLN EKIKKEELKKSLYAIFSQFG AILDIIALKTLKMRGQAFVI

Hazt ELATDEYL------------ ---PLTPIMEGFVILRKNPD F-QA---------------- -----------LYAIFSQFG QILDIIALKTLKMRGQAFVV

Meda -------------------- -------------------- -------------------- -------------------- --------------------

Afos ELATEEYL------------ ---PVTTIMEGFVILKKNPD Y----MDNRPNHTIYINNLN EKIKKEDLKKSLYAIFSQFG QILDIVALKTLKMRGQAFVI

Dmel ELATDEYL------------ ---ALTPVMEGFVILKKNPN F-VK-MEMLPNQTIYINNLN EKIKKEELKKSLYAIFSQFG QILDIVALKTLKMRGQAFVI

Tcas ELATEEYI------------ ---ALTPIMEGFVILRKNPD FYPS-MDIRPNNTIYINNLN EKIKKEELKKSLYAIFSQFG QILDIVALKTLKMRGQAFVI

Pmon ELATDEYL------------ ---PLTSIMEGFVILKKNPD Y-ETMLDIRPNHTIYVNNLN EKVKKEELKRSLYAIFSQFG QILDIVALKTLKMRGQAFVI

Smar ELATEECIFLKFDFYSFEIE VVSSLMPIMEGFVILH-SCE F--G-MSTQPNNTIYINNLN EKTKLSELKKSLFAIFSQFG QILDIVALKTLKKKGQAFVI

Tcal ELATEEYI------------ ---PVTPIMEGFVILKKNPD Y----MENRPNHTIYINNLN EKVKKEDLKKSLYAIFSQFG QILDIVALKTLKMRGQAFVV

Crog ELATEEYT------------ ---PMTPIMEGFVILKKNPN Y----MDVRPNHTIYINNLN EKVKKDDLKKSLYAIFSQFG QILDIVALKTLKMRGQAFVI

Dpul ELATEDYV------------ ---SLTPIMEGFVILRKKKV D----MDVRPNSTIYINNLN EKVKKEELKKSLYAIFSQFG QILDIVALKTLKMRGQAFVI

Aver -------------------- -------------------- -------------------- -------------------- --------------------

Csin -------------------- -------------------- -------------------- -------------------- --------------------

Isca FKDLNSATNALRSMQGFPFY DKPM-------VSVIVLPRR WTF----------------- -------------------C --------------------

Eaff FKEISSSTNALRSMQGFPFY DKPMKIAYGKTDSDVIAKIK GTFQERPKRAPV-------D KKKGKKGGKSGATVGA---- --------AGAG--------

Lcyp FKDISAATSALKSMQGFPFY DKPMRIAFAKTDSDVVAKQK GTFQERPKRLPGA--GAGEA KKKKKKPHAEGAGAGRH--G --------GGGG--------

Lsal FKEITSATNAMRSMQGFPFY DKPMRIAFSKTDSDVIAKIK GTYQERPKKAAS-------D SKKAKKRAAKEAAARQI--C --------ALAG--------

Aamp FKEIGAATNALRSMQGFPFY DKQMRLQYGKSESDAVSKMK GTFRQEGGKRKDP-----SA PKKAKKAKTSSTAAATT--A KP------SEDG--------

Afra FKDIGAATNALRSMQGFPFY DKPMRIQYAKVDSDAIAKVK GTFTEKKKKVKE-------I KKDKKKEQRKEAPLPPPAYG APAPFTPSAMPAV-------

Hazt FQEIHSATTALRAMQGFPFY DKPMKIAYSKSDSDVIAKIK GTYEERAHKNKD-------D KKKRKKEAKAAAAAAAAASA ATSAAVAPATAAAGSALAAA

Meda -------------------- -------------------- -------------------- -------------------- --------------------

Afos FKEIGSATNALRSMQGFPFY DKPMRIAYSKTDSDLIAKMK GTFTERPKKAPM-------D RKKGKKGKSGGAAAGA---G AMGS----AGAGMG------

Dmel FKEIGSASNALRTMQGFPFY DKPMQIAYSKSDSDIVAKIK GTFKERPKKVKPPKPAPGTD EKKDKKKKPS---------- --------SAEN--------

Tcas FKEIQSSTNALRSMQGFPFY DKPMRIQYAKTDSDVISKMK GTFQERPKKIRPP--PSKED EAPKKKKKNKDPN------- --------------------

Pmon FKEIQSATNAMRAMQGFPFY DKPMKIAYAKTDSDLIAKQK GTYKERGSKRKE------ED KKKKKGKEAKGATAVPA--G AAAAAKAGAGAGV----VPP

Smar FKQIESGTNAMHAMQGFPFY DKPMRIQYAKSDSDVIAKIK GTFVERPKKKPQR--P--AE AKKAKKKTKEQAKSQQTH-- --------PAAS--------

Tcal FKDISSATQAMRSMQGFPFY DKPMRINYSKTDSDAVAKLK GTFTERPKRPAGG--PAGAE SKKAKKRAAKEAAVRQ---G --------GGSG--------

Crog FKEITSGTNALRSMQGFPFY DKPMRISFSKTDSDIIAKLK GTFTERPKKASG-------D SKKAKKKAAKEAAARQL--C --------ALAG--------

Dpul FKEVTSATNALRGMQGFPFY DKPMRIQYAKTDSDVIAKMK GTFQERPAKPKP-----EGE PKKKKKKESKQQQVVTP--G --------SFGGP-------

Aver -------------------- -------------------- -------------------- -------------------- --------------------

Csin -------------------- -------------------- -------------------- -------------------- --------------------

Isca -------------------- -------------------- -------------------- -------------------- -------------------S

Eaff -------------------- ---MQTQG--VENAPPNQIL FLTNLPSETNEMMLSMLFNQ FPGFKEVRLVPGRHDIAFVE FENEMQSAAARDALQGFKIT

Lcyp -------------------- ---SASNS--NMEAPPNQIL FLTSLPEETNEVMLSMLFNQ FPGYKEVRLVPGRHDIAFVE FENEIQSAAARDALQGFKIT

Lsal -------------------- ---ANAVH--PGEAPPNQIL FLTNLPDETNEMMLSMLFNQ FPGFKEVRLVPNRHDIAFVE FENEVQSAAARGSLQGFKIT

Aamp -------------------- ---DGADE--AADLPPNQIL FLTSLPEETNEMMLQMLFNQ FPGFKEVRLIPGRHDIAFVE FDTDAQSAAARSALQGFKIT

Afra --------QP---------- --PIQAAP--VLEQPPNQIL FLTGLPEETNEMMLSMLFNQ FPGFKEVRLVPGRHDIAFVE FESEIQSSAARNALQGFKLT

Hazt GGGISLAAQPPKPAAARSTL FSGATGVGGVVPEQPPNQIL FLTNLPDETSEMMLSMLFTQ FPGFKEVRLVPGRHDIAFVE FENEIQSATAKEALQGFKIT

Meda -------------------- -------------------- -------------------- -------------------- --------------------

Afos --G----------------- --GASMQG--VDNAPPNQIL FLTNLPQETNEMMLSMLFNQ FPGFKEVRLVPGRHDIAFVE FENELQSAAARDALQGFKIT

Dmel -------------------- ---SNPNA--QTEQPPNQIL FLTNLPEETNEMMLSMLFNQ FPGFKEVRLVPNRHDIAFVE FTTELQSNAAKEALQGFKIT

Tcas -------------------- --RIPGGS--NAEQPPNQIL FLTNLPDETSEMMLSMLFNQ FPGFKEVRLVPNRHDIAFVE FENELQSGAAKDALQGFKIT

Pmon RPG--LFNQPP--------P VHGAGGIG--VPEQPPNQIL FLTNLPDETSEMMLSMLFTQ FPGFKEVRLVPGRHDIAFVE FENEMQSAAAKDALQGFKIT

Smar -------------------- ---VVGFG--GEQQPANQIL FLTNLPENTNEVMLSMLFSQ FPGLKEVRLVPGRCDIAFVE YENDLQSAAARQALQGFRIT

Tcal -------------------- ---TSGGP--VLEAPPNQIL FLTNLPEETNEMMLSMLFNQ FPGFKEVRLVPGRHDIAFVE FENEVQSTGARDALQGFKIT

Crog -------------------- ---ANAVH--PGEAPPNQIL FLTNLPDETNEMMLSMLFNQ FPGFKEVRLVPNRHDIAFVE FENEVQSAAARESLQGFKIT

Dpul --A--TYGYP---------- --AIGGAG--VPEQPPNQIL FLTNLPEETNEMMLSMLFTQ FPGFKEVRLVPGRHDIAFVE FENEVQSAGARESLQGFKIT

Aver -------------------- -------------------- -------------------- -------------------- --------------------

Csin -------------------- -------------------- -------------------- -------------------- --------------------

Isca PC--------------MCG- RIPVLLCAVLATVL-----R YTAAFYLPGLAPVNYCKEGA TSSQCQSKVNLYVNRLDSDE SVIPYEYQHFDFCT--SSE-

Eaff PTTAMKISFAKKMHSLISEK MLKILLISLLL--------G PVWSFYLPGLAPVTYCKS-A DVTTCQNDIKLFVNRLNSEE SVIPFEYHHFDFCE--AAE-

Lcyp PTAAMKISFAKK----MMR- ---SATWTWLLTFS-----G LAQCFYLPGLAPVTYCDNPG -DDQCQSEIKLYVNRLNSEQ SILPYEYKDFDFCP--APED

Lsal PNASMKISFAKK-------- -------------------- -------------------- -------------------- --------------------

Aamp PTHAMRISFAKK-------- -------------------- -------------------- -------------------- --------------------

Afra PTHGMKISFAKK----MKN- ------FAKLLVIF--SCTY GVSCFYLPGLAPVNYCKADN EKNNCKSTVQLFVNRLTSEE HILPYDYSQFDFCT--ASE-

Hazt PTHAMKIVFAKK-------- -------------------- -------------------- -------------------- --------------------

Meda -------------------- -------------------- -------------------- -------------------- --------------------

Afos PTTAMKISFAKK-------- -------------------- RSQGFYLPGLAPVTYCDRDE -TNICTKEIKLFVNRLNSEE SVIPYEYQHFDFCQ--TKE-

Dmel PTHAMKITFAKKM-ILLSG- ------LLPLLGVVLLHLAT PTQAFYLPGLAPVNFCKKTD VSSTCKSEILLYVNRLNTEE SVIPYEYHHFDFCL--GEE-

Tcas PTHAMKISFAKK----M--- ------YLPLLCLLFLWHET RVEAFYLPGLAPVNYCRKGE -SDTCKSEVLLYVNRLNTEE SVIPYEYNHFDFCQ--PDEN

Pmon PTHAMKIAFAKK-------- -------------------- -------------------- -------------------- --------------------

Smar AANAMKVSFAKK----MTR- LSRVMLVVKFILVF-----E LSSAFYLPGLAPVNYCRT-- ESPNCKSKVALYVNKLDSEE SVIPYEYHHFDFCT--VDE-

Tcal PTTSMKISFAKK----MKS- ------LALLVGVV-----A VCHAFYLPGLAPVTYCERAD -DGKCQTEIKLYVNRLNSEE SVIPYEYDHFDFCK--SDS-

Crog PNASMKISFAKK----MRN- ---RLSGGHLLLLL-TFCAL NSHGFYLPGLAPVTYCEVPD DQNRCQTQIPLFVNRLNSDE SVIPYEYDHFDFCP--APES

Dpul PTHSMKISFAKK----MST- ---KQIFSSILLLL-----H LSYGFYLPGLAPVNYCIKNK QSDSCKNDVPVFVNRLNSEE AIIPYEYHHFDFCT--DDH-

Aver -------------------- -------------------- -------------------- -------------------- --------------------

Csin -------------------- -------------------- -------------------- -------------------- --------------------

Isca ATSPAENLGQVVFGERIRLS PYIMNFM----ENK-TCTPL C-SKFYSK---SQPESMAKL ELLKKGMMKQYKHHWIVDNM PV-TWCYLVEPNSQ-FCSMG

Eaff DSSPAENLGQVVFGERIRPS PYTIKFG----EPQ-TCTIL CATKKYRS---NDAESVKKL AQLKKGIFMNYQHHWIVDNM PV-TWCYQVENQMH-YCSTG

Lcyp ATSPSENLGQVVFGERIRPS PYKISFN----KDSGKCKRV C-KKSYDL---SKKEDKDKV MFMKRGMSMNYQHHWIVDNM PV-TWCYQVENNQQ-YCATG

Lsal -------------------- -------------------- -------------------- -------------------- --------------------

Aamp -------------------- -------------------- -------------------- -------------------- --------------------

Afra EVSPVENLGQVVFGERIKSS PYELKFL----EDA-SCSQV C-TKEYDV---NDKDSKRKL DFLLEGIKKNYQHHWIVDNM PV-TWCYNIESNRQ-YCNRG

Hazt -------------------- -------------------- -------------------- -------------------- --------------------

Meda -------------------- -------------------- -------------------- -------------------- --------------------

Afos DHSPAENLGQVVFGERIRPS PYSIRFN----EPK-KCVKV CDTKVYKA---NDNEAKKKL ALLKKGMLMNYQHHWIVDNM PI-TWCYQVENQQH-YCATG

Dmel QNSPVENLGQVVFGERIRPG PYKIQFL----ENQ-QCATT C-VKTYKG---DDPASNRRM MVLKKGISLNYQHHWIVDNM PV-TWCYPLENGKQ-YCGIG

Tcas QPSPVENLGQVVFGERIRPS PYILEFM----KNI-TCKEV C-TRKYSG---SDPSANRRL SILRKGISLNYQHHWIVDNM PV-TSCYETEDQKQ-FCTTG

Pmon -------------------- -------------------- -------------------- -------------------- --------------------

Smar SDSPVENLGQVVFGERIRPS PYKILFG----TDE-ECVTL C-SRRYSG---DNKESAAKL S--------------IIDNM PV-TWCYKAEGGSN-YCATG

Tcal SSGPSENLGQVVFGERIRPS PYKIVFN----KNNDKCQTV C-TKEYNL---RNADSQKKL IQLRKGMSMNYQHHWIVDNM PV-TWCYPVESGQK-YCATG

Crog AYTPAENLGQVVFGERIRPS PYKIMFG----KKV-ECATV C-TKTYSP---STKESLKKL KQVKRAIKMNYKHHWIMDNM PI-TWCYHVKNEEQLYCARG

Dpul ETGPVENLGQVVFGERIRSS PYKLEFK----NNQ-EWNYA C-KKSYQP---GNQDDLKKI KELIHGISLNYQHHWIVDNM PV-TWCYQGEPGQQ-FCSTG

Aver -------------------- -------------------- -------------------- -------------------- --------------------

Csin -------------------- -------------------- -------------------- -------------------- --------------------

Isca FPMGCFTYRTSQPKGMCGIY -SAYS-KPDTFYLFNHVDLT VSYHKSDKESWGSSF----- ------IEEGGRIISVKVQP K---------SLKHKAGPN-

Eaff FPMGCYVDKEGKPKDACVIS -HEYS-QSDTYYIFNHVDIT ISYHNGQDEDWGKYL----- -----GNVLGGRIVAVKLVP R---------SINHEGAEKG

Lcyp FPMGCYVNKNGSPQDACVIS -KDYS-QRDTFYIFNHVDIV ITYHNGKQEDWGKFLLN--- -----SNEVGGRIVSVKLEP R---------SIDHSLGSSE

Lsal -------------------- -------------------- -------------------- -------------------- --------------------

Aamp -------------------- -------------------- -------------------- -------------------- --------------------

Afra FPIGCYIDPKGRKSEGCYLS TSNFD-QKDSYFVYNHIDLT IKYHSGKTEPWGIDL----- ------GEGGGRVVAIKVEP R---------SIKHTADGKC

Hazt -------------------- -------------------- -------------------- -------------------- --------------------

Meda -------------------- -------------------- -------------------- -------------------- --------------------

Afos FPMGCYVDKQGEAKDACVIN -REYN-KKDTYYLFNHVDIT IYYHSGQNEDWGKYLGGGNV GGGAAGSTVRGRIVAVKLVP K---------SIDHGDDPEK

Dmel FPMGCLVRSDG---EGCPIN -SIYN-QPMHYYPFNHVDLE ITYHSGQSEDWGIQF----- -------GNSGRIISVKVTP K---------SIKHTDKENP

Tcas FQMGCFA-KDG--RDTCLRT -PD---KQDAYYIYNHVDLT ITYHSGEQEEWGNKF----- ------KSNGGRIISVKVVP R---------SIDHRDKIDC

Pmon -------------------- -------------------- -------------------- -------------------- --------------------

Smar FPMGCYVNKVGKPKYACVLN -RAYK-TPDSYYIFNHVDIT ITYHPGTNEDWGSSL----- -------KESGRIISAKVFM KRWVINIGFYSIKHMAPGPR

Tcal FPMGCFVDKQGNPKDACVIS -RDYT-KPNHFYIFNHIDLE ISYHPGNNEDWGRYL----- -----SDQVGGRIVSVKATP R---------SIDHGASPTD

Crog FPVGCYVNKNGKPKDSCVMS -NKFR-TPDSYYVFNHIDLV VTFNPGENEDWSRYIK---- -----SEEVGGRIVSVKATP R---------SINHKNLPKD

Dpul FPMGCYLPKSGKPKDACVMN -PAFS-KPDTYYIFNHIDLT ITYHGGKGETWGAEL----- ------DDNIGRIL---IGL R---------SL---SVEKD

Aver -------------------- -------------------- -------------------- -------------------- --------------------

Csin -------------------- -------------------- -------------------- -------------------- --------------------

Isca -MC-----DTGEPLTLGV-- -KDEDDINITYTYSVSFVRN DHIRWSSRWDYILESMPQTN IQWFSIMNSLIIVLFLTGMV AMILLRTLHKDIARYNQMDS

Eaff -KCT-D--PAAKPMAIPA-- -TGTADIDITYSYSITFQQN QATKWSSRWDYILESMPHTN IQWFSILNSLVIVLFLSGMV AMILLRTLHKDIARYNQIDS

Lcyp QDCN-AELSAGRPLSLSN-- -EHTGKFDIVYSYTVSFKED NTFRWSSRWDYILESMPHTN IQWFSILNSLVIVLFLSGMV AMILLRTLHKDIARYNESDR

Lsal -------------------- -------------------- -------------------- -------------------- --------------------

Aamp -------------------- -------------------- -------------------- -------------------- --------------------

Afra --------DTNEYLRIPG-- -KPQGKIAITYTYSYKFQET -ETKWSSRWDYLLDSMPNSN IQWFSILNSLVIVLFLSGMV AMILLRTLHKDIARYNQIDS

Hazt -------------------- -------------------- -------------------- -------------------- --------------------

Meda -------------------- -------------------- -------------------- -------------------- --------------------

Afos -ACE-N--PTSPPMAIPG-- -EQREDLKISYTYSVSFEEN SNIKWSSRWDYILESMPHTN IQWFSILNSLVIVLFLSGMV AMILMRTLHKDIARYNQIES

Dmel -NC-----LSTEPLAISENS LKAGEQLEIVYTYSVKFVKN DSIKWSSRWDYILESMPHTN IQWFSILNSLVIVLFLSGMV AMIMLRTLHKDIARYNQMDS

Tcas -SKQ-N--TKLLELKTSL-- -KPKEEYQITYTYSVTFIQN NAVKWSSRWDYILESMPHTN IQWFSILNSLVIVLFLSGMV AMIMLRTLHKDIARYNQIDS

Pmon -------------------- -------------------- -------------------- -------------------- --------------------

Smar -GCS----DNAPPMEIPGSP LKDGDKLQINYTYSVFFKED QNVKWSSRWDYILESMPHAN IQWFSILNSLVIVLFLSGMV AMIMLRTLHKDIARYNQIES

Tcal -DCS-ASLQSKAPLAIPATP -ENNGKLSITYSYTVRFIAN ADVKWSSRWDYILESMPHTN IQWFSIMNSLVIVLFLSGMV AMILLRTLHKDIARYNQIDS

Crog GTCKISNPKSDDPLVIPGG- -DLKGDFSITYSYSVTFEPN YNTKWSSRWDYILESNPHTN IQWFSIVNSLVIVLFLSGMV AMIILRTLHRDIARYNQIDN

Dpul --------PTSPPMIVPK-- -ELKEPLTITYTYSVKFIDD PMTKWSSRWDYLLESMPSAN IQWFSILNSLVIVLFLSGMV AMILLRTLHKDIARYNQIDS

Aver -----------LVHGDVFRP PRKGMILSVFLGSGTQILCM TAITLVFACLGFLSPANRGA LMTCAMVLFVCL-------- -------------GTPAGYV

Csin -------------------- -------------------- -------------------- -------------------- --------------------

Isca GDDAQEEFGWKLVHGDVFRT PRKGMLLSVFLGSGTQIFFM TFITLLFACLGFLSPANRGA LMTCAMVLFVCL-------- -------------GTPAGYV

Eaff GEDAQEEFGWKLVHGDIFRP PRKGMLLSVFLGSGIQLFSM ASITLVFACLGFLSPANRGA LMTCALVLYVN--------- --------------------

Lcyp GEGTQEESGWKLVHGDVFRP PRKGMLLSVFLGSGVQILCM AGITLVFACLGFLSPANRGA LMTCALVLFVCL-------- -------------GTPAGYA

Lsal -------------------- -------------------- -------------------- -------------------- --------------------

Aamp -------------------- -------------------- -------------------- -------------------- --------------------

Afra GDDAQEEFGWKLVHGDVFRP PRKGMLLSVFVGSGVQVFLM TLVTLVFACLGFLSPANRGA LMTCAMVLFVCM-------- -------------GTPAGYV

Hazt -------------------- -------------------- -------------------- -------------------- --------------------

Meda -----------LVHGDVFRP PRKGMLLAVFLGSGTQILCM TAITLVFACLGFLSPANRGA LMTCAMVLFVCL-------- -------------GTPAGYV

Afos GEDAQEEFGWKLVHGDVFRP PRKGMLLSVLLGSGAQLISM VSITLAFACLGFLSPANRGA LMTCALVLYVCL-------- -------------GTPAGYV

Dmel GEDAQEEFGWKLVHGDVFRP PRKGMLLSVFLGSGVQVLVM AMITLAFACLGFLSPANRGA LMTCSMVLFVSL-------- -------------GTPAGYV

Tcas GEDAQEEFGWKLVHGDVFRP PRKGMFLSVLLGSGVQVFFM TLVTLAFACLGFLSPANRGA LMTCAMVLYVLL-------- -------------GSPAGYV

Pmon -------------------- -------------------- -------------------- -------------------- --------------------

Smar GEDAQEEFGWKLVHGDVFRP PRKGMLLSVFLGSGIQVLFM TLITLVFACLGFLSPANRGA LMTCAMVLYVCL-------- -------------GTPAGYV

Tcal GEDAQEEFGWKLVHGDVFRP PRKGMLLSVFLGSGAQILAM SGITLIFACLGFLSPANRGS LMTCALVLYVCL-------- -------------GTPAGYV

Crog GEDAQEEFGWKLVHGDVFRP PRYGMLLSVFLGSGTQVLLM SSITLLFACMGFLSPANRGS LMTCALVLFACL-------- -------------GTPAGYI

Dpul GEDAQEEFGWKLVHGDIFRP PRKGMLLSVFVGSGVQVFIM TLITLIFACLGFLSPANRGA LMTCALVLYVCL-------- -------------GTPAGYV

Aver SARVYKSFGGEKWKSNVLLT SILCPGVVFALFFIMNLVLW YEGSSAAIPFFTLVALLALW LCVSVPLTFVGAFFGFRKRA IEHPVRTNQIPRQIPEQSIY

Csin -------------------- -------------------- -------------------- -------------------- --------------------

Isca SARIYKAFGGEKWKSNVLLT ALVCPGVVFSLFFVLNLLLW AKDSSAAVPFTTLLALLALW FGISLPLTFVGAYFGFKKRV MEYPVRTNQIPRQIPEQSLY

Eaff -------------KSNVLLT SMLCPGIVFAHFFIMNLVLW YYGSSAAIPFFTLVALLALW FGVSVPLTFVGAYFGFRKPV IEQPVRTNQIPRQIPDQSIY

Lcyp SARVYKSFGGEKWKSNVLLT SMCCPGIVFAHFFIMNLVLW YEGSSAAIPFLTLVALLALW FCVSVPLTFVGALFGFRKRA IEHPVRTNKLPRIIPEQSIY

Lsal -------------------- -------------------- -------------------- -------------------- --------------------

Aamp -------------------- -------------------- -------------------- -------------------- --------------------

Afra SARIYKSFGGERWKMNVLLT SMLCPGFIFSIFFLLNIVLW ANSSSATVPFPTLVALLALW FGISLPLTFVGAFFGFRKRG IEHPVRTNQIPRQIPEQSFY

Hazt -------------------- -------------------- -------------------- -------------------- --------------------

Meda SARIYKSFGGEKWKSNVLLT SVFCPGVVFALFFIMNLVLW YEGSSAAIPFFTLVALLALW FCVSVPLTFVGAFFGFRKRA IEHPVRTNQIPRQIPEQSIY

Afos SSRIYKSFGGEKWKSNVLLT SMLCPGIVFAHFFVMNLVLW YYGSSAAIPFFTLVALLALW FGVSVPLTFVGAYFGFRKPC IEQPVRTNQIPRQIPDQSLY

Dmel SARIYKSFGGLKWKSNVILT SIVCPGVVFSLFFVMNLVLW GENSSGAVPFSTLIALLALW FGVSVPLTFVGAYFGFRKRA LEHPVRTNQIPRQIPDQSIY

Tcas SARIYKSFGGEKWKSNVLLT SMLAPGIVFGLFFVMNLVLW SKGSSAAVPFSTLVGLLALW LLVSVPLTFVGAFFGFRKRA LEHPVRTNQIPRLIPEQSIY

Pmon -------------------- -------------------- -------------------- -------------------- --------------------

Smar SSRIYKSFGGEKWKSNVLLT SLFCPGVVFVLFFTMNLLLW GMGSSAAIPFTTLLALLALW FGISLPLTFVGAYFGFRKRI IEHPVRTNQIPRQIPEQSIY

Tcal SARIYKSFGGEKWKSNVLLT SMLCPGVVFGHFFIMNLVLW YKDSSAAIPFFTLVALLALW FGVSVPLTFVGAFFGFRKRA LEHPVRTNQIPRQIPDQSFY

Crog SARIYKSFYGEKWKSNIMLT SLLCPGVVFAHFFVMNLVLW YKGSSAAIPFFTLLGLLALW ILVCVPLTFLGSIFGFRKRS LEHPVRTNPIPRQIPEQTIY

Dpul SSRIYKSFGGERWKLNVLLT SMLCPGIVFGVFFILNLVLW SKGSSGAISFGILVALLALW FGISVPLTFVGAFFGFRKRP IEHPVRTNQIPRQVPDQSVY

Aver TQPIPGIIMGGVLPFGCIFI QLFFVLNSIWSSQI------ -------------------- -------------------- --------------------

Csin -------------------- -------------------- -------------------- -------------------- --------------------

Isca TQALPAIFMGGILPFGCIFI QLFFILNSIWSSQMYYMFGF LFLVFIILIITCSETTAPLG LWRLGTTDYHWWWRSFLTSG CTAFYLFVYCVHYF-SRLSI

Eaff TQDANLMV------------ -------------------- -------------------- HFQQCG------WR------ --------------------

Lcyp TQPIPGIIMGGVLPFGCIFI QLFFVLNSIWSSQIYYMYGF LFLVFIILIITCSETTILLC YFHLCAEDYHWWWRSFLTSG FTAFYLFIYCIHYFFTKLEI

Lsal -------------------- -------------------- -------------------- -------------------- --------------------

Aamp -------------------- -------------------- -------------------- -------------------- --------------------

Afra TKPLPGIIMGGILPFGCIFI QLFFILNSIWSNQMYYMFGF LFLVFIILVITCSETTILLC YFHLCAEDYQWWWRSFMTSG FTAFYLFAYCIHYYMTKLNI

Hazt -------------------- -------------------- -------------------- -------------------- --------------------

Meda TQPIPGIIMGGVLPFGCIFI QLFFVLNSIWSSQI------ -------------------- -------------------- --------------------

Afos TQPVPGIIMGGVLPFGCIFI QLFFVLNSIWSSHIYYMYGF LFLVYLILIITCSETTILLC YFHLCAEDYHWWWRSFLTSG FTAFYLAMYCIHYFFTKLDI

Dmel TQPIPGIVMGGVLPFGCIFI QLFFILSSLWSSQIYYMFGF LFLVFVILVITCSETTILLC YFHLCAEDYHWWWRSFLTSG FTAVYLFIYCCHYFVTKLSI

Tcas TQPIPGIVMGGVLPFGCIFI QLFFILNSIWSSQMYYMFGF LFLVFIILVITCAETTILLC YFHLCAEDYHWWWRSYLTSG FTAVYLFLYCCHYFFTKLQI

Pmon -------------------- -------------------- -------------------- -------------------- --------------------

Smar SQPLPGIV------------ -------------------- -------------------- -------------------- --------------------

Tcal TQPIPGIAMGGVLPFGCIFI QLFFVLNSIWSSQIYYMYGF LFLVFIILLITCSETTILLC YFHLCAEDYHWWWRSFLTSG FTAFYLAVYCIHYFVTKLEI

Crog TQPIPAIAMGGVLSFGCIFI QLFFVLNSIWASHIYYMYGF LFLVFIILIITCSETTILLC YFQLCAEDYHWWWRSFLTSG FTAFYLFLYCIHYFATKLEI

Dpul TRPAPGIVMGGVLPFGCIFI QLFFILNSLWSNQMYYMFGF LFLVFIILVITCSETTVLLC YFHLCAEDYHWWWRSFLTSG FTAFYLFLYCVHYFVTKLEI

Aver -------------------- -------------------- -------------------- -------DKATIEGICNTLK SADTDPKVDVVVGVSPCYLE

Csin -------------------- -------------------- -------------------- -------------------- --------------------

Isca TGLASTFLYFGYTTIIVFLF FLLTGTVGFFSCFWFVRKIY GVVKV----DM-SGRKFCVG GNWKMNGNKSSIKEICDMLK TAKLDPNTEVVLGCPAPYLD

Eaff -------------------- -------------------- ----------M--GRKFFVG GNWKMNGTKESIDKIVDFLT KGPLDPNTEVVVGVSPCYLE

Lcyp EGAASTFLYFGYTSIMVFIV FLLTGTIGFLACFWFVRKIY SVVKV----DM--GRKFFVG GNFKMNGDKASLETICSTLA KADTDKDVEVVVGVPACYLA

Lsal -------------------- -------------------- ----------MGGGRKFFVG GNWKMNGDKKSIDGIVDFLS KGDLDPNCEVVVGASPCYLD

Aamp -------------------- -------------------- -------------------- -------------------- --------------------

Afra DPGASSFLYFGYTFIMVALF FIMTGTIGFFACFWFVRKIY SVVKV----DMSAPRNFFVG GNWKLNGNKQEIGNIINFLT SGNLDPNTEVVCAPPTIYLE

Hazt -------------------- -------------------- -------------------- -------------------- ---------VVVGCPQCYLA

Meda -------------------- -------------------- -------------------- -------------------- --------------------

Afos EGAASTFLYFGYTSIMVFCF FLLTGTIGFFASFWFVRKIY SVVKV----DM--GRKFFVG GNWKMNGTKESIDKIVEFLS AGPLDPNTEVVVGCPAIYMD

Dmel KDSASTFLYFGYTAIMVFLF FLLTGTIGFFACFWFIRKIY SVVKV----DM--SRKFCVG GNWKMNGDQKSIAEIAKTLS SAALDPNTEVVIGCPAIYLM

Tcas EDTASAFLYFGYTLIMVFLF NLLTGSIGFFACFWFIRKIY SVVKV----DM--GRKFVVG GNWKMNGDKKQISEIINTLK TGPLSPNTEVIVGVPAIYLE

Pmon -------------------- -------------------- -------------------- -------------------- --------------------

Smar -------------------- -------------------- -------------------- -------------------- --------------------

Tcal EGPASTFLYFGYTSIMVFLF FLLTGTIGFFACFWFVRKIY SVVKV----DM--GRKFFVG GNWKMNGDKESMDKIIDFLK AGPLDPNCDVVVGVPAPYLM

Crog DGTASTFLYFGYTSIMVFIF FLMTGTIGFFACFWFTRKIY SVVKVKERRGMAGGRKFFVG GNWKMNGDKKSIDGIVDFLS KGDLDPNCEVVVGASPCYLD

Dpul QDATSTFLYFGYTFIMVFMF FLLSGTIGFFACFWFVRKIY SVVKV----D---------- -------------------- --------------------

Aver FVRNHLPADRFGVAAQNCYK VASGAFTGEISPMMIKDCGC DWVILGHSERRHVFGESDAL IGEKVAFALENGLHIIPCIG EKLEERESGKTEEVCFHQLK

Csin -------------------- -------------------- -------------------- -------------------- --LEERESGATMDVCYKQLT

Isca YVRRILPA-AIAVSAQNCYK VEKGAFTGEISPAMIKDCGA TWVILGHSERRNVFKESDEL IGDKVHHALESGLNVIACIG ELLEEREAGKTEEVVYRQTA

Eaff YVRSKLPQ-TIGVAAQNCYK ADSGAFTGEICPAMIKDCGC EWVILGHSERRNVFGETDQL IGEKVGFALKQGLKVVPCIG EQLEEREAGKTTEVCFRQLK

Lcyp FVRAHLPD-RFGVAAQNCYK AASGAFTGEIAPKMIEDCGC HWVILGHSERRHVFGESDAL IGDKVAFALQSGLSIIPCIG EKLEERESGKTEEVCFHQLK

Lsal YSRSKLPA-NIGVAAQNCYK VAKGAFTGEISPQMIKDVGC EWAILGHSERRNVFGESDEL IGEKVAFALESGLKIIPCIG EKLDERESGKTEEVCFKQLK

Aamp -------------------- -------------------- -------------------- -------------------- --------------------

Afra HVRKTLPS-SVGVAAQNCYK VPKGAFTGETSPAMIKDVGV EWAILGHSERRNVFGESDEL IGAKVGHALEAGLKVIPCIG EKLEERESGKTEEVVFRQLK

Hazt YSRETVPH-NIGVAAQNCYK AAKGAYTGEIAPGMIKDLGC EWVILGHSERRNVFGESDQL ISEKIGFALSEGLKVIPCFG EQLAERESGRTQEVVFSQLA

Meda -------------------- -------------------- -------------------- -------------------- --------------------

Afos YVRTKLPA-SIGVAAQNCYK AEKGAFTGETSAAMIKDVGC EWVILGHSERRNVFGESDQL VGEKVGFALQSGLKVIPCIG ELLEEREAGKTTEVCFRQLK

Dmel YARNLLPC-ELGLAGQNAYK VAKGAFTGEISPAMLKDIGA DWVILGHSERRAIFGESDAL IAEKAEHALAEGLKVIACIG ETLEEREAGKTNEVVARQMC

Tcas HVRTNLPQ-SIEVAAQNCYK VPKGAFTGEISPAMIKDIGV NWVILGHSERRQIFGETDEL IAEKVAHALEAGLKVIACIG ETLEEREGGKTEEVVFRQTK

Pmon -------------------- -------------------- -------------------- -------------------- --------------------

Smar -------------------- -------------------- -------------------- -------------------- --------------------

Tcal YCRERLPE-NVGVAAQNCHK AAKGAFTGEIAAAMIKDCGC KWVILGHSERRNVFGETDEL IGEKVAFALENGLHIIPCIG EKLEEREAGKTEEVCFKQLK

Crog YSRSKLPA-NIGVAAQNCYK AAKGAFTGEISPQMIKDVGC NWVILGHSERRNVFGESDEL IGEKVAFALESGLHIIPCIG EKLEERESGRTEEVCFKQLK

Dpul -------------------- -------------------- -------------------- -------------------- --------------------

Aver AIADKVS--DWSRVVLA--- -------------------- -------------------- -------------------- --------------------

Csin AIAGGVS--DWSRVVLAYEP VWAIGTGKTATPAQAQEVHA ACRAWLEKNVSPAVAKSTRI LYGGSVTPANCRDLAKCPDI DGSLVGGASLKPDFVQIVNA

Isca AIAAKVT--DWNRVVLAYEP VWAIGTGKTASPEQA-EVHA QLRQWLSKNVSPDVAKKVRI QYGGSVTAANCQELAKKPDV DGFLVGGASLKPEFVEIINA

Eaff AIADNVS--DWSKVVIAYEP VWAIGTGKTASPEQAQEVHA AIRNWLAENVSAEVAESTRI LYGGSVSAGNCRELAACPDI DGSLVGGASLKPDFVQIINA

Lcyp AIADKVK--DWSKVVLAYEP VWAIGTGKTATPQQAQDVHL ALRKWLSEHVSAEVSETVRI LYGGSVTAANCKELAACPDI DGFLVGGASLKPEFVNIINA

Lsal AISDKVS--DWDRVVLAYEP VWAIGTGKTATPAQAQETHL ALRKWLKENVSEEVSQKVRI LYGGSVSAGNCKELGTQPDI DGFLVGGASLKPDFVQIINA

Aamp -------------------- -------------------- -------------------- -------------------- --------------------

Afra AIADNIKPDQWQNVVIAYEP VWAIGTGKTASPAQAQEIHV KLRKWLNDNVSADVAQKTRI IYGGSVTADNCRELAREGDI DGFLVGGASLKPDFVQIVNA

Hazt AMKPNIT--DWSRVVLAYEP VWAIGTGKTATPQQAQEVHA QLRQWLRDNVSEEVANSTRI IYGGSVTAATCKELASMPDI DGFLVGGASLKPEFVAIINA

Meda -------------------- -------------------- -------------------- -------------------- --------------------

Afos AIADNVS--DWSRVVIAYEP VWAIGTGKTATPEQAQEVHS QLRTWLADNVNPEVAAATRI LYGGSVTAANCRELAACPDI DGSLVGGASLKPDFVAIANA

Dmel AYAQKIK--DWKNVVVAYEP VWAIGTGQTATPDQAQEVHA FLRQWLSDNISKEVSASLRI QYGGSVTAANAKELAKKPDI DGFLVGGASLKPEFVDIINA

Tcas AIAGKIK--DWSNVVIAYEP VWAIGTGKTATPQQAQEVHK ALRQWFCTHVNADVGNSIRI QYGGSVTGANCKELAAQPDI DGFLVGGASLKPEFVNIVNA

Pmon -------------------- -------------------- -------------------- -------------------- --------------------

Smar -------------------- -------------------- -------------------- -------------------- --------------------

Tcal AIADKVS--DWSRVVLAYEP VWAIGTGKTATPEQAQETHA ALRKWLSENVSPEVAENLRI LYGGSVTAANCKELAKCADI DGSLVGGASLKPEFVEIINA

Crog AIADKVS--DWDRVVLAYEP VWAIGTGKTATPQQAQDTHL ALRKWVKENVSEEVANKLRI LYGGSVSAGNCKELGGQADI DGFLVGGASLKPDFVQIINA

Dpul -------------------- -------------------- -------------------- -------------------- --------------------

Aver -------------------- -------------------- -------------------- -------------------- --------------------

Csin TQ------------------ -------------------- -------------------- -------------------- --------------------

Isca RQM-----PEVRSAAVASKE HALAVSRDYISQPRLVYKTV CGVNGPLVILDEVKFPKYAE IVHLVLADGTTRTGQVLEVS GSKAVVQVFEGTSGIDAKNT

Eaff TQM-----TATISIRQGLID QTLAITRDYIAEPRITYKTV SGVNGPLVILDNVKFPKFAE IVNLKLPDGSYRAGQVLEVS GSKAVVQVFEGTSGIDAKRT

Lcyp NKMTMA--APTISLRHGLVE HAMAVTRDYVAQPRITYRTV SGVNGPLVILDNVKFPKFAE IVNLQLPDGSHRAGQVLEVT GSKAVVQVFEGTSGIDAQKT

Lsal TK------------------ -------------------- -------------------- -------------------- --------------------

Aamp -------------------- -------------------- -------------------- -------------------- --------------------

Afra RQM-----NGIITHKQANNE HKLAVTRDFISQPRLSYRTV SGVNGPLVILDDVKFPKYAE IVNLRLADGSIRSGQVLEVS GNKAVVQVFEGTSGIDAKNT

Hazt KCM----------PSQGHFD QVAAVTRDYIAQPHLCYKTV TGVNGPLVILDDVKFPKFAE IVNLHLADGSERKGQVLEVS GSKAVVQVFEGTSGIDAKHT

Meda -------------------- -------------------- -------------------- -------------------- --------------------

Afos TQ------------------ --------YIAQPRITYRTV SGVNGPLVILDDVKFPKYAE IVNLNLPDGTQRAGQVLEVS GSKAVVQVFEGTSGIDAKRT

Dmel RQ---------MNAQQAQRE HVLAVSRDFISQPRLTYKTV SGVNGPLVILDEVKFPKFAE IVQLRLADGTVRSGQVLEVS GSKAVVQVFEGTSGIDAKNT

Tcas KQMSY---QNSISSKQAARE HVLAVSRDFVSQPRLTYKTV TGVNGPLVILDDVKFPKFNE IVQLKLSDGSIRSGQVLEVS GSKAVVQVFEGTSGIDAKHT

Pmon --M----------TSLGHLQ QVAAVQRDYIAQPRLCYKTV TGVNGPLVILDDVKFPKFAE IVNLHLADGTERKGQVLEVS GSKAVVQVFEGTSGVDAKHT

Smar --MASKVVARTLSGDQAFKE HTLAVSRDYISQPRLTYRTV SGVNGPLVILDDVKFPKYAE IVQLRLADGSLRSGQVLEVS GSKAVVQVFEGTSGIDAKNT

Tcal SQMSP---HPTVEIRQGMVD QFMAITRDYVAQPRLTYRTV SGVNGPLVILDNVKFPKFAE IVNLKLPDGSYRAGQVLEVT GSKAVVQVFEGTSGIDAKKT

Crog TK--------MIEIRQNKIE EIMAVTRDYIAQPRITYRTV SGVNGPLVILEDVKFPKFAE IVNLKLPDGSFRAGQVLEVS GSKAVVQVFEGTSGIDAKKT

Dpul --MAHK--TGTLNEAQARLE HKMAVNRDYIFQPRMSYRTV SGVNGPLVILDDVKFPKYAE IVNLRLHDGTIRSGQVLEVS GSKAVVQVFEGTSGIDAKNT

Aver -------------------- -------------------- -------------------- --GISAIDVMNSIARGQKIP IFSANGLPHNEIAAQICRQG

Csin ------------------RR V------------------- -------------------- -------------------- --------------------

Isca VCEFTGDILRIPVSEDMLGR VFNGSGKPIDKGPPVLAEDF LDIQGQPINPWARIYPEEMI QTGISAIDVMNSIARGQKIP IFSAAGLPHNEIAAQICRQG

Eaff TCEFTGDILRSPVSEDMLGR VFNGSGKPIDKGPSVMAEDY LDIQGQPINPWSRIYPEEMV QTGISAIDVMNSIARGQKIP IFSANGLPHNEVAAQICRQA

Lcyp TCEFTGDILRTPVSEDMLGR IFNGSGKPIDKGPNVLAEDF LDIQGQPINPYSRIYPEEMV QTGISAIDVMNSIARGQKIP IFSANGLPHNEIAAQICRQG

Lsal -------------------- -------------------- -------------------- -------------------- --------------------

Aamp -------------------- -------------------- -------------------- -------------------- --------------------

Afra ICDFTGDILRTPVSEDMLGR IFNGSGKPIDKGPSILAEEF LDIQGQPINPWSRIYPEEMI QTGVSSIDVMNSIARGQKIP IFSAAGLPHNEIAAQICRQA

Hazt VCEFTGDIMRTAVSEDMLGR VFNGSGKPIDNGPAVLAEDF LDIQGQPINPWSRTYPQEMI QTGISSIDVMNSIARGQKIP IFSAAGLPHNEIAAQICRQA

Meda -------------------- -------------------- -------------------- --GISAIDVMNSIARGQKIP IFSANGLPHNEIAAQICRQG

Afos TCEFTGDILRTPVSEDMLGR VFNGSGKPIDKGPTVMAEDY LDIQGQPINPWSRIYPEEMV QTGISAIDVMNSIARGQKIP IFSANGLPHNEVAAQICRQA

Dmel LCEFTGDILRTPVSEDMLGR VFNGSGKPIDKGPPILAEDF LDIQGQPINPWSRIYPEEMI QTGISAIDVMNSIARGQKIP IFSAAGLPHNEIAAQICRQA

Tcas VCEFTGDILRTPVSEDMLGR VFNGSGKPIDKGPPILAEDF LDIQGQPINPWSRIYPEEMI QTGISAIDVMNSIARGQKIP IFSAAGLPHNEIAAQICRQA

Pmon VCEFTGDILRTPVSEDMLGR VFNGSGKPIDQGPTVLAEDF LDIQGQPINPWSRTYPEEMI QTGISSIDVMNSIARGQKIP IFSAAGLPHNEIAAQICRQA

Smar VCEFTGDILRTPVSEDMSGR VFNGSGKPIDKGPNVLAEDY LDIQGQPINPWSRIYPEEMI QTGISAIDVMNSIARGQKIP IFSAAGLPHNEIAAQICRQG

Tcal TCEFTGDILRTPVSEDMLGR VFNGSGKPIDKGPAVMAEDY LDIQGQPINPYSRIYPEEMV QTGISAIDVMNSIARGQKIP IFSANGLPHNEIAAQICRQG

Crog TCEFTGDILRTPVSEDMLGR VFNGSGKPIDKGPNVMAEDY LDIQGQPINPYSRIYPEEMV QTGISAIDVMNSIARGQKIP IFSANGLPHNEIAAQICRQG

Dpul VCEFTGDILKSPVSEDMLGR VFNGSGKPIDKGPTVLAEDF LDIQGQPINPHSRIYPEEMI QTGISAIDIMNSIARGQKIP IFSAAGLPHNEIAAQICRQG

Aver SLV---RLPGKSVLDDHEDN FAIVFAAMGVNMETARFFKQ DFEENGSMENVCLFLNLAND PTIERIITPRLALTTAEFLA YQCEKHMLVILTDMSSYAEA

Csin ----------HGVLDDHQDN FAIVFAAMGVNMETARFFKQ DFEENGSMENVCLFLNLAND PTIERIITPRLALTAAEFMA YQCEMHMLVILTDMSSYAEA

Isca GLV---KLPGKSVLDTSDDN FAIVFAAMGVNMETARFFKQ DFEENGSMENVCLFLNLAND PTIERIITPRLALTTAEFLA YQCEKHVLVILTDMSSYAEA

Eaff GLV---RLPGKSVLDDHQDN FAIVFAAMGVNMEAARFFKQ DFEENGSMENVCLFLNLAND PTIERIITPRLALTAAEFMA YQCEMHMLVILTDMSSYAEA

Lcyp SLV---KLPGKSVLDDHEDN FAIVFAAMGVNMETARFFKQ DFEENGSMENVCLFLNLAND PTIERIITPRLALTTAEFLA YQCEKHMLVILTDMSSYAEA

Lsal -------------------- -------------------- -------------------- -------------------- --------------------

Aamp -------------------- -------------------- -------------------- -------------------- --------------------

Afra GLV---KLPGKSVLDDHEDN FAIVFAAMGVNMETARFFKL DFEENGSMENVCLFLNLAND PTIERIITPRLALTAAEYLA YQCEKHVLVILTDMSSYAEA

Hazt GLVNAAQATKGSVIDDHHDN FAIVFAAMGVNMEAARFFKQ DFEENGSMENVCLFLNLAND PTIERIITPRLALTTAEYLA YQCEKHVLIILTDMSSYAEA

Meda SLV---KLPGKSVLDDHEDN FAIVFAAMGVNMETARFFKQ DFEENGSMENVCLFLNLAND PTIERIITPRLALTTAEFLA YQCEKHMLVILTDMSSYAEA

Afos GLV---RLPGKSVLDDHQDN FAIVFAAMGVNMEAARFFKQ DFEENGSMENVCLFLNLAND PTIERIITPRLALTTAEFMA YQCEKHMLVILTDMSSYAEA

Dmel GLV---KLPGKSVLDDHTDN FAIVFAAMGVNMETARFFKQ DFEENGSMENVCLFLNLAND PTIERIITPRLALTAAEFLA YQCEKHVLVILTDMSSYAEA

Tcas GLV---KVPGKSVLDDHEDN FAIVFAAMGVNMETARFFKQ DFEENGSMENVCLFLNLAND PTIERIITPRLALTAAEFMA YQCEKHVLVILTDMSSYAEA

Pmon GLV---NLPSKGVLDDHKDN FAIVFAAMGVNMETARFFKQ DFEENGSMENVCLFLNLAND PTIERIITPRLALTTAEYLA YQCEKHVLIILTDMSSYAEA

Smar GLV---KVPGKSVLDDHSDN FAIVFAAMGVNMETARFFKQ DFEENGSMENVCLFLNLAND PTIERIITPRLALTTAEFLA YQCEKHVLVILTDMSSYAEA

Tcal SLV---RLPGKSVLDDHQDN FGIVFAAMGANMETARFFKQ DFEENGSMESTCLFLNLAND PTIERIITPRLALTTAEFLA YQCEKHMLVILTDMSSYAEA

Crog SLV---RLPGKSVLDDHKDN FAIVFAAMGVNMEAARFFKQ DFEENGSMENVCLFLNLAND PTIERIITPRLALTTAEFLA YQCEKHMLVILTDMSSYAEA

Dpul GLV---KVPGKSVLDDHDDN FAIVFAAMGVNMETARFFKQ DFEENGSMENVCLFLNLAND PTIERIITPRLALTAAEFLA YQCEKHVLVILTDMSSYAEA

Aver LREVSAAREEVPGRRGFP-- -------------------- -------------------- -------------------- --------------------

Csin LREVSAAREEVPGRRGFPGY MYTDLATIYERAGRVEGRNG SITQIPILTMPNDDITHPIP DLTGYITEGQIYVDRQLHNR QIYPPINVLPSLSRLMKSAI

Isca LREVSAARE----------- ---------ERAGRVEGRNG SITQIPILTMPNDDITHPIP DLTGYITEGQVYVDRQLHNR QVYPPINVLPSLSRLMKSAI

Eaff LREVSAAREEVPGRRGFPGY MYTDLATIYERAGRVEGRNG SITQIPILTMPNDDITHPIP DLTGYITEGQIYVDRQLHNR QIYPPINVLPSLSRLMKSAI

Lcyp LREVSAAREEVPGRRGFPGY MYTDLATIYERAGRVEGRNG SITQIPILTMPNDDITHPIP DLTGYITEGQIYVDRQMHNR QIFPPINVLPSLSRLMKSAI

Lsal -------------------- -------------------- -------------------- -------------------- --------------------

Aamp -------------------- -------------------- -------------------- -------------------- --------------------

Afra LREVSAAREEVPGRRGFPGY MYTDLATIYERAGRVEGRNG SITQIPILTMPNDDITHPIP DLTGYITEGQIYVDRQLYNR QIYPPINVLPSLSRLMKSAI

Hazt LREVSAAREEVPGRRGFPGY MYTDLATIYERAGRVEGRQG SITQIPILTMPNDDITHPIP DLTGYITEGQIYVDRQLHNR QIYPPINVLPSLSRLMKSAI

Meda LREVSAAREEVPGRRGFP-- -------------------- -------------------- -------------------- --------------------

Afos LREVSAAREEVPGRRGFPGY MYTDLATIYERAGRVEGRNG SITQIPILTMPNDDITHPIP DLTGYITEGQIYVDRQLHNR QVYPPINVLPSLSRLMKSAI

Dmel LREVSAAREEVPGRRGFPGY MYTDLATIYERAGRVEGRNG SITQIPILTMPNDDITHPIP DLTGYITEGQIYVDRQLHNR QIYPPVNVLPSLSRLMKSAI

Tcas LREVSAAREEVPGRRGFPGY MYTDLATIYERAGRVEGRNG SITQIPILTMPNDDITHPIP DLTGYITEGQIYVDRQLHNR QIYPPINVLPSLSRLMKSAI

Pmon LREVSAAREEVPGRRGFPGY MYTDLATIYERAGRVEGRQG SITQIPILTMPNDDITHPIP DLTGYITEGQIYVDRQLHNR QIYPPINVLPSLSRLMKSAI

Smar LREVSAAREEVPGRRGFPGY MYTDLATIYERAGRVEGRNG SITQIPILTMPNDDITHPIP DLTGYITEGQIYVERQLHNR QIYPPINVLPSLSRLMKSAI

Tcal LREVSAAREEVPGRRGFPGY MYTDLATIYERAGRVEGRNG SITQIPILTMPNDDITHPIP DLTGYITEGQIYVDRQLHNR QIYPPINVLPSLSRLMKSAI

Crog LREVSAAREEVPGRRGFPGY MYTDLATIYERAGRVEGRNG SITQIPILTMPNDDITHPIP DLTGYITEGQIYVDRTMHNR QIYPPINVLPSLSRLMKSAI

Dpul LREVSAAREEVPGRRGFPGY MYTDLATIYERAGRVEGRNG SITQIPILTMPNDDITHPIP DLTGYITEGQIYVDRQLHNR QIYPPINVLPSLSRLMKSAI

Aver -------------------- -------------------- -------------------- -------------------- --------------------

Csin GEGMTRKDHSDVSNQLYACY AIGKDVQAMKAVVGEEALTA DDLLYLEFLGKFEKNFIAQG NYENRTVFETLDIGWQLLRI FPKEMLKRIPAATLAEFYPR

Isca GEGMTRKDHADVSNQLYACY AIGKDVQAMKAVVGEEALSP EDLLFLEFLGKFEKNFISQG SYENRTIFESLDIGWQLLRI FPKEMLKRIPQALLQEFYPR

Eaff GEGMTRKDHSDVSNQLYACY AIGKDVQAMKAVVGEEALTP DDLLYLEFLGKFEKNFIAQG SYENRTVFESLDIGWQLLRI FPKEMLKRIPTSILAEFYPR

Lcyp GEGMTRKDHSDVSNQLYACY AIGKDVQAMKAVVGEEALTQ DDLLYLEFLGKFEKNFIAQG RYENRTVFESLDIGWQLLRI FPKEMLKRIPANVLAEFYPR

Lsal -------------------- -------------------- -------------------- -------------------- --------------------

Aamp -------------------- -------------------- -------------------- -------------------- --------------------

Afra GEGMTRKDHSDVSNQLYACY AIGKDVQAMKAVVGEEALTP DDLLYLEFLTKFEKNFISQG SYENRTVFESLDIGWQLLRI FPKEMLKRIPASVLAEYYPR

Hazt GEGMTRRDHSDVSNQLYACY AIGKDLQAMKAVVGEEALTS DDLLYLEFLAKFEKNFLTQS SYSKRSVFESLDIGWQLLRI FPKEMLKRIPASTLAEFYPR

Meda -------------------- -------------------- -------------------- -------------------- --------------------

Afos GEGMTRKDHSDVSNQLYACY AIGKDVQAMKAVVGEEALTP DDLLYLEFLAKFEKSFITQG SYENRTVFESLDIGWQLLRI FPKEMLKRIPASILAEYYPR

Dmel GEGMTRKDHSDVSNQLYACY AIGKDVQAMKAVVGEEALTP DDLLYLEFLTKFEKNFISQG NYENRTVFESLDIGWQLLRI FPKEMLKRIPASILAEFYPR

Tcas GEGMTRKDHSDVSNQLYACY AIGKDVQAMKAVVGEEALTP DDLLYLEFLSKFEKNFITQG SYENRTVFESLDIGWQLLRI FPKEMLKRIPAATLAEFYPR

Pmon GEGMTRKDHSDVSNQLYACY AIGKDLQAMKAVVGEEALTS DDLLYLEFLAKFEKTFISQG PYMKRTIFESLDIGWQLLRI FPKEMLKRIPAATLAEFYPR

Smar GEGMTRKDHADVSNQLYACY AIGKDVQAMKAVVGEEALGP DDLLYLEFLSKFEKNFIAQG NYENRTVFESLNIGWQLLRI FPKEMLKRIPQATLAEFYPR

Tcal GEGMTRKDHSDVSNQLYACY AIGKDVQAMKAVVGEEALTP DDLLYLEFLSKFEKNFIAQG SYENRTVFESLDIGWQLLRI FPKEMLKRIPASTLAEFYPR

Crog GEGMTRKDHSDVSNQLYACY AIGKDVQAMKAVVGEEALTP DDLLYLEFLTKFEKNFIAQG NYENRSVFESLDIGWQLLRI FPKEMLKRIPASTLAEFYPR

Dpul GEGMTRKDHSDVSNQLYACY AIGKDVQAMKAVVGEEALTP DDLLYLEFLTKFEKNFIAQG NYENRTVFESLDIGWQLLRI FPKEMLKRIPASVLAEFYPR

Aver ----------

Csin DSRH------

Isca DGRQGADAKQ

Eaff DSRH------

Lcyp DARH------

Lsal ----------

Aamp ----------

Afra DARGRHNE--

Hazt DRPQ------

Meda ----------

Afos DARH------

Dmel DSRH------

Tcas DSRH------

Pmon DRPQ------

Smar DSRAT-----

Tcal DARH------

Crog DSRH------

Dpul DARQGK----

;

END;
