# Supplementary material for: Phylogenomic analysis of Copepoda (Arthropoda, Crustacea) reveals unexpected similarities with earlier proposed morphological phylogenies
Source: BMC Evol Biol. 2017 Jan 19;17:23. doi: 10.1186/s12862-017-0883-5 (PMC5244711; doi:10.1186/s12862-017-0883-5)
Supplement: Additional file 5: — This file contains the data matrix of 54 morphological characters from Ho et al. [13] in Nexus format. (DOCX 17 kb) [file 12862_2017_883_MOESM5_ESM.docx]

#NEXUS

[Data from Ho JS, Dojiri M, Gordon H, Deets GB. 2003, A New Species of Copepoda (Thaumatopsyllidae) Symbiotic with a Brittle star from California, U.S.A., and Designation of a New Order Thaumatopsylloida. J Crustacean Biol 23:582-594]

BEGIN DATA;

DIMENSIONS NTAX=11 NCHAR=54;

FORMAT datatype=standard missing=?;

[Morphological Data]

MATRIX

Siphonostomatoida 11111 11112 11110 ??111 2???1 ???12 11111 11101 11111 11111 1111

Monstrilloida 11111 11112 111?0 ????? ????? ????? 11111 11111 11111 11111 111?

Thaumatopsyllidae 01111 11112 111?0 ????? ????? ????? 11111 11211 1?111 1?111 1111

Cyclopoida 01111 01112 11101 ??010 21110 11111 11111 11101 11010 0?100 1001

Poecilostomatoida 01111 11122 11110 ??111 2???1 ???11 11111 11111 11011 1?110 1111

Gelyelloida 01111 ?1112 11101 11110 21110 11111 11??1 1???? ????? ????? ????

Mormonilloida 01111 ?1112 11110 10110 21000 ???10 111?1 11?22 22??? ????? ????

Misophrioida 01110 01012 01001 11110 11000 10000 11111 11101 11010 10100 1011

Harpacticoida 01111 11112 01010 11110 11000 10001 11111 11111 11011 10110 1010

Calanoida 00110 01011 00100 00000 10000 00000 11111 11100 00000 00100 0000

Platycopioida 00000 00000 01000 00110 0???0 ??011 00000 000?0 00000 10000 0010

;

END;
