# Supplementary material for: Phylogenomic analysis of Copepoda (Arthropoda, Crustacea) reveals unexpected similarities with earlier proposed morphological phylogenies
Source: BMC Evol Biol. 2017 Jan 19;17:23. doi: 10.1186/s12862-017-0883-5 (PMC5244711; doi:10.1186/s12862-017-0883-5)
Supplement: Additional file 6: — Figure S1. Bayesian phylogenetic analysis of copepod orders with morphological characters taken from Ho et al. [13]. (DOCX 63 kb) [file 12862_2017_883_MOESM6_ESM.docx]

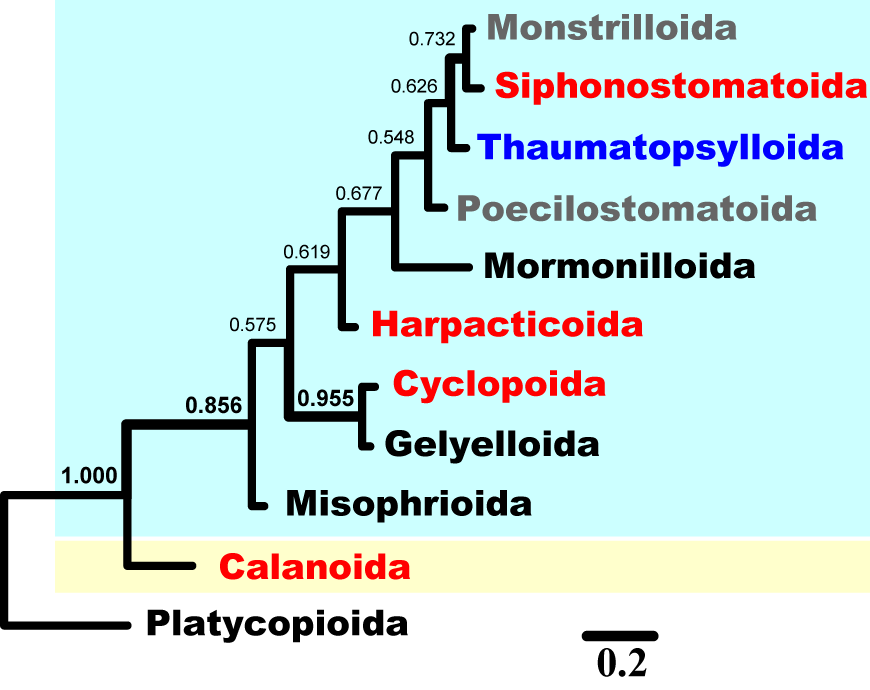


**Figure S1. Bayesian analysis of copepod orders with morphological characters adopted from Ho et al. (2003).** The data matrix of morphological characters adopts from Ho et al. (2003). The numbers above the branches show the posterior probability (boldfaces when *P* > 0.80) for the Bayesian phylogeny. The scale bar represents the expected state changes per character. The order Platycopioida is used as the outgroup in this analysis. Cyan and yellow boxes indicate the superorders, Podoplea and Gymnoplea. A new order, Thaumatopsylloida (indicated by blue) is proposed by Ho et al. (2003). Poecilostomatoida and Monstrilloida (indicated by grey) are considered as the subgroup of Cyclopoida and Siphonostomatoida, respectively (Boxshall and Halsey 2004; Huys et al. 2006; Huys et al. 2007). Four copepod orders (indicated by red) are examined in this study.

**References**

Boxshall GA, Halsey SH. 2004. An Introduction to Copepod Diversity: The Ray Society, London.

Ho J-S, Dojiri M, Gordon H, Deets GB. 2003. A New Species of Copepoda (Thaumatopsyllidae) Symbiotic with a Brittle star from California, U.S.A., and Designation of a New Order Thaumatopsylloida. *J Crustacean Biol*. 23:582-594.

Huys R, Llewellyn-Hughes J, Conroy-Dalton S, Olson PD, Spinks JN, Johnston DA. 2007. Extraordinary host switching in siphonostomatoid copepods and the demise of the Monstrilloida: integrating molecular data, ontogeny and antennulary morphology. *Mol Phylogenet Evol*. 43:368-378.

Huys R, Llewellyn-Hughes J, Olson PD, Nagasawa K. 2006. Small subunit rDNA and Bayesian inference reveal *Pectenophilus* *ornatus* (Copepoda *incertae sedis*) as highly transformed Mytilicolidae, and support assignment of Chondracanthidae and Xarifiidae to Lichomolgoidea (Cyclopoida). *Biol J Linnean Soc*. 87:403-425.
