# Supplementary material for: Phylogenomic analysis of Copepoda (Arthropoda, Crustacea) reveals unexpected similarities with earlier proposed morphological phylogenies
Source: BMC Evol Biol. 2017 Jan 19;17:23. doi: 10.1186/s12862-017-0883-5 (PMC5244711; doi:10.1186/s12862-017-0883-5)
Supplement: Additional file 7: — Figure S2. Bayesian phylogeny using the “degen-1” nucleotide coding sequences. (DOCX 172 kb) [file 12862_2017_883_MOESM7_ESM.docx]

**
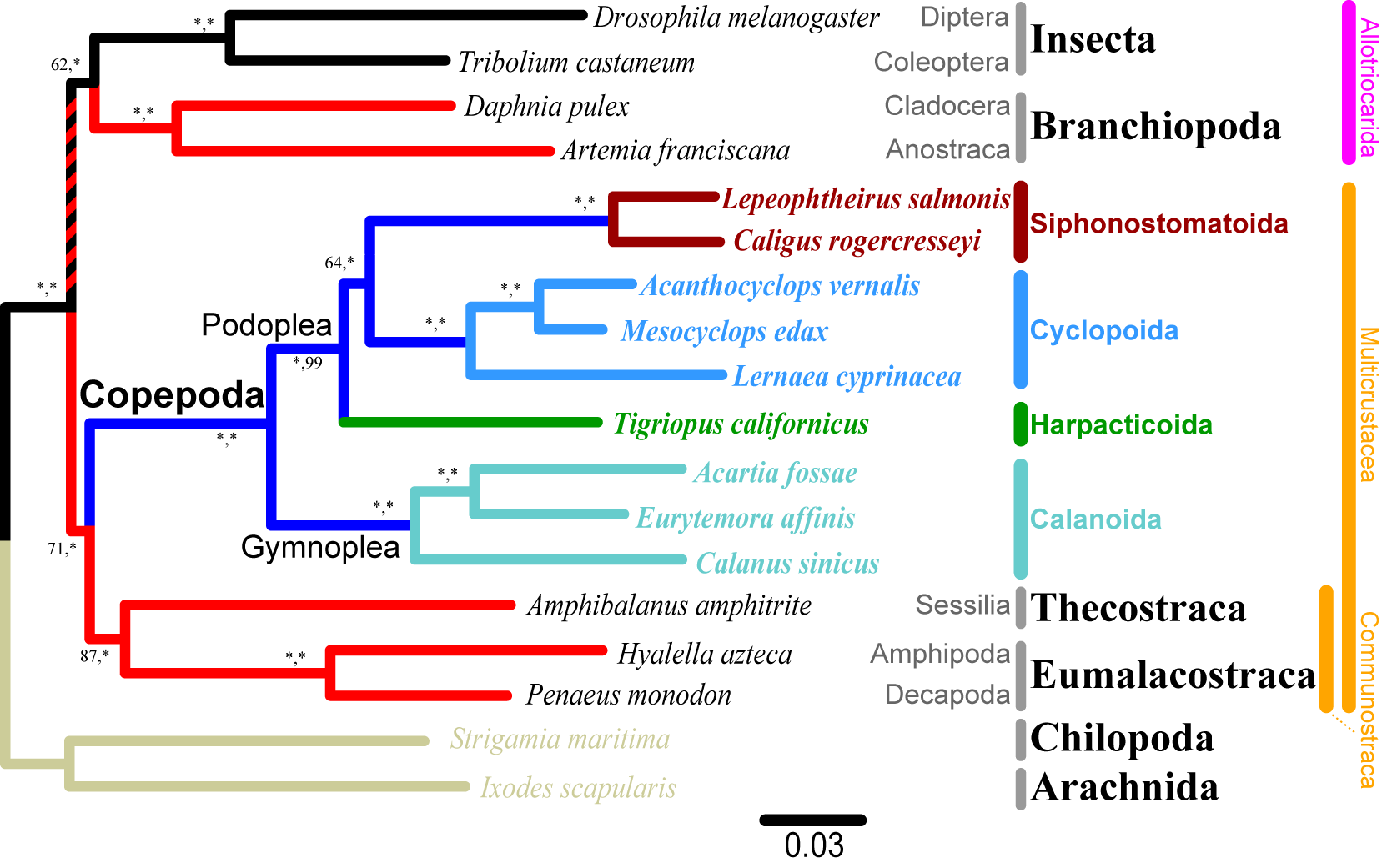
**

**Figure S2. Bayesian phylogeny using the “degen-1” nucleotide coding sequences.** To produce the degenerated synonymous matrices (“degen-1” coding sequences) (Regier *et al.* 2010), the Perl script, Degen_v1_4.pl, is used (http://www.phylotools.com). *Strigamia* *maritima* (Myriapoda) and *Ixodes* *scapularis* (Chelicerata) are used as the outgroups (indicated by light olive). Blue-colored and red-colored branches indicate the copepod groups and all other crustaceans. The numbers at internal branches show the bootstrap support values (%) for the maximum-likelihood phylogeny and the posterior probability (%) for the Bayesian phylogeny. Asterisks indicate bootstrap values of 100%. The scale bar represents the number of amino acid substitutions per site. The new proposed taxonomic classifications from Regier *et al.* (2010) and Oakley *et al.* (2013) are marked by the orange and magenta bars.

**References**

Oakley TH, Wolfe JM, Lindgren AR, Zaharoff AK (2013) Phylotranscriptomics to Bring the Understudied into the Fold: Monophyletic Ostracoda, Fossil Placement, and Pancrustacean Phylogeny. *Molecular Biology and Evolution* **30**, 215-233.

Regier JC, Shultz JW, Zwick A*, et al.* (2010) Arthropod relationships revealed by phylogenomic analysis of nuclear protein-coding sequences. *Nature* **463**, 1079-1083.
