# Supplementary material for: Phylogenomic analysis of Copepoda (Arthropoda, Crustacea) reveals unexpected similarities with earlier proposed morphological phylogenies
Source: BMC Evol Biol. 2017 Jan 19;17:23. doi: 10.1186/s12862-017-0883-5 (PMC5244711; doi:10.1186/s12862-017-0883-5)
Supplement: Additional file 8: — Figure S3. Bayesian phylogeny of nine copepod species with two outgroups. (DOCX 107 kb) [file 12862_2017_883_MOESM8_ESM.docx]

**
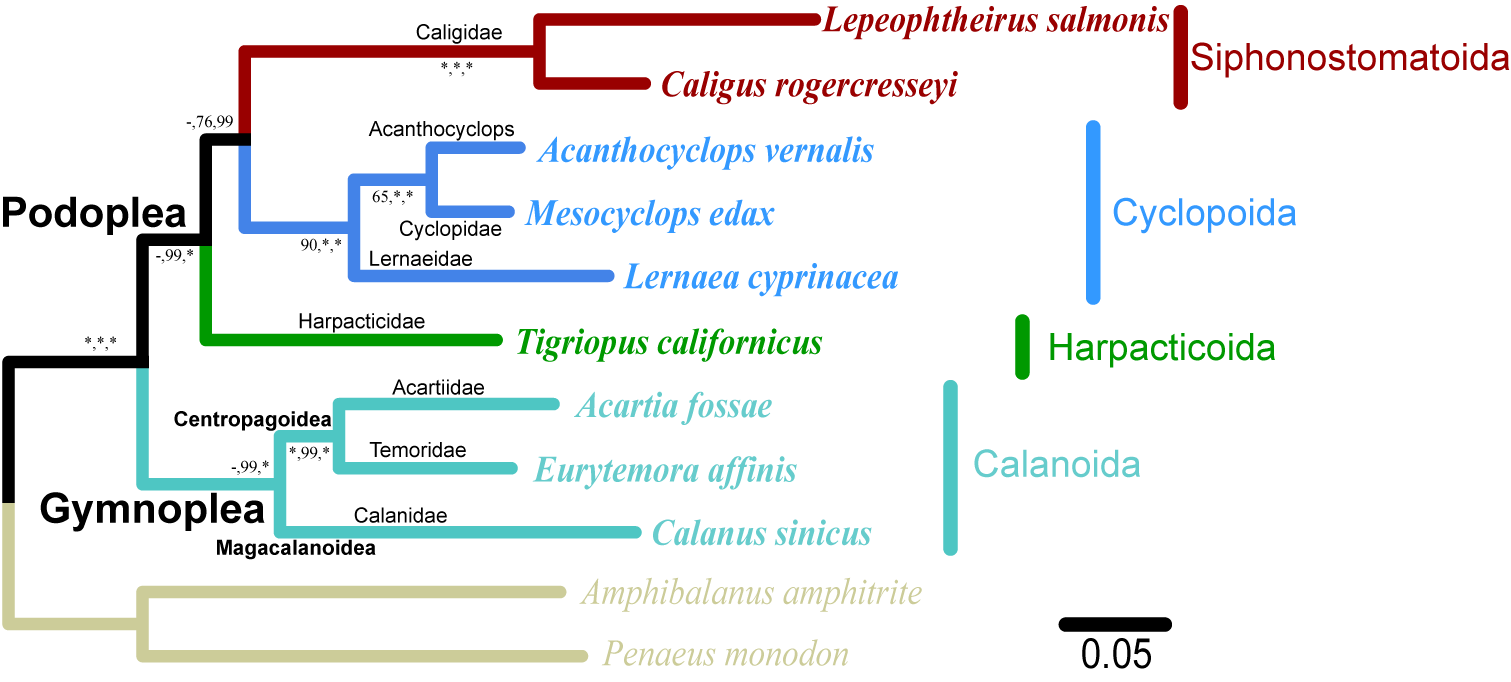
**

**Figure S3. Bayesian phylogeny of nine copepod species with two outgroups.** *Amphibalanus* *amphitrite* (Sessilia) and *Penaeus* *monodon* (Decapoda) are used as the outgroups (indicated by light olive). The numbers at internal branches show the bootstrap support values (%) for the neighbor-joining and maximum-likelihood phylogenies and the posterior probability (%) for the Bayesian phylogeny in this order. Asterisks indicate bootstrap values of 100%. Supporting values are shown only when higher than 60%. The scale bar represents the number of amino acid substitutions per site.
