# Supplementary material for: Phylogenomic analysis of Copepoda (Arthropoda, Crustacea) reveals unexpected similarities with earlier proposed morphological phylogenies
Source: BMC Evol Biol. 2017 Jan 19;17:23. doi: 10.1186/s12862-017-0883-5 (PMC5244711; doi:10.1186/s12862-017-0883-5)
Supplement: Additional file 9: — Figure S4. Maximum-likelihood phylogenies of arthropods focused on copepod species, based on a single gene region from A) 6-phosphogluconate dehydrogenase, B) carbamoylphosphate synthetase, and C) alanyl-tRNA synthetase. (DOCX 185 kb) [file 12862_2017_883_MOESM9_ESM.docx]

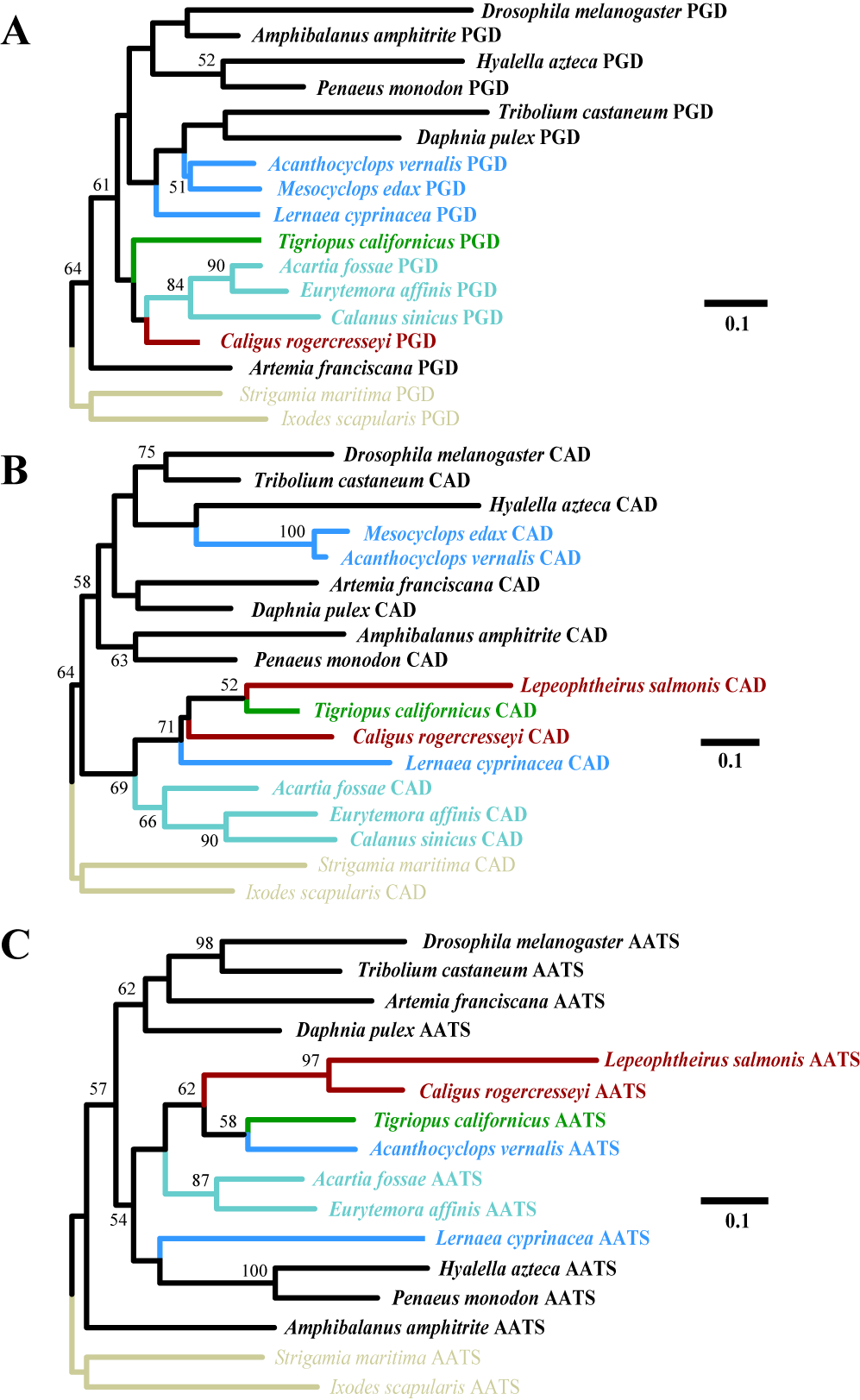


**Figure S4. Maximum-likelihood phylogenies of arthropods focused on copepod species, based on the single gene region from A) 6-phosphogluconate dehydrogenase, B) carbamoylphosphate synthetase, and C) alanyl-tRNA synthetase.** *Strigamia* *maritima* (Myriapoda) and *Ixodes* *scapularis* (Chelicerata) are used as the outgroups. The numbers at internal branches show the bootstrap support values (%) for the maximum-likelihood phylogeny. Supporting values are shown only when higher than 50%. The scale bar represents the number of amino acid substitutions per site. All single-gene phylogenetic trees are available in: http://bioinformatics.unl.edu/eyun/Copepoda_Phylogenomics.
